# Synthesis and characterization of a novel phosphatidylinositol 5-phosphate (PI(5)P) photoaffinity probe

**DOI:** 10.1039/d6cb00148c

**Published:** 2026-06-01

**Authors:** Glen Brodie, Ahmed Sayed, Sarah Kreuz, Wolfgang Fischle, Stuart J. Conway

**Affiliations:** a Department of Chemistry, Chemistry Research Laboratory, University of Oxford Mansfield Road Oxford OX1 3TA UK stuartconway@ucla.edu; b Department of Chemistry & Biochemistry, University of California Los Angeles 607 Charles E. Young Drive East Los Angeles California 90095 USA; c Bioscience Program, Biological and Environmental Science and Engineering Division, King Abdullah University of Science and Technology (KAUST) Thuwal 23955 Kingdom of Saudi Arabia; d Chemistry Department, Faculty of Science, Assiut University Assiut 71516 Egypt

## Abstract

Phosphatidylinositol 5-phosphate (PI(5)P) plays a crucial role in cellular signaling, cell proliferation, the DNA damage repair response, and gene transcription. However, the underlying mechanism of PI(5)P function in these cellular pathways is poorly understood. This lack of understanding results at least in part, from the dearth of available chemical tools to enable the investigation of PI(5)P interaction with target proteins in the corresponding biological systems. Here, we report the design and synthesis of a novel phosphatidylinositol 5-phosphate-based photoaffinity probe. The probe bound and photo-crosslinked to purified, recombinant hUHRF1 and TAF1 proteins that are known PI(5)P-interacting factors. Copper-catalyzed azide-alkyne cycloaddition (CuAAC) click chemistry with an azide-functionalized TAMRA dye allowed visualization of these proteins. We further show that the PI(5)P photoaffinity probe was functional in complex cell lysate by demonstrating protein crosslinking and fluorescent visualization with a TAMRA-azide. The data presented here validate the novel photoaffinity probe as a molecular tool for analyzing interactions and mapping the PI(5)P interactome.

## Abstract 2

A novel PI(5)P photoaffinity probe was synthesized. The probe was characterized in copper-catalyzed alkyne-azide click reactions with the known PI(5)P nuclear binding proteins hUHRF1 and hTAF3 using dyes and affinity tags.

## Graphical abstract

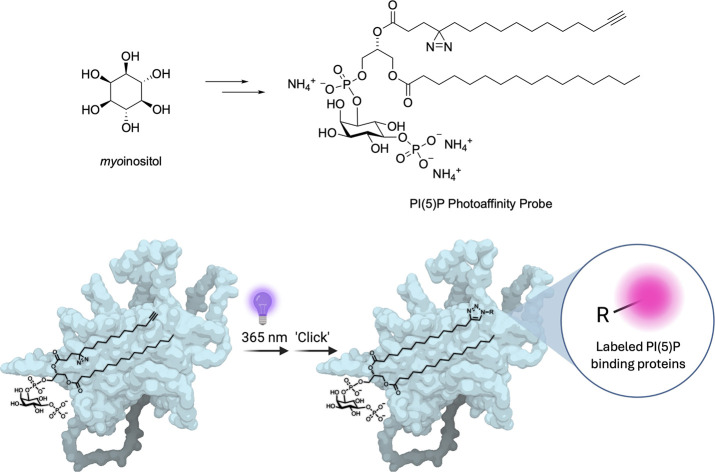

## Introduction

Phosphatidylinositol 5-phosphate (PI(5)P) is one of seven essential membrane phospholipids (phosphatidylinositols/PIs) and is thought to play a crucial role in cellular signaling, cell proliferation, the DNA damage repair response, and gene transcription.^1^ In comparison to other members of the PI family, PI(5)P exists in low concentrations, and is usually found in the membranes of cellular compartments including in the cell nucleus,^2,3^ the Golgi apparatus,^4^ in lysosomes,^5^ and in mitochondria.^6^ Despite PI(5)P being discovered over 25 years ago,^7^ the metabolism and function of PI(5)P are still poorly understood. Chemical probes capable of revealing the cellular PI(5)P interactome are scarce,^8^ and so any additional probes have the potential to greatly enhance our understanding of the biological functions of PI(5)P. Within the literature, there has been great strides in elucidating lipid-protein interactions, as well as other PIP interactions through the use of photoaffinity probes or photo-caged lipid species, highlighting the utility of such scientific approaches.^9–12^

Besides their membrane directed functions, phosphoinositides are known to interact with soluble nuclear proteins. For example, an X-ray crystal structure of PI(3,4,5)P_3_ bound to the nuclear protein SF-1 (steroidogenic factor-1) revealed that the acyl groups of the phosphoinositide are bound within the protein, rather than to the membrane. The charged inositol headgroup is solvent exposed, creating an enhanced binding surface for co-factors, that increases the activity of SF-1.^13^ PI(5)P seems particularly important in controlling nuclear processes, as it has been found to interact with and control the activity of different transcriptional and chromatin regulators. PI(5)P has, for example, been reported to modulate p53 acetylation and apoptosis by ING2 (inhibitor of growth family member 2),^14^ inhibit the E3 ubiquitin ligase activity of Cul3 (Cullin 3),^15^ and alter transcription mediated by TAF3 (TATA box binding protein associated factor 3).^16^

**Fig. 1:**
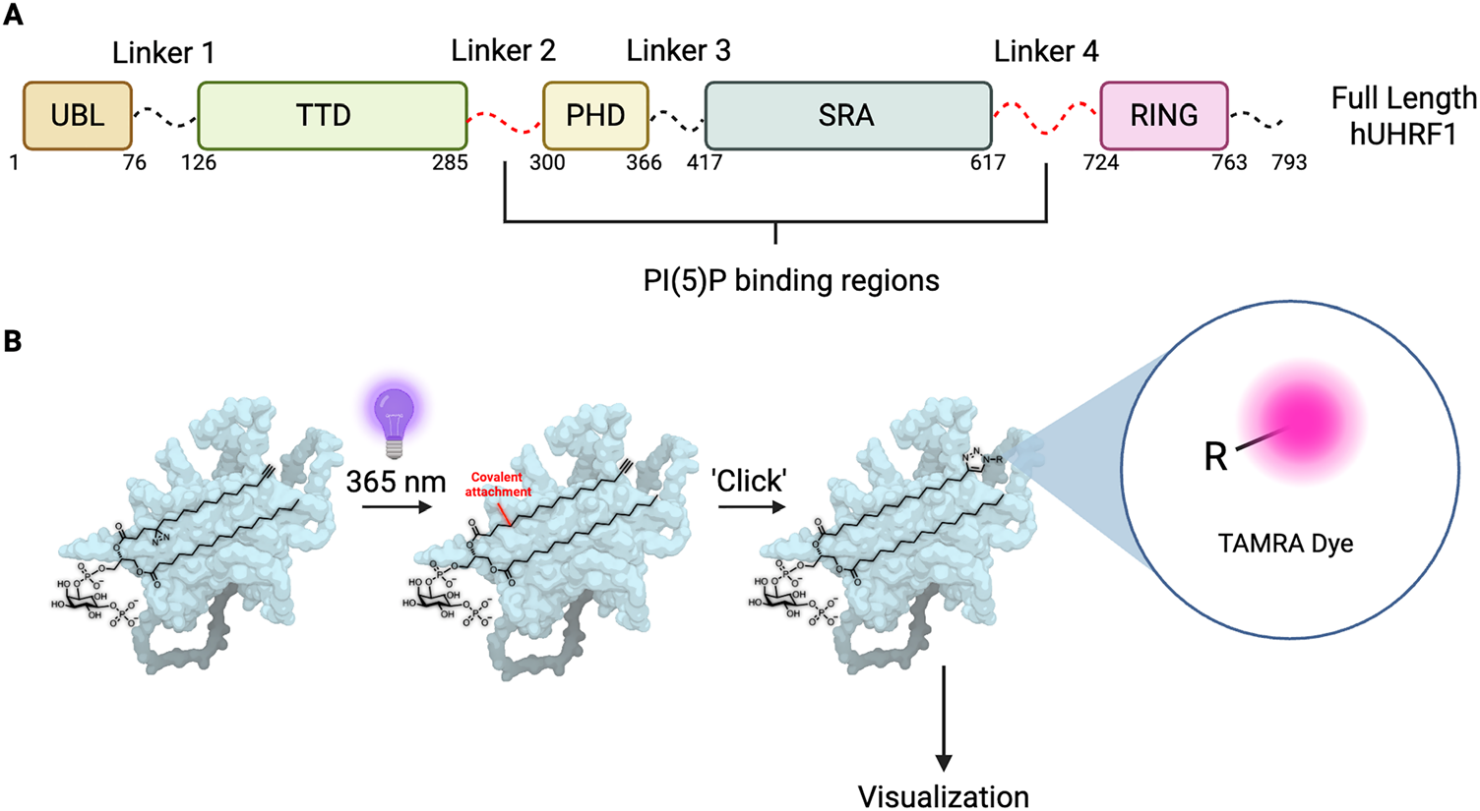
(A) Domain structure of hUHRF1, highlighting unstructured Linkers 2 and 4 as the binding regions for PI(5)P. (B) The concept of a photo-affinity PI(5)P probe being used to visualize cross-linked proteins.

Of particular interest to us is the role that PI(5)P plays as an allosteric activator of hUHRF1 [ubiquitin-like with plant homeodomain (PHD) and really interesting new gene (RING) finger domains 1].^17–19^ hUHRF1 is a fundamental epigenetic regulator that protects the genome by regulating DNA methylation levels, recognizing and binding damaged DNA, and recruiting DNA repair factors.^20^ hUHRF1 comprises five domains connected *via* flexible linker regions (Fig. 1A). These five domains are: the ubiquitin-like domain (UBL), which directs E3 ligase activity of hUHRF1 towards the N-terminal tail of histone H3 in chromatin;^21,22^ the Tandem Tudor Domain (TTD), which recognizes methylation at lysine 9 (K9Me) of the N-terminal tail of histone H3;^23^ the plant homeodomain (PHD), which recognizes the unmodified very N-terminal residues of histone H3 (unmodified arginine 3, H3Rme0);^24^ the SET- and RING-associated (SRA) domain, which binds hemi-methylated DNA;^25,26^ and the RING domain, which catalyzes ubiquitination of lysine 18 (H3K18) and/or lysine 23 (H3K23) on histone H3 (Fig. 1A).^27^ Recently, we have shown that PI(5)P interacts simultaneously with two distant flexible linker regions of hUHRF1 (Linkers 2 and 4) connecting distinct domains of the protein (Fig. 1A). This interaction enhances the binding of hUHRF1 to H3K9me3, however, the exact mechanistic details of how PI(5)P regulates the function of hUHRF1 are yet to be fully explored.^17^

To enable further understanding of the nuclear biology of PI(5)P, and analyze how PI(5)P interacts with hUHRF1, we aimed to develop a phosphatidylinositol 5-phosphate-based photoaffinity probe, by utilizing a standard photoaffinity labelling (PAL) strategy. To achieve this goal, we aimed to synthesize a probe that incorporates both a photoaffinity component for protein crosslinking, and an alkyne click tag to enable visualization or affinity purification strategies (Fig. 1B). Diazirine–alkyne photo-crosslinkers have been used extensively within the literature, enabling target identification/proteomic profiling, photoaffinity-based fragment screening and ligand mapping.^28–34^ In addition, diazirine-alkyne probes have proven to be tremendously useful in profiling lipid-protein and membrane interactions,^10,12,35–37^ with Schultz *et al.*,^38,39^ having previously applied this strategy towards lipids and inositides, allowing for visualization of their interactions within the cell. In particular, by using diazirine-alkyne inositol photoaffinity probes of PI(3,4)P_2_ and PI(3,4,5)P_3_, Schultz *et al.* found that lipid binding proteins ATP11A and MPP6 are involved in the transport of PI(3,4,5)P_3_ to the plasma membrane,^11^ highlighting the applicability of this strategy in elucidating the cellular functions of phosphoinositides.

As we have previously shown that small changes in the lipid chain conformation of PI(5)P can abolish binding to hUHRF1,^17^ we contemplated which photoactivatable functional groups and click tag to employ, and where they should be placed to minimize disruptions of the PI(5)P-protein interaction. We selected a diazirine-based photo crosslinker, as it is the smallest light activatable group, and an alkyne as the click tag. The alkyne was incorporated at the end of the acyl chain, without altering the length of the chain, keeping it to a length of 16 carbons, with the aim to minimally perturb protein binding. For the location of the diazirine within the acyl chain, we opted for a location closer to the inositol head group, rather than towards the terminal alkyne, rationalizing that we would be able to capture more protein interactions, as opposed to membrane interactions. This idea was corroborated by Schultz *et al.*,^40^ in which they highlighted the profound effect the position of the diazirine on the lipid chain can have on binding, suggesting optimization may be required in future.

Building upon our recent development of an isotope-enriched PI(5)P probe, here we present a synthesis of two PI(5)P analogs, and a synthesis of a PI(5)P photoaffinity probe, which will be a valuable tool for furthering our understanding the biological functions of PI(5)P.^41^

### Design of the PI(5)P probe

**Fig. 2:**
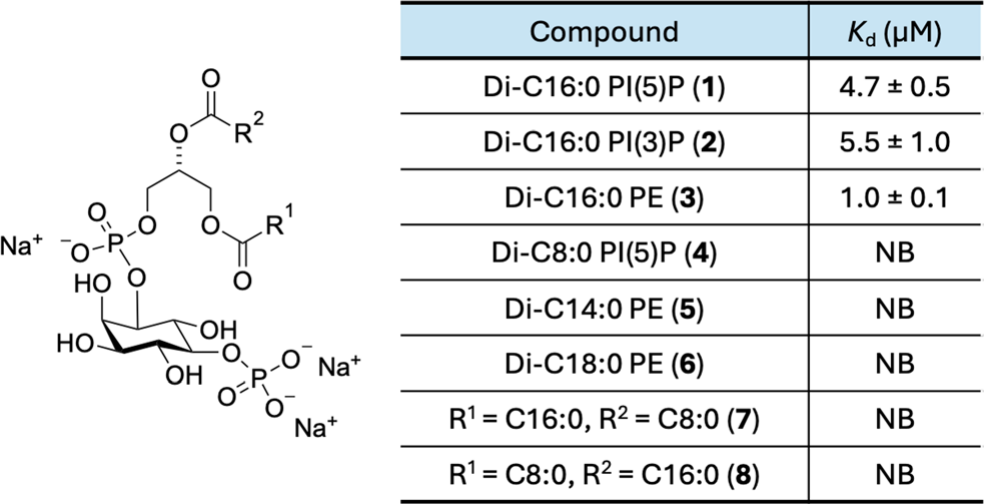
Dissociation constants of the phospholipid shown for purified hUHRF1, measured using microscale thermophoresis. *K*_d_ values for compounds 1–6 were obtained from Mandal *et al.*^17^ PE = Phosphatidylethanolamine. NB = no binding.

To aid the design of our probes, we first sought to determine whether both palmitic glyceride chains (di-C16 : 0 PI(5)P 1) are required for binding of PI(5)P to hUHRF1. Previously, it has been shown that the inositol head group alone (inositol 1,5-bisphosphate (1,5-IP)), is not enough for binding to hUHRF1.^17^ Other PI(5)P derivatives with unsaturated acyl chains did also not bind recombinant hUHRF1, implying hUHRF1 specifically requires the di-C16 acyl chains. In addition to this, di-C16 : 0 PI(3)P 2 and di-C16 : 0 PE 3, did bind hUHRF1, while di-C8 : 0 PI(5)P 4, di-C14 : 0 PE 5, di-C18 : 0 PE 6 did not (Fig. 2).^17^ However, the ability of hUHRF1 to distinguish between molecules containing a single C16 : 0 lipid, in addition to another lipid chain of varying lengths, has not been determined. To investigate this, we synthesized two PI(5)P derivatives, 7 and 8 (Fig. 2), containing a mixture of C16 : 0 and C8 : 0 acyl chains. The synthetic details can be found in the supplementary information (Scheme S1). Microscale thermophoresis (MST) analysis of biding to hUHRF1 confirmed that the di-C16 : 0 chains were required for protein affinity, as neither C16 : 0, C8 : 0 PI(5)P 7, nor C8 : 0, C16 : 0 PI(5)P 8 showed significant binding to hUHRF1 (Fig. S2).

**Fig. 3:**
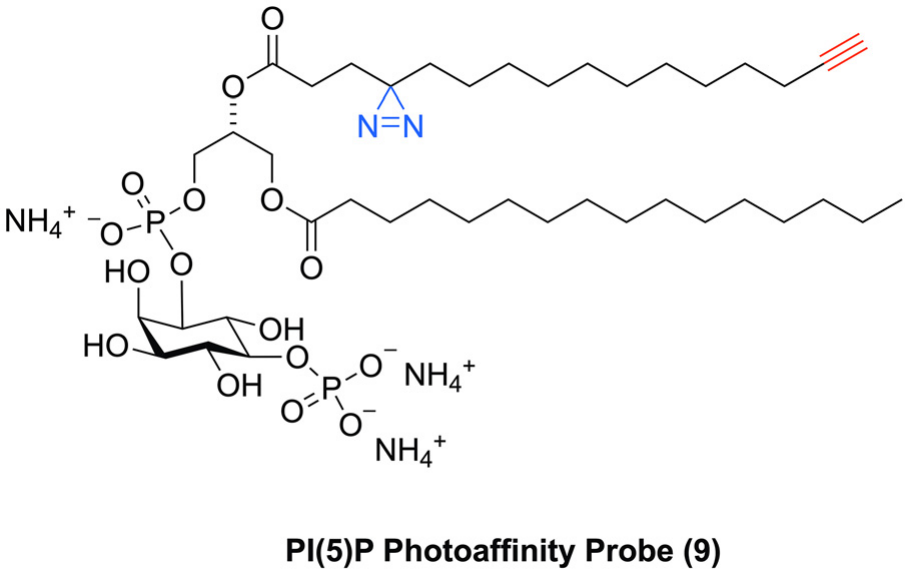
Structure of phosphatidylinositol 5-phosphate-based photoaffinity probe (9).

These data confirmed that the development of a functionalized PI(5)P probe for hUHRF1 requires minimizing any changes to the structure of the C16 chains. The design of the probe 9 (Fig. 3), therefore, incorporates the diazirine, terminal alkyne, and chain length of 16 carbons, minimizing the digression from the parent PI(5)P structure.

**Scheme 1:**
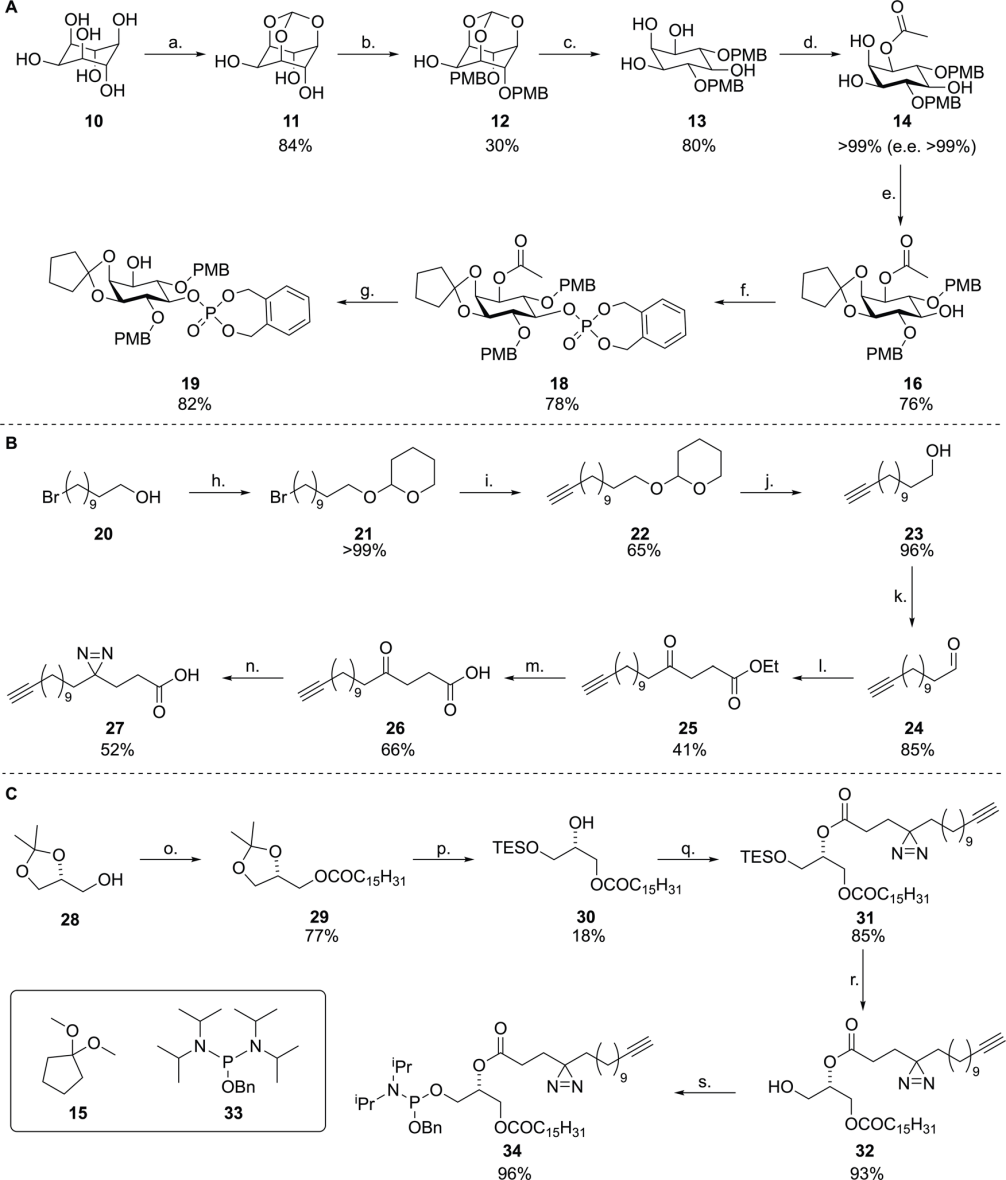
Synthesis of the PI(5)P (9) precursors. Reagents and conditions: A Synthesis of inositol core; (a) Triethylorthoformate, PTSA·H_2_O, DMF, 110 °C, 84% of 11; (b) NaH, PMBCl, DMF, 0 °C to rt, 18 h, 30% of 12; (c) 2 M HCl_(aq)_, MeOH/H_2_O, 45 °C, 5 h, 80% of 13; (d) Lipozyme TL-IM®, vinyl acetate:hexane (1 : 1), 45 °C, 18 h, >99% of 14, (>99% e.e.); e. 1,1-Dimethoxycyclopentane 15, PTSA·H_2_O, CH_2_Cl_2_, 76% of 16; (f). (i) *N*,*N*-Diethyl-1,5-dihydrobenzo[e][1,3,2]dioxaphosphepin-3-amine 17, 1*H*-tetrazole, CH_2_Cl_2_; (ii) *m*CPBA, 78% of 18; g. K_2_CO_3_, MeOH, 82% of 19; B Synthesis of alkyne/diazirine; (h) 3,4-Dihydro-2*H*-pyran, pyridinium 4-toluene sulfonate, CH_2_Cl_2_, rt, 18 h, >99% of 21; (i) Lithium acetylide (ethylenediamine complex), DMSO, 24 h, 65% of 22; (j) 2 M HCl_(aq)_, EtOH, reflux, 18 h, 96% of 23; (k) Oxalyl chloride, DMSO, Et_3_N, CH_2_Cl_2_, −78 °C to rt, 5 h, 85% of 24; (l) Ethyl acrylate, 3-benzyl-5-(2-hydroxyethyl)-4-methylthiazolium chloride, Et_3_N, 1,4-dioxane, 80 °C, 56 h, 41% of 25; m. LiOH, H_2_O, MeOH, rt, 18 h, 66% of 26; (n). (i) NH_3_ (7 M in methanol), hydroxylamine-*O*-sulfonic acid, (ii) Et_3_N, I_2_ 52% of 27; C Synthesis of di-palmitoyl diazirine phosphoramidite; o. palmitic acid, DCC, DMAP, CH_2_Cl_2_, rt, 16 h, 77% of 29; p. (i) TESOTf, DIPEA, reflux, 16 h; (ii) 10% aq. NaHCO_3_, I_2_, rt, 3 h, 18% of 30; (q) DCC, DMAP, CH_2_Cl_2_, rt, 16 h, 85% of 31; (r). FeCl_3_, MeOH/CH_2_Cl_2_ (3 : 1), rt, 3 h, 93% of 32; s. Phosphoramidite 33, 1*H*-tetrazole, CH_2_Cl_2_, 16 h, rt, 96% of 34. Ac = acetyl, Bn = benzyl; DCC = *N*,*N*′-dicyclohexylcarbodiimide; 4-DMAP = 4-dimethylaminopyridine; DMF = dimethylformamide; *m*CPBA = *meta*-chloroperoxybenzoic acid; PMB = *para*-methoxybenzyl; PTSA = 4-toluene sulfonic acid; TES = Triethylsilane; Tf = triflyl.

The synthetic strategy for the development of the diazirine-functionalized PI(5)P, 9, was adapted from our recent report of isotope enriched PI4P and PI(5)P derivatives.^41^ Briefly, using standard conditions, *myo*-inositol 10 was protected to form the orthoformate ester 11 (Scheme 1).^41,42^ The axial hydroxyl groups were then alkylated with PMBCl and sodium hydride to afford orthoformate 12 in a 30% yield. The orthoformate was removed under acidic conditions to form inositol 13 in an 80% yield. The 1-position of inositol 13 was then selectively acetylated using an enzyme from *Thermomyces lanuginosus*, Lipozyme TL-IM® as first reported by Simas *et al.*,^43^ to form a single enantiomer of compound 14 (>99% *e.e.*, >99% yield). The 2- and 3-hydroxyl positions were simultaneous protected as the cyclopentylidene acetal, using dimethoxycyclopentane 15 and PTSA·H_2_O, giving 16 in a 76% yield. The remaining free hydroxyl was then phosphitylated using *o*-xylylene *N*,*N*-diethylphosphoramidite and 1*H*-tetrazole, and the phosphite intermediate was oxidized using *m*CPBA, to give the acetylated-inositol phosphate ester core 18, in a 78% yield. Compound 18 was deprotected with potassium carbonate to give the free alcohol 19, ready for phosphorylation, in an 82% yield.

Following the synthesis of the inositol core 19, the diazirine- and alkyne-containing palmitic acid derivative 27 was synthesized starting from 9-bromo-1-nonanol 20 (Scheme 1). 9-Bromo-1-nonanol 20 was first protected, using DHP and pyridinium 4-toluene sulfonate, to give the THP ether 21 in a 99% yield. The bromide was then substituted with lithium acetylide to give the alkyne 22 in a 65% yield. The alkynylated THP ether 22 was deprotected under acidic conditions to furnish the free alcohol 23 (96%), before it was oxidized to tridec-12-ynal 24 using standard Swern conditions (85%). The aldehyde 24 was reacted in a Stetter reaction with ethyl acrylate and a thiazolium salt catalyst, to form the required palmitic ethyl ester ketone 25 (41%), which was subsequently hydrolyzed to afford the free acid 26 using LiOH (66%). Ketone 26 was then converted to the diazirine using ammonia, hydroxylamine-*O*-sulfonic acid, and iodine, giving the desired diazirine and alkyne derivatized palmitic acid 27 in a 52% yield.

To attach 27 to the remainder of the PI(5)P scaffold, phosphoramidite 34 was required. To prevent racemization at the glycerol *sn*-2 position, two protecting group strategies were investigated, however, here we detail the more robust strategy that resulted in no racemization (Scheme 1). Details of the alternative strategy can be found in the SI (SI, Scheme S2). Both routes started by reacting (*R*)-(−)-2,3-isopropylidene-*sn*-glycerol with palmitic acid and DCC to form the palmitate 29 in a 77% yield. The method used for the opening of the acetonide, which was first reported by Rychnovsky *et al.* in the total synthesis of (–)-Roxaticin and then more recently used by Painter *et al.* in the synthesis of a stearoyl-based glyceride, minimizes the risk of racemization.^44,45^ Briefly, acetonide 29 was treated with TESOTf, followed by sodium bicarbonate and iodine, which resulted in the TES-protected glyceride 30 (18% yield). The free alcohol was then esterified with the previously synthesized analogue of palmitic acid, 27, to give the TES-protected diglyceride 31 in a yield of 85%. The TES group was removed using FeCl_3_, giving the desired diglyceride 32 in a 93% yield (>99% *e.e.*). *e.e.* was determined using ^1^H NMR analysis, by derivatizing the obtained alcohol with either (+)- or (–)-methoxyphenylacetic acid. Details are provided in the SI (Fig. S3). The enantiomerically pure glyceride 32 was then reacted with phosphoramidite 33 and 1*H*-tetrazole, to furnish the diglyceral phosphoramidite 34 (96%), ready to be coupled with inositide 19.

**Scheme 2:**
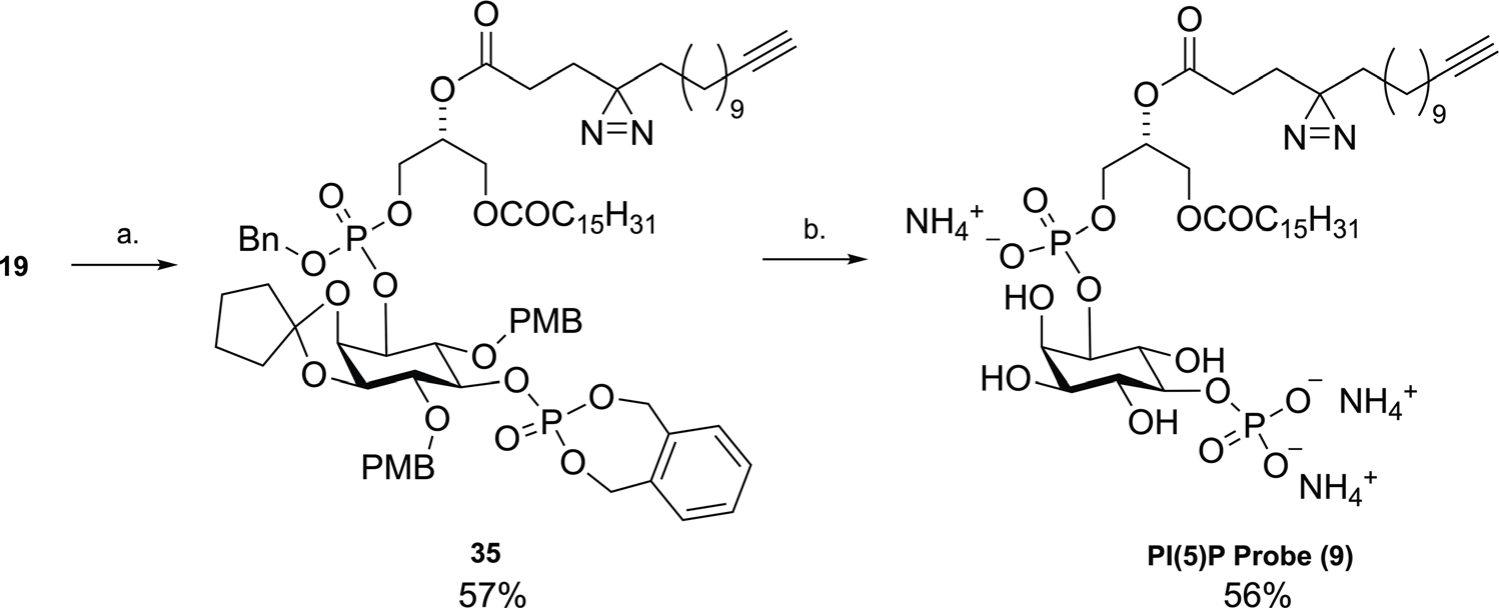
The synthesis of PI(5)P-DIAZ (9). Reagents and conditions: (a). (i) Phosphoramidite 34, 1H-tetrazole, CH_2_Cl_2_ (ii) *m*CPBA, 57% of 35; (b). (i) TMSBr, toluene, rt, 2 h (ii) MeOH, 0 °C, 1 h, 56% of PI(5)P-Probe 9. Bn = benzyl; PMB = *para*-methoxybenzyl; TMS = trimethylsilane.

Inositide 19 was reacted with diglyceral phosphoramidite 34 and 1*H*-tetrazole, followed by *m*CPBA oxidation, to give the protected functionalized PI(5)P 35 in a 57% yield (Scheme 2). The protecting groups were removed by treatment with TMSBr and methanol, furnishing the desired functionalized PI(5)P diazirine 9 in a yield of 56%, which was purified by silica gel column chromatography, eluting with CHCl_3_ : MeOH : 2.2 M NH_4_OH, (9 : 7 : 2) giving the ammonium salt. Due to the instability of the deprotected substrate, and the fact that upon deprotection, all chromophores are removed, the purity of the penultimate compound, 35, was ensured to be >99% by HPLC before deprotection. The purity of the final compound was necessarily assessed using ^1^H NMR analysis.

**Fig. 4:**
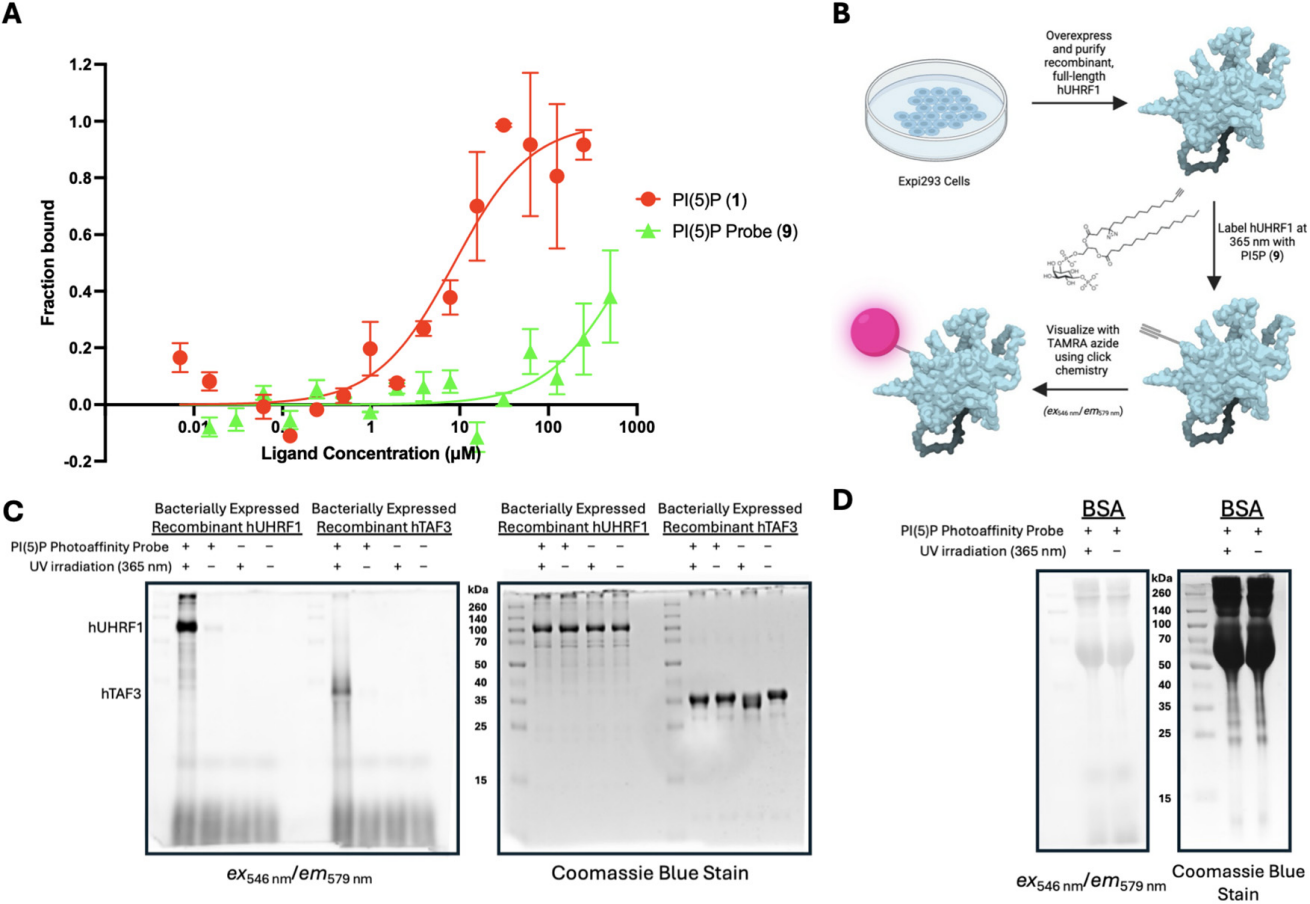
(A) The binding of PI(5)P (1) and PI(5)P-diazirine (9) to bacterially expressed recombinant hUHRF1 (200 nM) was analyzed using MST. Fraction bound was plotted as an average of three independent measurements. Error bars correspond to the standard deviation. The *K*_d_ value of the PI(5)P-probe (9) could not be determined due to the titration curve not reaching saturation, however, a binding interaction was observed at higher concentrations; (B) A cartoon depiction of the workflow used to visualize PI(5)P (9) labelled hUHRF1, and other PI(5)P binding partners; (C) demonstrates the photo cross-linking of PI(5)P-diazirine (9) to bacterially expressed, purified recombinant hUHRF1 and hTAF3; D demonstrates the specificity of the photo cross-linking of PI(5)P-diazirine (9) to its binding partners, as it shows little cross-linking to Bovine Serum Albumin (BSA). For (C) and (D), each SDS-PAGE gel was analyzed for fluorescence emitted by a TAMRA-azide dye, which was conjugated to the alkyne functionality of probe 9*via* ‘click’ chemistry, using the appropriate filters for excitation at 546 nm and emission at 579 nm (left panel), total protein in each lane was observed *via* Coomassie staining (right panel). UV irradiation refers to the lysate/sample being exposed to 365 nm UV light for 30 min, which is necessary for photo-crosslinking of the PI(5)P probe (9) to bound proteins.

With compound 9 in hand, microscale thermophoresis (MST) was used to determine if the PI(5)P diazirine probe 9 could bind hUHRF1 (Fig. 4A). Binding of the diazirine probe 9 to recombinant, bacterially expressed hUHRF1, (bacterially expressed hUHFR1 does not contain PI(5)P as a competing co-factor)^19^ was observed at concentrations exceeding 50 µM, therefore a *K*_d_ value could not be determined due to limitations in the titration scheme. The *K*_d_ of PI(5)P 1 for hUHRF1 was approximately 8.6 µM by MST. Modest alteration of a single C16 acyl chains, by introduction of a diazirine photo crosslinking group and terminal alkyne, resulted in a *K*_d_ that could not be determined for the PI(5)P diazirine 9, highlighting either the specificity of hUHRF1 for the unaltered di-16C acyl chain, or that introduction of the diazerine and alkyne moieties drastically changes the properties of the PI(5)P molecule.

To further investigate the reason for the substantial decrease in binding affinity, we performed dynamic light scattering (DLS) (Fig. S4). Unmodified PI(5)P exhibited comparable size distributions in both deionized water and MST buffer (containing 0.05% TWEEN), with mean hydrodynamic diameters of 37 ± 1.1 nm in water and 48 ± 3.0 nm in MST buffer. In contrast, the diazirine-modified PI(5)P formed considerably larger assemblies overall, measuring 270 ± 18 nm in water and 153 ± 35 nm in MST buffer, indicating that the chemical modifications lead to a substantial increase in particle size in comparison to unmodified PI(5)P. Yet, the presence of detergent at the concentration used, did not significantly alter the assembly state of the lipids. In general, detergents can promote the formation of lipid–detergent micelles or mixed aggregates, and the coexistence of such species with free lipid molecules can create complex equilibria that influence lipid–protein interactions.^46,47^ However, under our experimental conditions, the comparable size distributions observed in the presence and absence of detergent suggest that PI(5)P and diazirine-modified PI(5)P remain predominantly in the same assembly state. The findings enable interpretation of the MST binding behavior of both PI(5)P and its diazirine-modified derivative 9 with hUHRF1. The weaker binding of the diazirine-modified derivative to hUHRF1 compared to PI(5)P, could either be a direct consequence of the chemical modifications perturbing the molecular recognition interface, or, indirectly, be due to the functionalized molecule biophysically behaving differently. The fact that the assemblies observed for the diazirine-modified derivative of PI(5)P are considerably larger than those formed by PI(5)P support the involvement of indirect effects. Although, in the absence of structural insights, it is impossible to determine the presence or scope of direct effects.

While the interpretation of the MST binding data and the derived *K*_d_ are complex, we note that our previous work has shown that hUHRF1 binds PI(5)P when in the state of a bicelle, albeit weaker when compared to monomeric PI(5)P.^17^ It should also be noted that PI(5)P was initially discovered as a co-factor for hUHRF1 through the use of a liposome floatation assay, further demonstrating the ability of hUHRF1 to interact with PI(5)P in an aggregated form. Based on these considerations, the affinity probe could still be functional despite the weaker affinity and higher state of aggregation.^19^

To test this, compound 9 was combined with either bacterially expressed, purified recombinant hUHRF1, or bacterially expressed, purified recombinant hTAF3, and irradiated with light at a wavelength of 365 nm for 30 minutes to induce photo cross-linking. A copper-catalyzed click reaction was then performed to conjugate a TAMRA-PEG-azide fluorophore to the covalently bound PI(5)P (Fig. 4B). Using SDS-PAGE gel electrophoresis, fluorescent bands were observed at the running positions corresponding to the molecular weights of either hUHRF1 or hTAF3 (Fig. 4C), confirming that the diazirine probe bound to bacterially expressed recombinant hUHRF1 and hTAF3, and could undergo the photo-crosslinking with either protein. Next, to determine if the diazirine probe 9 was selectively binding its targets, and not non-specifically labelling hydrophobic proteins, the diazirine probe 9 was incubated with bovine serum albumin (BSA), and subject to the photo-crosslinking/click chemistry reaction. Following SDS-PAGE analysis, Fig. 4D, shows that there was no substantial labelling of BSA, confirming the selectivity of the diazirine probe 9.

**Fig. 5:**
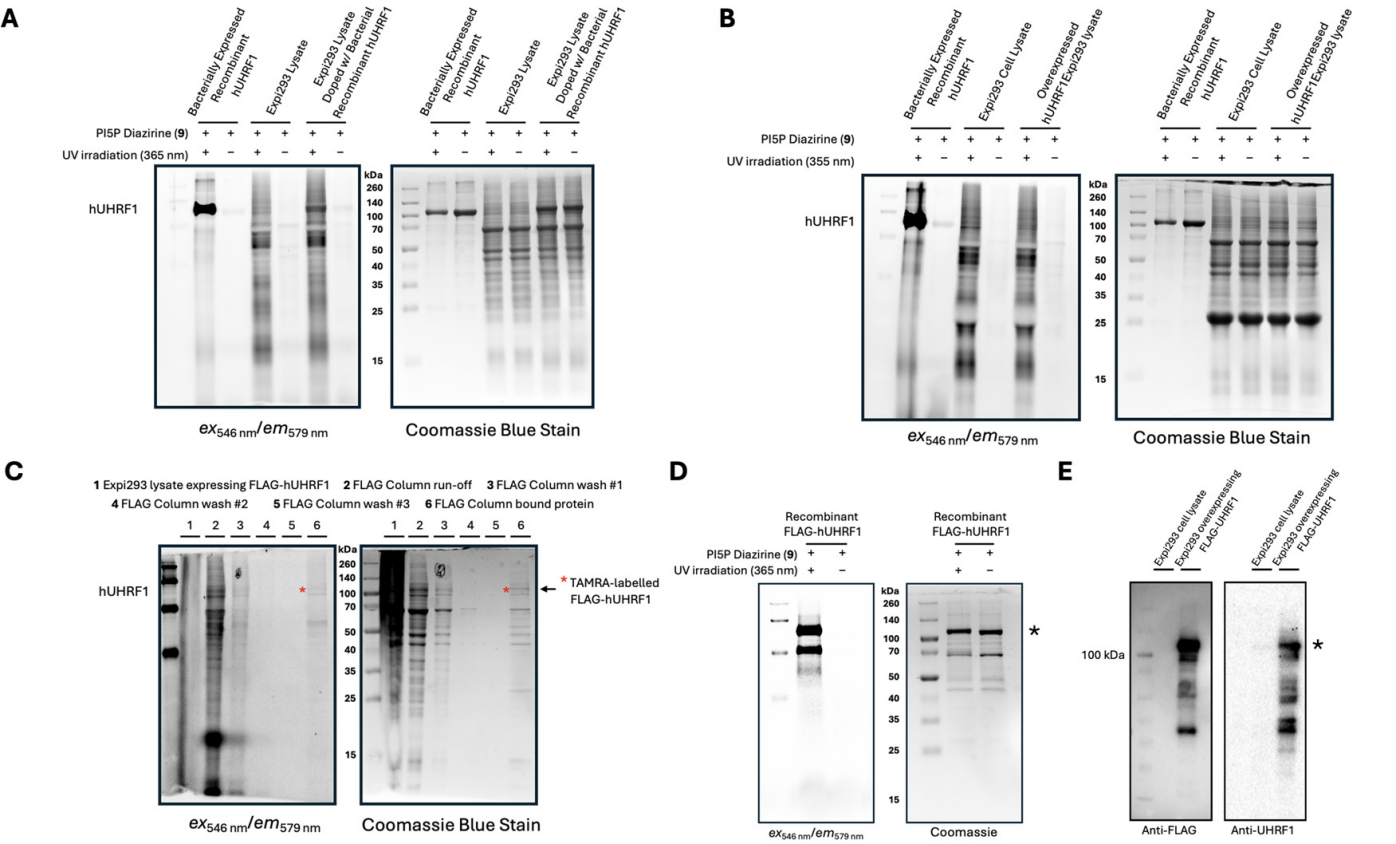
(A) demonstrates; the photo-crosslinking of PI(5)P diazirine (9) to purified bacterially expressed recombinant hUHRF1 (contains no PI(5)P as co-factor); the photo-crosslinking of PI(5)P diazirine (9) to protein binding partners in Expi293 human cell lysate (contains endogenous PI(5)P); the photo-crosslinking of PI(5)P diazirine (9) to bacterially expressed recombinant hUHRF1 doped into Expi293 cell lysate. (B) demonstrates the functionality of the PI(5)P diazirine probe (9) in cell lysate, highlighting many binding partners. Labeling of hUHRF1 expressed in cells (either endogenous levels or after overexpression) was not possible, likely do to the probe (9) not capable of outcompeting endogenous PI(5)P; Panel (C) shows the FLAG-affinity purification of FLAG-tagged hUHRF1, following crosslinking with the probe 9 and click reaction with the azide-dye, demonstrating that PI(5)P diazirine (9) was able to bind and photo-crosslink to FLAG-hUHRF1 in cell lysate (*N.B.* lane 1 contains lysate in which no diazirine probe (9) was added. If added, images became oversaturated which diminished the ability to observe weaker signals as those observed in lane 6). Panel (D) demonstrates the photo-crosslinking of PI(5)P diazirine (9) to purified recombinant FLAG-tagged hUHRF1. Panel (E) shows a western blot of Expi293 cell lysate visualized with both an anti-FLAG and anti-hUHRF1 antibodies. Together panels (D) and (E) confirm the identity of the labelled protein in Panel (C). UV irradiation refers to the lysate/sample being exposed to 365 nm UV light for 30 min, which is necessary for photo-crosslinking of PI(5)P diazirine (9) to bound proteins. Each SDS-PAGE gel was analyzed for the fluorescence of the conjugated TAMRA-azide dye using the appropriate filters for excitation at 546 nm and emission at 579 nm (left panel), total protein in each lane was observed *via* Coomassie staining (right panel). The running positions of molecular weight markers are indicated. In panels (C)–(E), the */arrow indicates the purified, diazirine-labelled protein running at the expected position of FLAG-hUHRF1.

As the functionality of the probe was now validated against purified, bacterially expressed recombinant protein, we next wanted to investigate whether the PI(5)P photoaffinity probe 9 would be functional in complex cell lysate. Following addition of the probe to Expi293 cell lysate, and subsequent crosslinking and click reaction with the TAMRA-azide fluorophore, we observed that the PI(5)P diazirine probe 9 could bind and crosslink to multiple proteins within the complex cell lysate (Fig. 5A). We could not, however, unambiguously establish binding to endogenous hUHRF1, potentially due to multiple factors, including low expression levels of endogenous hUHRF1, or due to competition of endogenous PI(5)P binding hUHRF1 as a co-factor. Nonetheless, artificially increasing the amount of hUHRF1 in the cell lysate by doping in concentrated, bacterially expressed recombinant hUHRF1, confirmed the probe (9) could bind and photo-crosslink to the bacterially expressed hUHRF1 in the experimental condition of cell lysate (Fig. 5A and Fig. S3). Based on these considerations, we engineered Expi293 cells to overexpress hUHRF1 increasing its level in the cell lysates (Fig. 5B, Coomassie). However, we could still not observe labeling of the protein with the TAMRA dye after adding the PI(5)P photoaffinity probe 9, following UV irradiation and subsequent click reaction. We interpret these results that the probe 9 was not cross-linking sufficiently to hUHRF1 to subsequently allow for visualization most likely due to the probe not being able to outcompete endogenous PI(5)P.

To observe even low levels of cross-linked protein, we overexpressed FLAG-tagged hUHRF1 in Expi293 cells, with the FLAG-tag allowing for affinity purification and concentration of the hUHRF1 protein. Following incubation with probe 9, cross-linking and click reaction with the TAMRA dye, and then purification with a FLAG-affinity column, we observed a band under UV excitation after SDS-PAGE running at the expected molecular weight of FLAG-tagged hUHRF1 (Fig. 5C). To confirm the identity of the band, recombinant FLAG-hUHRF1 was purified, and then visualized following crosslinking/click with probe 9 (Fig. 5D). In addition, western blot analysis of Expi293 cell lysate expressing FLAG-hUHRF1, using both anti-FLAG and anti-hUHRF1 antibodies (Fig. 5E), confirmed the presence of FLAG-hUHRF1. Together these data confirm the identity of the band and support the utility of the diazirine probe (9) as a tool for finding and visualizing PI(5)P binding partners.

## Conclusion

In conclusion, we have synthesized and validated the first photoaffinity probe based on PI(5)P (9). The probe was furnished using a concise synthesis that involved a key enzymatic desymmetrization step. The ability of the probe to bind and under-go photo-crosslinking to hUHRF1 and other known PI(5)P binders was confirmed using gel electrophoresis. The covalently cross-linked probe was subject to a ‘copper click’ CuAAC reaction with a TAMRA dye, demonstrating that the probe can be functionalized to visualize bound proteins. In addition, the probe bound to and cross-linked to a variety of proteins within complex cell lysate, while also not binding to BSA, indicating selectivity interactions with these proteins. Together, these results support the potential for this PI(5)P photo-affinity probe 9 to be a useful tool for exploring the biological functions of PI(5)P.

## Conflicts of interest

There are no conflicts to declare.

## Supplementary Material

CB-OLF-D6CB00148C-s001

## Data Availability

The data supporting the findings of this study are available within the article and its supplementary information (SI). Supplementary information: experimental procedures, and compound characterization data. See DOI: https://doi.org/10.1039/d6cb00148c. Additional data supporting the conclusions of this work are available from the corresponding authors upon reasonable request.
