# Supplementary material for: Synthesis and characterization of a novel phosphatidylinositol 5-phosphate (PI(5)P) photoaffinity probe
Source: RSC Chem Biol. 2026 Jun 1;7(7):1372–81. doi: 10.1039/d6cb00148c (PMC13224050; doi:10.1039/d6cb00148c)
Supplement: CB-007-D6CB00148C-s001 [file CB-007-D6CB00148C-s001.pdf]

Supplementary Information (SI) for RSC Chemical Biology.
This journal is © The Royal Society of Chemistry 2026

Synthesis and characterization of a novel phosphatidylinositol 5-phosphate (PI(5)P) photoaffinity probe

Supplementary Information

Glen Brodie,[1,2] Ahmed Sayed,[3,4] Sarah Kreuz, [3] Wolfgang Fischle,[3] Stuart J. Conway.[1,2,]*

Author affiliations.

[1]Department of Chemistry, Chemistry Research Laboratory, University of Oxford, Mansfield Road, Oxford OX1 3TA, U.K.

[2]Department of Chemistry & Biochemistry, University of California Los Angeles, 607 Charles E. Young Drive East, Los Angeles, California 90095, U.S.A.

[3]Bioscience Program, Biological and Environmental Science and Engineering Division, King Abdullah University of Science and Technology (KAUST), Thuwal 23955, Kingdom of Saudi Arabia

[4]Chemistry Department, Faculty of Science, Assiut University, Assiut 71516, Egypt

To whom correspondence should be addressed: stuartconway@ucla.edu

TABLE OF CONTENTS

SUPPLEMENTARY FIGURES AND SCHEMES 3

BIOLOGY AND BIOLOGICAL METHODS 8

SYNTHESIS AND CHARACTERIZATION 13

NMR SPECTRA AND OTHER DATA 48

REFERENCES 155

Supplementary Figures and Schemes

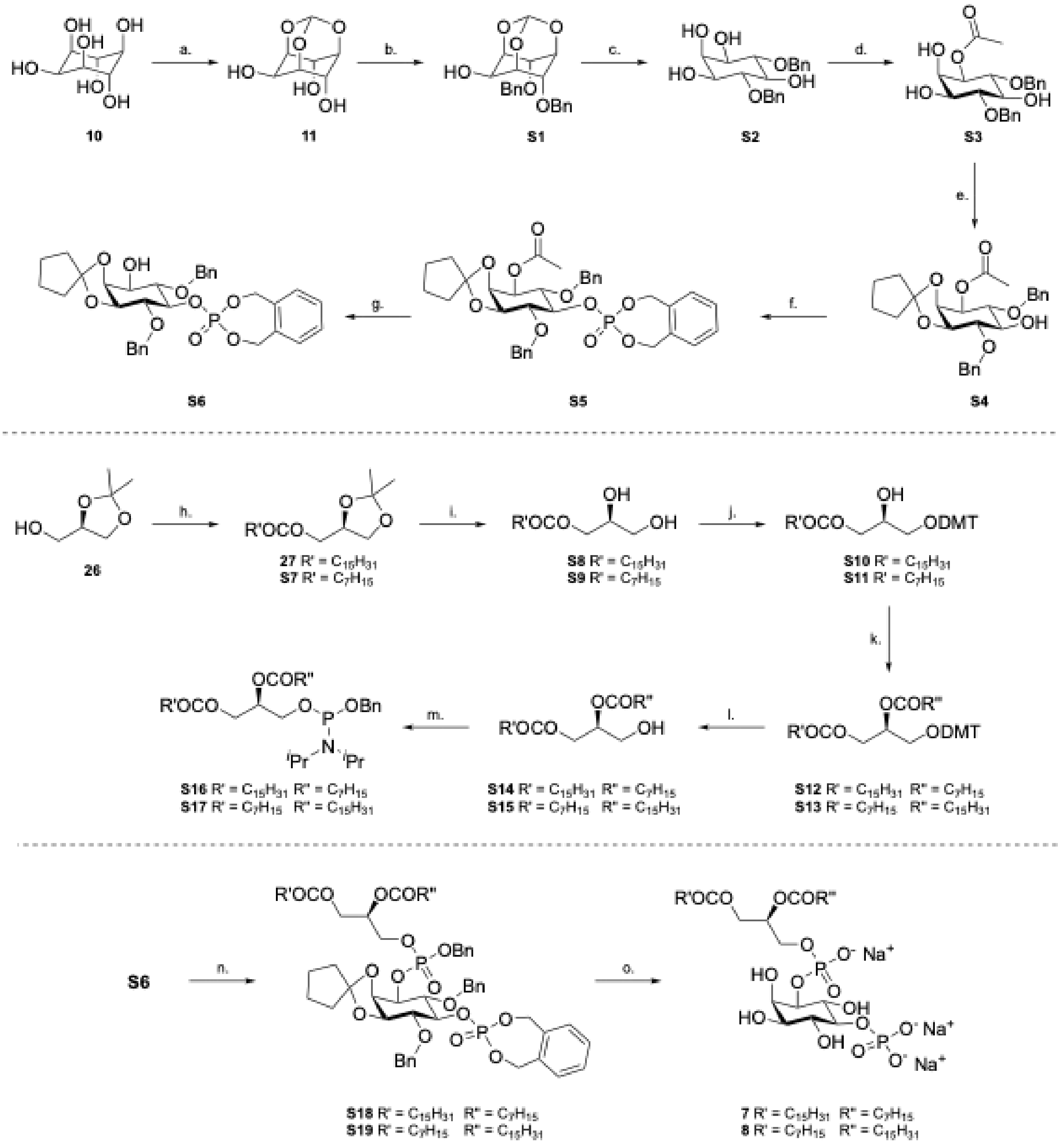

Scheme S1: Synthesis of PI(5)P analogues **7** and **8**. *Reagents and conditions:* a. Triethylorthoformate, PTSA, DMF, 110 °C, 84% **9**; b. i) LiH, DMF, RT, 30 min; ii) BnBr, RT, 72 h, 63% of **S1**; c. PTSA·H_2O, MeOH, RT, 50 min, 97% of **S2**; d. Lipozyme TL-IM®, vinyl acetate:hexane (1:1), 45 °C, 15 h, 95%, >99% e.e. of **(−)-S3**; e. 1,1-Dimethoxycyclopentane**,** PTSA·H_2O, CH_2Cl_2, RT, 18 h, 96% of **(−)-S4**; f. i) (1,5-dihydro-2,4,3-benzodioxaphosphepin-3-yl)diethylamine, 1*H*-tetrazole, CH_2Cl_2, RT, 1 h; ii) *m*CPBA, −78 °C to RT, 1.5 h, 93% of **(−)-S5**; g. K_2CO_3, MeOH, RT, 1 h, 72% of (+)-**S6**; h. Palmitic acid or octanoic acid, DCC, 4-DMAP, CH_2Cl_2, 0 °C to RT, 13 h, 77 to 91% of **(−)-27** or **(−)-S7**; i. AcOH:H_2O (4:1), 50 °C, 2 h, 91–99% of **S8** or **S9**; j. DMTCl, pyridine, RT, 50 min, 78–79% of **(+)-S10** or **(+)-S11** k. Octanoic acid or palmitic acid, DCC, 4-DMAP, CH_2Cl_2, RT, 24 h, 69–84% of **(+)-S12** or **(+)-S13**; l. AcOH acid:H_2O (4:1), RT, 2 h, 81%, >94% *e.e.** of **(−)-S14** or 81%, >90% *e.e.** of **(−)-S15**; m. **31**, 1*H*-tetrazole, CH_2Cl_2, RT, 19 h, 92–>99% of **(+)-S16** or **(+)-S17;** n. i) **(+)-S16** or **(+)-S17**, 1*H*-tetrazole, CH_2Cl_2, RT, 20–22 h; ii) *m*CPBA, −78 °C to RT, 3–4 h, 81−88% of **(+)-S18** or **(+)-S19**; o. i) Pd black, $H_{2(g)}$, ^{t}BuOH/H_2O, RT, 18 h; ii) $NaHCO_3$, RT, 2 h, 60−75% of **(+)-7** or **(+)-8**. Ac = acetyl, Bn = benzyl; DCC = *N*,*N′*-dicyclohexylcarbodiimide; 4-DMAP = 4-dimethylaminopyridine; DMF = dimethylformamide; DMT = dimethoxytrityl; *m*CPBA = *meta*-chloroperoxybenzoic acid; PTSA = 4-toluene sulfonic acid. **e.e* was determined using NMR (see Figure S1).

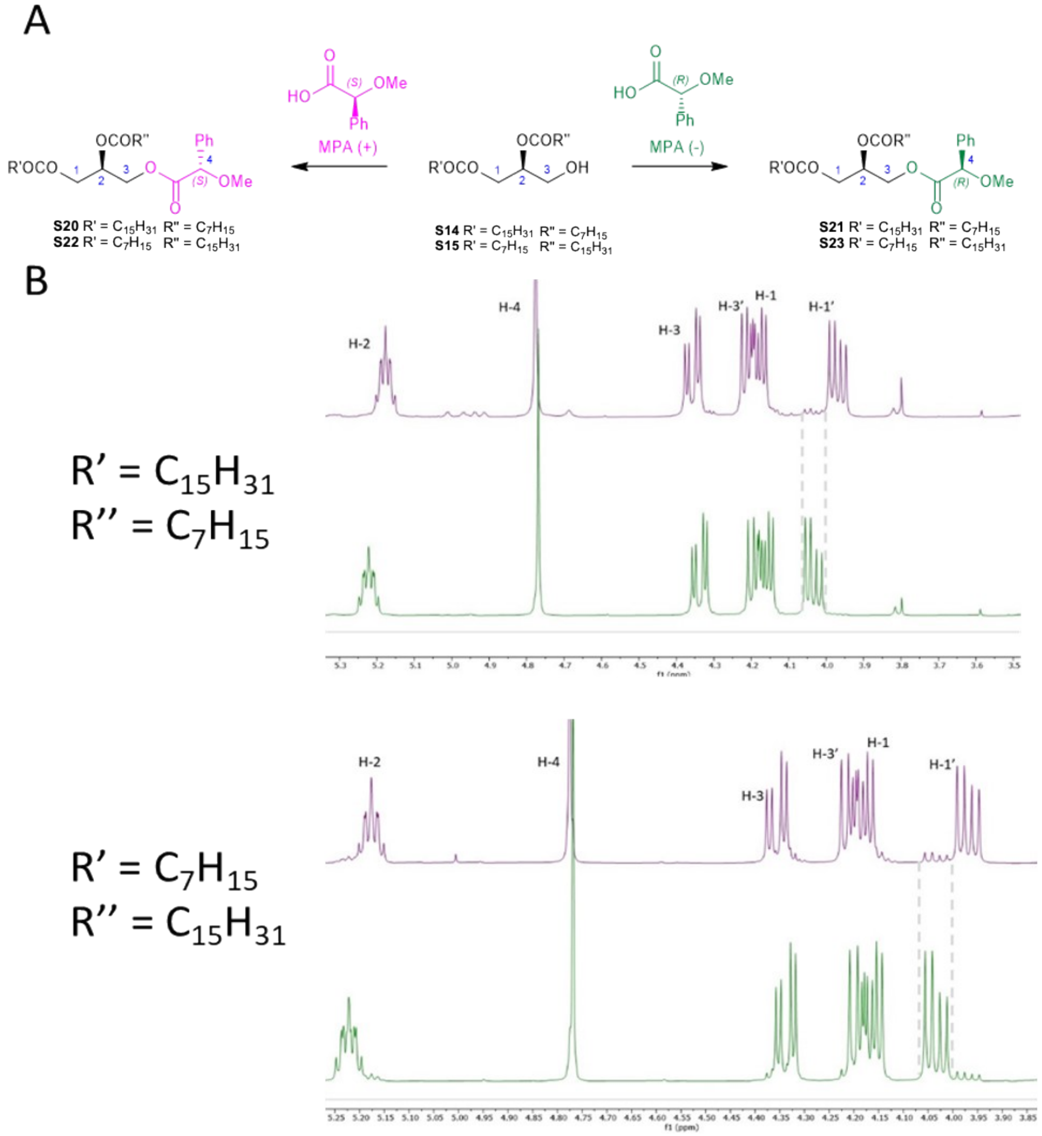

Figure S1: Determination of the *e.e.* values of diglycerides **S14** and **S15**. **A** Derivatization of diglycerides. *Reagents and conditions:* i. (+)-MPA or (–)-MPA, DCC, 4-DMAP, CH_2Cl_2, RT, 6–18 h, 75–83% of **S20, S21**, **S22**, or **S23**. **B** ^1H NMR spectra of diastereoisomers revealing the *e.e.* of **S14** to be ~94% and **S15** to be ~90% using a method described by Seco *et al.*[1] DCC = *N*,*N*′-dicyclohexylcarbodiimide; 4-DMAP = 4-dimethylaminopyridine; MPA = methoxyphenylacetic acid.

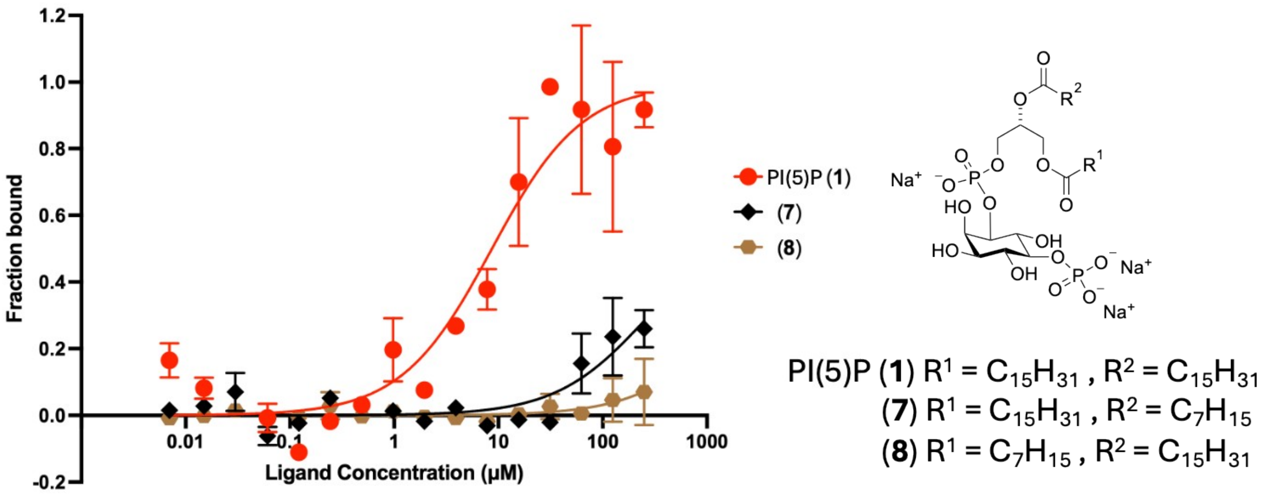

Figure S2: The binding of PI(5)P (**1**) and PI(5)P-analogues (**7**) and (**8**) to recombinant hUHRF1 (200 nM) was analyzed using MST. Fraction bound was plotted as an average of three independent measurements. Error bars correspond to the Std Dev. The K_d values of (**7**) and (**8**) could not be determined due to the titration curve not reaching saturation, however, a binding interaction was observed at higher concentrations.

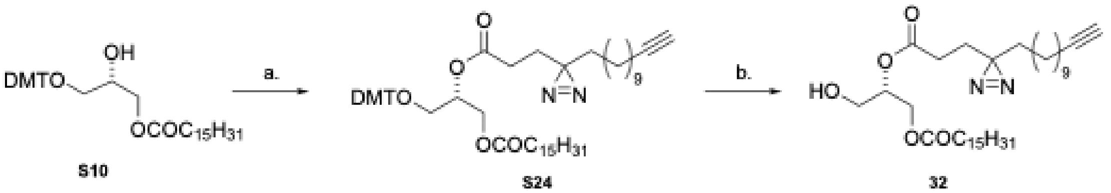

Scheme S2: Alternative reaction conditions for synthesis of alcohol **32**. *Reagents and conditions:* a. Diazirine **27**, DCC, 4-DMAP, CH_2Cl_2, RT, 18 h, 41% of **(+)-S24**; b. AcOH acid:H_2O (4:1), 50 °C, 2.5 h, 68% of **(−)-32**. Ac = acetyl; DCC = *N*,*N′*-dicyclohexylcarbodiimide; 4-DMAP = 4-dimethylaminopyridine.

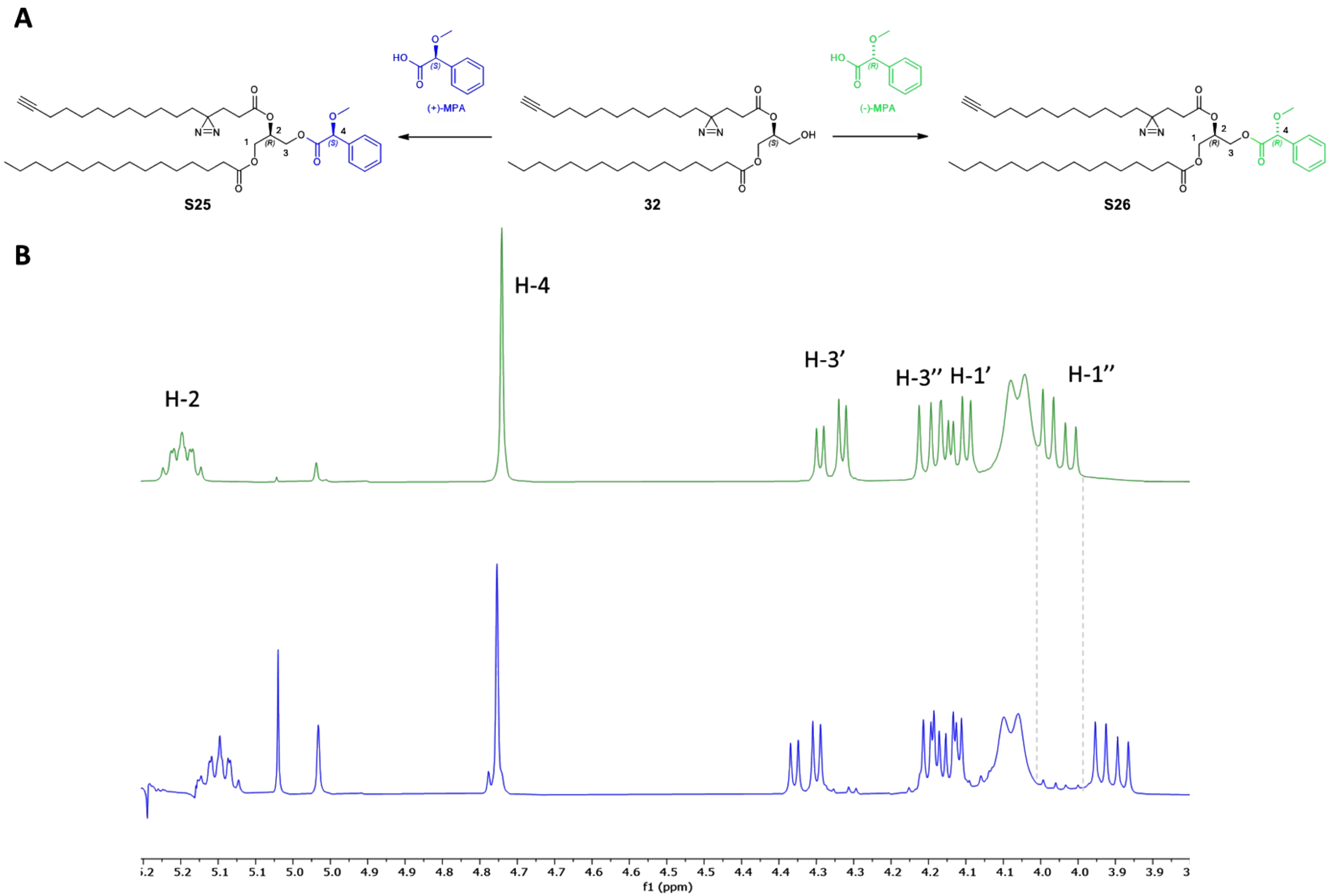

Figure S3: Determination of the *e.e.* value of diglyceride **32**. (A) Derivatization of diglyceride **32** with MPA. *Reagents and conditions:* (+)-MPA or (–)-MPA, DCC, 4-DMAP, CH_2Cl_2, RT, 6–18 h, 65–82% of **S25** or **S26**. (B) ^{1}H NMR spectra of each diastereoisomer revealing the *e.e.* of **32** to be >95% using a method described by Seco *et al.*[1]. DCC = *N*,*N'*-dicyclohexylcarbodiimide; 4-DMAP = 4-dimethylaminopyridine; MPA = methoxyphenylacetic acid

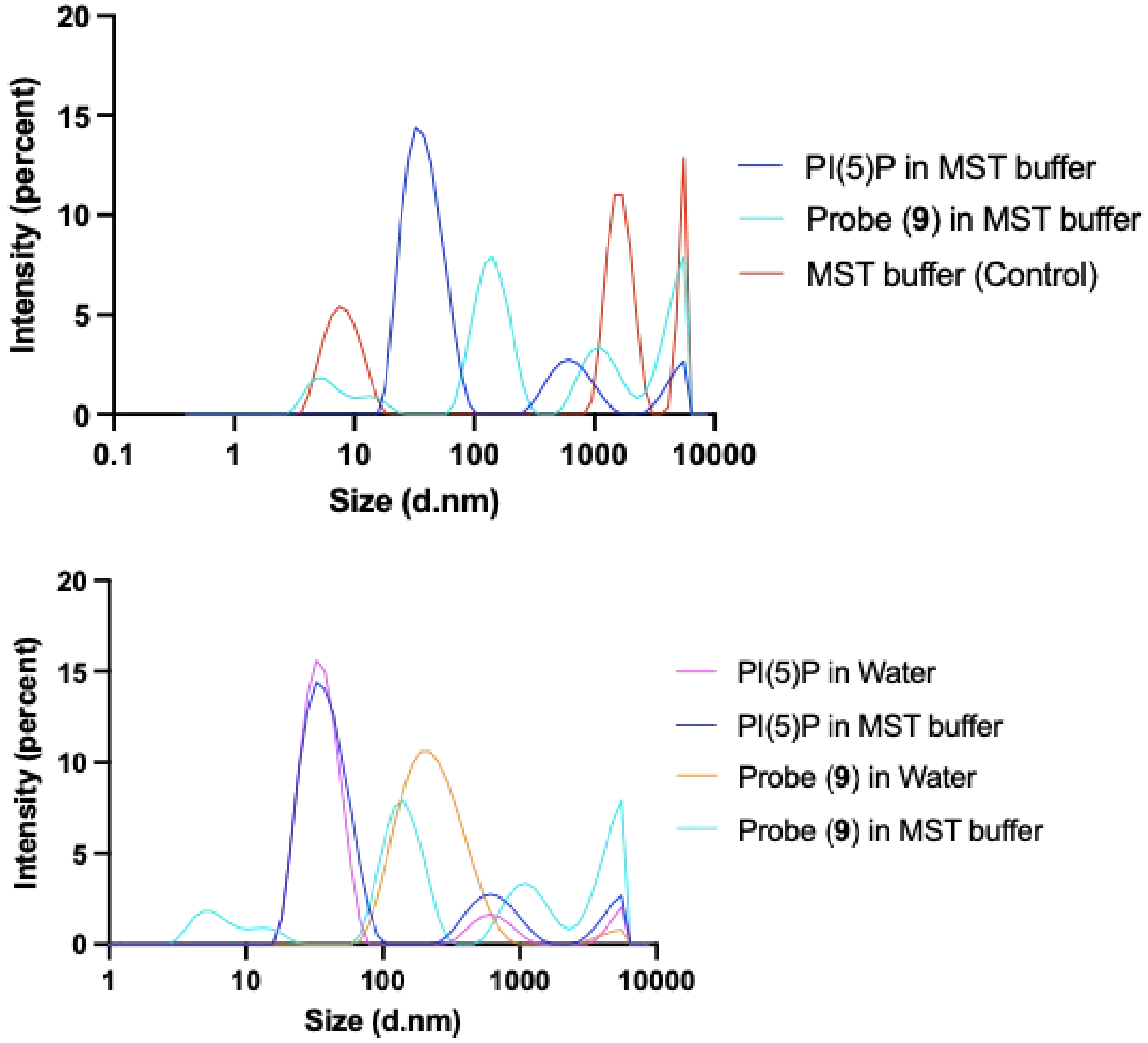

Figure S4: Dynamic light scattering (DLS) data on the distribution of the relative intensity of scattered light by particle size (Stokes diameter) for PI(5)P and probe (**9**) at a concentration of 50 µM in either deionized water or in MST buffer containing 0.05% Tween-20 at 25 °C. MST buffer alone was measured as a control. Unmodified PI(5)P exhibited comparable size distributions under both buffer conditions, with mean hydrodynamic diameters of 37 ± 1.1 nm in water and 48 ± 3.0 nm in MST buffer. In contrast, the diazirine-modified PI(5)P probe (**9**) formed considerably larger assemblies overall, measuring 270 ± 18 nm in water and 153 ± 35 nm in MST buffer.

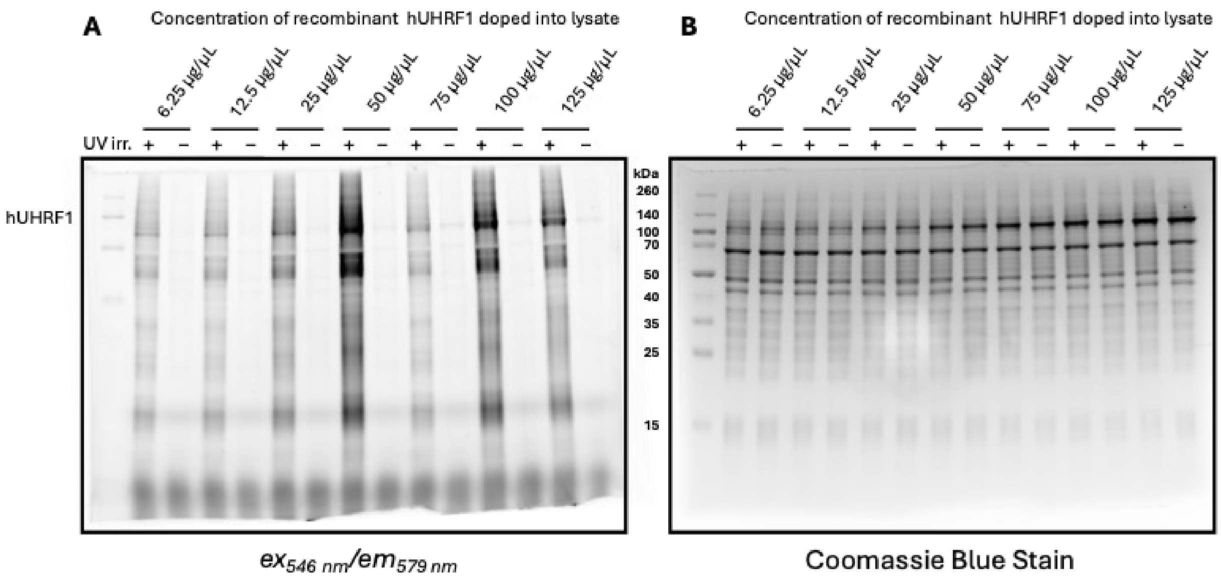

Figure S5: SDS-PAGE analysis of total cells lysate prepared from Expi293 cells containing the indicated amounts of doped bacterially expressed recombinant hUHRF1-FLAG after UV-crosslinking of PI(5)P-diazirine (**9**) and labelling with TAMRA-PEG-azide using click chemistry. The gel was analyzed for fluorescence of the TAMRA dye using excitation with light at 546 nm wavelength (**A**) and following Coomassie staining (**B**). UV irr. refers to the lysate/sample being exposed to 365 nm UV light for 30 min, which is necessary for photo-crosslinking of PI(5)P-diazirine (**9**) to bound proteins. The molecular weight markers are indicated. The gel shows that with increased doped hUHRF1, an increase in signal is detected under UV, confirming that the photo-crosslinking and click reaction with diazirine probe (**9**) to hUHRF1 is detected.

Molecular Biology and Biochemistry

Expression and purification of recombinant proteins

C-terminally His6-tagged hUHRF1 was expressed from a pETM-13 plasmid in BL21-DE3 RIL *E. coli* cells cultured in 2xYT medium supplemented with kanamycin (50 µg/mL) and chloramphenicol (34 µg/mL). The culture was grown at 30 °C with shaking at 180 rpm until the culture reached an OD_{600} of 0.5–0.6, after which, protein expression was induced by the addition of IPTG (0.75 mM aqueous), followed by incubation for 3 hours at 25 °C while shaking. *C*-terminal His6-tagged hTAF3 was expressed from a pET-29b(+) plasmid in a manner similar to hUHRF1 but in the presence of $ZnSO_4$ (50 µM aqueous), with induction at an OD_{600} of 0.6 using IPTG (300 mM aqueous), followed by incubation overnight at 20 °C.

Bacterial cells were harvested by centrifugation at 7,808 × g for 20 minutes at 4 °C, resuspended in Ni-NTA binding buffer (50 mM Tris-HCl, pH 8.0; 300 mM NaCl; 20 mM imidazole) and lysed using an Avestin Emulsiflex-C5 homogenizer at 4 °C. The lysate was cleared by centrifugation at 30,624 x g for 30 minutes at 4 °C. The resulting supernatant containing either hUHRF1 or hTAF3, was incubated with pre-equilibrated Ni-NTA resin in binding buffer for 1 hour at 4 °C. The resin was subsequently washed three times with washing buffer (50 mM Tris-HCl, pH 8.0; 500 mM NaCl; 30 mM imidazole). Proteins were eluted in elution buffer (50 mM Tris-HCl, pH 8.0; 150 mM NaCl; 500 mM imidazole) and dialyzed overnight at 4 °C against storage buffer [50 mM Tris-HCl, pH 8.0; 150 mM NaCl; 10% (*v/v*) glycerol, and 1 mM DTT]. Proteins were concentrated using Amicon Ultra centrifugal filter units (Millipore, Billerica, USA) with a 30 kDa cutoff for hUHRF1 and a 10 kDa cutoff for hTAF3, and further purified by size exclusion chromatography using a Superose^{TM} 6 increase 10/300 GL column.

Mammalian expression of hUHRF1

Expi293F™ cells (Thermo Fisher Scientific, Cat. No. A14527) were cultured in Expi293™ Expression Medium (Thermo Fisher Scientific, Cat. No. A1435101) under controlled conditions at 37 °C with 8% CO_2 in a humidified incubator. hUHRF1-FLAG was expressed from a pcDNA3.1+ plasmid, containing a CMV promoter. Cells were transfected using the polyethylenimine reagent (Polyscience, Cat No. 23966-1), following the manufacturer's protocol. Post-transfection, the cells were cultured for 18 to 72 hours to achieve optimal protein expression. Subsequently, cells were harvested by centrifugation at 300 × g for 10 minutes at 4°C. The resulting cell pellet was resuspended in lysis buffer (50 mM Tris-HCl, pH 8.0, 150 mM NaCl) supplemented with cOmplete™ EDTA-free Protease Inhibitor Cocktail. Cells were lysed by sonication, and the lysate was clarified by centrifugation at 12,000 × g for 15 minutes at 4 °C.

Western blot analysis

Cell lysates (5 mg/ml) were heated for 5 minutes at 95 °C in SDS loading buffer and resolved on 12% acrylamide gels. Proteins were then transferred to nitrocellulose or PVDF membranes, which were subsequently blocked in Tris Buffered Saline with Tween 20 (TBST) containing 5% (w/v) dry milk powder. Anti-hUHRF1 antibodies (sc-373750, Santa Cruz Biotechnology, 1:1000 dilution) and Anti-FLAG antibody (Monoclonal anti-FLAG® BioM2 antibody produced in mouse, Sigma-Aldrich (F1804)) were applied in blocking buffer and incubated overnight. Afterwards, the membranes were washed three times for 10 minutes each with TBST and then incubated with secondary antibodies labeled with HRP in blocking buffer for 1 hour at room temperature. Following another three 10-minute washes with TBST, the membranes were treated with ECL substrate for 2 minutes before visualization.

Microscale thermophoresis

C-terminal His6-tagged hUHRF1 was labelled using the Monolith His-tag labelling kit RED-tris-NTA (Nanotemper #MO-L008) as per the manufacturer's instructions. In summary, MST buffer (20 mM Hepes-NaOH (pH 7.9), 150 mM NaCl, 0.05% (v/v) Tween 20) was prepared. 400 nM of the protein was then incubated with 100 nM of the His-tag labelling dye for 30 minutes at room temperature. Subsequently, the labelled proteins were centrifuged and collected at 15,000 × g for 10 minutes at 4 °C to remove any precipitate. The fluorescently labelled protein was then incubated with a range concentrations of PI(5)P and the diazirine-modified PI(5)P compounds at room temperature for 15 minutes prior to measurement on a Monolith NT.115 instrument (NanoTemper, 80% LED power, 40% MST power).[2] The data points were fitted using MO.Affinity Analysis v2.3 analysis software, and the dissociation constants were then determined using a single-site model to fit the curve.

Particle Size Measurements

Particle size distribution and mean hydrodynamic diameter were determined using a Malvern Zetasizer Nano ZS (Malvern Instruments, St. Laurent, QC, Canada). Measurements were performed in triplicate using various concentrations of unmodified PI(5)P or the diazirine probe **9**. Dynamic light scattering (DLS) measurements were performed in deionized water and in MST buffer containing 0.05% (v/v) Tween-20.

PI(5)P-diazirine probe crosslinking with hUHRF1 and cell lysate and CuAAC Click reaction

PI(5)P-diazirine probe (50 μM) in DMSO (final conc. 0.5%) was incubated with 0.5 mg/mL hUHRF1 and 2 mg/mL Expi293 cell lysate for 30 min at 4 °C (20 μL reaction volume) in reaction buffer (50 mM Tris-HCl pH 8.0, 150 mM NaCl, 10% glycerol, and 1 mM DTT). The solutions were

irradiated with UV light (365 nm) for 15 min at 4 °C using a UVP Blak-Ray™ B-100A UV Lamp. Meanwhile, the negative control samples were incubated in the dark at 4°C.

The components of the click reaction were prepared as follows: Azide Fluor 545 (100 μM final concentration from a 10 mM DMSO stock solution), $CuSO_4$ (1 mM final concentration from a 50 mM aqueous stock solution), TCEP-HCl (1 mM final concentration from a 50 mM aqueous stock solution), and TBTA (100 μM final concentration from a 10 mM DMSO stock solution). Subsequently, the click reaction was carried out by the addition of a click mixture with the following volume ratio 1:2:2:1 of Azide Fluor 545: $CuSO_4$: TCEP: TBTA to the cross-linked samples, and incubated with shaking at 700 rpm for 1 h, in the dark, at room temperature. Then, 4× Laemmli sample buffer premixed with DDT was added to 20 μl of reaction mixture and heated for 5 min at 95 °C. 10 μl was loaded onto 8% SDS-PAGE and gel electrophoresis was performed to separate the proteins. The bottom of the gel was cut to avoid the residual excess of Azide Fluor 545 which could reduce the fluorescence signal of the crosslinked protein. Then, in-gel fluorescence was acquired at 546 nm using a Bio-Rad imager. The gel was further subjected to Coomassie staining followed by a destaining step. The Bio-Rad imager was used to image the Coomassie-stained gel.

For the Biotin-Azide click reaction, the volumes of reagents were scaled up 5-fold while maintaining the same molar ratios as described above. Post-click reaction, 0.5 mL of ice-cold acetone (−20 °C) was added to the mixture. Samples were vortexed and incubated at −20 °C for 30 minutes. The resulting precipitate was collected by centrifugation at 20,000 × g for 10 minutes at 4 °C. The pellet was washed with ice-cold acetone to remove excess Biotin-Azide, followed by sonication in a water bath. Acetone wash and centrifugation steps were repeated twice. The protein pellet was air-dried for 10 minutes at room temperature to remove residual acetone and then resuspended in 50 μL of 1% SDS in PBS, vortexed, and sonicated. The suspension was further diluted with 0.5 mL of affinity purification buffer (50 mM Hepes-NaOH, pH 7.4; 100 mM

NaCl; 1% (v/v) IGEPAL). 50 µL magnetic streptavidin beads (Streptavidin MagneSphere® Paramagnetic Particles, Promega) pre-equilibrated with affinity purification buffer were added to the solution, and the mixture was incubated overnight at 4 °C on a rotating mixer. The beads were recovered using a magnetic rack and washed three times with wash buffer (50 mM Hepes-NaOH, pH 7.4; 500 mM NaCl; 1% (v/v) IGEPAL). Bound proteins were eluted with 50 µL of elution buffer (2 mM D-biotin; 1% (v/v) IGEPAL in PBS) for 30 minutes at room temperature, separated by SDS-PAGE (12% gel), transferred to a PVDF membrane, and detected using streptavidin-HRP (Pierce™ High Sensitivity Streptavidin-HRP, 1:1000).[3]

Anti-FLAG affinity purification

Cell lysate from 293Expi cells overexpressing hUHRF1-FLAG (100 mg total) was incubated with pre-equilibrated Pierce™ Anti-DYKDDDDK Affinity Resin for 2 hours at 4 °C in a buffer containing 50 mM Tris-HCl (pH 8.0), 150 mM NaCl, 1 mM DTT, and 10% (v/v) glycerol. The mixture was gently rotated to facilitate binding of the FLAG-tagged protein to the resin. The resin was then washed three times with the same buffer to remove unbound and non-specifically bound proteins. Elution of bound proteins was performed using 0.5 mg/mL 3×FLAG peptide in binding buffer, with incubation overnight at 4 °C.

Synthesis and Characterization

Chemistry General Methods

^{1}H NMR spectra were recorded on Bruker AVIIIHD 400 nanobay (400 MHz), Bruker NEO 600 (600 MHz) with ^{1}H helium-cooled cryoprobe, or Bruker AVIIIHD 500 (500 MHz) spectrometer in the stated solvents as a reference for the internal deuterium lock. The chemical shift data for each signal are given as δ_H in units of parts per million (ppm) relative to tetramethylsilane (TMS) where δ_H (TMS) = 0.00 ppm. The spectra are calibrated using the solvent peak with the data provided by Fulmer *et al.*[4] The multiplicity of each signal is indicated by s (singlet); br s (broad singlet); d (doublet); dd (doublet of doublets), ddd (doublet of doublet of doublets), t (triplet), q (quartet), dq (double of quartet) or m (multiplet). The number of protons (n) for a given resonance signal is indicated by nH. Where appropriate, coupling constants (*J*) are quoted in Hz and are recorded to the nearest 0.1 Hz. Identical proton coupling constants (*J*) are averaged in each spectrum and reported to the nearest 0.1 Hz. The coupling constants are determined by analysis using Bruker TopSpin version 4.1.3 software. ^{1}H spectra were assigned using 2D NMR experiments including COSY, HMBC, HSQC, HMBC, and ^{1}H-^{31}P HMBC, as required. ^{31}P NMR spectra were recorded on a Bruker AVIIIHD 400 nanobay (162 MHz), or Bruker NEO 600 (243 MHz) spectrometer in the stated solvents as a reference for the internal deuterium lock, using a broadband proton decoupling pulse sequence. The chemical shift for each signal is given as δ_P in units of parts per million (ppm) relative to 85% phosphoric acid as an external reference where δ_P (H_3PO_4) = 0.00 ppm. Signals are singlets unless otherwise stated. ^{31}P spectra were assigned using ^{1}H-^{31}P NMR experiments as necessary. ^{13}C NMR spectra were recorded on a Bruker AVIIIHD 400 nanobay (101 MHz), Bruker NEO 600 (151 MHz) spectrometer in the stated solvents, with broadband proton decoupling and an internal deuterium lock. The chemical shift data for each signal are given as δ_C in units of parts per million (ppm) relative to tetramethylsilane (TMS) where δ_C (TMS) = 0.00 ppm. The spectra are calibrated using the solvent peak with the data provided by Fulmer

et al.[4] The shift values of resonances are quoted to 1 decimal place unless peaks have similar chemical shifts, in which case 2 decimal places are used. Where appropriate, the multiplicity of each signal is indicated by d (doublet), t (triplet) or m (multiplet). Coupling constants (J) are quoted in Hz and are recorded to the nearest 0.1 Hz. These were determined using Bruker TopSpin version 4.1.

When two diastereoisomers or regioisomers are present in the sample, A and B denote each of the two diastereoisomers without distinguishing between them. A is arbitrarily assigned to the diastereoisomer with the highest ppm shift and B to the diastereoisomer with the lowest ppm shift, in ^{1}H NMR, ^{13}C NMR and ^{31}P NMR spectra.

Low resolution electrospray ionization spectra were acquired on a Waters LCT Premier spectrometer or Agilent 6120 Quadrupole spectrometer. High resolution mass spectra were recorded on either a Bruker MicroTOF spectrometer, operating in positive or negative mode, or Waters Micromass LCT from solutions of MeOH, MeCN or H_2O. *m/z* values are reported in Daltons and followed by their percentage abundance in parentheses.

Specific optical rotations were measured using a Schmidt + Haensch UniPol L2000 polarimeter, in cells with a path length of 1 dm, using a sodium lamp at 589 nm. The concentration (c) is expressed in g/100 mL (equivalent to g/0.1 dm^3) Specific rotations are denoted $[\alpha]_D^T$ and are given in implied units of 10^{-1}degcm^{2}g^{-1} at the temperature stated.

Melting points were determined using a Gallenkamp MF370 and are uncorrected. The solvents of crystallization are shown in parentheses. Infrared (IR) spectra were obtained from neat samples, either as liquids or solids using a diamond ATR module. The spectra were recorded on a Bruker Tensor 27 spectrometer. Absorption maxima are reported in wavenumbers (cm^{-1}).

Thin layer chromatography (TLC) was carried out on normal phase Merck silica gel 60 F254 aluminum-supported chromatography sheets. Visualization was by absorption of UV light (λ_{max} 254 nm) or by development from an aqueous solution of potassium permanganate. Reaction progress was monitored at appropriate times either by TLC or by ^{31}P NMR. Normal phase silica gel flash column chromatography was performed either manually using VWR Prolabo silica gel 60 (240–400 mesh) under a positive pressure of nitrogen or using a biotage Selekt System with pre-packaged normal phase Biotage Sfär columns.

Chemicals were purchased from Apollo Scientific, Merck UK, Alfa Aesar UK, Fisher Scientific UK, and Fluorochem. All reagents were purified, when necessary, by standard techniques. Et_3N, DIPEA, DIPA were dried by stirring over CaH_2 followed by distillation. These were stored under Ar and over 3 Å molecular sieves. PCl_3 was heated under reflux to expel dissolved HCl, then distilled and stored under Ar. Anhydrous solvents were obtained under the following conditions: THF, MeCN, DMF, Et_2O, toluene, MTBE and CH_2Cl_2 were dried by passing through a column of activated basic alumina, and then then further dried over 3 Å molecular sieves. Anhydrous MeOH was purchased from Sigma Aldrich UK in SureSeal™ bottles and used without further purification. All other solvents were used as supplied (analytical or HPLC grade) without purification.

Where appropriate and if not otherwise stated, all non-aqueous reactions were performed in a flame dried flask under an inert atmosphere. Hexane refers to a mixture of hexane isomers and petroleum ether refers to the fraction of light petroleum ether boiling within the range of 40–60 °C. Brine refers to a saturated aqueous solution of sodium chloride. *In vacuo* refers to the removal of solvents under reduced pressure using a Büchi™ rotary evaporator in a water bath at 40 °C, unless otherwise stated. Vacuum transfer refers to the removal of solvents on a manifold linked to a high vacuum pump at RT. Lyophilization refers to the removal of H_2O and MeCN from aqueous solutions by freeze drying using a CHRIST Alpha 2-4 LSC basic lyophilizer. Celite® refers to Celite® 545 filter aid, treated with sodium carbonate, flux-calcined 23 which was

purchased from Merck. Glass microfiber filter refers to Whatman® borosilicate glass microfiber filters, Grade GF/B.

Compound purity was determined by analytical high-performance liquid chromatography (HPLC) on a PerkinElmer Flexar system with a Binary LC Pump and UV/VIS LC Detector using a normal phase HyperSil GOLD™ Silica column (5 µm, 4.6 × 150 mm) with heptane (A) and IPA (B) as eluents. Gradient methods of 19 minutes were employed with a constant flow rate, and detection at 254 or 220 nm where an isocratic method was employed. Samples were injected by dissolving in the relevant solvent system. The method was A = Heptane; B = IPA; 2.0 $mL.min^{-1}$; 245, 220, or 280 nm, 90% A, 10% B, isocratic 18 min, with 1 min pre-equilibration before injection. Absolute configuration of alcohols **32**, **S14** and **S15** were determined using both enantiomers of α-methoxyphenylacetic acid (MPA) as a chiral derivatizing agent *via* esterification as described by Seco *et al.* and Joffrin *et al.*[1,5]

Synthetic Protocols, Characterization data, and Compound Assignments

***myo*-Inositol 1,3,5-orthoformate 11**

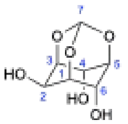

To a solution of *myo*-inositol **10** (5.00 g, 27.8 mmol, 1.0 eq) in DMF (50 mL) were added triethylorthoformate (8.24 g, 55.6 mmol, 2.0 eq) and PTSA·H_2O (211 mg, 1.11 mmol, 0.04 eq). The reaction mixture was stirred at 110 °C for 18 h and then allowed to cool to RT. The solution was neutralized by the addition of solid $NaHCO_3$ (10 g) and filtered to remove excess $NaHCO_3$ and sodium tosylate. The volatile components were removed *in vacuo* and residual DMF was removed using toluene as an azeotrope. The resulting solid was then crystallized from hot MeOH following cooling to −20 °C for 1 h. The resulting colorless crystalline solid was isolated by filtration, washed with $CHCl_3$ (25 mL), and dried to give *myo*-inositol-1,3,5-orthoformate **11** (4.82 g, 84%): R_f 0.40 (MeCN:EtOAc, 8:2); m.p. 255−260 °C (dec.); ^{1}H NMR (400 MHz; D_6-DMSO) δ_H 5.52–5.38 (2H, m, C(4)O*H* and C(6)O*H*), 5.45 (1H, d, *J* 1.4, H-7), 5.30 (1H, br s, C(2)O*H*), 4.28 (2H, dd, *J* 4.0, 4.0, H-1 and H-3), 4.09–4.05 (1H, m, H-5), 4.02–3.99 (1H, m, H-2), 3.97–3.93 (2H, m, H-4 and H-6); LRMS *m/z* (ESI$^+$) 191.0 ([M+H]$^+$ 100%). These data are in good agreement with the literature values.[5]

4,6-Di-*O*-(4-methoxybenzyl)-*myo*-inositol 1,3,5-orthoformate 12

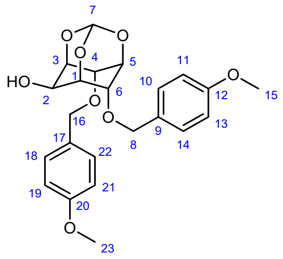

To a solution of orthoformate **11** (5.00 g, 26.3 mmol, 1.0 eq) in anhydrous DMF (200 mL) was added NaH 60% dispersion in mineral oil (2.21 g, 25.2 mmol, 2.1 eq) as a single batch. The solution was stirred for 1.5 h, while being heated to 50 °C. The solution was then cooled to RT and PMBCl (7.13 mL, 52.6 mmol, 2.0 eq) was added dropwise over 10 min. The reaction was stirred for a further 18 h, after which the reaction was quenched with H_2O, and then concentrated *in vacuo*. Residual DMF was removed using toluene as an azeotrope. The residue was then dissolved in MeCN and extracted with petroleum ether to remove any mineral

oil present. The MeCN layer was then concentrated *in vacuo*. The residue was then dissolved in EtOAc (100 mL) and partitioned with H_2O (100 mL). The EtOAc phase was removed, and the remaining aqueous fraction was extracted with EtOAc (3 × 100 mL). The combined organic phases were then washed (brine), dried (Na_2SO_4), filtered, and concentrated *in vacuo*, before being dissolved in the minimum amount of warm EtOAc. The solution was then allowed to cool where orthofomate **12** formed as a colorless crystalline powder (3.39 g, 30%), which was retrieved *via* filtration and required no further purification. The process was repeated twice to recover the maximum amount of material. R_f 0.50 (petroleum ether:EtOAc, 5:5); m.p. 110–112 °C (EtOAc), [lit.[5] 105–107 °C (hexane:EtOAc)]; ^{1}H NMR (400 MHz, $CDCl_3$) δ_H 7.22–7.15 (4 H, m,H-11, H-13, H-19, H-20), 6.85–6.79 (4 H, m, H-10, H-14, H-18, H-22), 5.45 (1 H, d, *J* 1.0, H-7), 4.58 (2 H, d, *J* 11.1, H-8', H-16'), 4.50 (2 H, d, *J* 11.1, H-8'', H-16''), 4.42–4.39 (2 H, m, 2 × inositol C*H*), 4.34 (2 H, t, *J* 3.7, inositol C*H*), 4.21–4.17 (3 H, m, 3 × inositol C*H*), 3.80 (6 H, s, H-15, H-23), 2.99 (1 H, d, *J* 11.5, C(2)O*H*); LRMS (ESI$^+$) 453 ([M+Na]$^+$, 100%). These data are in good agreement with the literature values.[5]

4,6-Di-*O*-(4-methoxybenzyl)-*myo*-inositol 13

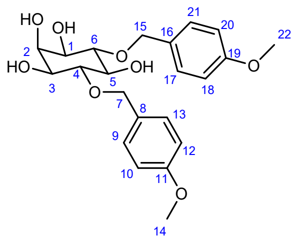

To a solution of the orthoformate **12** (2.00 g, 4.65 mmol, 1.0 eq) in MeOH:H_2O (132 mL, 10:1 *v/v*) was added a few drops of 2 M HCl until a pH of 2–3 was achieved. The reaction was stirred at 45 °C for 1 h after which TLC analysis showed potential minor deprotection of the PMB groups. The reaction was then cooled to 40 °C and allowed to stir for a further 3 h where TLC showed most of the starting material had been consumed. The reaction was then cooled to RT and neutralized with sat. aqueous $NaHCO_3$ until a pH of approximately 7 was reached. The mixture was then concentrated *in vacuo* and the resulting solid was taken up in EtOAc. The remaining solid was removed by filtration and the solution was then passed through a column of

silica gel, eluting with 100% EtOAc to give tetraol **13** (1.56 g, 80%) as a colorless solid. R_f 0.30 (EtOAc); m.p. 134–136 °C (EtOAc), [lit.[5] 144–145 °C (EtOAc)]; ^{1}H NMR (400 MHz, $CDCl_3$) δ_H 7.34–7.28 (4 H, m, H-10, H-12, H-18, H-20), 6.93–6.88 (4 H, m, H-9, H-13, H-17, H-21), 4.83 (2 H, d, *J* 11.1, H-7', H-15'), 4.77 (2 H, d, *J* 11.1, H-7'', H-15''), 4.16–4.12 (1 H, m, inositol C*H*), 3.81 (6 H, s, H-14, H-22), 3.67–3.60 (2 H, m, 2 × inositol C*H*), 3.56–3.47 (3 H, m, 3 × inositol C*H*), 2.55 (1 H, d, *J* 1.3, -OH), 2.46 (1 H, d, *J* 2.1, -OH), 2.42 (2 H, d, *J* 4.9, 2 × -OH). These data are in good agreement with the literature values.[5]

(+)-1D-1-*O*-Acetyl-4,6-di-*O*-(4-methoxybenzyl)-*myo*-inositol 14

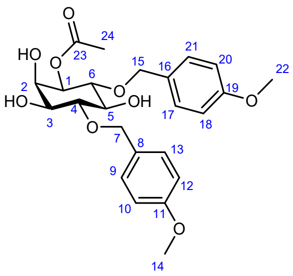

The immobilized Lipozyme® TL-IM (2.00 g) was added to a solution of 4,6-di-*O*-benzyl-*myo*-inositol **13** (1.20 g, 2.85 mmol, 1.0 eq) in vinyl acetate (250 mL) and hexane (250 mL). After stirring at 45 °C for 18 h the reaction mixture was filtered through a pad of Celite®, washed with hexane (3 × 20 mL) and the combined filtrates were concentrated *in vacuo* to give (**−**)-**14** (1.30 g, 99%, >99% *e.e.* other enantiomer not observed) as a colorless amorphous solid, requiring no further purification: R_f 0.61 (EtOAc); $[\alpha]_D^{25}$ +24.4 (*c* 1.0, $CHCl_3$). [lit.[5] $[\alpha]_D^{25}$ +24.0 (*c* 1.0, CH_3Cl)]; ^{1}H NMR (400 MHz, $CDCl_3$) δ_H 7.33–7.28 (2 H, m, H-9 and H-13), 7.25–7.21 (2 H, m, H-17 and H-21), 6.92–6.86 (4 H, m, H-10, H-12, H-18 and H-20), 4.91 (1 H, d, *J* 11.1, H-7'), 4.85 (1 H, dd, *J* 10.1, 2.7, H-1), 4.74 (1 H, d, *J* 11.1, H-15'), 4.69–4.62 (2 H, m, H-7'' and H-15''), 4.20 (1 H, dd, *J* 2.7, 2.7, H-2), 3.89 (1 H, dd, *J* 10.0, 9.9, H-6), 3.80 (6 H, s, H-14 and H-22), 3.71–3.62 (1 H, m, H-4), 3.61–3.53 (2 H, m, H-3 and H-5), 2.52–2.39 (3 H, m, 3 × -OH), 2.12 (3 H, s, H-24). These data are in good agreement with the literature values.[5]

1,1-Dimethoxycyclopentane 15

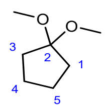

To a solution of cyclopentanone (6.31 mL, 71.3 mmol, 1.0 eq) in hexane (48 mL) were added K-10 montmorillonite clay (10.8 g) and trimethylorthoformate (17.9 mL, 159 mmol, 2.3 eq). The suspension was stirred rapidly for 18 h at RT. The dark brown reaction mixture was then filtered through a pad of Celite® and washed with hexane (2 × 10 mL) and Et_2O (2 × 10 mL). The combined filtrates were concentrated *in vacuo* to give 1,1-dimethoxycyclopentane **15** (7.08 g, 88%) as a colorless oil with sufficient purity for use in the next step: R_f 0.51 (petroleum ether:EtOAc, 8:2); ^{1}H NMR (400 MHz; $CDCl_3$) δ_H 3.21 (6H, s, C(2)OCH_3), 1.80–1.71 (4H, m, H-3 and H-1), 1.69–1.61 (4H, m, H-4 and H-5); These data are in good agreement with the literature values.[5]

(−)-1D -1-*O*-Acetyl-2,3-*O*-cyclopentylidene-4,6-di-*O*-(4-methoxybenzyl)-*myo*-inositol 16

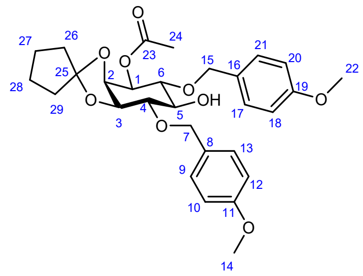

To a solution of (+)-1D-1-*O*-acetyl-4,6-di-*O*-(4-methoxybenzyl)-*myo*-inositol **14** (1.00 g, 2.16 mmol, 1.0 eq) and 1,1-dimethoxycyclopentane **15** (4.22 g, 32.4 mmol, 15 eq) in CH_2Cl_2 (6 mL) was added PTSA·H_2O (31 mg, 0.17 mmol, 0.08 eq). The resulting solution was stirred at RT for 8 h, after which time TLC analysis confirmed complete consumption of starting material. The solution was then quenched with a few drops of Et_3N (until pH 7 was achieved) and concentrated *in vacuo*. The residue obtained was purified using flash column chromatography over silica gel with a Biotage system (petroleum ether:EtOAc, 10:0 to 7:3) to give alcohol **16** (870 mg, 76%) as a pale-yellow oil: R_f 0.63 (petroleum ether:EtOAc, 1:1); $[\alpha]_D^{25}$ −24.4 (*c* 1.0, $CHCl_3$), [lit.[5] $[\alpha]_D^{25}$ −25.3 (*c* 1.0, $CHCl_3$)]; ^{1}H NMR (600 MHz, $CDCl_3$) δ_H 7.33–7.28 (2 H, m, H-9 and H-13), 7.27–7.23 (2 H, m, H-17 and H-21), 6.91–6.84 (4 H, m, H-10, H-12, H-18, and H-20), 5.15 (1 H, dd, *J* 8.3, 3.8, H-1), 4.84 (1 H, d, *J* 11.2, H-7'), 4.72 (1 H, d, *J* 11.2, H-15'), 4.68 (1 H, d, *J* 11.2, H-15''), 4.63 (1 H, d, *J* 11.2, H-7''), 4.31 (1 H, dd, *J* 5.5, 3.8, H-2), 4.14 (1 H, dd, *J* 5.9, 5.9, H-3), 3.80 (3 H, s, H-14 or H-22), 3.80 (3

H, s, H-14 or H-22), 3.77 (1 H, dd, *J* 7.9, 7.9, H-6), 3.63–3.57 (2 H, m, H-4 and H-5), 2.62 (1 H, s, -OH), 2.11 (3 H, s, H-24), 1.98–1.85 (2 H, m, H-26 or H-29), 1.76–1.62 (6 H, m, H-26 or H-29, H-27 and H-28); LRMS *m/z* (ESI$^+$) 551.2 ([M+Na]$^+$, 100%). These data are in good agreement with the literature values.[5]

(1,5-Dihydro-2,4,3-benzodioxaphosphepin-3-yl)diethylamine 17

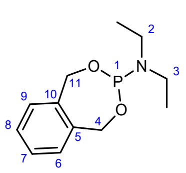

To a two-neck round bottomed flask equipped with a stirrer bar, was added anhydrous Et_2O and freshly distilled PCl_3 (2.54 mL, 29.1 mmol, 1.0 eq) under an atmosphere of argon. The solution was cooled to −78° C, after which a solution of freshly distilled anhydrous diethylamine (6.03 mL, 58.2 mmol 2.0 eq) in anhydrous Et_2O (232 mL) was added dropwise *via* cannula. The reaction mixture was stirred for 1.5 h before being allowed to reach RT and then stirred for an additional 16 h. The resulting precipitate was removed by Schlenk filtration, and the filtrate collected in a two-neck round bottomed flask equipped with a stirrer bar. The filtered solid was washed with anhydrous Et_2O (3 × 50 mL) and the filtrate was then cooled to −78 °C under an atmosphere of argon. A solution of Et_3N (8.11 mL, 58.3 mmol, 2.0 eq) and 1,2-benzenedimethanol (4.02 g, 29.1 mmol, 1.0 eq) in a mixture of anhydrous THF (40 mL) and anhydrous Et_2O (160 mL) was cooled to −78 °C and then added dropwise *via* cannula to the stirred filtrate. After complete addition of the 1,2-benzenedimethanol solution, the reaction was allowed to proceed for 1.5 h, before being allowed to reach RT. The reaction mixture was then stirred for a further 16 h. The resulting precipitate was removed using Schlenk filtration, and the filtered solid was washed with anhydrous Et_2O (3 × 50 mL). The filtrate was then concentrated *in vacuo* to give (1,5-dihydro-2,4,3-benzodioxaphosphepin-3-yl)diethylamine **17** as a colorless oil (5.26 g, 76%). Analysis by ^{31}P NMR showed that the product was ~87% pure. This unstable phosphoramidite was therefore used in subsequent steps without further purification or characterization. The product was stored under Ar at −20 °C and was checked by ^{31}P NMR before

each use: ^{1}H NMR (400 MHz; $CDCl_3$) δ_H 7.31–7.16 (4H, m, H-6 to H-9), 5.18 (2H, dd, *J* 13.8, 7.0, H-4 and H-11), 4.91 (2H, dd, *J* 19.4, 13.8, H-4' and H-11'), 3.19 (4H, dq, *J* 9.9, 7.0, H-2 and H-3), 1.11 (6H, t, *J* 7.0, C(2)CH_3 and C(3)CH_3); ^{31}P NMR (162 MHz, $CDCl_3$) δ_P 145.3 (P-1); These data are in good agreement with the literature values.[5]

1D-1-*O*-Acetate-2,3-*O*-cyclopentylidene-4,6-di-*O*-(4-methoxybenzyl)-5-*O*-(2-oxo-5.6-benzo-1,3,2-dioxaphosphep-2-yl)-*myo*-inositol (−)-18

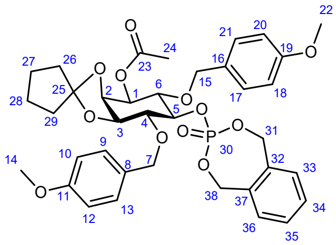

To phosphoramidite **17** (770 mg, 3.22 mmol, 2.0 eq) in anhydrous CH_2Cl_2 (8 mL) was added 1*H*-tetrazole (7.16 mL, 3.22 mmol, 2.0 eq, 0.45 M in MeCN). After stirring at RT for 10 min, a solution of alcohol **(−)-16** (850 mg, 1.61 mmol, 1.0 eq) in anhydrous CH_2Cl_2 (2 mL) was added dropwise. The cloudy reaction mixture was allowed to stir for 16 h, after which the reaction mixture was cooled to −78 °C and *m*CPBA (722 mg, 3.22 mmol, 2.0 eq, 77%) was added in a single portion. The reaction mixture was stirred at −78 °C and then gradually warmed to RT and stirred for a further 3 h. The reaction was then quenched with an aqueous solution of $Na_2S_2O_3$ (10% *w/v*, 20 mL) and the aqueous layer was extracted with CH_2Cl_2 (3 × 20 mL). The combined organic layers were washed with brine (60 mL), dried over Na_2SO_4, filtered, and concentrated *in vacuo*. Purification using column chromatography over silica gel (petroleum ether:EtOAc, 10:0 to 6:4) afforded **(−)-18** (1.06 g, 92%) as a colorless foam: R_f 0.34 (petroleum ether:EtOAc, 1:1); $[\alpha]_D^{25}$ −2.9 (*c* 1.0, $CHCl_3$); ν_{max}(thin film)/cm^{-1} 2398, 1744, 1613, 1514, 1247, 1017; ^{1}H NMR (600 MHz, $CDCl_3$) δ_H 7.34–7.31 (4 H, m, H-9, H-13, H-33, H-36), 7.29–7.26 (2 H, m, H-17, H-21), 7.21–7.17 (2 H, m, H-34 and H-35), 6.87–6.81 (4 H, m, H-10, H-12, H-18, H-20), 5.23 (1 H, dd, *J* 8.5, 3.6, H-1), 5.18 (1 H, dd, *J* 15.8, 13.9, H-31' or H-38'), 5.12 (1 H, dd, *J* 16.4, 13.9, H-31' or H-38'), 5.09–5.00 (2 H, m, H-37'' and H-38''), 4.77 (1 H, d, *J* 10.8, H-15'), 4.76 (1 H, d, *J* 11.1, H-7'), 4.68 (1 H, d, *J* 11.2, H-7''), 4.63–4.58 (2 H, m, H-5, H-15''), 4.38 (1 H, dd, *J* 6.3, 3.6, H-2), 4.20 (1 H,

dd, *J* 6.0, 6.0, H-3), 4.02 (1 H, dd, *J* 8.5, 6.8, H-6), 3.89 (1 H, dd, *J* 7.5, 6.0, H-4), 3.79 (6 H, s, H-14 and H-22), 2.08 (3 H, s, H-24), 1.98–1.85 (2 H, m, H-26’, H-29’), 1.73–1.61 (6 H, m, H-26’’, H-27, H-28, H-29’’); ^{31}P NMR (243 MHz, $CDCl_3$) δ_P −0.7 (P-30); ^{13}C NMR (151 MHz, $CDCl_3$) δ_C 170.1 (C-23), 159.35, 159.34 (C-11 and C-19), 135.49, 135.45 (C-32 and C-37), 130.33, 130.23 (C-8 and C-16), 129.82 (C-9 and C-13), 129.63 (C-17, C-21), 129.07, 129.04 (C-33 and C-36), 128.8 (C-34 and C-35), 120.1 (C-25), 113.85, 113.76 (C-10, C-12, C-18, C-20), 80.8 (d, *J* 6.5, C-5), 78.9 (d, *J* 3.3, C-4), 77.7 (d, *J* 4.4, C-6), 77.6 (C-3), 68.5 (d, *J* 2.2, C-31 or C-38), 68.4 (d, *J* 2.7, C-31 or C-38), 74.0 (C-15), 73.7 (C-2), 72.9 (C-7), 70.5 (C-1), 68.45 (d, *J* 2.2, C-31 or C-38), 68.41 (d, *J* 2.7, C-31 or C-38), 55.4 (C-14 and C-22), 36.9, 36.7, 24.1, 23.5 (C-26, C-27, C-28 and C-29), 21.2 (C-24); HRMS *m/z* (ESI$^+$) found 711.2551 [M+H]$^+$ ($C_{37}H_{44}O_{12}P$ requires 711.2565 [M+H]$^+$).

1D-2,3-*O*-Cyclopentylidene-4,6-di-*O*-(4-methoxybenzyl)-5-*O*-(2-oxo-5.6-benzo-1,3,2-dioxaphosphep-2-yl)-*myo*-inositol (+)-19

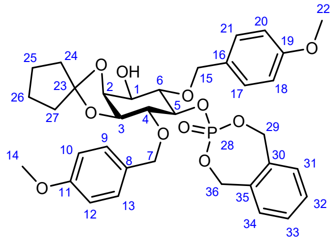

To a solution of **(−)-18** (600 mg, 0.884 mmol, 1.0 eq) in MeOH (80 mL) was added ground K_2CO_3 (233 mg, 1.68 mmol, 2.0 eq). The reaction mixture was stirred at RT for 1 h, after which TLC confirmed the reaction had gone to completion. The solution was neutralized with 1 M HCl (pH 7) and the volatiles partially removed *in vacuo*. H_2O (30 mL) was added to the resulting residue and the aqueous layer was extracted with EtOAc (4 × 10 mL). The combined organic fractions were washed with brine (40 mL), dried over Na_2SO_4, filtered, and concentrated *in vacuo*. The resulting residue was purified by column chromatography over silica gel (petroleum ether:EtOAc, 10:0 to 3:7) and afforded alcohol **(+)-19** (462 mg, 82%) as a colorless solid: R_f 0.34 (petroleum ether/EtOAc, 1:1); $[\alpha]_D^{25}$ +0.5 (*c* 2.0, $CHCl_3$); m.p. 125–127 °C (MeCN); ν_{max} (thin film)/cm^{-1} 2938, 1613, 1514, 1249, 1016; ^{1}H NMR (600 MHz, $CDCl_3$) δ_H

7.35–7.28 (6 H, m, H-9, H-13, H-17, H-21, H-31, H-34), 7.24–7.15 (2 H, m, H-32, H-33), 6.88–6.85 (2 H, m, H-10, H-12 or H-18, H-20), 6.83–6.80 (2 H, m, H-10, H-12 or H-18, H-20), 5.22–5.03 (4 H, m, H-29 and H-36), 4.87 (1 H, d, *J* 10.8, H-7’ or H-15’), 4.77 (1 H, d, *J* 11.2, H-7’ or H-15’), 4.66 (1 H, d, *J* 11.2, H-7’’ or H-15’’), 4.63–4.58 (2 H, m, H-5 and H-7’’ or H-15’’), 4.35 (1 H, dd, *J* 6.5, 3.1, H-2), 4.20 (1 H, dd, *J* 6.5, 6.5, H-3), 3.99–3.93 (3 H, m, H-1, H-4, H-6), 3.79 (3 H, s, H-14 or H-22), 3.78 (3 H, s, H-14 or H-22), 2.49 (1 H, d, *J* 3.1, C(1)O*H*), 1.99–1.88 (2 H, m, C-24’ and C-27’’), 1.79–1.64 (6 H, m, H-24’’, H-25, H-26, H-27’’); ^{31}P NMR (243 MHz, $CDCl_3$) δ_P −0.7 (P-28); ^{13}C NMR (151 MHz, $CDCl_3$) δ_C 159.5, 159.3 (C-11 and C-19), 135.6, 135.5 (C-30 and C-35), 130.4, 130.3 (C-8 and C-16), 130.0 (C-9 and C-13), 129.7 (C-17 and C-21), 129.1 (d, *J* 7.1, C-32 or C-33), 128.9 (d, *J* 6.5, C-32 or C-33), 119.9 (C-23), 114.0, 113.7 (C-10, C-12, C-18 and C-20), 81.1 (d, *J* 6.5, C-5), 79.7 (d, *J* 3.8, H-4 or H-6), 79.6 (d, *J* 3.8, H-4 or H-6), 77.6 (C-3), 75.1 (C-2), 73.9, 72.9 (C-7, C-15), 68.8 (C-1), 68.5 (d, *J* 6.5, C-29 or C-36), 68.4 (d, *J* 6.0, C-29 or C-36), 55.41, 55.39 (C-14 and C-22), 36.6, 24.1, 23.4 (C-24, C-25, C-26, and C-27); LRMS (ESI$^+$) 691 ([M+Na]$^+$, 100%); HRMS *m/z* (ESI$^+$) found 669.2479 [M+H]$^+$ ($C_{35}H_{41}O_{11}P$ requires 669.2459 [M+H]$^+$).

1-Tetrahydropyrannyloxy-11-bromoundecane 21

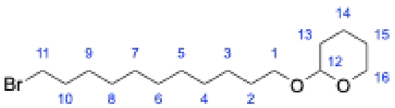

To a solution of 1-bromo-11-hydroxyundecane **20** (20.0 g, 79.6 mmol, 1 eq) in dichloromethane (250 mL) was added 3,4-dihydro-2*H*-pyran (30.1 g, 368 mmol, 4.5 eq) and pyridinium *p*-toluene sulfonate (1.20 g, 4.78 mmol, 0.06 eq). The resulting solution was stirred for 18 h at RT. The reaction was then quenched by the addition of solid sodium hydrogen carbonate (1 g) and dried by vigorously stirring the solution with $MgSO_4$. The mixture was then filtered through a large plug of silica gel, which was subsequently washed with petroleum ether. The

combined organic filtrates were then concentrated *in vacuo* to give 1-tetrahydropyrannyloxy-11-bromoundecane **21** as a colorless oil (26.4 g, 99%): R_f 0.73 (petroleum ether:EtOAc, 9:1); ^{1}H NMR (400 MHz, $CDCl_3$) δ_H 4.57 (dd, *J* 4.5, 2.7, 1H, H-12), 3.92–3.81 (m, 1H, H-16’), 3.72 (dt, *J* 9.6, 6.9, 1H, H-13’), 3.56–3.44 (m, 1H, H-16”), 3.44–3.32 (m, 3H, H-1, H-13’’), 1.93–1.22 (m, 24H, H-2 to H-11, H-14, H-15); LRMS *m/z* (ESI$^+$) 357.162 ([M+Na]$^+$, 100%); These data are in good agreement with the literature values.[6]

13-{(Tetrahydropyran-2'-yl)oxy}tridecan-1-yne 22

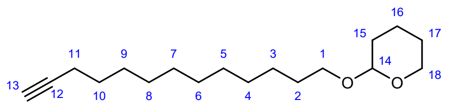

A solution of lithium acetylide (ethylenediamine complex 90%, 3.62 g, 39.4 mmol, 1.32 eq) in anhydrous DMSO (20 mL) was added dropwise to a solution of 1-tetrahydropyrannyloxy-11-bromoundecane **21** in anhydrous DMSO (50 mL) under an atmosphere of argon. The reaction was stirred for 16 h, after which the reaction was quenched with distilled H_2O (40 mL) until effervescence ceased. The mixture was then extracted with Et_2O (4 × 40 mL), the combined organic extracts were dried over Na_2SO_4 and were then concentrated *in vacuo.* The residue was purified by column chromatography over silica gel (petroleum ether:Et_2O, 10:0 to 9:1) resulting in **22** as a colorless oil (5.45 g, 65%): R_f 0.52 (petroleum ether:Et_2O, 9:1); ν_{max} (thin film)/cm^{-1} 3312, 2929, 2855, 2118, 1034, 631; ^{1}H NMR (400 MHz, $CDCl_3$) δ_H 4.57 (1H, dd, *J* 4.4, 2.7, H-14), 3.87 (1H, m, H-18’), 3.72 (1H, dt, *J* 9.6, 6.8, H-1’), 3.53–3.46 (1H, m, H-18’’), 3.38 (1H, dt, *J* 9.6, 6.8, H-1’’), 2.17 (2H, td, *J* 7.1, 2.7, H-11), 1.93 (1H, t, *J* 2.7, H-13), 1.88–1.23 (m, 24H, H-2 to H-10, H-15 to H-17);^{13}C NMR (101 MHz, $CDCl_3$) δ_C 99.0 (C-14), 84.9 (C-12), 68.2 (C-13), 67.8 (C-1), 62.5 (C-18), 31.0 (C-15), 29.9, 29.70, 29.68, 29.62, 29.2, 28.9, 28.7, 26.4, 25.7 (C-2 to C-10, C-16, C-17) 19.9 (C-15), 18.5

(C-11); LRMS *m/z* (ESI$^+$) 303.189 ([M+Na]$^+$, 100%); HRMS *m/z* (ESI$^+$) found 303.2299 [M+Na]$^+$ ($C_{18}H_{32}O_2Na$ requires 303.2295 [M+Na]$^+$).

Tridec-12-yn-1-ol 23

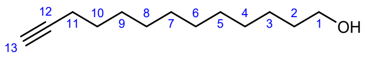

To a solution of THP-protected alcohol **22** (5.00 g, 17.8 mmol, 1 eq) in ethanol (89 mL), was added $HCl_{(aq)}$(2 M, 0.89 mL). The mixture was heated under reflux for 16 h, after which the reaction was diluted with distilled H_2O (100 mL). The aqueous solution was then extracted with Et_2O (3 × 100 mL). The combined extracts were then dried over Na_2SO_4 and were then concentrated under a stream of nitrogen resulting in tridec-12-yn-1-ol **23** as a colorless solid (3.35 g, 96%): R_f 0.43 (petroleum ether:EtOAc, 8:2); m.p. 28–30 °C (Et_2O) [lit.[7] 30–32 °C]; ^{1}H NMR (400 MHz, $CDCl_3$) δ_H 3.64 (2H, t, *J* 6.6, H-1), 2.18 (2H, td, *J* 7.1, 2.7, H-11), 1.93 (1H, t, *J* 2.7, H-13), 1.62–1.47 (4H, m, H-10, H-2), 1.45–1.24 (14H, m, H-3 to H-9). These data are in good agreement with the literature values.[7,8]

Tridec-12-ynal 24

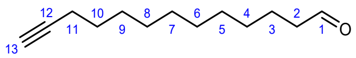

Anhydrous DMSO (11.6 mL) in CH_2Cl_2 (30 mL) was added dropwise over 10 min to a stirred solution of oxalyl chloride (7 mL) in CH_2Cl_2 (150 mL) at –78 °C, under an atmosphere of argon. After 30 min, tridec-12-yn-1-ol **23** (7.24 g, 40.7 mmol, 1 eq) in CH_2Cl_2 (60 mL) was added dropwise *via* cannula over 30 min. Following 1 h of stirring, 35 mL of Et_3N was added, and the reaction was allowed to stir for a further 1.5 h at –78 °C. The reaction was then allowed to reach RT and stirred for a further 3 h. The reaction mixture was then washed with 5% aqueous HCl (200 mL), saturated $NaHCO_{3(aq)}$ (300 mL) and finally brine (300 mL), before being dried over Na_2SO_4, and then concentrated *in vacuo* to afford

tridec-12-ynal **24** as a brown oil (5.23 g, 66%): R_f 0.67 (petroleum ether:EtOAc, 9:1); ^{1}H NMR (400 MHz, $CDCl_3$) δ_H 9.76 (1H, t, *J* 1.9, H-1), 2.41 (2H, td, *J* 7.4, 1.9, H-2), 2.18 (2H, td, *J* 7.1, 2.7, H-11), 1.93 (1H, t, *J* 2.7, H-13), 1.67–1.58 (2H, m, H-10 or H-3), 1.56–1.47 (2H, m, H-10 or H-3), 1.43–1.24 (12H, m, H-4 to H-9). These data are in good agreement with the literature values.[8]

Ethyl 4-oxohexadec-15-ynoate 25

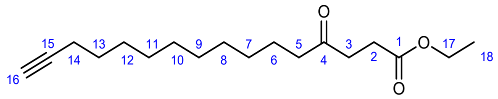

Tridec-12-ynal **24** (6.66 g, 34.3 mmol, 1.0 eq) and ethyl acrylate (8.00 mL, 73.5 mmol, 2.1 eq) in 1,4-dioxane (75 mL) was added dropwise *via* cannula over 2 h to a suspension of 3-benzyl-5-(2-hydroxyethyl)-4-methylthiazolium chloride (1.30 g, 4.80 mmol, 0.14 eq) in Et_3N (3.35 mL, 24.0, mmol, 0.7 eq) and 1,4-dioxane (75 mL) at 80 °C under an atmosphere of argon and the reaction was stirred for 56 h. The reaction mixture was then concentrated *in vacuo* and the resultant residue was redissolved in CH_2Cl_2 (250 mL), which was washed in succession with 10% $H_2SO_{4(aq)}$ (250 mL), saturated $NaHCO_3$ (250 mL), and then brine (250 mL). The organic mixture was then dried over Na_2SO_4, filtered, and concentrated *in vacuo*. Purification using silica gel column chromatography (petroleum ether:EtOAc, 100:0 to 93:7) resulted in **25** (3.85 g, 41%) with an unknown impurity that could not feasibly be separated on large scale. The bulk of the material was used without further purification; however, a small amount was further purified using column chromatography over a slow gradient and large volume of silica gel (petroleum ether:EtOAc, 100:0 to 95:5) for analysis: R_f 0.76 (petroleum ether:EtOAc, 8:2); m.p. 34–35 °C (petroleum ether:EtOAc); ν_{max} (thin film)/cm^{-1} 3297, 2927, 1735, 1716, 1186, 631; ^{1}H NMR (600 MHz, $CDCl_3$) δ_H 4.12 (2 H, q, *J* 7.2, H-17), 2.71 (2 H, t, *J* 6.6, H-3), 2.57 (2 H, t, *J* 6.6, H-2), 2.43 (2 H, t, *J* 7.5, H-5), 2.17 (2 H, td, *J* 7.2, 2.6, H-14), 1.93 (1 H, t, *J* 2.6, H-16), 1.61–1.55 (2 H, m, H-6), 1.54–1.49 (2 H, m, H-13), 1.41–1.34 (2 H, m, H-12), 1.31–1.26 (10 H, m, H-7 to H-11), 1.25 (3 H, t, *J* 7.2, H-18); ^{13}C NMR (151 MHz, $CDCl_3$) δ_C 209.3 (C-4), 173.0 (C-1), 84.9 (C-15), 68.2 (C-16), 60.7 (C-17), 43.0 (C-5), 37.2 (C-3), 29.6, 29.51, 29.50, 29.3, 29.2 (C-11 to C-7), 28.9 (C-12), 28.6 (C-13), 28.2 (C-2), 23.9 (C-

6), 18.5 (C-14), 14.3 (C-18); HRMS *m/z* (ESI$^+$) found 295.2265 [M+H]$^+$ ($C_{18}H_{31}O_3$ requires 295.2268 [M+H]$^+$).

4-Oxohexadec-15-ynoic acid 26

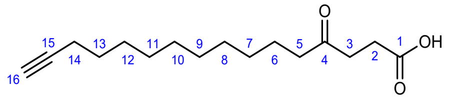

To a solution of ethyl 4-oxohexadec-15-ynoate **25** (3.18 g, 10.8 mmol, 1.0 eq) in methanol (82 mL), was added LiOH (1.29 g, 54.0 mmol, 5.0 eq) and H_2O (0.97 mL, 54 mmol, 5.0 eq). The mixture was stirred at RT for 16 h before the solution was half-concentrated *in vacuo* and then acidified to a pH of 3 using aqueous HCl (1 M). The resulting solution was then extracted with dichloromethane and the combined organic layers were then dried over anhydrous Na_2SO_4. The solution was then concentrated *in vacuo* and crystallized under a stream of nitrogen which resulted in 4-oxohexadec-15-ynoic acid **26** as a colorless crystalline solid (2.85 g, 99%): R_f 0.21 (petroleum ether:EtOAc:AcOH, 80:19:1); m.p. 75–80 °C (CH_2Cl_2); ν_{max} (thin film)/cm^{-1} 3284, 2851, 1702, 906, 729, 647; ^{1}H NMR (600 MHz, $CDCl_3$) δ_H 2.71 (2 H, t, *J* 6.5, H-3), 2.63 (2 H, t, *J* 6.5, H-2), 2.44 (2 H, t, *J* 7.4, H-5), 2.17 (2 H, td, *J* 7.1, 2.6, H-14), 1.93 (1 H, t, *J* 2.6, H-16), 1.61–1.55 (2 H, m, H-6), 1.55–1.48 (2 H, m, H-13), 1.41–1.34 (2 H, m, H-12), 1.32–1.24 (10 H, m, H-11 to H-7); ^{13}C NMR (151 MHz, $CDCl_3$) δ_C 209.1 (C-4), 178.1 (C-1), 84.9 (C-15), 68.2 (C-16), 42.9 (C-5), 36.9 (C-3), 29.5, 29.5, 29.5, 29.3, 29.2 (C-7 to C-11), 28.9 (C-12), 28.6 (C-13), 27.8 (C-2), 23.9 (C-6), 18.5 (C-14); LRMS *m/z* 531.309 ([2M−H]$^-$, 100%); HRMS *m/z* (ESI$^+$) found 267.1955 [M+H]$^+$ ($C_{16}H_{27}O_3$ requires 267.1955 [M+H]$^+$).

3-[3-(Dodec-11-yn-1-yl)diazirin-3-yl]propanoic acid 27

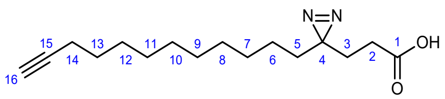

A dried round bottom flask containing acid **26** (1.00 g, 3.25 mmol, 1.0 eq) under an atmosphere of argon was cooled to 0 °C and was charged with 7 M NH_3 in methanol (60 mL). The resulting solution was stirred at 0 °C for 3 h before hydroxylamine-*O*-sulfonic acid (594 mg, 5.25 mmol, 1.4 eq) was added as a single batch. The resulting solution was stirred at 0 °C for 1 h and was then allowed to warm to room temperature over 16 h. The resulting suspension was evaporated to dryness *in vacuo* and resuspended in anhydrous methanol (30 mL). The solid by-product was filtered off through a PTFE syringe filter and the filtrate was collected in a dry RBF, equipped with a stir bar under argon. The solution was cooled to 0 °C and anhydrous diisopropylethylamine (2 mL) was added, followed by iodine (portion-wise), until a dark brown colour persisted for more than 30 min — indicating total oxidation of the diaziridine intermediate. The solution was then partially concentrated before being diluted with EtOAc (60 mL) and washed with 1 M HCl (60 mL, 10% (w/v), $Na_2S_2O_4$ (60 mL portions until clarified) and brine (60 mL). Volatiles were removed *in vacuo* and the resulting residue was purified by silica gel column chromatography (petroleum ether:EtOAc , 100:0 to 88:12 with 0.1% acetic acid), resulting in diazirine **27** as a colorless waxy amorphous solid (467 mg, 52%): R_f 0.48 (petroleum ether:EtOAc:AcOH, 79:20:1); ν_{max} (thin film)/cm^{-1} 3288, 2852, 1694, 1462, 932; ^{1}H NMR (600 MHz, $CDCl_3$) δ_H 2.20–2.12 (4 H, m, H-2, H-14), 1.93 (1 H, t, *J* 2.6, H-16), 1.73 (2 H, t, *J* 7.8, H-3), 1.52 (2 H, p, *J* 7.2, H-13), 1.38 (4 H, m, H-12, H-5), 1.32–1.18 (10 H, m, H-7 to H-11), 1.10–1.04 (2 H, m, H-6); ^{13}C NMR (151 MHz, $CDCl_3$) δ_C 177.9 (C-1), 84.9 (C-15), 68.2 (C-16), 32.8 (C-5), 29.54, 29.52, 29.47, 29.3, 29.2 (C-7 to C-11), 28.9 (C-12), 28.6 (C-13), 28.4 (C-2), 28.20 (C-4), 28.17 (C-3), 23.9 (C-6), 18.5 (C-14); HRMS *m/z* (ESI$^-$) found 277.1919 [M−H]$^-$ ($C_{16}H_{25}O_2N_2$ requires 277.1911 [M−H]$^-$).

(−)-(*S*)-(2,2-Dimethyl-1,3-dioxolan-4-yl)methyl palmitate (−)-29

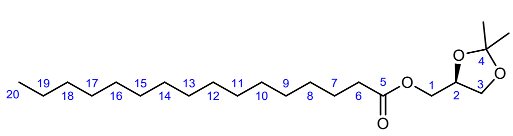

N,*N'*-Dicyclohexylcarbodiimide (6.24 g, 30.2 mmol, 2.0 eq) was added to a stirred solution of (*R*)-(−)-2,3-isopropylidene-*sn*-glycerol **(−)-28** (2.00 g, 15.1 mmol, 1.0 eq), palmitic acid (3.88 g, 15.1 mmol, 1.0 eq) and 4-DMAP (0.11 g, 0.91 mmol, 0.06 eq) in CH_2Cl_2 (45 mL) and was stirred for 16 h. The mixture was then diluted with hexane (20 mL), filtered through Celite® and washed with hexane (3 × 10 mL). The combined filtrates were concentrated *in vacuo*. The residue obtained was purified by column chromatography over silica gel (petroleum ether:EtOAc , 95:5) to yield **(−)-29** (4.33 g, 77%) as a colorless solid: R_f 0.18 (petroleum ether:EtOAc, 20:1); m.p. 29–30 °C (heptane:EtOAc), [lit.[9] 32.0–32.5 °C, pentane:EtOAc)]; $[\alpha]_D^{25}$ −8.4 (*c* 1.0, hexane), [lit.[9] $[\alpha]_D^{20}$ −7.91 (c 3.14, hexane)]; ^{1}H NMR (400 MHz, $CDCl_3$) δ_H 4.35–4.28 (1H, m, H-2), 4.16 (1H, dd, *J* 11.5, 4.7, H-1'), 4.10 (1H, d, *J* 6.2, H-3'), 4.09–4.05 (1H, m, H-1''), 3.74 (1H, dd, *J* 8.4, 6.2, H-3''), 2.34 (2H, t, *J* 7.6, H-6), 1.67–1.57 (2H, m, H-7), 1.44 (3H, s, C(4)CH_3), 1.37 (3H, s, C(4)CH_3), 1.33–1.23 (29H, m, H-8 to H-19), 0.92–0.84 (3H, m, H-20); LRMS *m/z* (ESI$^+$) 393.350 ([M+Na]$^+$, 100%); These data are in good agreement with the literature values.[9]

(+)-(*R*)-2-Hydroxy-3-((triethylsilyl)oxy)propyl palmitate 30

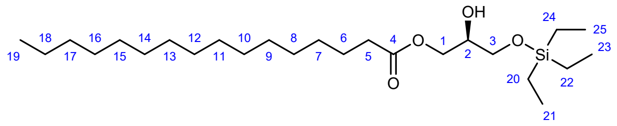

Acetonide **(−)-29** (2.04 g, 5.50 mmol, 1.0 eq) was dissolved in anhydrous dichloroethane (40 mL) under an atmosphere of argon. DIPEA (1.92 mL, 11.0 mmol, 2 eq) was added followed by TESOTf (1.49 mL, 6.60 mmol, 1.2 eq) and the mixture was stirred at reflux for 24 h. Additional TESOTf (0.50 mL, 2.20 mmol, 0.4 eq) was then added due to presence of remaining starting material, and the mixture was stirred for a further 24 h. The reaction was cooled to RT, diluted with petroleum ether and washed with 0.1 M aqueous HCl. The aqueous fraction was extracted with petroleum ether (3 × 10 mL). The combined organic

fractions were then washed with brine, dried over Na_2SO_4 and concentrated *in vacuo*. The resulting residue was then dissolved in a mixture of THF (42 mL) and 10% aqueous Na_2CO_3 (18 mL), before I_2 (2.09 g, 8.25 mmol, 1.5 eq) was added. The reaction was stirred for 2 h and then quenched with 10% aqueous $Na_2S_2O_3$ (100 mL). The solution was extracted with Et_2O (3 × 100 mL), and the combined Et_2O fractions were washed with brine (300 mL), dried over Na_2SO_4, filtered, and concentrated *in vacuo*. The resulting residue was then purified by silica gel column chromatography (cyclohexane:EtOAc, 100:0 to 90:10) giving alcohol **(+)-30** as a yellow oil (438 mg, 18%): R_f 0.17 (petroleum ether:EtOAc, 95:5); $[\alpha]_D^{25}$ +1.4 (*c* 4.0, $CHCl_3$); ^1H NMR (400 MHz, $CDCl_3$) δ_H 4.16 (1 H, dd, *J* 11.5, 5.0, H-1'), 4.11 (1 H, dd, *J* 11.5, 5.9, H-1''), 3.92–3.84 (1 H, m, H-2), 3.67 (1 H, dd, *J* 10.1, 4.6, H-3'), 3.60 (1 H, dd, *J* 10.1, 5.7, H-3''), 2.54 (1 H, d, *J* 5.3, C(2)O*H*), 2.34 (2 H, t, *J* 7.5, H-5), 1.62 (2 H, p, *J* 7.5, H-6), 1.35–1.23 (24 H, m, H-7 to H-18), 0.96 (9 H, t, *J* 7.9, H-21, H-23, H-25), 0.88 (3 H, t, *J* 6.7, H-19), 0.62 (6 H, q, *J* 7.9, H-20, H-22, H-24); LRMS (ESI$^+$) 467.3 ([M+Na]$^+$, 100%), 911.5 ([2M+Na]$^+$, 100%); These data are in good agreement with the literature values.[10]

(+)-(*R*)-2-((3-(3-(Dodec-11-yn-1-yl)-3*H*-diazirin-3-yl)propanoyl)oxy)-3-((triethylsilyl)oxy)propyl palmitate 31

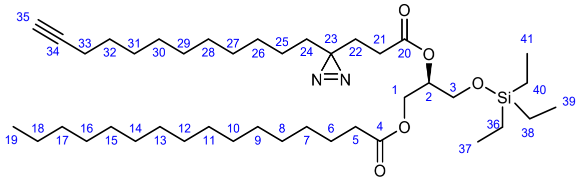

To a solution of alcohol **(+)-30** (240 mg, 0.540 mmol, 1.0 eq) in anhydrous CH_2Cl_2 (2 mL) was added DCC (223 mg, 1.08 mmol, 2.0 eq), 4-DMAP (4 mg, 0.03 mmol, 0.06 eq) and diazirine **50** (150 mg, 1.08 mmol, 1.0 eq). The solution was stirred at RT for 18 h, after which the reaction mixture was diluted with hexane (10 mL). The suspension was filtered through a PTFE syringe filter, and the filtrate was concentrated *in vacuo* at RT. The resulting residue was purified by column chromatography over silica gel (petroleum ether:Et_2O, 10:0 to 9:1) which yielded **(+)-31** (324 mg, 85%) as a colorless oil:

R_f 0.44 (hexane:Et_2O, 90:10); $[\alpha]_D^{25}$ +8.8 (*c* 1.0, $CHCl_3$); ν_{max} (thin film)/cm^{-1} 3313, 2925, 2854, 1744, 1459, 1167, 1115; ^{1}H NMR (600 MHz, $CDCl_3$) δ_H 5.08–5.04 (1 H, m, H-2), 4.35 (1 H, dd, *J* 11.9, 3.6, H-1’), 4.16 (1 H, dd, *J* 11.9, 6.1, H-1’’), 3.75–3.68 (2 H, m, H-3), 2.30 (1 H, t, *J* 7.5, H-5), 2.17 (2 H, td, *J* 7.2, 2.6, H-33), 2.13–2.09 (2 H, m, H-21), 1.93 (1 H, t, *J* 2.6, H-35), 1.72 (1 H, t, *J* 7.8, H-22), 1.60 (2 H, p, *J* 7.4, H-6), 1.52 (1 H, p, *J* 7.2, H-32), 1.41–1.34 (4 H, m, H-31, H-29), 1.34–1.18 (34 H, m, H-7 to H-18 and H-24 to H-28), 1.11–1.03 (2 H, m, H-30), 0.94 (9 H, t, *J* 7.9, H-37, H-39, H-41), 0.88 (3 H, t, *J* 7.0, H-19), 0.59 (6 H, q, *J* 7.9, H-36, H-38, H-40); ^{13}C NMR (151 MHz, $CDCl_3$) δ_C 173.6 (C-4), 171.8 (C-20), 84.9 (C-34), 72.4 (C-2), 68.2 (C-35), 62.4 (C-1), 61.2 (C-3), 34.3 (C-5), 32.9 (C-24), 32.1, 29.8, 29.8, 29.8, 29.6, 29.6, 29.6, 29.5, 29.4, 29.3, 29.3, 29.2, 28.9, 28.8, 28.6, 28.4 (C-7 to C-17, C-21 to C-22, C-26 to C-32), 28.3 (C-23), 25.1 (C-6), 24.0 (C-25), 22.8 (C-18), 18.5 (C-33), 14.3 (C-19), 6.8 (C-37, C-39, C-41), 4.4 (C-36, C-38, C-40); HRMS *m/z* (ESI$^+$) found 727.5410 [M+Na]$^+$ ($C_{41}H_{76}O_5N_2SiNa$ requires 727.5416 [M+Na]$^+$).

(−)-(*S*)-2-((3-(3-(Dodec-11-yn-1-yl)-3*H*-diazirin-3-yl)propanoyl)oxy)-3-hydroxypropyl palmitate 32

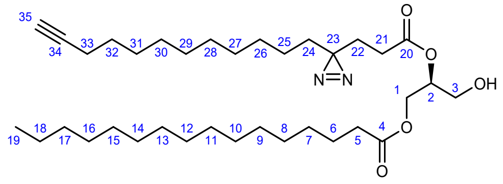

Method 1: Aqueous acetic acid (AcOH:H_2O 4:1 *v/v*, 2 mL) was added to protected diglyceride **S24** (374 mg, 0.419 mmol, 1.0 eq) and stirred vigorously. MeCN was then added dropwise in an attempt to homogenize the solution. The mixture was then stirred at 50 °C for 3.5 h, after which TLC showed remaining starting material. The solution was concentrated *in vacuo* and the resulting residue was redissolved in acetic acid (2 mL) and a few drops of H_2O was added. The solution was stirred for a further 30 min after which the mixture was cooled to RT and concentrated *in vacuo.* The resulting residue was then purified *via* column chromatography over silica gel (petroleum ether:EtOAc, 100:0 to 76:24) giving alcohol **32** as a colorless waxy solid (167 mg, 68%): R_f 0.23 (petroleum ether:EtOAc, 8:2); $[\alpha]_D^{25}$ –3.3 (*c* 1.0,

$CHCl_3$); ν_{max} (thin film)/cm^{-1} 3313, 2981, 2925, 2854, 1742, 1463, 1381, 1251, 1165, 955; ^{1}H NMR (600 MHz, $CDCl_3$) δ_H 5.09 (1 H, p, *J* 4.9, H-2), 4.32 (1 H, dd, *J* 12.0, 4.5, H-1'), 4.23 (1 H, dd, *J* 12.0, 5.6, H-1''), 3.80–3.70 (2 H, m, H-3), 2.32 (2 H, t, *J* 7.5, H-5), 2.20–2.12 (4 H, m, H-33 and H-21), 1.93 (1 H, t, *J* 2.6, H-35), 1.82–1.71 (2 H, m, H-22), 1.65–1.58 (2 H, m, H-6), 1.52 (2 H, p, *J* 7.2, H-32), 1.42–1.34 (4 H, m, H-30 and H-31), 1.34–1.19 (34 H, m, H-28 to H-24 and H-18 to H-7), 1.12–1.04 (2 H, m, H-29), 0.88 (3 H, t, *J* 7.0, H-19); ^{13}C NMR (151 MHz, $CDCl_3$) δ_C 173.9 (C-4), 172.0 (C-20), 84.9 (C-34), 72.9 (C-2), 68.2 (C-35), 62.0 (C-1), 61.6 (C-3), 34.2 (C-5), 32.8 (C-31), 32.1 (C-17), 29.85, 29.83, 29.81, 29.77, 29.62, 29.55, 29.54, 29.51, 29.49, 29.41, 29.29, 29.27, 29.21, 29.20 (C-24 to C-30, C-7 to C-16), 28.9 (C-21), 28.6 (C-32), 28.4 (C-23), 28.3 (C-22), 25.0 (C-6), 23.9 (C-29), 22.8 (C-18), 18.5 (C-33), 14.3 (C-19); LRMS *m/z* (ESI$^-$) 635.5 (M+FA−H, 100%); HRMS *m/z* (ESI$^+$) found 613.4553 $[M+Na]^+$ ($C_{35}H_{62}O_5N_2Na$) requires 613.4551 $[M+Na]^+$).

Method 2: Silane **31** (200 mg, 0.284 mmol, 1.0 eq) was dissolved in a mixture of anhydrous MeOH (2.1 mL) and anhydrous CH_2Cl_2 (0.7 mL). A substoichiometric amount of $FeCl_3$ (2 mg, 0.01 mmol, 0.05 eq) was added and the reaction mixture, which was stirred for 3 h until TLC analysis confirmed complete consumption of the starting material. The solution was diluted with Et_2O (20 mL), washed with H_2O (20 mL), and the aqueous component was extracted further with Et_2O (3 × 20 mL). The combined Et_2O extracts were then washed with brine, dried over Na_2SO_4, filtered, and concentrated *in vacuo*, resulting in alcohol **32** (156 mg, 93%, >95% *e.e.*) as a colorless waxy solid requiring no further purification. No transesterification by-product was observed by ^{1}H NMR, suggesting the reaction did not affect the enantiopurity of the product. $[\alpha]_D^{25}$ –3.5 (*c* 1.0, $CHCl_3$); ^{1}H NMR (600 MHz, $CDCl_3$) δ_H 5.09 (1 H, p, *J* 5.0, H-2), 4.32 (1 H, dd, *J* 12.0, 4.5, H-1'), 4.23 (1 H, dd, *J* 12.0, 5.6, H-1''), 3.80–3.70 (2 H, m, H-3), 2.32 (2 H, t, *J* 7.6, H-5), 2.17 (3 H, td, *J* 7.1, 2.5), H-33), 2.15 (2 H, t, *J* 7.4, H-21), 1.93 (1 H, t, *J* 2.6, H-35), 1.82–1.71 (2 H, m, H-22), 1.61 (2 H, p, *J* 7.4, H-6), 1.52 (2 H, p, *J* 7.2, H-32), 1.41–1.35 (4 H, m, H-30, H-31), 1.32–1.19 (34 H, m,

H-7 to H-18 and H-24 to H-28), 1.10–1.03 (2 H, m, H-29), 0.88 (3 H, t, *J* 7.0, H-19); ^{13}C NMR (151 MHz, $CDCl_3$) δ_C 173.9 (C-4), 172.0 (C-20), 84.9 (C-34), 72.9 (C-2), 68.2 (C-35), 62.0 (C-1), 61.6 (C-3), 34.2 (C-5), 32.8 (C-31), 32.1 (C-17), 29.85, 29.83, 29.81, 29.77, 29.62, 29.55, 29.54, 29.51, 29.49, 29.41, 29.29, 29.27, 29.21, 29.20 (C-30 to C-24, C-16 to C-7), 28.9 (C-21), 28.6 (C-32), 28.4 (C-23), 28.3 (C-22), 25.0 (C-6), 23.9 (C-29), 22.8 (C-18), 18.5 (C-33), 14.3 (C-19). All other characterization data matched those reported above. The *e.e.* of **(–)-32** was calculated by ^{1}H NMR using a chiral auxiliary (see **Figure S3**).

(2*R*)-3-(((Benzyloxy)(diisopropylamino)phosphaneyl)oxy)-2-((3-(3-(dodec-11-yn-1-yl)-3*H*-diazirin-3-yl)propanoyl)oxy)propyl palmitate 34

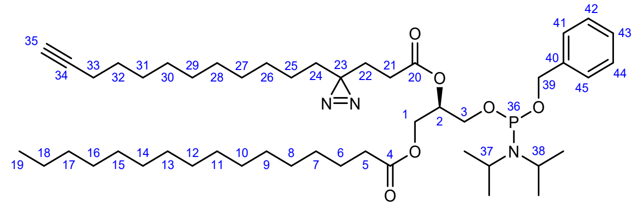

To a solution of benzyloxy-bis(*N*,*N*-diisopropylamino) phosphine **33** (172 mg, 0.507 mmol, 3.0 eq) in anhydrous dichloromethane (1 mL) was added 1*H*-tetrazole (1.13 mL, 0.507 mmol, 3.0 eq, 0.45 M in MeCN). The solution was stirred for 15 min, after which alcohol **32** (100 mg, 0.169 mmol, 1.0 eq) was added as a single batch. The reaction mixture was stirred at RT for 18 h, diluted with CH_2Cl_2 (10 mL), and then quenched by the addition of saturated aqueous $NaHCO_3$ (3 mL). The organic phase was extracted with CH_2Cl_2 (3 × 3 mL). The combined organic layers were washed with brine (10 mL), dried over Na_2SO_4, filtered, and then concentrated *in vacuo*. The residue obtained was purified by column chromatography over silica gel (hexane:EtOAc:Et_3N, 90:5:5) to give phosphoramidite **34** (135 mg, 96%) as a colorless viscous oil, containing a mixture of two diastereoisomers. Due to its unstable nature, this phosphoramidite was used without further characterization: R_f 0.73 (petroleum ether:EtOAc:Et_3N, 90:5:5); $[\alpha]_D^{25}$ +5.3 (*c* 1.0, $CHCl_3$); ν_{max} (thin film)/cm^{-1} 3312, 2926, 2854, 2118, 1743, 1497, 1364, 1184, 976; ^{1}H NMR (400 MHz, $CDCl_3$) δ_H 7.39–7.26 (10 H, m, H-41 to H-45 Diast. A and B), 5.21–5.14 (2 H, m, H-2 Diast. A and B), 4.82–4.61 (4 H, m, H-1 Diast. A and B), 4.36 (1 H, dd, *J* 8.5, 3.7, H-3' Diast. A), 4.32 (1 H, dd, *J* 8.5,

3.7, H-3’ Diast. B), 4.21–4.12 (2 H, m, H-3’’ Diast. A and B), 3.83–3.54 (8 H, m, H-37, H-38, H-39. Diast A and B), 2.29 (4 H, t, *J* 7.6, H-5 Diast. A and B), 2.18 (2 H, td, *J* 7.2, 2.7, H-33 Diast. A and B), 2.12–2.06 (2 H, m, H-21 Diast. A and B), 1.93 (2 H, t, *J* 2.7, H-35), 1.73–1.68 (4 H, m, H-22 Diast. A and B), 1.65–1.56 (4 H, m, H-6 Diast. A and B), 1.56–1.47 (4 H, m, H-32 Diast. A and B), 1.43–1.15 (100H, m, H-31, H-30, H-28 to H-24 and H-18 to H-7, 2 × C(37)CH_3, 2 × C(38)CH_3, Diast. A and B), 1.10–1.00 (4 H, m, H-29 Diast. A and B), 0.88 (6 H, t, *J* 6.6, H-19 Diast. A and B); ^{31}P NMR (162 MHz, $CDCl_3$) δ_P 148.9 (P-36 Diast. A), 148.7 (P-36 Diast. B); HRMS *m/z* (ESI$^+$) found 828.6007 [M+H]$^+$ ($C_{48}H_{83}O_6N_3P$) requires 828.6014 [M+H]$^+$).

(+)-(2*R*)-3-(((Benzyloxy)(((3a*R*,4*R*,5*S*,6*R*,7*S*,7a*R*)-4,6-*bis*((4-methoxybenzyl)oxy)-5-((3-oxido-1,5-dihydrobenzo[e][1,3,2]dioxaphosphepin-3-yl)oxy)hexahydrospiro[benzo[d][1,3]dioxole-2,1'-cyclopentan]-7-yl)oxy)phosphoryl)oxy)-2-((3-(3-(dodec-11-yn-1-yl)-3*H*-diazirin-3-yl)propanoyl)oxy)propyl palmitate (+)-35

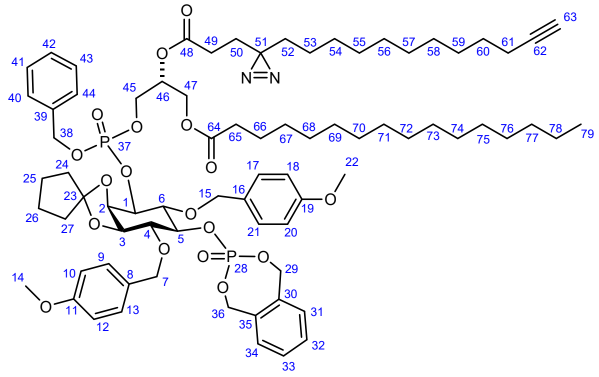

To a solution of phosphoramidite **34** (150 mg, 0.181 mmol, 1.0 eq) in anhydrous CH_2Cl_2 (1.8 mL) was added 1*H*-tetrazole (402 µL, 0.181 mmol, 1.0 eq, 0.45 M in MeCN). After 10 min, inositol **(+)-19** (120 mg, 0.181 mmol, 1.0 eq) was added as a single batch to the cloudy reaction mixture, and the mixture was stirred at RT for a further 18 h. The reaction mixture was then cooled to −78 °C and *m*CPBA (41 mg, 0.181 mmol, 1.0 eq, 77%) was added in one portion. The reaction mixture was stirred for 1 h, warmed to RT, stirred for a further 3 h, and then quenched by the addition of an aqueous solution of $Na_2S_2O_3$ (10% w/v, 5 mL). After stirring for 15 min, the aqueous phase was extracted with CH_2Cl_2 (4 × 5 mL) and the combined organic layers were washed with brine (15 mL), dried over Na_2SO_4, filtered, and then concentrated *in vacuo*. Purification by column chromatography over silica gel (petroleum ether:EtOAc, 10:0, 5:5) yielded protected PI(5)P **(+)-35** (147 mg, 57%) as a colorless, gummy oil containing a mixture of two diastereoisomers: R_f 0.51 (petroleum

ether:EtOAc, 5:5); $[\alpha]_D^{25}$ +2.4 (*c* 0.7, $CHCl_3$); ν_{max}(thin film)/cm^{-1} 2927, 2854, 2360, 2341, 1744, 1515, 1250, 1015, 763; ^{1}H NMR (600 MHz, $CDCl_3$) δ_H 7.38 – 6.75 (34 H, m, H-9, H-10, H-12, H-13, H-17, H-18, H-20, H-21, H-31 to H-34, H-40 to H-44 Diast. A and B), 5.19–4.98 (13 H, m, H-38 Diast. A, H-38' Diast. B, H-29, H-36, H-46 Diast. A and B), 4.94 (1 H, dd, *J* 11.8, 7.6, H-38'' Diast B), 4.78 (2 H, dd, *J* 10.5, 4.1, H-15' Diast. A and B), 4.73 (2 H, dd, *J* 11.3, 4.0, H-7' Diast. A and B), 4.70–4.65 (3 H, m, H-7'' Diast. A, H-1 Diast. A and B), 4.66–4.59 (5 H, m, H-7'' Diast. B, H-15'', H-5 Diast A and B), 4.48 (2 H, dd, *J* 6.4, 3.7, H-2 Diast. A and B), 4.26 (1 H, dd, *J* 12.1, 4.0, H-47' Diast. A), 4.19 (1 H, dd, *J* 6.1, 6.1, H-3 Diast. A), 4.18 (1 H, dd, *J* 6.1, 6.1, H-3 Diast. B), 4.17–4.12 (2 H, m, H-45', H-47'' Diast. A), 4.12–3.97 (6 H, m, H-6, H-45'' Diast. A, H-45', H-45'', H-47' Diast. B), 3.94–3.85 (3 H, m, H-47'' Diast. B, H-4 Diast. A and B), 3.78 (3 H, s, PMB -OCH_3 Diast. A), 3.77 (6 H, s, PMB -OCH_3 Diast. A and B), 3.75 (3 H, s, PMB -OCH_3 Diast B.), 2.27 (2 H, t, *J* 7.6, H-65 Diast. A), 2.25 (2 H, t, *J* 7.6, H-65 Diast. B), 2.17 (4 H, td, *J* 7.2, 2.6, H-61 Diast. A and B), 2.07 (2 H, td, *J* 7.9, 3.1, H-49 Diast. A), 2.02–1.98 (2 H, m, H-49 Diast. B), 1.98–1.85 (6 H, m, H-63, H-24', H-27' Diast. A and B), 1.74–1.61 (16 H, m, H-24'', H-27'', H-25, H-26, H-50 Diast. A and B), 1.59–1.54 (4 H, m, H-66 Diast. A and B), 1.51 (4 H, p, *J* 7.2, H-60 Diast. A and B), 1.41–1.15 (76 H, m, H-52 to H-57, H-59, H-67 to H-78 Diast A. and B), 1.07–0.98 (4 H, m, H-58 Diast. A and B), 0.88 (6 H, t, *J* 7.0, H-79 Diast. A and B); ^{31}P NMR (243 MHz, $CDCl_3$) δ_P −0.64 (P-28 Diast. A), −0.67 (P-28 Diast. B), −1.85 (P-37 Diast. A), −1.97 (P-37 Diast. B); ^{13}C NMR (151 MHz, $CDCl_3$) δ_C 173.33, 173.27 (C-64 Diast. A and B), 171.53, 171.48 (C-48 Diast. A and B), 159.38, 159.37, 159.32 (C-11, C-19 Diast A and B), 135.9 – 135.7 (m, C-39 Diast. A and B), 135.48, 135.46, 135.39 (C-8, C-16, C-30, C-35), 130.1 (d, *J* 2.7), 129.84 (d, *J* 2.2), 129.76, 129.69, 129.11, 129.10, 129.07, 128.89, 128.87, 128.84, 128.83, 128.73, 128.72, 128.67, 128.60, 128.0, 127.8 (Aromatic C-10, C-12, C-18, C-20, C-31, C-32, C-33, C-34, C-40, C-41, C-42, C-43, C-44 Diast. A and B), 120.1 (C-23 Diast. A and B), 113.80, 113.79, 113.77 (C-9, C-13, C-17, C-21 Diast. A and B), 84.9 (C-62 Diast. A and B), 80.50 (C-5 Diast. A), 80.47 (C-5 Diast. B), 78.62

(d, *J* 2.7, C-4, Diast. A), 78.56 (d, *J* 2.7, C-4 Diast. B), 78.2–78.0 (m, C-6 Diast. A), 78.0–77.8 (m, C-6 Diast. B), 77.2 (C-3 Diast. A and B by HSQC), 75.35 (C-1 Diast. A), 75.31 (C-1 Diast. B), 74.24 (C-2 Diast. A), 74.17 (C-2 Diast. B), 74.0 (d, *J* 2.7, C-15 Diast. A), 72.8 (d, *J* 4.4, C-15 Diast. B), 70.04 (d, *J* 3.3, C-46 Diast. A), 69.98 (d, *J* 3.8, C-46 Diast. B), 69.6 (t, *J* 5.7, C-38 Diast. A and B), 68.5–68.4 (m, C-29 and C-36 Diast. A and B), 68.2 (C-63 Diast. A and B), 65.7 (d, *J* 5.4, C-45 Diast. A), 65.4 (d, *J* 5.5, C-45 Diast. B), 61.7 (C-47 Diast. A), 61.6 (C-47 Diast. B), 55.39, 55.36, 55.35 (C-14, C-22 Diast A and B), 36.57, 36.56, 36.39, 36.36 (C-24, C-27 Diast. A and B), 34.11 (C-65 Diast A), 34.07 (C-65 Diast. B), 32.81, 32.77, 32.1, 29.85, 29.82, 29.82, 29.81, 29.79, 29.65, 29.64, 29.56, 29.51, 29.4, 29.31, 29.29, 29.2 (C-52 to C-57, C-67 to C-76 Diast A and B), 28.9 (C-59 Diast. A and B), 28.62 (C-60 Diast. A and B), 28.57 (C-49 Diast. A), 28.54 (C-49 Diast. B), 28.21 (C-50 Diast. A), 28.20 (C-50 Diast. B), 28.17 (C-51 Diast. A and B), 24.97 (C-66 Diast. A), 24.95 (C-66 Diast. B), 24.03 (C-58 Diast. A), 24.00 (C-58 Diast. B), 23.9 (C-59 Diast. A and B), 23.4 (C-25 or C-26 Diast. A and B), 22.8 (C-77 or C-78 Diast. A and B), 18.5 (C-61 Diast A and B), 14.3 (C-79 Diast. A and B); HRMS *m/z* (ESI$^+$) found 1411.7101 $[M+H]^+$ ($C_{77}H_{109}O_{18}N_2P_2$ requires 1411.7145 $[M+H]^+$). NP-HPLC t_R = 11.28 min (Diast. A), 12.20 (Diast B.), 99.55% @ 280 nm, 98.74% @ 254 nm.

Ammonium (1*S*,2*R*,3*R*,4*R*,5*R*,6*R*)-3-((((*R*)-2-((3-(3-(dodec-11-yn-1-yl)-3*H*-diazirin-3-yl)propanoyl)oxy)-3-(palmitoyloxy)propoxy)oxidophosphoryl)oxy)-2,4,5,6-tetrahydroxycyclohexyl phosphate, PI(5)P-DIAZ, 9

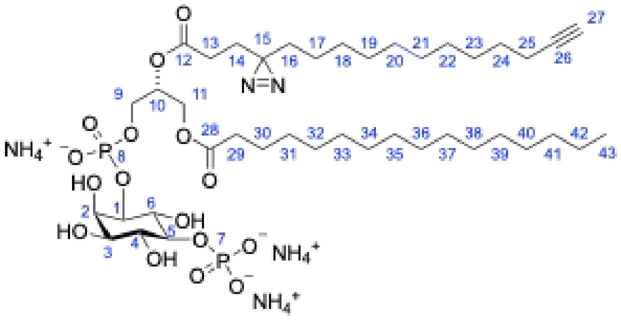

To a solution of protected PI(5)P **(+)-35** (15 mg, 0.011 mmol, 1.0 eq) in anhydrous toluene (1 mL) was added TMSBr (44 µL, 0.33 mmol, 30 eq). The solution was stirred at RT for 2 h after which TLC confirmed complete

consumption of the starting material. The reaction mixture was cooled to 0 °C and concentrated *in vacuo*. The resulting residue was redissolved in anhydrous toluene (1 mL) and concentrated again *in vacuo*. This was repeated three times to remove residual TMSBr, being careful to keep the flask under inert and dry conditions. The residue was then dissolved in MeOH (1 mL), stirred at 0 °C for 1 h and was then concentrated *in vacuo* (2–3 mbar) at 0 °C to avoid bumping. The residue obtained was dissolved in the minimal amount of MeOH:CH_2Cl_2 (1:1 *v/v*) and purified by column chromatography over silica gel ($CHCl_3$:MeOH:2.2 M NH_4OH, 9:7:2). The combined fractions were partially concentrated *in vacuo* at 0 °C, then milli Q H_2O (30 mL) was added. Lyophilization yielded PI(5)P DIAZ **9** (6 mg, 56%) as a colorless, fluffy powder. The compound was stored at −80 °C as the assumed *tris*-ammonium salt: R_f 0.53 ($CHCl_3$:MeOH:2.2 M NH_4OH, 9:7:2); ν_{max} (solid)/cm^{-1} 3317, 2918, 2850, 1742, 1727, 1199, 1048, 947, 727; ^1H NMR (600 MHz, MeOD:$CDCl_3$:D_2O, 4:3:1) δ_H 5.28–5.22 (1 H, m, H-10), 4.42–4.40 (1 H, m, H-11' by HSQC), 4.20 (1 H, dd, *J* 12.3, 7.4, H-11''), 4.17 (1 H, dd, *J* 2.7, 2.7, H-2), 4.07–4.00 (2 H, m, H-9), 3.98–3.93 (1 H, m, H-1), 3.90–3.81 (2 H, m, H-5, H-6), 3.78 (1 H, dd, *J* 9.3, 9.3 H-4), 3.50 (1 H, dd, *J* 9.7, 2.8, H-3), 2.33 (2 H, t, *J* 7.6, H-29), 2.23–2.09 (4 H, m, H-13, H-25), 2.05 (1 H, t, *J* 2.6, H-27), 1.80–1.73 (1 H, m, H-14'), 1.71–1.64 (1 H, m, H-14''), 1.62–1.55 (2 H, m, H-30), 1.50 (2 H, p, *J* 7.2, H-24), 1.42–1.35 (4 H, m, H-21, H-23), 1.35–1.18 (34 H, m, H-16 to H-20, H-31 to H-42), 1.09–1.01 (2 H, m, H-22), 0.87 (3 H, t, *J* 7.0, H-43); ^{31}P NMR (243 MHz, MeOD:$CDCl_3$:D_2O, 4:3:1) δ_P 1.6 (P-7), –0.2 (P-8); ^{13}C NMR (151 MHz, MeOD:$CDCl_3$:D_2O, 4:3:1) δ_C 175.3 (C-28), 173.3 (C-12), 85.4 (C-26), 80.2 (d, *J* 5.4, C-5), 77.0 (d, *J* 6.0, C-1), 72.8 (d, *J* 2.2, C-4), 72.0 (C-2), 71.92 (C-6), 71.87 (C-10), 71.7 (C-3), 69.0 (C-27), 64.4 (d, *J* 4.4, C-9), 63.6 (C-11), 34.8 (C-29), 33.3 (C-23), 32.6, 30.37, 30.32, 30.2, 30.13, 30.10, 30.0, 29.89, 29.88, 29.76, 29.4 (C-16 to C-21, C-31 to C-41), 29.2 (C-13, C-24), 28.9 (C-15), 28.8 (C-14), 25.6 (C-30), 24.5 (C-22), 23.3 (C-42), 18.9 (C-25), 14.5 (C-43); LRMS *m/z* (ESI⁻) 911.3 $[M-H]^-$, 455.4 $[M-H]^-$; HRMS *m/z* (ESI⁻) found 911.4425 $[M-H]^-$ ($C_{41}H_{73}O_{16}N_2P_2$ requires 911.441 $[M-H]^-$).

*[α]$_D$ measurements could not be reliably obtained due to micelle formation.

4,6-Di-*O*-benzyl-*myo*-inositol 1,3,5-orthoformate S1

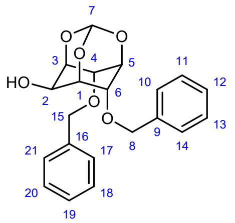

LiH (998 mg, 126 mmol, 4.0 eq) was gradually added to a solution of *myo*-Inositol-1,3,5-orthoformate **11** (6.00 g, 31.5 mmol, 1.0 eq) in anhydrous DMF (90 mL), and the resulting solution was stirred at RT for 30 min. Benzyl bromide (8.58 mL, 72.2 mmol, 2.3 eq) was then added dropwise and the solution was stirred for a further 72 h. The reaction was quenched with H_2O (90 mL), and the volatile components were removed *in vacuo.* Residual DMF was removed using toluene as an azeotrope. The resultant yellow paste was dissolved in EtOAc (250 mL) and washed with H_2O (2 × 100 mL). The organic layer was dried over Na_2SO_4, filtered, and concentrated *in vacuo*. The remaining residue was dissolved in the minimum volume of hot EtOAc and then cooled to –30 °C to yield 4,6-di-*O*-benzyl-*myo*-inositol 1,3,5-orthoformate **S1** (7.29 g, 63%) as a colorless solid: R_f 0.48 (petroleum ether:EtOAc, 1:1); m.p. 117–119 °C (EtOAc) [lit.[5] 123–124 °C (EtOAc)]; ^{1}H NMR (400 MHz; $CDCl_3$) δ_H 7.31–7.24 (10H, m, H-10 to H-14 and H-17 to H-21), 5.47 (1H, d, *J* 1.0, H-7), 4.67 (2H, d, *J* 11.6, H-8 and H-15), 4.59 (2H, d, *J* 11.6, H-8’ and H-15’), 4.48–4.45 (1H, m, H-2), 4.37 (2H, dd, *J* 3.8, 3.8, H-4 and H-6), 4.25–4.18 (3H, m, H-1, H-3 and H-5), 3.00 (1H, d, *J* 11.6, C(2)O*H*); LRMS *m/z* (ESI$^+$) 370.75 ([M+H]$^+$ 100%). These data are in good agreement with the literature values.[5,11]

4,6-Di-*O*-benzyl-*myo*-inositol S2

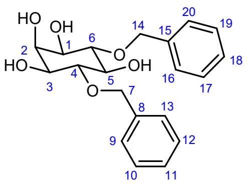

The protected orthoformate **S1** (3.00 g, 8.10 mmol, 1.0 eq) was dissolved in MeOH (8.1 mL) and PTSA·H_2O (1.54 g, 8.10 mmol, 1.0 eq) was added. The solution was heated to 50 °C and stirred for 50 min. The reaction was then

quenched with Et_3N (1.12 mL, 8.10 mmol, 1.0 eq) and then concentrated *in vacuo*. The residue was purified by column chromatography over silica gel (EtOAc) to yield 4,6-di-*O*-benzyl-*myo*-inositol **S2** as a colorless, fluffy solid (2.82 g, 97%): R_f 0.40 (EtOAc); m.p. 130–132°C (EtOAc), [lit.[12] 138.5–139 °C (hexane:CH_2Cl_2)]; ^1H NMR (400 MHz; $CDCl_3$) δ_H 7.41–7.29 (10H, m, H-9 to H-13 and H-16 to H-20), 4.91 (2H, d, *J* 11.4, H-7 and H-14), 4.85 (2H, d, *J* 11.4, H-7' and H-14'), 4.15 (1H, dd, *J* 2.9, 2.9, H-5), 3.67 (2H, dd, *J* 9.3, 9.3, H-4 and H-6), 3.59–3.52 (3H, m, H-1, H-2 and H-3), 2.20 (4H, br s, C(1)O*H*, C(2)O*H*, C(3)O*H* and C(5)O*H*); LRMS *m/z* (ESI$^+$) 383.0 ([M+Na]$^+$, 100%). These data are in good agreement with the literature values.[5]

(−)-1D-1-*O*-Acetyl-4,6-di-*O*-benzyl-*myo*-inositol (−)-S3

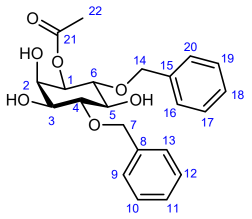

The immobilized Lipozyme® TL-IM (3.00 g) was added to a solution of 4,6-di-*O*-benzyl-*myo*-inositol **S2** (1.00 g, 2.77 mmol, 1.0 eq) in vinyl acetate (250 mL) and hexane (250 mL). After stirring at 45 °C for 18 h the reaction mixture was filtered through a pad of Celite®, washed with hexane (3 × 20 mL) and the combined filtrates were concentrated *in vacuo* to give (**−**)-**S3** (1.06 g, 95%, >99% *e.e.* (other enantiomer not observed) as a colorless solid. The product was deemed to be pure enough, requiring no further purification: R_f 0.78 (EtOAc); $[\alpha]_D^{25}$ −34.4 (*c* 1.0, MeOH) [lit.[5] $[\alpha]_D^{25}$ −39.3 (*c* 1.0, MeOH); m.p. 92–95°C (EtOAc) [lit. 96–97 °C (EtOAc)]; ^1H NMR (400 MHz; $CDCl_3$) δ_H 7.40–7.27 (10H, m, H-16 to H-20 and H-9 to H-13), 4.98 (1H, d, *J* 11.4, H-7), 4.86 (1H, dd, *J* 10.2, 2.8, H-1), 4.80 (1H, d, *J* 11.6, H-14), 4.75 (1H, d, *J* 11.6, H-7'), 4.74 (1H, d, *J* 11.6, H-14'), 4.19 (1H, dd, *J* 2.8, 2.8, H-2), 3.92 (1H, dd, *J* 10.2, 9.1, H-6), 3.70 (1H, dd, *J* 9.3, 9.3, H-4), 3.65–3.55 (2H, m, H-5 and H-3), 2.64 (4H, br s, C(5)OH, C(3)OH, C(2)OH), 2.08 (3H, s, H-22); *m/z* LRMS (ESI$^+$) found 425.2 ([M+Na]$^+$, 100%). These data are in good agreement with the literature values.[5]

(−)-1D-1-O-Acetyl-2,3-O-cyclopentylidene-4,6-di-*O*-benzyl-*myo*-inositol (−)-S4

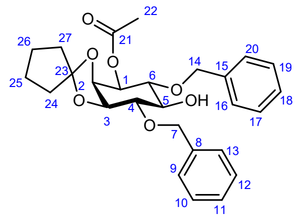

To a clear solution of (−)-1D-1-*O*-acetyl-4,6-di-*O*-benzyl-*myo*-inositol **S3** (800 mg, 1.99 mmol, 1.0 eq) and 1,1-dimethoxycyclopentane (4.55 mL, 33.2 mmol, 17 eq) in CH_2Cl_2 (5.0 mL) was added PTSA·H_2O (30 mg, 0.16 mmol, 0.080 eq). The resulting solution was stirred at RT for 18 h. The solution was then quenched with Et_3N (0.020 mL, 0.16 mmol, 0.08 eq) and concentrated *in vacuo*. The residue obtained was purified by column chromatography over silica gel (petroleum ether:EtOAc, 9:1 to 5:5) to give **(−)-S4** (899 mg, 96%) as a pale yellow oil: R_f 0.59 (petroleum ether:EtOAc, 8:2); $[\alpha]_D^{25}$ −30.1 (*c* 1.0, $CHCl_3$), [lit.[5] $[\alpha]_D^{25}$ 23.2 (*c* 1.0, $CHCl_3$)]; ^{1}H NMR (400 MHz; $CDCl_3$) δ_H 7.41–7.25 (10H, m, H-9 to H-13 and H-16 to H-20), 5.18 (1H, dd, *J* 8.4, 3.7, H-1), 4.92 (1H, d, *J* 11.6, H-7), 4.82 (1H, d, *J* 11.6, H-14), 4.75 (1H, d, *J* 11.6, H-14'), 4.71 (1H, d, *J* 11.6, H-7'), 4.33 (1H, dd, *J* 5.6, 3.7, H-2), 4.19–4.15 (1H, m, H-3), 3.84–3.76 (1H, m, H-6), 3.68–3.59 (2H, m, H-5 and H-4), 2.65 (1H, s, C(5)O*H*), 2.08 (3H, s, H-22), 1.99–1.83 (2H, m, H-24', H-27'), 1.79–1.62 (6H, m, H-25, H-26 and H-24'' and H-27''). These data are in good agreement with the literature values.[5]

(−)-1D-1-*O*-Acetate-2,3-*O*-cyclopentylidene-4,6-di-*O*-benzyl-5-*O*-(2-oxo-5.6-benzo-1,3,2-dioxaphosphep-2-yl)-*myo*-inositol (−)-S5

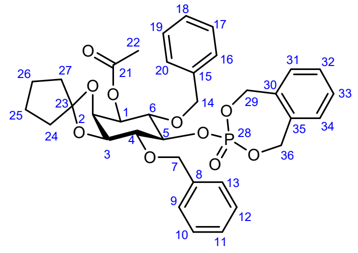

To a solution of alcohol **(−)-S4** (400 mg, 0.854 mmol, 1.0 eq) and *N*,*N*-diethyl-1,5-dihydrobenzo[*e*][1,3,2]dioxaphosphepin-3-amine **17** (613 mg, 2.56 mmol, 3.0 eq) in CH_2Cl_2 (15 mL) was added 1*H*-tetrazole (5.70 mL, 2.56 mmol, 3.0 eq, 0.45 M in MeCN). After stirring at RT for 1 h, the cloudy reaction mixture was cooled to −78 °C and *m*CPBA (574 mg, 2.56 mmol, 3.0 eq, 77%) was added in a single portion. The reaction mixture was gradually warmed to RT over 20 min, stirred for 1 h and then finally quenched with

an aqueous solution of $Na_2S_2O_3$ (10% *w/v*, 10 mL). The aqueous layer was extracted with CH_2Cl_2 (4 × 10 mL) and the combined organic layers were washed with an aqueous solution of $Na_2S_2O_3$ (10% *w/v*, 3 × 10 mL), brine (30 mL) and then dried over Na_2SO_4, filtered, and concentrated *in vacuo*. Purification by column chromatography over silica gel (petroleum ether:EtOAc, 9:1 to 5:5) afforded **(−)-S5** (512 mg, 93%) as an amorphous, colorless foam: R_f 0.13 (petroleum ether:Et_2O, 4:6); $[\alpha]_D^{25}$ −5.9 (*c* 1.0, $CHCl_3$), [lit.[5] $[\alpha]_D^{25}$ −8.1 (*c* 1.0, $CHCl_3$)]; ^1H NMR (400 MHz; $CDCl_3$) δ_H 7.44–7.39 (2H, m, H-9 and H-13), 7.37–7.24 (10H, m, H-10 to H-12, H-16 to H-20, H-32 and H-33), 7.20–7.13 (2H, m, H-31 and H-34), 5.25 (1H, dd, *J* 8.7, 3.6, H-1), 5.22–4.93 (4H, m, H-29 and H-36), 4.86 (1H, d *J* 11.3, H-14), 4.85 (1H, d *J* 11.6, H-7), 4.76 (1H, dd *J* 11.6, H-7'), 4.69 (1H, dd, *J* 11.3, H-14'), 4.67–4.60 (1H, m, H-5), 4.39 (1H, dd, *J* 6.2, 3.6, H-2), 4.23 (1H, dd, *J* 6.2, 6.2, H-3), 4.04 (1H, dd, *J* 8.7, 7.0, H-6), 3.89 (1H, dd, *J* 7.9, 6.0, H-4), 2.05 (3H, s, H-22), 1.99–1.83 (2H, m, H-24 and H-27), 1.75–1.61 (6H, m, H-24', H-25, H-26 and H-27'); ^{31}P NMR (162 MHz, $CDCl_3$) δ_P −0.24; LRMS *m/z* (ESI$^+$) 650.7 ([M+H]$^+$, 100%). These data are in good agreement with the literature values.[5]

(+)-1D-2,3-*O*-Cyclopentylidene-4,6-di-*O*-benzyl-5-*O*-(2-oxo-5.6-benzo-1,3,2-dioxaphosphep-2-yl)-*myo*-inositol (+)-S6

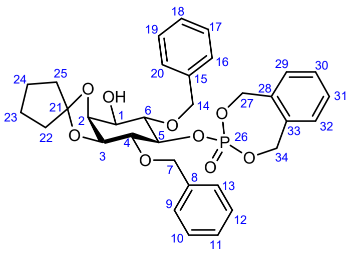

To a solution of acetate **(−)-S5** (660 mg, 1.08 mmol, 1.0 eq) in MeOH (92 mL) was added ground K_2CO_3 (298 mg, 2.16 mmol, 2.0 eq). The reaction mixture was stirred at RT for 1 h. The solvent was then partially removed *in vacuo* at RT and H_2O (30 mL) was added to the resulting residue. The aqueous layer was extracted with EtOAc (4 × 10 mL) and the combined organic layers were washed with brine (40 mL), and then dried over Na_2SO_4, filtered, and concentrated *in vacuo*. Purification using column chromatography over silica gel (petroleum ether:EtOAc, 9:1 to 5:5) afforded alcohol **(+)-S6** (474 mg, 72%) as an amorphous

solid: R_f 0.59 (petroleum ether:EtOAc, 2:8); $[\alpha]_D^{25}$ +1.6 (*c* 1.0, $CHCl_3$), [lit.[5] $[\alpha]_D^{25}$ +3.1 (*c* 2.0, $CHCl_3$)]; ^{1}H NMR (400 MHz, CD_2Cl_2) δ_H 7.62–7.00 (14H, m, H-9 to H-13, H-16 to H-20, H-29 to H-32), 5.18–5.07 (3H, m, H-27 or H-34, H-27' and H-34'), 5.00 (1H, dd, *J* 17.8, 13.7, H-27 or H-34), 4.88 (1H, d, *J* 11.1, H-7 or H-14), 4.87 (1H, d, *J* 11.4, H-7 or H-14), 4.73 (2H, d, *J* 11.4, H-7' or H-14'), 4.60–4.50 (1H, m, H-5), 4.33 (1H, dd, *J* 6.5, 3.7, H-2), 4.23 (1H, dd, *J* 6.5, 6.5, H-3), 4.04–3.89 (3H, m, H-1, H-5, H-6), 2.55 (1H, d, *J* 4.1, C(1)OH), 2.00–1.91 (2H, m, H-22 or H-25), 1.82–1.63 (6H, m, H-23, H-24 and H-22 or H-25). ^{31}P NMR (162 MHz, CD_2Cl_2) δ_P −1.0; LRMS *m/z* (ESI$^+$) 609.285 ([M+H]$^+$, 100%). These data are in good agreement with the literature values.[5]

(−)-[(4*S*)-2,2-Dimethyl-1,3-dioxolan-4-yl]methyl octanoate (−)-S7

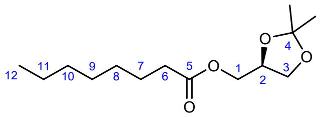

N,*N'*-Dicyclohexylcarbodiimide (6.14 g, 30.3 mmol, 2.0 eq) was added to a stirred solution of (*R*)-(−)-2,3-isopropylidene-*sn*-glycerol (−)-**28** (2.00 g, 15.1 mmol, 1.0 eq), octanoic acid (3.09 mL, 15.1 mmol, 1.0 eq) and 4-DMAP (0.11 g, 0.91 mmol, 0.06 eq) in CH_2Cl_2 (45 mL), and was stirred for 16 h. The mixture was then diluted with hexane (20 mL), filtered through Celite® and washed with hexane (3 × 10 mL). The combined filtrates were concentrated *in vacuo* and the residue obtained was purified by column chromatography over silica gel (petroleum ether:EtOAc, 20:1) to yield **(−)-S7** (3.55 g, 91%) as a colorless oil: R_f 0.18 (petroleum ether:EtOAc, 20:1); $[\alpha]_D^{25}$ **−**12.0 (*c* 1.0, hexane); ν_{max} (thin film)/cm^{-1} 2987, 2956, 2930, 2858, 1741, 1457, 1371, 1255, 1215, 1161, 1087, 1057, 843; ^{1}H NMR (400 MHz, $CDCl_3$) δ_H 4.36–4.25 (1H, m, H-2), 4.16 (1H, dd, *J* 11.5, 4.7, H-1), 4.13–4.03 (2H, m, H-1' and H-3), 3.74 (1H, dd, *J* 8.4, 6.2, H-3'), 2.34 (2H, t, *J* 7.6, H-6), 1.68–1.57 (2H, m, H-7), 1.43 (3H, s, C(4)CH_3), 1.37 (3H, s, C(4)CH_3), 1.34–1.24 (8H, m, H-8 to H-11), 0.90–0.85 (3H, m, H-12). ^{13}C NMR (101 MHz, $CDCl_3$) δ_C 173.8 (C-5), 110.0 (C-4), 73.8 (C-2), 66.5 (C-3), 64.7 (C-1), 34.3 (C-6), 31.8 (C-10), 29.2, 29.0 (C-8 and C-9), 26.8 (C(4)-CH_3), 25.6 (C(4)-CH_3), 25.0 (C-7), 22.7 (C-11), 14.2 (C-12); LRMS *m/z* (ESI$^+$) 281.198 ([M+Na]$^+$, 100%); HRMS *m/z* (ESI$^+$) found

281.1724 [M+Na]$^+$ ($C_{14}H_{26}O_4Na$ requires 281.1723 [M+Na]$^+$). These data are in good agreement with the literature values.

(+)-(2*S*)-2,3-Dihydroxypropyl hexadecanoate (+)-S8

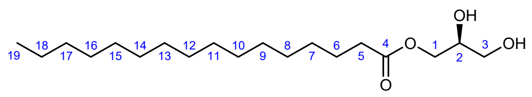

To acetonide **(−)-29** (3.00 g, 8.10 mmol, 1.0 eq), was added 30 mL of a mixture of acetic acid and distilled H_2O (4:1). The reaction mixture was stirred and heated to 50 °C for 2 h. Following complete consumption of the starting material as observed *via* TLC, the reaction mixture was transferred to a conical flask and diluted with distilled H_2O (100 mL). Any solid formed was dissolved *via* the addition of CH_2Cl_2 and vigorous swirling. Solid K_2CO_3 was then slowly added to the resultant biphasic solution with swirling until effervescence ceased. The aqueous layer was then extracted with CH_2Cl_2 (3 × 150 mL) and the combined organic extracts were washed with brine (150 mL), dried over Na_2SO_4, and concentrated *in vacuo*. The resultant colorless solid was then subject to crystallization from EtOAc and heptane to give 2,3-dihydroxypropyl hexadecanoate **(+)-S8** as white shiny crystals (2.43 g, 91%): R_f 0.31 (petroleum ether:EtOAc, 1:1) m.p. 59–63 °C (hexane:EtOAc), [Lit.[9] 68.5–69.5 °C (pentane:EtOAc)]; $[\alpha]_D^{25}$ +2.8 (*c* 3.4, pyridine) [lit.[5] $[\alpha]_D^{25}$ +3.9 (c 2.9, pyridine)], [lit.[9] $[\alpha]_D^{23}$ +4.14 (c 2.53, pyridine)]; δ_H (400 MHz, $CDCl_3$) 4.21 (1H, dd, *J* 11.6, 4.6, H-1), 4.15 (1H, dd, *J* 11.7, 6.1, H-1’), 3.98–3.89 (1H, m, H-2), 3.75–3.66 (1H, m, H-3), 3.60 (1H, dd, *J* 11.4, 5.7, H-3’), 2.49–2.42 (1H, br s, C(2)OH), 2.35 (2H, t, *J* 7.6, H-5), 2.01 (1H, br s, C(3)OH), 1.68–1.58 (2H, m, H-6), 1.36–1.22 (24H, m, H-7 to H-18) 0.91–0.84 (3H, t, *J* 6.7, H-19); LRMS *m/z* (ESI$^+$) 353.309 ([M+Na]$^+$, 100%); These data are in good agreement with the literature values.[9]

(+)-(2*S*)-2,3-Dihydroxypropyl octanoate (+)-S9

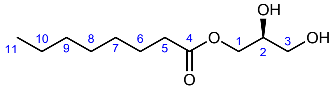

To acetonide **(−)-S7** (3.00 g, 11.6 mmol, 1.0 eq), was added 30 mL of a mixture of acetic acid and distilled H_2O (4:1). The reaction mixture was stirred and heated to 50 °C for 2 h. Following complete consumption of the starting material as observed *via* TLC, the reaction mixture was transferred to a conical flask and diluted with distilled H_2O (100 mL). Any solid formed was dissolved *via* the addition of CH_2Cl_2 and vigorous swirling. Solid K_2CO_3 was then slowly added to the resultant biphasic solution with swirling until effervescence ceased. The aqueous layer was then extracted with CH_2Cl_2 (3 × 150 mL) and the combined organic extracts were washed with brine (150 mL), dried over Na_2SO_4, and concentrated *in vacuo* to give diol **(+)-S9** (2.51 g, 99%) as a colorless oil with no need for further purification: R_f 0.25 (petroleum ether:EtOAc, 1:1); $[\alpha]_D^{25}$ +0.45 (*c* 3.1, pyridine); $\boldsymbol{\nu}_{max}$ (thin film)/cm^{-1} 2956, 2926, 2857, 1737, 1169, 1109, 1049; ^{1}H NMR (400 MHz, $CDCl_3$) δ_H 4.20 (1H, dd, *J* 11.7, 4.7, H-1), 4.15 (1H, dd, *J* 11.7, 6.1, H-1’), 3.97–3.89 (1H, m, H-2), 3.70 (1H, dd, *J* 11.5, 4.0, H-3), 3.60 (1H, dd, *J* 11.5, 5.8, H-3’), 2.57 (1H, s, C(2)OH), 2.35 (2H, t, *J* 7.6, H-5), 2.14 (1H, s, C(3)OH), 1.69–1.57 (2H, m, H-6), 1.36–1.22 (8H, m, H-7 to H-10), 0.92–0.84 (3H, m, H-11); ^{13}C NMR (101 MHz, $CDCl_3$) δ_C 174.5 (C-4), 70.4 (C-2), 65.3 (C-1), 63.5 (C-3), 34.3 (C-5), 31.8, 29.2, 29.0, 25.1, 22.7 (C-6 to C-10), 14.2 (C-11); LRMS *m/z* (ESI$^+$) 241.0 ([M+Na]$^+$, 100%); HRMS *m/z* (ESI$^+$) found 241.1411 [M+Na]$^+$ ($C_{11}H_{22}O_4Na$ requires 241.1410 [M+Na]$^+$).

(+)-(2*S*)-3-[Bis(4-methoxyphenyl)(phenyl)methoxy]-2-hydroxypropyl hexadecanoate (+)-S10

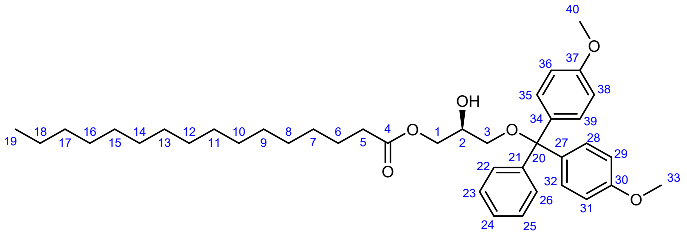

To a solution of diol **(+)-S8** (1.50 g, 4.54 mmol, 1.0 eq) in anhydrous pyridine (45 mL) was added 4,4′-dimethoxytrityl chloride (1.85 g, 5.45 mmol, 1.2 eq) in one portion. After 50 min stirring at RT, the yellow solution was concentrated *in vacuo* at RT. The

residue obtained was purified by column chromatography over silica gel (petroleum ether:EtOAc, 95:5 to 80:20) to give **(+)-S10** (2.24 g, 78%) as a green-tinted oil: R_f 0.54 (petroleum ether:EtOAc, 8:2); $[\alpha]_D^{25}$ +2.4 (*c* 1.0, $CHCl_3$); $\boldsymbol{\nu}_{max}$ (thin film)/cm^{-1} 2924, 2853, 1737, 1608, 1509, 1251, 1177, 1036, 829; ^{1}H NMR(400 MHz, $CDCl_3$) δ_H 7.44–7.39 (2H, m, H-22 and H-26), 7.34–7.26 (6H, m, H-23, H-25, H-H-28, H-32, H-35, H-39), 7.25–7.16 (1H, m, H-24), 6.86–6.80 (4H, m, H-29, H-31, H-36, H-38), 4.21 (1H, dd, *J* 11.5, 4.4, H-1), 4.16 (1H, dd, *J* 11.5, 6.1, H-1'), 4.03–3.94 (1H, m, H-2), 3.79 (6H, s, H-33 and H-40), 3.25–3.17 (2H, m, H-3), 2.40 (1H, d, *J* 5.2, C(2)OH), 2.29 (2H, t, *J* 7.6, H-5), 1.63–1.53 (2H, m, H-6), 1.35–1.21 (24H, m, H-7 to H-18), 0.92–0.84 (3H, m, H-19). ^{13}C NMR (101 MHz, $CDCl_3$) δ_C 174.1 (C-4), 158.7 (C-30 and C-37), 144.7 (C-21), 135.9 (C-27 and C-34), 130.2 (C-28, C-32, C-35 and C-39), 128.2 (C-22 and C-26), 128.0 (C-23 and C-25), 127.0 (C-24), 113.3 (C-29, C-31, C-36 and C-38), 86.4 (C-20), 69.5 (C-2), 65.9 (C-1), 64.2 (C-3), 55.4 (C-33 and C-40), 34.3 (C-5), 32.1 (C-17), 29.84, 29.80, 29.79, 29.6, 29.5, 29.4, 29.3 (C-7 to C-16), 25.0 (C-6), 22.8 (C-18), 14.3 (C-19); LRMS *m/z* (ESI$^+$) 655.473 ([M+Na]$^+$, 100%); HRMS *m/z* (ESI$^+$) found 655.3965 [M+Na]$^+$ ($C_{40}H_{56}O_6Na$ requires 655.3969 [M+Na]$^+$).

(+)-(2*S*)-3-[Bis(4-methoxyphenyl)(phenyl)methoxy]-2-hydroxypropyl octanoate (+)-S11

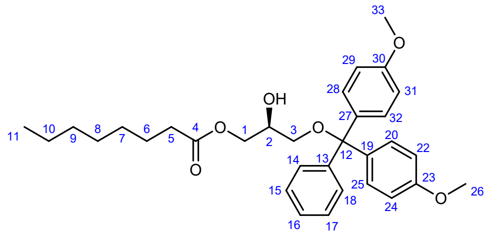

To a solution of diol (+)-**S9** (1.50 g, 6.87 mmol, 1.0 eq) in anhydrous pyridine (69 mL) was added 4,4′-dimethoxytrityl chloride (2.79 g, 8.24 mmol, 1.2 eq) in one portion. After 50 min stirring at RT, the yellow solution was concentrated *in vacuo* at RT. The residue obtained was purified by column chromatography over silica gel (petroleum ether:EtOAc, 10:0 to 8:2) to give alcohol **(+)-S11** (2.83 g, 79%) as a green-tinted oil: R_f 0.54 (petroleum ether:EtOAc, 8:2); $[\alpha]_D^{25}$ +3.1 (*c* 1.0, $CHCl_3$); $\boldsymbol{\nu}_{max}$ (thin film)/cm^{-1} 2929, 2857, 1736, 1608, 1509, 1250, 1176, 1035, 829; ^{1}H NMR (400 MHz, $CDCl_3$) δ_H 7.44–7.39 (2H, m, H-14, H-16), 7.34–7.26 (6H, m, H15, H-17, H-20, H-25, H-28, H-32), 7.24–7.19

(1H, m, H-16), 6.86–6.80 (4H, m, H-22, H-24, H-29, H-31), 4.21 (1H, dd, *J* 11.5, 4.4, H-1), 4.16 (1H, dd, *J* 12.1, 5.6, H-1'), 4.02–3.94 (1H, m, H-2), 3.79 (6H, s, H-26, H-33), 3.22 (1H, dd, *J* 9.6, 5.0, H-3), 3.19 (1H, dd, *J* 9.6, 5.6, H-3'), 2.42 (1H, d, *J* 5.2, C(2)OH), 2.29 (2H, t, *J* 7.6, H-5), 1.64–1.53 (2H, m, H-6), 1.35–1.22 (8H, m, H-7 to H-10), 0.88 (3H, t, *J* 6.8, H-11); ^{13}C NMR (101 MHz, $CDCl_3$) δ_C 174.1 (C-4), 158.7 (C-23, C-30), 144.7 (C-13), 135.9 (C-19,C-27), 130.2 (C-20, C-25, C-28, C-32), 128.2 (C-14, C-18), 128.0 (C-15, C-17), 127.0 (C-16), 113.3 (C-22, C-24, C-29, C-31), 86.4 (C-12), 69.5 (C-2), 65.9 (C-1), 64.2 (C-3), 55.4 (C-26, C-33), 34.3 (C-5), 31.8, 29.2, 29.0 (C-7 to C-9), 25.0 (C-6), 22.7 (C-10), 14.2 (C-11); LRMS *m/z* (ESI$^+$) 543.348 ([M+Na]$^+$, 100%); HRMS *m/z* (ESI$^+$) found 543.2714 [M+Na]$^+$ ($C_{32}H_{40}O_6Na$ requires 543.2717 [M+Na]$^+$).

(+)-(2*S*)-3-[Bis(4-methoxyphenyl)(phenyl)methoxy]-2-(octanoyloxy)propyl hexadecanoate (+)-S12

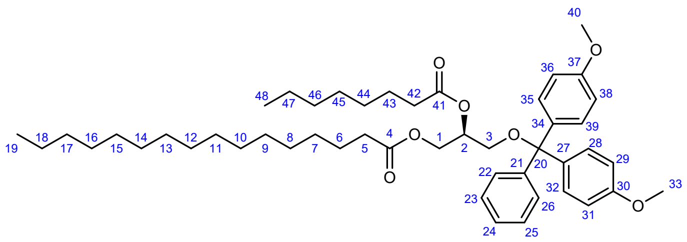

To a solution of alcohol **(+)-S10** (1.00 g, 1.58 mmol, 1.0 eq) in CH_2Cl_2 (20 mL) was added DCC (392 mg, 1.90 mmol, 1.2 eq), 4-DMAP (12 mg, 0.10 mmol, 0.06 eq) and octanoic acid (0.25 mL, 1.6 mmol, 1.0 eq). The solution was stirred at RT for 18 h, after which the reaction mixture was diluted with hexane (20 mL). The suspension was filtered through celite, and the celite was subsequently washed with hexane (3 × 10 mL). The combined filtrates were concentrated *in vacuo* at RT. The resulting residue was purified by column chromatography over silica gel (petroleum ether:EtOAc, 100:0, to 92:8) which yielded **(+)-S12** (1.01 g, 84%) as a colorless oil: R_f 0.50 (petroleum ether:EtOAc 9:1); $[\alpha]_D^{25}$ +14.6 (*c* 1.0, $CHCl_3$); ν_{max} (thin film)/cm^{-1} 2925, 2854, 1742, 1608, 1509, 1251, 1176, 1037, 829; ^{1}H NMR(400 MHz, $CDCl_3$) δ_H 7.45–7.39 (2H, m, H-22 and H-26), 7.34–7.24 (6H, m, H-23, H-25, H-28, H-32, H-35, H-39), 7.24–7.18 (1H, m, H-24), 6.86–6.79 (4H, m, H-29, H-31, H-36, H-38), 5.30–5.22 (1H, m, H-2), 4.34 (1H, dd, *J*

11.9, 3.7, H-1), 4.23 (1H, dd, *J* 11.9, 6.7, H-1’), 3.79 (6H, s, H-33, H-40), 3.23 (1H, d, *J* 1.6, H-3), 3.22 (1H, d, *J* 1.9, H-3’), 2.33 (2H, td, *J* 7.4, 1.0, H-5), 2.24 (2H, td, *J* 8.0, 7.1, H-42), 1.69–1.49 (4H, m, H-6, H-43), 1.39–1.20 (32H, m, H-7 to H-18 and H-44 to H-47), 0.89 (2H, t, H-48), 0.88 (3H, t, H-19). ^{13}C NMR (101 MHz, $CDCl_3$) δ_C 173.5, 173.2 (C-4, C-41), 158.7 (C-30 and C-37), 144.7 (C-21), 135.89, 135.86 (C-27, C-34), 130.14, 130.11 (C-28, C-32, C-35, C-39), 128.22, 127.94 (C-22, C-23, C-25, C-26), 127.0 (C-24), 113.3 (C-29, C-31, C-35, C-39), 86.2 (C-20), 70.6 (C-2), 63.1 (C-1), 62.2 (C-3), 55.3 (C-33, C-40), 34.6, 34.3 (C-5, C-42), 32.1, 31.8 (C-17, C-46), 29.83, 29.79, 29.62, 29.49, 29.47, 29.3, 29.2, 29.1 (C-7 to C-16 and C-44 and C-45), 25.1, 25.0 (C-6, C-43), 22.8, 22.8, (C-18, C-47), 14.3, 14.2 (C-19, C-48); LRMS *m/z* (ESI$^+$) 781.591 ([M+Na]$^+$, 100%); HRMS *m/z* (ESI$^+$) found 781.5010 [M+Na]$^+$ ($C_{48}H_{70}O_7Na$ requires 781.5014 [M+Na]$^+$).

(+)-(2*S*)-1-[Bis(4-methoxyphenyl)(phenyl)methoxy]-3-(octanoyloxy)propan-2-yl hexadecanoate (+)-S13

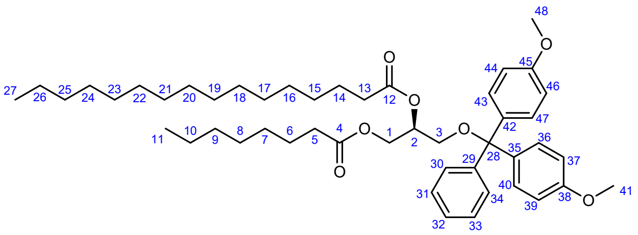

To a solution of alcohol **(+)-S11** (1.00 g, 1.92 mmol, 1.0 eq) in CH_2Cl_2 (20 mL) was added DCC (475 mg, 2.30 mmol, 1.2 eq), 4-DMAP (14 mg, 0.12 mmol, 0.06 eq) and palmitic acid (492 mg, 1.92 mmol, 1.0 eq). The solution was stirred at RT for 18 h, after which the reaction mixture was diluted with hexane (20 mL). The suspension was filtered through celite, and the celite subsequently washed with hexane (3 × 10 mL). The combined filtrates were concentrated *in vacuo* at RT. The resulting residue was purified by column chromatography over silica gel (petroleum ether:EtOAc, 10:0 to 9:1) which yielded **(+)-S13** (1.00 g, 69%) as a colorless oil: R_f 0.50 (petroleum ether:EtOAc, 9:1); $[\alpha]_D^{25}$ +12.9 (c 1.0, $CHCl_3$); ν_{max} (thin film)/cm^{-1} 2925, 2854, 1742, 1608, 1509, 1251, 1176, 1037, 829; ^{1}H NMR (400 MHz, $CDCl_3$) δ_H 7.44–7.39 (2H, m, H-30, H-34), 7.29 (6H, m, H-31, H-33, H-36, H-40, H-43, H-47), 7.23–7.17 (1H, m, H-32), 6.85–

6.79 (4H, m, H-37, H-39, H-44, H-46), 5.29–5.22 (1H, m, H-2), 4.34 (1H, dd, *J* 11.8, 3.7, H-1), 4.23 (1H, dd, *J* 11.8, 6.7, H-1'), 3.79 (6H, s, H-41, H-48), 3.24 (1H, dd, *J* 10.0, 4.9, H-3), 3.21 (1H, dd, *J* 10.0, 5.1, H-3'), 2.36–2.30 (2H, m, H-5 or H-13), 2.26–2.21 (2H, m, H-5 or H-13), 1.68–1.50 (4H, m, H-6, H-14), 1.36–1.22 (32H, m, H-7 to H-10, and H-15 to H-26), 0.91–0.86 (6H, m, H-11 and H-27); ^{13}C NMR (101 MHz, $CDCl_3$) δ_C 173.5, 173.2 (C-4, C-12), 158.6 (C-38 and C-35), 144.7 (C-29), 135.89, 135.86 (C-35, C-42), 130.13, 130.12, (C-36, C-40, C,43, C-47), 128.2, 127.9 (C-37, C-39, C-43, C-47), 127.0 (C-32), 113.3 (C-38, C-40, C-45, C-47), 86.2 (C-28), 70.6 (C-2), 63.1 (C-1), 62.2 (C-3), 55.3 (C-41, C-48), 34.6, 34.3 (C-5, C-13), 32.1, 31.8 (C-9, C-25), 29.83, 29.78, 29.6, 29.49, 29.47, 29.3, 29.2, 29.1 (C-7, C-8, C-15 to C-24), 25.1, 25.0 (C-6, C-14), 22.8, 22.7 (C-10, C-26), 14.3, 14.2 (C-11, C-27); LRMS *m/z* (ESI^+) 781.604 ($[M+Na]^+$, 100%); HRMS *m/z* (ESI^+) found 781.5009 $[M+Na]^+$ ($C_{48}H_{70}O_7Na$ requires 781.5014 $[M+Na]^+$).

(–)-(2*S*)-3-Hydroxy-2-(octanoyloxy)propyl hexadecanoate (–)-S14

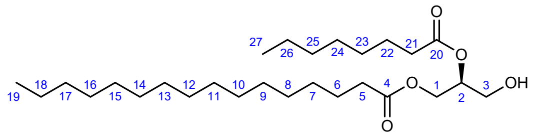

Aqueous acetic acid ($AcOH:H_2O$, 4:1 *v/v*, 5 mL) was added to **(+)-S12** (800 mg, 1.05 mmol, 1.0 eq) and stirred vigorously. CH_2Cl_2 was then added dropwise until the biphasic mixture homogenized and became bright orange. The mixture was then stirred further at 50 °C for 2 h. Complete consumption of the starting material was monitored *via* TLC. The solution was then cooled to RT and concentrated *in vacuo.* The resulting residue was then purified *via* column chromatography over silica gel (petroleum ether:EtOAc, 100:0 to 84:16) yielding alcohol **(−)-S14** (387 mg, 81%, ~94% *e.e.*) which crystalized under a stream of nitrogen from EtOAc to form a colorless solid: R_f 0.34 (petroleum ether:EtOAc, 9:1); $[\alpha]_D^{25}$ –4.5 (*c* 1.0, $CHCl_3$); m.p. 27–28 °C (EtOAc); ν_{max} (thin film)/cm^{-1} 2924, 2854, 1742, 1466, 1164, 1108, 1052, 723; ^1H NMR (400 MHz, $CDCl_3$) δ_H 5.08 (1H, m, H-2), 4.32 (1H, dd, *J* 12.0, 4.5, H-1), 4.24 (1H, dd, *J* 12.0, 5.6, H-1'), 3.75–3.71 (2H, m), 2.35 (2H, t, *J* 7.5, H-5), 2.32 (2H, t, *J* 7.5, H-21), 2.01

(1H, t, *J* 6.5, C(3)OH), 1.68–1.57 (4H, m, H-6, H-22), 1.37–1.20 (32H, m, H-7 to H-18 and H-23 to H-26), 0.92–0.84 (6H, m, H-19, H-27); ^{13}C NMR (101 MHz, $CDCl_3$) δ_C 173.9, 173.6 (C-4, C-20), 72.3 (C-2), 62.1 (C-3), 61.8 (C-1), 34.4, 34.3 (C-5, C-21), 32.1, 31.8 (C-17, C-25), 29.85, 29.81, 29.77, 29.6, 29.5, 29.4, 29.3, 29.2 (C-7 to C-16, C-23, C-24), 25.1, 25.0 (C-6, C-22), 22.8, 22.7 (C-18, C-26), 14.3, 14.2 (C-19, C-27); LRMS *m/z* (ESI$^+$) 479.443 ([M+Na]$^+$, 100%); HRMS *m/z* (ESI$^+$) found 479.3708 [M+Na]$^+$ ($C_{27}H_{52}O_5Na$ requires 479.3707 [M+Na]$^+$); The *e.e.* of **(–)-S14** was calculated by ^{1}H NMR using a chiral auxiliary (see **Figure S1**).

(2*S*)-1-Hydroxy-3-(octanoyloxy)propan-2-yl hexadecanoate (–)-S15

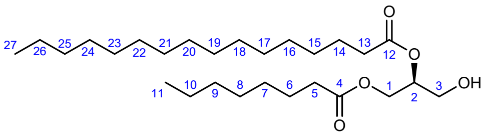

Aqueous acetic acid (AcOH:H_2O, 4:1 *v/v*, 5 mL) was added to **(+)-S13** (800 mg, 1.05 mmol, 1.0 eq) and stirred vigorously. CH_2Cl_2 was then added dropwise until the biphasic mixture homogenized and became bright orange. The mixture was then stirred further at 50 °C for 2 h. Complete consumption of the starting material was monitored *via* TLC. The solution was then cooled to RT and concentrated in *vacuo.* The resulting residue was then purified *via* column chromatography over silica gel (petroleum ether:EtOAc, 100:0, 84:16) yielding alcohol **(–)-S15** (389 mg, 81%, ~90% *e.e.*) which crystalized under a stream of nitrogen from EtOAc to form a colorless solid: R_f 0.37 (petroleum ether:EtOAc, 9:1); $[\alpha]_D^{25}$ –3.8 (*c* 1.0, $CHCl_3$); m.p. 26–28 °C (EtOAc); ν_{max} (thin film)/cm^{-1} 2924, 2854, 1742, 1466, 1164, 1109, 1051, 723; ^{1}H NMR (400 MHz, $CDCl_3$) δ_H 5.11–5.05 (1H, m, H-2), 4.32 (1H, dd, *J* 12.0, 4.5, H-1), 4.24 (1H, dd, *J* 12.0, 5.7, H-1'), 3.75–3.71 (2H, m, H-3), 2.37–2.29 (4H, m, H-5, H-13), 2.02 (1H, t, *J* 6.5, C(3)OH), 1.68–1.57 (4H, m, C-6, C-14), 1.37–1.21 (34H, m, H-7 to H-10 and H-15 to H-26), 0.92–0.85 (6H, m, H-11, H-27); ^{13}C NMR (101 MHz, $CDCl_3$) δ_C 173.9, 173.6 (C-4, C-12), 72.3 (C-2), 62.1 (C-3), 61.7 (C-1), 34.5, 34.3 (C-5, C-13), 32.1, 31.8 (C-9, C-25), 29.84, 29.80, 29.77, 29.6, 29.5, 29.4, 29.24, 29.22, 29.1 (C-7, C-8, C-15 to C-24), 25.1, 25.0 (C-6, C-14), 22.8, 22.7 (C-10, C-26), 14.3, 14.2

(C-11, C-27); LRMS *m/z* (ESI+) 935.83 ([2M+Na]+, 100%); HRMS *m/z* (ESI+) found 479.3706 [M+Na]+ ($C_{27}H_{52}O_5Na$ requires 479.3707 [M+Na]+); The *e.e.* of **(–)-S15** was calculated by ^{1}H NMR using a chiral auxiliary (see **Figure S1**).

(+)-(2*R*)-3-({(Benzyloxy)[di(propan-2-yl)amino]phosphanyl}oxy)-2-(octanoyloxy)propyl hexadecanoate (+)-S16

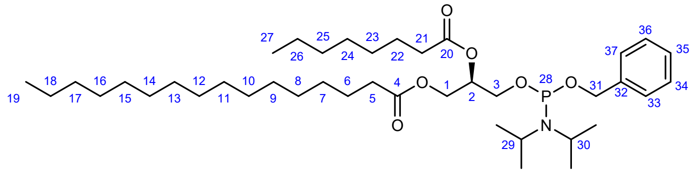

To a solution of benzyloxy-bis(*N*,*N*-diisopropylamino) phosphine **33** (558 mg, 1.65 mmol, 3.0 eq) in anhydrous CH_2Cl_2 (6 mL) was added 1*H*-tetrazole (3.70 mL, 1.65 mmol, 3.0 eq, 0.45 M in MeCN). The solution was stirred for 10 min, after which, alcohol **(−)-S14** (250 mg, 0.547 mmol, 1.0 eq) was added in a single batch. The reaction mixture was stirred at RT for 18 h, then diluted with CH_2Cl_2 (10 mL), and was then quenched by the addition of a saturated aqueous solution of $NaHCO_3$ (10 mL). The organic phase was extracted with CH_2Cl_2 (3 × 10 mL). The combined organic layers were washed with brine (20 mL), dried over $MgSO_4$, filtered, and then concentrated *in vacuo*. The residue obtained was purified by column chromatography over silica gel (hexane:EtOAc:Et_3N, 85:10:5) to give phosphoramidite **(+)-S16** (415 mg, >99%) as a colorless viscous oil, containing a mixture of two diastereoisomers. Due to its unstable nature, this phosphoramidite was synthesized fresh before each use and was used without further characterization: R_f 0.75 (petroleum ether:EtOAc:Et_3N, 85:10:5); $[\alpha]_D^{25}$ +7.9 (*c* 1.0, $CHCl_3$); ν_{max} (thin film)/cm^{-1} 2962, 2925, 2854, 1743, 1457, 976; ^{1}H NMR (400 MHz, $CDCl_3$) δ_H 7.40–7.22 (10H, m, H-33, H-34, H-35, H-36, H-37, Diast. A and B), 5.23–5.14 (2H, m, H-2, Diast. A and B), 4.81–4.61 (4H, m, H-31, Diast. A and B), 4.38–4.30 (2H, m, H-1, Diast. A and B), 4.21–4.13 (2H, m, H-1', Diast. A and B), 3.83–3.58 (8H, m, H-3, H-29, H-30, Diast. A and B), 2.32–2.26 (8H, m, H-5, H-21, Diast. A and B), 1.66–1.56 (8H, m, H-6, H-22, Diast. A and B), 1.34–1.23 (64H, m, H-7 to H-18 and H-23 to H-26, Diast. A and B),

1.21–1.16 (24H, m, 2×C(29)CH_3, 2×C(30)CH_3, Diast. A and B), 0.91–0.84 (12H, m, H-19, H-27, Diast. A and B); ^{31}P NMR (162 MHz, $CDCl_3$) δ_P 148.8 (P-28, Diast. A), 148.7 (P-28, Diast. B); LRMS *m/z* (ESI^+) 694.575 ($[M+H]^+$, 62%); HRMS *m/z* (ESI^+) found 694.5164 $[M+H]^+$ ($C_{40}H_{73}NO_6P$ requires 694.5170 $[M+H]^+$).

(+)-(7*R*)-4-(Benzyloxy)-2-methyl-10-oxo-3-(propan-2-yl)-5,9-dioxa-3-aza-4-phosphaheptadecan-7-yl hexadecanoate (+)-S17

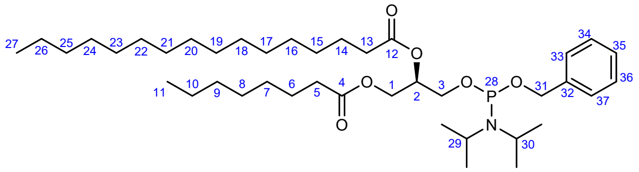

To a solution of benzyloxy-bis(*N*,*N*-diisopropylamino) phosphine **33** (558 mg, 1.65 mmol, 2.5 eq) in anhydrous CH_2Cl_2 (7 mL) was added 1*H*-tetrazole (4.40 mL, 1.98 mmol, 3.0 eq, 0.45 M in MeCN). The solution was stirred for 10 min, after which, a solution of alcohol **(−)-S15** (300 mg, 0.657 mmol, 1.0 eq) in anhydrous CH_2Cl_2 (18 mL) was added dropwise. The reaction mixture was stirred at RT for 18 h, then diluted with CH_2Cl_2 (10 mL), and was then quenched by the addition of a saturated aqueous solution of $NaHCO_3$ (10 mL). The organic phase was extracted with CH_2Cl_2 (3 × 10 mL). The combined organic layers were washed with brine (30 mL), dried over $MgSO_4$, filtered, and then concentrated *in vacuo*. The residue obtained was purified by column chromatography over silica gel (hexane:EtOAc:Et_3N, 85:10:5) to give phosphoramidite **(+)-S17** (420 mg, 92%) as a colorless viscous oil, containing a mixture of two diastereoisomers. Due to its unstable nature, this phosphoramidite was synthesized fresh before each use and used without further characterization: R_f 0.75 (petroleum ether:EtOAc:Et_3N, 85:10:5); $[\alpha]_D^{25}$ +6.9 (*c* 1.0, $CHCl_3$); ν_{max} (thin film)/cm^{-1} 2963, 2926, 2855, 1743, 1457, 976; ^{1}H NMR (400 MHz, $CDCl_3$) δ_H 7.39–7.22 (10H, m, H-33, H-34, H-35, H-36, H-37, Diast. A and B), 5.24–5.15 (2H, m, H-2, Diast. A and B), 4.81–4.61 (4H, m, H-31, Diast. A and B), 4.36 (1H, dd, *J* 8.5, 3.8, H-1' Diast. A), 4.33 (1H, dd, *J* 8.5, 3.8, H-1', Diast. B), 4.19 (1H, dd, *J* 6.3, 5.0, H-1'', Diast. A), 4.16 (1H, dd, *J* 6.3, 5.0, H-1'' Diast. B), 3.83–3.58 (8H, m, H-3, H-29, H-30, Diast. A and B), 2.33–2.26 (8H, m, H-5, H-21, Diast. A and B), 1.66–1.55 (8H, m, H-6, H-14, Diast. A and B), 1.35–1.23 (64H, m, H-7 to H-10 and H-15

to H-26, Diast. A and B), 1.21–1.16 (24H, m, 2×C(29)CH_3, 2×C(30)CH_3), 0.92–0.84 (12H, m, H-11, H-28, Diast. A and B). ^{31}P NMR (162 MHz, $CDCl_3$) δ_P 148.8 (P-28, Diast. A), 148.7 (P-28, Diast. B); LRMS *m/z* (ESI$^+$) 694.549 ([M+H]$^+$, 86%).

(+)-(2*R*)-3-{[(Benzyloxy)({(3a*R*,4*S*,5*R*,6*S*,7*R*,7a*R*)-5,7-bis(benzyloxy)-6-[(3-oxo-1,5-dihydro-3*H*-2,4,3^5-benzodioxaphosphepin-3-yl)oxy]hexahydrospiro[1,3-benzodioxole-2,1'-cyclopentan]-4-yl}oxy)phosphoryl]oxy}-2-(octanoyloxy)propyl hexadecanoate (+)-S18

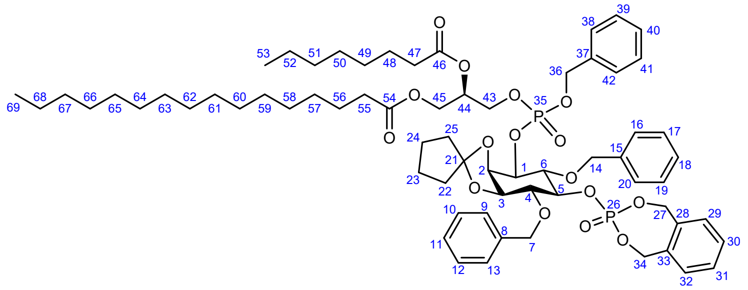

To a solution of phosphoramidite **(+)-S16** (270 mg, 0.387 mmol, 3.0 eq) in anhydrous CH_2Cl_2 (6 mL) was added 1*H*-tetrazole (0.86 mL, 0.39 mmol, 3.0 eq, 0.45 M in MeCN). After 10 min, protected inositol **(+)-S6** (78 mg, 0.13 mmol, 1 eq) was added as a single batch to the cloudy reaction mixture, and the mixture was stirred at RT for a further 18 h. The reaction mixture was then cooled to −78 °C and *m*CPBA (66 mg, 0.39 mmol, 3.0 eq, 77%) was added in one portion. The reaction mixture was stirred for 1 h, warmed to RT, stirred for a further 3 h, and then quenched by the addition of an aqueous solution of $Na_2S_2O_3$ (10% w/v, 6 mL). After stirring for 15 min, the aqueous phase was extracted with CH_2Cl_2 (4 × 5 mL) and the combined organic layers were washed with brine (10 mL), dried over Na_2SO_4, filtered, and then concentrated *in vacuo*. Purification by column chromatography over silica gel (petroleum ether:EtOAc, 10:0 to 5:5) yielded **(+)-S18** (128 mg, 81%) as a colorless, amorphous solid containing a mixture of two diastereoisomers: R_f 0.34 (petroleum ether:EtOAc, 5:5); $[\alpha]_D^{25.6}$ +2.48 (*c* 1.0, $CHCl_3$); ν_{max} (thin film)/cm^{-1} 2954, 2925, 2854, 1743, 1338, 1013, 735, 698; ^{1}H NMR (600 MHz, CD_2Cl_2) δ_H 7.43–7.25 (32H, m, H-9 to H-13, H-16 to H-20, H-38 to H-42, H-30 and H-31 Diast. A and B), 7.23–7.20 (4H, m, H-29 and H-32 Diast. A and B), 5.20–5.15 (1H, m, H-44 Diast. A), 5.12–4.93 (13H, m, H-44 Diast. B, H-27, H-34, H-36 Diast. A and B), 4.86–4.81 (4H, m, H-7, H-14 Diast. A and B), 4.76–4.71 (4H, m, H-7', H-14' Diast. A and B), 4.71–4.66 (2H, m, H-1 Diast.

A and B), 4.63–4.57 (2H, m, H-5 Diast. A and B), 4.49 (1H, dd, *J* 6.3, 3.7, H-2 Diast. A), 4.47 (1H, dd, *J* 6.3, 3.7, H-2 Diast. B), 4.27–4.22 (3H, m, H-3 Diast. A and B, H-45 Diast. A), 4.19–4.04 (7H, m, H-45' Diast. A, H-45 Diast. B, H-43 Diast. A and B, H-43' Diast. A, H-6 Diast. A and B), 4.01 (1H, ddd, *J* 11.0, 6.5, 4.2, H-43' Diast. B), 3.95 (1H, dd, *J* 12.0, 6.0, H-45' Diast B), 3.91–3.87 (2H, m, H-4 Diast. A and B), 2.31–2.20 (8H, m, H-47, H-55 Diast. A and B), 2.01–1.89 (4H, m, H-22, H-25 Diast. A and B), 1.76–1.50 (20H, m, H-22', H-25' Diast. A and B, H-23, H-24, H-48, H-56 Diast. A and B), 1.32–1.22 (64H, m, H-49 to H-52, H-57 to H-68 Diast. A and B), 0.88 (6H, t, *J* 7.0, H-53 or H-69 Diast. A and B), 0.87 (6H, t, *J* 7.0, H-53 or H-69 Diast. A and B);^{13}C NMR (151 MHz, CD_2Cl_2) δ_C 173.42, 173.38 (C-54 Diast. A and B), 173.1, 173.0 (C-46 Diast. A and B), 138.5, 138.40, 138.35 (C-8, C-15 Diast. A and B), 136.27 (d, *J* 7.8, C-37 Diast. A), 136.21 (d, *J* 7.6, C-37 Diast. B), 135.84, 135.79 (C-28, C-33 Diast. A and B) 129.38, 129.36, 129.1, 128.99, 128.96, 128.93, 128.9, 128.7, 128.6, 128.38, 128.35, 128.32, 128.27, 128.1, 128.06 (t, *J* 2.8) (C-9 to C-13, C-16 to C-20, C-29 to C-32, C-38 to C-42 Diast. A and B), 120.5 (C-21 Diast. A and B), 80.49, 80.45 (C-5 Diast. A and B), 79.59, 79.57 (C-4 Diast. A and B), 78.69–78.45 (m_P, C-6 Diast. A and B), 77.7 (C-3 Diast. A and B), 75.64 (t, *J* 5.8, C-1 Diast. A and B), 74.6 (C-2, C-7 Diast. A and B), 73.5 (C-14 Diast. A and B), 69.89 (d, *J* 2.7, C-36 Diast. A), 69.86 (d, *J* 2.7, C-36 Diast. B), 69.68 (t, *J* 7.3, C-44 Diast. A and B), 68.73, 68.68 (C-27 and C-34 Diast. A and B), 66.07 (d, *J* 5.3, C-43 Diast. A), 65.94 (d, *J* 5.3, C-43 Diast. B), 62.0 (C-45 Diast. A), 61.9 (C-45 Diast. B), 36.9, 36.80, 36.77 (C-22, C-25 Diast. A and B), 34.5, 34.4, 34.34, 34.32 (C-47, C-55 Diast. A and B), 32.3, 32.1, 30.12, 30.10, 30.07, 29.9, 29.8, 29.7, 29.5, 29.44, 29.42, 29.4 (C-49, C-50, C-57 to C-66 Diast. A and B), 25.3, 25.23, 25.20 (C-48, C-56 Diast. A and B), 24.25, 24.22, 23.6 (C-23, C-24 Diast. A and B), 23.1, 23.0 (C-52, C-68 Diast. A and B), 14.29, 14.25 (C-53, C-69 Diast. A and B). ^{31}P NMR (243 MHz, CD_2Cl_2) δ_P −0.9 (P-26), −1.8 (P-35 Diast. A), −1.9 (P-35 Diast B); LRMS *m/z* (ESI$^+$) 1217.63 ($[M+H]^+$, 100%); HRMS *m/z* (ESI$^+$) found 1239.5873 $[M+Na]^+$ ($C_{67}H_{94}O_{16}P_2Na$ requires 1239.5909 $[M+Na]^+$); NP-HPLC t_R = 4.66 min (Diast. A), 5.19 (Diast B.), 99.7% @ 254 nm, 99.0% @ 220 nm.

(+)-(6*R*)-3-({(3a*R*,4*S*,5*R*,6*S*,7*R*,7a*R*)-5,7-Bis(benzyloxy)-6-[(3-oxo-1,5-dihydro-3*H*-2,4,3^5-benzodioxaphosphepin-3-yl)oxy]hexahydrospiro[1,3-benzodioxole-2,1'-cyclopentan]-4-yl}oxy)-3,9-dioxo-1-phenyl-2,4,8-trioxa-3^5-phosphahexadecan-6-yl hexadecanoate (+)-S19

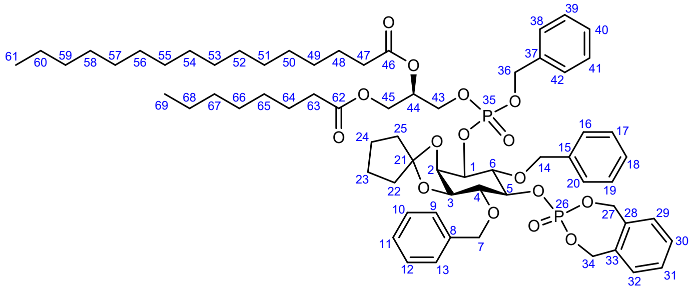

To a solution of phosphoramidite **(+)-S17** (270 mg, 0.387 mmol, 3.0 eq) in anhydrous CH_2Cl_2 (6 mL) was added 1*H*-tetrazole (0.86 mL, 0.39 mmol, 3.0 eq, 0.45 M in MeCN). After 10 min, inositol **(+)-S6** (78 mg, 0.13 mmol, 1.0 eq) was added as a single batch to the cloudy reaction mixture, and the mixture was stirred at RT for a further 18 h. The reaction mixture was then cooled to −78 °C and *m*CPBA (66 mg, 0.39 mmol, 3.0 eq, >77%) was added in one portion. The reaction mixture was stirred for 1 h, warmed to RT, stirred for a further 3 h, and then quenched by the addition of an aqueous solution of $Na_2S_2O_3$ (10% w/v, 5 mL). After stirring for 15 min, the aqueous phase was extracted with CH_2Cl_2 (4 × 5 mL) and the combined organic layers were washed with brine (15 mL), dried over Na_2SO_4, filtered, and then concentrated *in vacuo*. Purification by column chromatography over silica gel (petroleum ether:EtOAc, 10:0 to 5:5) yielded **(+)-S19** (137 mg, 88%) as a colorless, amorphous solid containing a mixture of two diastereoisomers: R_f 0.34 (petroleum ether:EtOAc, 5:5); $[\alpha]_D^{25}$ +2.09 (*c* 1.0, $CHCl_3$); ν_{max} (thin film)/cm^{-1} 2925, 2854, 1743, 1338, 1014, 735, 698; ^1H NMR (600 MHz, CD_2Cl_2) δ_H 7.45–7.24 (34H, m, H-9 to H-13, H-16 to H-20, H-38 to H-42, H-30 and H-31 Diast. A and B), 7.24–7.19 (4H, m, H-29 and H-32 Diast. A and B), 5.20–5.15 (1H, m, H-44 Diast. A), 5.12–4.92 (13H, m, H-44 Diast. B, H-27, H-34, H-36 Diast. A and B), 4.86–4.81 (4H, m, H-7, H-14 Diast. A and B), 4.76–4.71 (4H, m, H-7', H-14' Diast. A and B), 4.71–4.66 (2H, m, H-1 Diast. A and B), 4.63–4.57 (2H, m, H-5 Diast. A and B), 4.49 (1H, dd, *J* 6.4, 3.8, H-2 Diast. A), 4.47 (1H, dd, *J* 6.3, 3.7, H-2 Diast. B), 4.27–4.22 (3H, m, H-3 Diast.

A and B, H-45 Diast. A), 4.19–4.05 (7H, m, H-45’ Diast. A, H-45 Diast. B, H-43 Diast. A and B, H-43’ Diast. A, H-6 Diast. A and B), 4.01 (1H, ddd, *J* 10.9, 6.5, 4.1, H-43’ Diast. B), 3.95 (1H, dd, *J* 12.0, 6.0, H-45’ Diast B), 3.89 (2H, ddd, *J* 7.3, 6.0, 2.8, H-4 Diast. A and B), 2.31–2.20 (8H, m, H-47, H-63 Diast. A and B), 2.00–1.89 (4H, m, H-22, H-25 Diast. A and B), 1.76–1.50 (20H, m, H-22’, H-25’ Diast. A and B, H-23, H-24, H-48, H-64 Diast. A and B), 1.40–1.21 (64H, m, H-49 to H-60, H-65 to H-68 Diast. A and B), 0.88 (12H, t, *J* 6.8, H-61, H-69 Diast. A and B); ^{13}C NMR (151 MHz, CD_2Cl_2) δ_C 173.41, 173.36 (C-62 Diast. A and B), 173.1, 173.0 (C-46 Diast. A and B), 138.5, 138.4, 138.3 (C-8, C-15 Diast. A and B), 136.25 (d_p, J_p 7.1, C-37 Diast. A), 136.18 (d_p, J_p 7.1, C-37 Diast. B), 135.82, 135.77 (C-28, C-33 Diast. A and B), 129.4, 129.3, 129.1, 128.97, 128.95, 128.92, 128.86, 128.7, 128.6, 128.4, 128.34, 128.30, 128.25, 128.1, 128.05 (t_p, J_p 2.9) (C-9 to C-13, C-16 to C-20, C-29 to C-32, C-38 to C-42 Diast. A and B), 120.4 (C-21 Diast. A and B), 80.45, 80.41 (C-5 Diast. A and B), 79.56, 79.54 (C-4 Diast. A and B), 78.6–78.4 (m_P, C-6 Diast. A and B), 77.6 (C-3 Diast. A and B), 75.6 (t_p, J_p 5.5, C-1 Diast. A and B) 74.64, 74.61, 74.60, 74.58, (C-2, C-7 Diast. A and B) 73.48, 73.47 (C-14 Diast. A and B), 69.87 (d_p, J_p 2.9, C-36 Diast. A), 69.83 (d_p, J_p 2.9, C-36 Diast. B), 69.73–69.57 (m_p, C-44 Diast. A and B), 68.75–68.61 (m_p, C-27 and C-34 Diast. A and B), 66.05 (d_p, J_p 5.3, C-43 Diast. A), 65.92 (d_p, J_p 5.2, C-43 Diast. B), 62.0 (C-45 Diast. A), 61.9 (C-45 Diast. B), 36.9, 36.79, 36.76 (C-22, C-25 Diast. A and B), 34.47, 34.42, 34.32, 34.29 (C-47, C-63 Diast. A and B), 32.3 (C-59 Diast. A and B), 32.1 (C-67 Diast. A and B), 30.11, 30.09, 30.06, 29.9, 29.8, 29.7, 29.49, 29.47, 29.46, 29.3 (C-49 to C-58, C-65, C-66 Diast. A and B), 25.23, 25.22, 25.20 (C-48, C-64 Diast. A and B), 24.23, 24.20, 23.6 (C-23, C-24 Diast. A and B), 23.1, 23.0 (C-60, C-68 Diast. A and B), 14.3, 14.2 (C-61, C-69 Diast. A and B); ^{31}P NMR (162 MHz, CD_2Cl_2) δ_P −0.9 (P-26), −1.8 (P-35 Diast. A), −1.9 (P-35 Diast. B); LRMS *m/z* (ESI$^+$) 1217.67 ([M+H]$^+$, 100%); HRMS *m/z* (ESI$^+$) found 1239.5879 [M+Na]$^+$ ($C_{67}H_{94}O_{16}P_2Na$ requires 1239.5909 [M+Na]$^+$); NP-HPLC t_R = 4.74 min (Diast. A), 5.19 min (Diast B.), 99.6% @ 254 nm, 99.5% @ 220 nm.

(+)-(2*R*)-3-[(Hydroxy{[(1R,2R,3R,4R,5S,6R)-2,3,4,6-tetrahydroxy-5-(phosphonooxy)cyclohexyl]oxy}phosphoryl)oxy]-2-(octanoyloxy)propyl hexadecanoate (+)-7

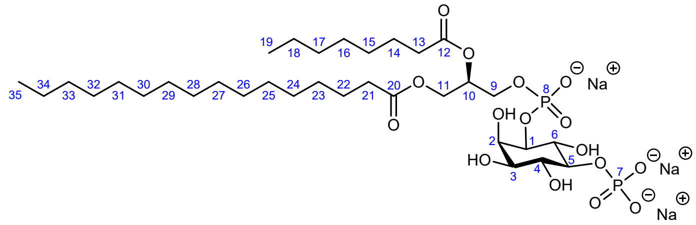

To a solution of protected PI(5)P **(+)-18** (36 mg, 0.030 mmol, 1.0 eq) in a mixture of ᵗBuOH (1.36 mL) and H_2O (0.24 mL) was added palladium black (64 mg, 0.60 mmol, 20 eq). The flask was purged with $Ar_{(g)}$, before being placed under vacuum. $H_{2(g)}$ was then bubbled through the solution, and the reaction mixture was stirred vigorously at RT under an atmosphere of $H_{2(g)}$ for 17 h. The flask was then purged with $Ar_{(g)}$ and $NaHCO_3$ (8 mg, 0.09 mmol, 3.0 eq) was then added in one portion to the reaction mixture. The mixture was stirred for a further 2 h under an atmosphere of $H_{2(g)}$, with freshly added palladium black (32 mg, 0.30 mmol, 10 eq) to ensure complete deprotection. The reaction mixture was finally purged with $Ar_{(g)}$ and filtered through a PTFE syringe filter. The remaining palladium was further washed with milliQ H_2O (3 × 5 mL), which again, was passed through the same syringe filter. The combined filtrates were lyophilized to give **(+)-7** (15 mg, 60%) as a fluffy, colorless solid. The compound was stored at −80 °C as the sodium salt: ν_{max} (solid)/cm^{-1} 2922, 2853, 1737, 1068, 975, 864, 816, 721; ^{1}H NMR (600 MHz, MeOD) δ_H 5.28–5.22 (1H, m, H-10), 4.43* (1H, m, H-11') 4.20 (1H, dd, *J* 12.2, 7.6, H-11''), 4.16 (1H, dd, *J* 2.8, 2.8, H-2), 4.08–3.97 (3H, m, H-1 and H-9), 3.87–3.73 (3H, m, H-4, H-5 and H-6), 3.51 (1H, dd, *J* 9.6, 2.8, H-3), 2.37–2.28 (4H, m, H-13 and H-21), 1.65–1.52 (4H, m, H-14 and H-22), 1.36–1.21 (32H, m, H-15 to H-19 and H-23 to H-34), 0.87 (3H, t, *J* 7.0, H-19 or H-35), 0.87 (3H, t, *J* 7.0, H-19 or H-35); ^{31}P NMR (243 MHz, MeOD) δ_P 4.32 (P-7), 0.10 (P-8); ^{13}C NMR (151 MHz, MeOD) δ_C 175.2, 174.9 (C-12, C-20), 79.1 (C-5), 77.0 (C-1), 72.9 (C-4), 72.1 (C-2), 72.0 (C-6), 71.8 (C-3), 71.44–71.38 (m, C-10), 64.4 (C-9), 63.7 (C-11), 34.9, 34.8 (C-13, C-

21), 32.6, 32.4, 30.32, 30.28, 30.19, 30.0, 29.8, 29.72, 29.68, 25.61, 25.56, 23.31, 23.28 (C-14 to C-18 and C-22 to C-34), 14.5 (C-19 and C-35); LRMS *m/z* (ESI^-) 777.3 ($[M–H]^-$, 100%); HRMS *m/z* (ESI^-) found 777.3592 $[M–H]^-$ ($C_{33}H_{63}O_{16}P_2$ requires 777.3597 $[M–H]^-$).*obscured by H_2O peak, observed in HSQC.

(2*R*)-1-[(Hydroxy{[(1*R*,2*R*,3*R*,4*R*,5*S*,6*R*)-2,3,4,6-tetrahydroxy-5-(phosphonooxy)cyclohexyl]oxy}phosphoryl)oxy]-3-(octanoyloxy)propan-2-yl hexadecanoate (+)-8

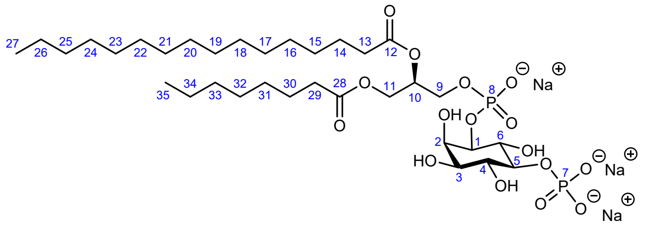

To a solution of protected PI(5)P **(+)-S19** (40 mg, 0.033 mmol, 1.0 eq) in a mixture of ^tBuOH (1.36 mL) and H_2O (0.24 mL) was added palladium black (70 mg, 0.66 mmol, 20 eq). The flask was purged with $Ar_{(g)}$, before being placed under vacuum. $H_{2(g)}$ was then bubbled through the solution, and the reaction mixture was stirred vigorously at RT under an atmosphere of $H_{2(g)}$ for 17 h. The flask was then purged with $Ar_{(g)}$ and $NaHCO_3$ (8 mg, 0.09 mmol, 3.0 eq) was then added in one portion to the reaction mixture. The mixture was stirred for a further 2 h under an atmosphere of $H_{2(g)}$, with freshly added palladium black (35 mg, 0.33 mmol, 10 eq) to ensure complete deprotection. The reaction mixture was finally purged with $Ar_{(g)}$ and filtered through a PTFE syringe filter. The remaining palladium was further washed with milliQ H_2O (3 × 5 mL), which again, was passed through the same syringe filter. The combined filtrates were lyophilized to give **(+)-8** (21 mg, 75%) as a fluffy, colorless solid. The compound was stored at −80 °C as the sodium salt: ν_{max} (solid)/cm^{-1} 2922, 2853, 1739, 1225, 1068, 978, 866, 833, 709; ^1H NMR (600 MHz, MeOD) δ_H 5.27–5.22 (1H, m, H-10), 4.42* (1H, m, H-11’), 4.19 (1H, dd, *J* 12.2, 7.5, H-11’’), 4.16 (1H, dd, *J* 2.9, 2.9, H-2), 4.08–3.92 (3H, m, H-1 and H-9), 3.88–3.73 (3H, m, H-4, H-5 and H-6), 3.51 (1H, dd, *J* 9.7, 2.9, H-3), 2.36–2.28 (4H, m, H-13 and H-29), 1.64–1.53 (4H, m, H-14 and H-30), 1.35–1.20 (32H, m, H-15 to H-26 and H-31 to H-34), 0.87 (3H, t, *J* 7.1, H-27 or H-35),

0.87 (4H, t, *J* 7.1, H-27 or H-35);^{31}P NMR (243 MHz, MeOD) δ_P 3.72 (P-7), 0.07 (P-8); ^{13}C NMR (151 MHz, MeOD) δ_C 175.2, 174.9 (C-12, C-28), *78.9 (C-5)*, 77.0 (C-1), 72.9 (C-4), 72.1 (C-2), 71.9 (C-6), 71.8 (C-3), 71.3 (C-10), 64.4 (C-9), 63.7 (C-11), 34.9, 34.8 (C-13 and C-29), 32.6, 32.4 (C-25 and C-33), 30.35, 30.32, 30.28, 30.24, 30.1, 30.0, 29.80, 29.76, 29.6 (C-15 to C-24 and C-31 to C-32), 25.6, 25.5 (C-14 and C-30), 23.30, 23.25 (C-26 and C-34), 14.50, 14.47 (C-27 and C-35); LRMS *m/z* (ESI⁻) 777.3 ([M–H]⁻, 100%); HRMS *m/z* (ESI⁻) found 777.3586 [M–H]⁻ ($C_{33}H_{63}O_{16}P_2$ requires 777.3597 [M–H]⁻). *obscured by H_2O peak, observed in HSQC.

(+)-(2*R*)-3-{[(2*S*)-2-Methoxy-2-phenylacetyl]oxy}-2-(octanoyloxy)propyl hexadecanoate (+)-S20

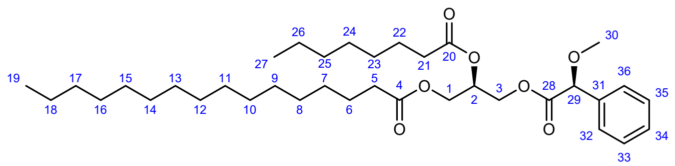

(*S*)-(+)-α-Methoxyphenylacetic acid (5 mg, 0.03 mmol, 2.0 eq), *N*,*N*′-dicyclohexylcarbodiimide (10 mg, 0.049 mmol, 2.7 eq) and 4-DMAP (1 mg, 0.008 mmol, 0.5 eq) were added to a solution of alcohol (−)-**S14** (7 mg, 0.02 mmol, 1.0 eq) in CH_2Cl_2 (0.5 mL). After stirring at RT for 4 h, the reaction mixture was diluted with CH_2Cl_2 (5 mL), and the organic layer was washed with a saturated aqueous solution of $NaHCO_3$ (2 × 3 mL), and brine (3 mL), then dried over $MgSO_4$, filtered, and concentrated *in vacuo*. Purification by column chromatography over silica gel (petroleum ether:Et_2O, 10:0 to 7:3) gave **(+)-S20** (10 mg, 83%) as a colorless film: R_f 0.77 (petroleum ether:EtOAc, 6:4); $[\alpha]_D^{25}$ +18.4 (*c* 0.83, $CHCl_3$); ν_{max} (thin film)/cm^{-1} 2921, 2851, 1745, 1463, 1163, 1114, 727; ^{1}H NMR (600 MHz, $CDCl_3$) δ_H 7.44–7.31 (5H, m, H-32 to H-36), 5.20–5.16 (1H, m, H-2), 4.77 (1H, s, H-29), 4.36 (1H, dd, *J* 11.9, 4.3, H-3’), 4.20 (1H, dd, *J* 11.9, 5.6, H-3’’), 4.18 (1H, dd, *J* 11.9, 4.5, H-1’), 3.97 (1H, dd, *J* 11.9, 5.9, H-1’’), 3.41 (3H, s, H-30), 2.28–2.24 (2H, m, H-5), 2.23–2.13 (2H, m, H-21), 1.61–1.50 (4H, m, H-6 and H-22), 1.33–1.21 (32H, m, H-7 to H-18 and H-23 to H-26), 0.89 (3H, t, *J* 7.0, H-19 or H-27), 0.88 (3H, t, *J* 7.0, H-19 or H-27); ^{13}C NMR (151

MHz, $CDCl_3$) δ_C 173.3 (C-4), 172.8 (C-20), 170.3 (C-28), 136.1 (C-31), 129.0 (C-34), 128.8 (C-32 and C-36), 127.3 (C-33 and C-35), 82.5 (C-29), 68.8 (C-2), 62.7 (C-3), 61.9 (C-1), 57.5 (C-30), 34.2 (C-21), 34.1 (C-5), 32.1, 31.8 (C-17, C-25), 29.85, 29.81, 29.77, 29.6, 29.5, 29.4, 29.3, 29.2, 29.1 (C-7 to C-16, C-23, C-24) 24.98, 24.89 (C-6, C-22), 22.84, 22.76 (C-18, C-26), 14.3, 14.2 (C-19, C-27); LRMS *m/z* (ESI^+) 627.4 ($[M+Na]^+$, 100%); HRMS *m/z* (ESI^+) found 627.4227 $[M+Na]^+$ ($C_{36}H_{60}O_7Na$ requires 627.4231 $[M+Na]^+$).

(–)-(2*R*)-3-{[(2*R*)-2-Methoxy-2-phenylacetyl]oxy}-2-(octanoyloxy)propyl hexadecanoate (–)-S21

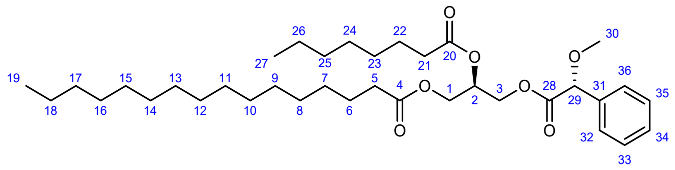

(*R*)-(–)-α-Methoxyphenylacetic acid (5 mg, 0.0310 mmol, 2.0 eq), *N*,*N*′-dicyclohexylcarbodiimide (10 mg, 0.049 mmol, 2.7 eq) and 4-DMAP (1 mg, 0.008 mmol, 0.5 eq) were added to a solution of **(–)-S14** (7 mg, 0.016 mmol, 1.0 eq) in CH_2Cl_2 (0.5 mL). After stirring at RT for 4 h, the reaction mixture was diluted with CH_2Cl_2 (5 mL), and the organic layer was washed with a saturated aqueous solution of $NaHCO_3$ (2 × 3 mL), and brine (3 mL), then dried over $MgSO_4$, filtered, and concentrated *in vacuo*. Purification by column chromatography over silica gel (petroleum ether:Et_2O, 10:0 to 7:3) gave **(–)-S21** (7 mg, 75%) as a colorless film: R_f 0.77 (petroleum ether:EtOAc, 6:4); $[\alpha]_D^{25}$ –27.9 (*c* 0.58, $CHCl_3$); ν_{max} (thin film)/cm^{-1} 2923, 2853, 1744, 1495, 1164, 1114, 727; ^1H NMR (600 MHz, $CDCl_3$) δ_H 7.44–7.31 (5H, m, H-32 to H-36), 5.24–5.20 (1H, m, H-2), 4.77 (1H, s, H-29), 4.34 (1H, dd, *J* 11.9, 4.0, H-3’), 4.19 (1H, dd, *J* 11.9, 6.3, H-3’’), 4.16 (1H, dd, *J* 11.9, 4.5, H-1’), 4.04 (1H, dd, *J* 11.9, 5.8, H-1’’), 3.41 (3H, s, H-30), 2.26 (2H, t, *J* 7.6, H-5), 2.23–2.12 (2H, m, H-21), 1.60–1.51 (4H, m, H-6 and H-22), 1.33–1.21 (32H, m, H-7 to H-18 and H-23 to H-26), 0.89 (3H, t, *J* 7.0, H-19 or H-27), 0.88 (3H, t, *J* 7.1, H-19 or H-27); ^{13}C NMR (151 MHz, $CDCl_3$) δ_C 173.3 (C-4), 172.9 (C-20), 170.4 (C-28), 136.1 (C-31), 129.0 (C-34), 128.8 (C-32

and C-36), 127.3 (C-33 and C-35), 82.5 (C-29), 68.7 (C-2), 63.0 (C-3), 61.9 (C-1), 57.5 (C-30), 34.2 (C-21), 34.1 (C-5), 32.1, 31.8 (C-17, C-25), 29.84, 29.83, 29.80, 29.76, 29.6, 29.5, 29.4, 29.3, 29.2, 29.1 (C-7 to C-16, C-23, C-24), 24.97, 24.90 (C-6, C-22), 22.83, 22.75 (C-18, C-26), 14.3, 14.2 (C-19, C-27); LRMS *m/z* (ESI$^+$) 627.4 ([M+Na]$^+$, 100%); HRMS *m/z* (ESI$^+$ found 627.4229 [M+Na]$^+$ ($C_{36}H_{60}O_7Na$ requires 627.4231 [M+Na]$^+$).

(+)-(2*R*)-1-{[(2*S*)-2-Methoxy-2-phenylacetyl]oxy}-3-(octanoyloxy)propan-2-yl hexadecanoate (+)-S22

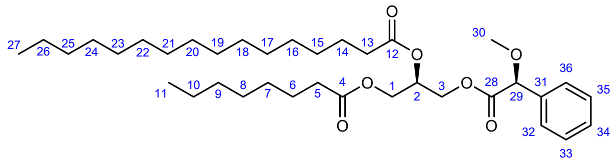

(S)-(+)-α-Methoxyphenylacetic acid (5 mg, 0.03 mmol, 2.0 eq), *N*,*N'*-dicyclohexylcarbodiimide (10 mg, 0.049 mmol, 2.7 eq) and 4-DMAP (1 mg, 0.008 mmol, 0.5 eq) were added to a solution of **(–)-S15** (7 mg, 0.02 mmol, 1.0 eq) in CH_2Cl_2 (0.5 mL). After stirring at RT for 4 h, the reaction mixture was diluted with CH_2Cl_2 (5 mL) and the organic layer was washed with a saturated aqueous solution of $NaHCO_3$ (2 × 3 mL), brine (3 mL), and then dried over $MgSO_4$, before being filtered, and concentrated *in vacuo*. Purification by silica gel column chromatography (petroleum ether:Et_2O, 10:0 to 7:3) resulted in **(+)-S22** (7 mg, 75%) as a colorless film: R_f 0.77 (petroleum ether:EtOAc, 6:4); $[\alpha]_D^{25}$ +35.5 (*c* 0.58, $CHCl_3$); ν_{max} (thin film)/cm^{-1} 2924, 2854, 1744, 1495, 1165, 1114, 728; ^{1}H NMR (600 MHz, $CDCl_3$) δ_H 7.44–7.31 (5H, m, H-32 to H-36), 5.20–5.15 (1H, m, H-2), 4.77 (1H, s, H-29), 4.35 (1H, dd, *J* 11.9, 4.3, H-3'), 4.22–4.16 (2H, m, H-3'' and H-1'), 3.97 (1H, dd, *J* 11.9, 5.8, H-1''), 3.41 (3H, s, H-30), 2.26 (2H, td, *J* 7.4, 0.9, H-5 or H-13), 2.18 (2H, td, *J* 7.6, 4.1, H-5 or H-13), 1.61–1.50 (4H, m, H-6 and H-14), 1.32–1.22 (32H, m, H-7 to H-10 and H-16 to H-26), 0.88 (6H, t, *J* 6.9, H-11 and H-27); ^{13}C NMR (151 MHz, $CDCl_3$) δ_C 173.3 (C-4), 172.8 (C-12), 170.3 (C-28), 136.1 (C-31), 129.0 (C-34), 128.8 (C-32 and C-36), 127.3 (C-33 and C-35), 82.5 (C-29), 68.8 (C-2), 62.7 (C-3), 61.9 (C-1), 57.5 (C-30), 34.2, 34.1 (C-5 and C-13), 32.1, 31.8 (C-9 and C-25),

29.84, 29.81, 29.80, 29.78, 29.6, 29.5, 29.4, 29.21, 29.19, 29.0 (C-7, C-8 and C-15 to C-24), 25.0, 24.9 (C-6, C-14), 22.8, 22.7 (C-10, C-26), 14.3, 14.2 (C-27, C-11); LRMS *m/z* (ESI$^+$) 627.4 ([M+Na]$^+$, 100%); HRMS *m/z* (ESI$^+$) found 627.4229 [M+Na]$^+$ ($C_{36}H_{60}O_7Na$ requires 627.4231 [M+Na]$^+$.

(–)-(2*R*)-1-{[(2*R*)-2-Methoxy-2-phenylacetyl]oxy}-3-(octanoyloxy)propan-2-yl hexadecanoate (–)-S23

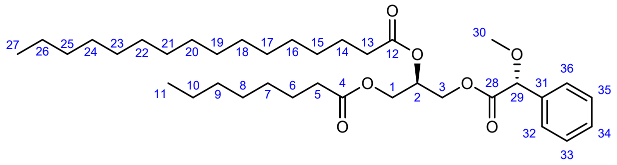

(*R*)-(–)-α-Methoxyphenylacetic acid (5 mg, 0.03 mmol, 2.0 eq), *N*,*N*′-dicyclohexylcarbodiimide (10 mg, 0.049 mmol, 2.7 eq) and 4-DMAP (1 mg, 0.008 mmol, 0.5 eq) were added to a solution of **(–)-S15** (7.0 mg, 0.016 mmol, 1.0 eq) in CH_2Cl_2 (0.5 mL). After stirring at RT for 4 h, the reaction mixture was diluted with CH_2Cl_2 (5 mL), and the organic layer was washed with a saturated aqueous solution of $NaHCO_3$ (2 × 3 mL) and brine (3 mL), and then dried over $MgSO_4$, filtered, and concentrated *in vacuo*. Purification by silica gel column chromatography (petroleum ether:Et_2O, 10:0 to 7:3) gave **(–)-S23** (8 mg, 81%) as a colorless film: R_f 0.77 (petroleum ether:EtOAc, 6:4); $[\alpha]_D^{25}$ –31.8 (*c* 0.67, $CHCl_3$); ν_{max} (thin film)/cm^{-1} 2925, 2854, 1745, 1457, 1165, 1115, 728; ^{1}H NMR (600 MHz, $CDCl_3$) δ_H 7.44–7.31 (5H, m, H-32 to H-36), 5.24–5.20 (1H, m, H-2), 4.77 (1H, s, H-29), 4.34 (1H, dd, *J* 11.9, 4.0, H-3’), 4.21–4.15 (2H, m, H-3’’ and H-1’), 4.04 (1H, dd, *J* 11.9, 5.8, H-1’’), 3.41 (3H, s, H-30), 2.26 (2H, t, *J* 7.6, H-5 or H-13), 2.22–2.12 (2H, m, H-5 or H-13), 1.62–1.50 (4H, m, H-6 and H-14), 1.32–1.23 (32H, m, H-7 to H-10 and H-16 to H-26), 0.88 (6H, t, *J* 6.9, H-11 and H-27); ^{13}C NMR (151 MHz, $CDCl_3$) δ_C 173.3 (C-4), 172.9 (C-12), 170.4 (C-28), 136.1 (C-31), 129.0 (C-34), 128.8 (C-32 and C-36), 127.3 (C-33 and C-35), 82.5 (C-29), 68.7 (C-2), 63.0 (C-3), 61.9 (C-1), 57.5 (C-30), 34.2, 34.1 (C-5, C-13), 32.1, 31.8 (C-9, C-25), 29.84, 29.81, 29.80, 29.78, 29.6, 29.5, 29.4, 29.22, 29.19, 29.0 (C-7, C-8 and C-15 to C-24), 25.0, 24.9 (C-6, C-14), 22.8, 22.7 (C-

10, C-26), 14.3, 14.2 (C-11, C-27); LRMS *m/z* (ESI$^+$) 627.4 ([M+Na]$^+$, 100%); HRMS *m/z* (ESI$^+$) found 627.4228 [M+Na]$^+$ ($C_{36}H_{60}O_7Na$ requires 627.4231 [M+Na]$^+$).

(+)-(*S*)-3-(Bis(4-methoxyphenyl)(phenyl)methoxy)-2-((3-(3-(dodec-11-yn-1-yl)-3*H*-diazirin-3-yl)propanoyl)oxy)propyl palmitate S24

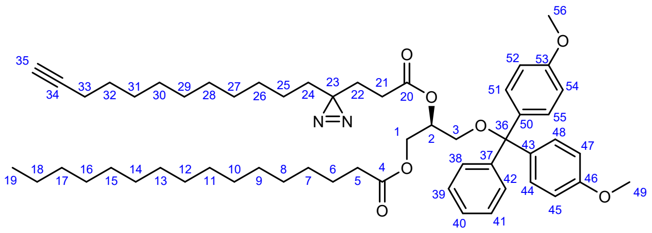

To a solution of alcohol **S10** (684 mg, 1.08 mmol, 1.0 eq) in CH_2Cl_2 (6 mL) was added DCC (446 mg, 2.16 mmol, 2.0 eq), 4-DMAP (8 mg, 0.1 mmol, 0.06 eq) and diazirine **27** (300 mg, 1.08 mmol, 1.0 eq). The solution was stirred at RT for 18 h, after which the reaction mixture was diluted with hexane (20 mL). The suspension was filtered through celite, and the celite subsequently was washed with hexane (3 × 10 mL). The combined filtrates were concentrated *in vacuo* at RT and the resulting residue was purified by column chromatography over silica gel (petroleum ether:Et_2O, 10:0 to 8:2) which yielded (+)-**S24** (400 mg, 41%) as a colorless oil with a slight impurity corresponding to the migration product: R_f 0.49 (petroleum ether:EtOAc, 9:1); $[\alpha]_D^{25}$ +10.66 (*c* 1.0, $CHCl_3$); ν_{max} (thin film)/cm^{-1} 3309, 2925, 2854, 1742, 1608, 1509, 1251, 1177, 1037, 829; ^{1}H NMR (600 MHz, $CDCl_3$) δ_H 7.43–7.38 (2 H, m, H-38, H-42), 7.31–7.26 (6 H, m, H-39, H-41, H-44, H-48, H-51, H-55), 7.23–7.19 (1 H, m, H-40), 6.84–6.80 (4 H, m, H-45, H-47, H-52, H-54), 5.27–5.21 (1 H, m, H-2), 4.34 (1 H, dd, *J* 11.9, 3.6, H-1'), 4.22 (1 H, dd, *J* 11.9, 6.6, H-1''), 3.79 (6 H, s, H-49, H-56), 3.22 (2 H, dd, *J* 5.0, 2.5, H-3), 2.24 (2 H, t, *J* 7.6, H-5), 2.17 (2 H, td, *J* 7.1, 2.6, H-33), 2.12 (2 H, td, *J* 7.6, 2.8, H-21 or H-22), 1.93 (1 H, t, *J* 2.6, H-35), 1.75–1.70 (2 H, m, H-21 or H-22), 1.56–1.49 (4 H, m, H-6 and H-32), 1.40–1.35 (4 H, m, H-24, H-31), 1.33–1.18 (34 H, m, H-30 to H-26 and H-18 to H-7), 1.10–1.03 (2 H, m, H-25), 0.88 (3 H, t, *J* 7.0, H-19); ^{13}C NMR (151 MHz, $CDCl_3$) δ_C 173.6 (C-4), 171.7 (C-20), 158.7 (C-46 and C-53), 144.7 (C-37), 135.83, 135.81 (C-43, C-50), 130.13, 130.12 (C-44, C-48, C-51, C-55), 128.2, 128.0 (C-38, C-39, C-41, C-42), 127.0 (C-40), 113.3 (C-52, C-54, C-45 and C-47), 86.3 (C-36), 84.9 (C-34), 71.2 (C-2), 68.2 (C-35), 62.9 (C-1), 62.0 (C-3), 55.3 (C-56 and C-49), 34.2 (C-5),

32.9 (C-24), 32.1, 29.85, 29.81, 29.80, 29.78, 29.64, 29.60, 29.56, 29.55, 29.50, 29.43, 29.42, 29.31, 29.28, 29.22, 29.20, 28.9 (C-7 to C-17, C-20, C-26 to C-31) 28.8, 28.6, 28.4 (C-21, C-22, C-32), 28.3 (C-23), 25.0 (C-6), 24.0 (C-25), 22.8 (C-18), 18.5 (C-33), 14.3 (C-19); LRMS *m/z* (ESI$^+$) 915.6 ([M+Na]$^+$, 100%); HRMS *m/z* (ESI$^+$) found 915.5853 [M+Na]$^+$ ($C_{56}H_{80}O_7N_2Na$) requires 915.5858 [M+Na]$^+$).

(*R*)-2-((3-(3-(dodec-11-yn-1-yl)-3H-diazirin-3-yl)propanoyl)oxy)-3-((*S*)-2-methoxy-2-phenylacetoxy)propyl palmitate (+)-S25

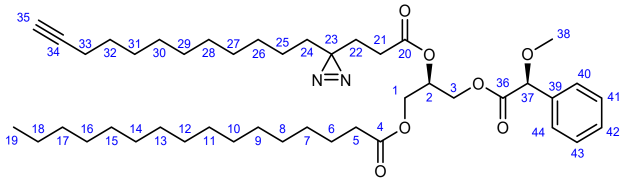

(*S*)-(+)-α-Methoxyphenylacetic acid (6 mg, 0.04 mmol, 2.2 eq), *N*,*N*′-dicyclohexylcarbodiimide (10 mg, 0.046 mmol, 2.7 eq) and 4-DMAP (1 mg, 0.008 mmol, 0.5 eq) were added to a solution of alcohol **32** (10 mg, 0.017 mmol, 1 eq) in CH_2Cl_2 (0.5 mL). After stirring at RT for 16 h, the reaction mixture was diluted with CH_2Cl_2 (5 mL), the organic layer was washed with 1 M $HCl_{(aq)}$ (5 mL), then a saturated aqueous solution of $NaHCO_3$ (5 mL) and then brine (5 mL), and then dried over Na_2SO_4, filtered, and concentrated *in vacuo*. Purification by silica gel column chromatography (petroleum ether:Et_2O, 10:0 to 7:3) gave **(+)-25** (8 mg, 65%) as a colorless film: R_f 0.63 (petroleum ether:Et_2O, 6:4); $[\alpha]_D^{25}$ +33.1 (*c* 0.8, $CHCl_3$); ν_{max} (thin film)/cm^{-1} 3313, 2928, 2855, 1748, 1466, 1171; ^{1}H NMR (600 MHz, $CDCl_3$) δ_H 7.47–7.31 (5 H, m, H-40 to H-44), 5.19–5.11 (1 H, m, H-2), 4.78 (1 H, s, H-37), 4.37 (1 H, dd, *J* 12.0, 4.1,m, H-3’), 4.22–4.16 (2 H, m, H-3’’, H-1’), 3.96 (1 H, dd, *J* 12.0, 5.8, H-1’’), 3.40 (3 H, s, H-38), 2.27 (2 H, t, *J* 7.6, H-5), 2.17 (2 H, td, *J* 7.2, 2.7, H-33), 1.98–1.92 (3 H, m, H-21, H-35), 1.66–1.61 (2 H, m, H-22), 1.60–1.54 (2 H, m, H-6), 1.54–1.49 (2 H, m, H-32), 1.41–1.33 (4 H, m, H-24, H-31), 1.32–1.18 (34 H, m, H-7 to H-18 and H-26 to H-30), 1.10–1.02 (2 H, m, H-25), 0.88 (3 H, t, *J* 7.0, H-19); ^{13}C NMR (151 MHz, $CDCl_3$) δ_C 173.3 (C-4), 171.4 (C-20), 170.3 (C-36), 136.1 (C-39), 129.1 (C-42), 128.9,

127.3 (C-40, C-41, C-43, C-44), 84.9 (C-34), 82.4 (C-37), 69.3 (C-2), 68.2 (C-35), 62.5 (C-3), 61.8 (C-1), 57.5 (C-38), 34.1 (C-5), 32.8 (C-24), 32.1, 29.9, 29.84, 29.81, 29.77, 29.62, 29.56, 29.53, 29.51, 29.4, 29.3, 29.3, 29.2, 28.9, 28.6 (C-7 to C-17 and C-25 to C-32), 28.5 (C-21), 28.2 (C-23), 28.1 (C-22), 25.0 (C-6), 23.9 (C-29), 22.8 (C-18), 18.5 (C-33), 14.3 (C-19); HRMS *m/z* (ESI$^+$) found 761.5106 $[M+Na]^+$ ($C_{44}H_{70}O_7N_2Na$) requires 761.5075$[M+Na]^+$).

(*R*)-2-((3-(3-(Dodec-11-yn-1-yl)-3*H*-diazirin-3-yl)propanoyl)oxy)-3-((*R*)-2-methoxy-2-phenylacetoxy)propyl palmitate (–)-S26

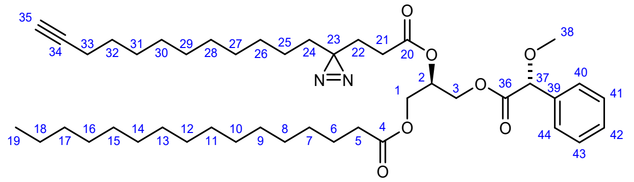

(*R*)-(–)-α-Methoxyphenylacetic acid (6 mg, 0.04 mmol, 2.2 eq), *N*,*N′*-dicyclohexylcarbodiimide (10 mg, 0.046 mmol, 2.7 eq) and 4-DMAP (1 mg, 0.008 mmol, 0.5 eq) were added to a solution of alcohol **32** (10 mg, 0.017 mmol, 1.0 eq) in CH_2Cl_2 (0.5 mL). After stirring at RT for 16 h, the reaction mixture was diluted with CH_2Cl_2 (5 mL), and the organic layer was washed with 1 M $HCl_{(aq)}$ (5 mL), a saturated aqueous solution of $NaHCO_3$ (5 mL), then brine (5 mL), and then dried over Na_2SO_4, filtered, and concentrated *in vacuo*. Purification using silica gel column chromatography (petroleum ether:Et_2O, 10:0 to 7:3) gave **(–)-S26** (10 mg, 82%) as a colorless film: R_f 0.63 (petroleum ether:Et_2O, 6:4); $[\alpha]_D^{25}$ –24.0 (*c* 1.0, $CHCl_3$); ν_{max} (thin film)/cm^{-1} 3312, 2928, 2855, 1749, 1466, 1171; ^{1}H NMR (600 MHz, $CDCl_3$) δ_H 7.44–7.32 (5 H, m, H-40 to H-44), 5.22–5.18 (1 H, m, H-2), 4.77 (1 H, s, H-37), 4.33 (1 H, dd, *J* 11.9, 4.0, H-3’), 4.19 (1 H, dd, *J* 11.5, 5.8, H-3’’), 4.16 (1 H, dd, *J* 11.5, 4.0, H-1’), 4.03 (1 H, dd, *J* 11.9, 5.8, H-1’’), 3.41 (3 H, s, H-38), 2.26 (2 H, t, *J* 7.6, H-5), 2.18 (2 H, td, *J* 7.2, 2.7, H-33), 2.00–1.88 (3 H, m, H-35 and H-21), 1.69–1.60 (2 H, m, H-22), 1.61–1.53 (2 H, m, H-6), 1.56–1.48 (2 H, m, H-32), 1.42–1.32 (4 H, m, H-24 and H-31), 1.30–1.19 (34 H, m, H-7 to H-18 and H-26 to H-30), 1.09–1.03 (2 H, m, H-25), 0.88 (3 H, t, *J* 7.0, H-19); ^{13}C NMR (151 MHz, $CDCl_3$) δ_C 173.3 (C-4), 171.4 (C-20), 170.4 (C-36), 136.1 (C-39), 129.1

(C-42), 128.9, 127.3 (C-40, C-41, C-43, C-44), 84.9 (C-34), 82.4 (C-37), 69.3 (C-2), 68.2 (C-35), 62.8 (C-3), 61.8 (C-1), 57.6 (C-38), 34.1 (C-5), 32.8 (C-24), 32.1, 29.9, 29.84, 29.81, 29.77, 29.62, 29.57, 29.5, 29.4, 29.32, 29.25, 29.21, 28.9, 28.6 (C-7 to C-17 and C-25 to C-32), 28.5 (C-21), 28.18 (C-23), 28.15 (C-22), 25.0 (C-6), 24.0 (C-29), 22.8 (C-18), 18.5 (C-33), 14.3 (C-19); HRMS *m/z* (ESI^+) found 761.5094 $[M+Na]^+$ ($C_{44}H_{70}O_7N_2Na$) requires 761.5075$[M+Na]^+$).

NMR Spectra and Other Data

1D-1-*O*-Acetate-2,3-*O*-cyclopentylidene-4,6-di-*O*-(4-methoxybenzyl)-5-*O*-(2-oxo-5.6-benzo-1,3,2-dioxaphosphep-2-yl)-*myo*-inositol (−)-18 ^{1}H NMR

Current Data Parameters
NAME gb653390802
EXPNO 1
PROCNO 1

F2 - Acquisition Parameters
Date_ 20220208
Time 21.07 h
INSTRUM Avance
PROBHD Z159656_0020 (
PULPROG zg30
TD 65536
SOLVENT CDCl3
NS 16
DS 2
SWH 11904.762 Hz
FIDRES 0.363304 Hz
AQ 2.7525120 sec
RG 84.3283
DW 42.000 usec
DE 22.00 usec
TE 298.0 K
D1 1.00000000 sec
TD0 1
SFO1 600.4230021 MHz
NUC1 1H
P0 4.00 usec
P1 12.00 usec
PLW1 13.51200008 W

F2 - Processing parameters
SI 65536
SF 600.4200137 MHz
WDW EM
SSB 0
LB 0.30 Hz
GB 0
PC 1.00

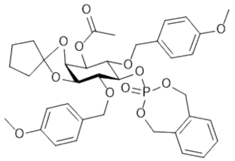

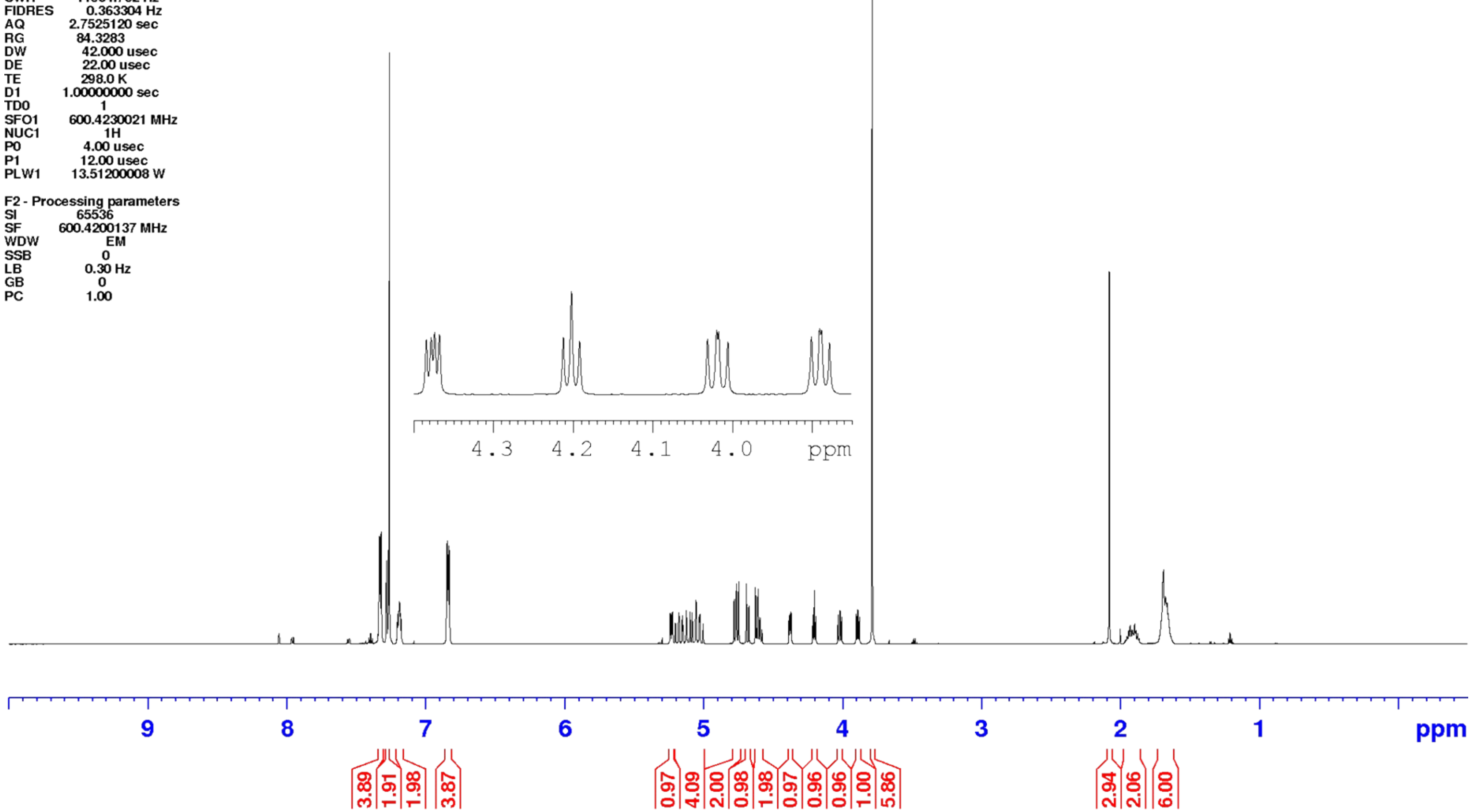

1D-1-*O*-Acetate-2,3-*O*-cyclopentylidene-4,6-di-*O*-(4-methoxybenzyl)-5-*O*-(2-oxo-5.6-benzo-1,3,2-dioxaphosphep-2-yl)-*myo*-inositol (−)-18 ^{31}P NMR

Current Data Parameters		
NAME	gb653390802	
EXPNO	6	
PROCNO	1	
F2 - Acquisition Parameters		
Date_	20220208	
Time	23.33	h
INSTRUM	Avance	
PROBHD	Z159656_0020 (	
PULPROG	zgpg30	
TD	65536	
SOLVENT	CDCl3	
NS	16	
DS	4	
SWH	48543.688	Hz
FIDRES	1.481436	Hz
AQ	0.6750208	sec
RG	101	
DW	10.300	usec
DE	18.00	usec
TE	298.0	K
D1	2.00000000	sec
D11	0.03000000	sec
TD0	1	
SFO1	243.0423184	MHz
NUC1	31P	
P0	4.00	usec
P1	12.00	usec
PLW1	39.40800095	W
SFO2	600.4224017	MHz
NUC2	1H	
CPDPRG[2	waltz16	
PCPD2	80.00	usec
PLW2	13.51200008	W
PLW12	0.30124050	W
PLW13	0.15098180	W
F2 - Processing parameters		
SI	32768	
SF	243.0544711	MHz
WDW	EM	
SSB	0	
LB	1.00	Hz
GB	0	
PC	1.40	

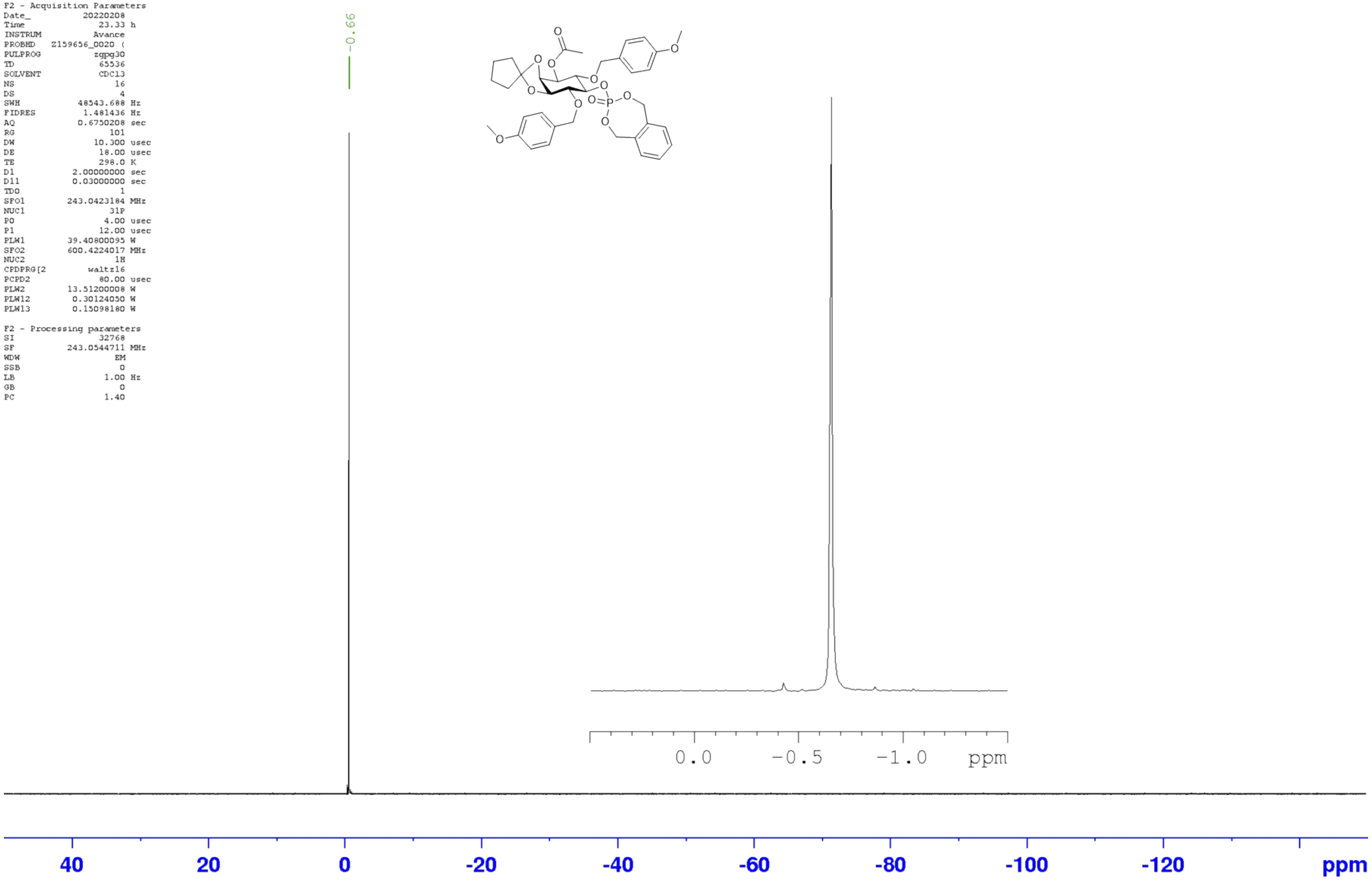

1D-1-*O*-Acetate-2,3-*O*-cyclopentylidene-4,6-di-*O*-(4-methoxybenzyl)-5-*O*-(2-oxo-5.6-benzo-1,3,2-dioxaphosphep-2-yl)-*myo*-inositol (−)-18 ^{13}C NMR

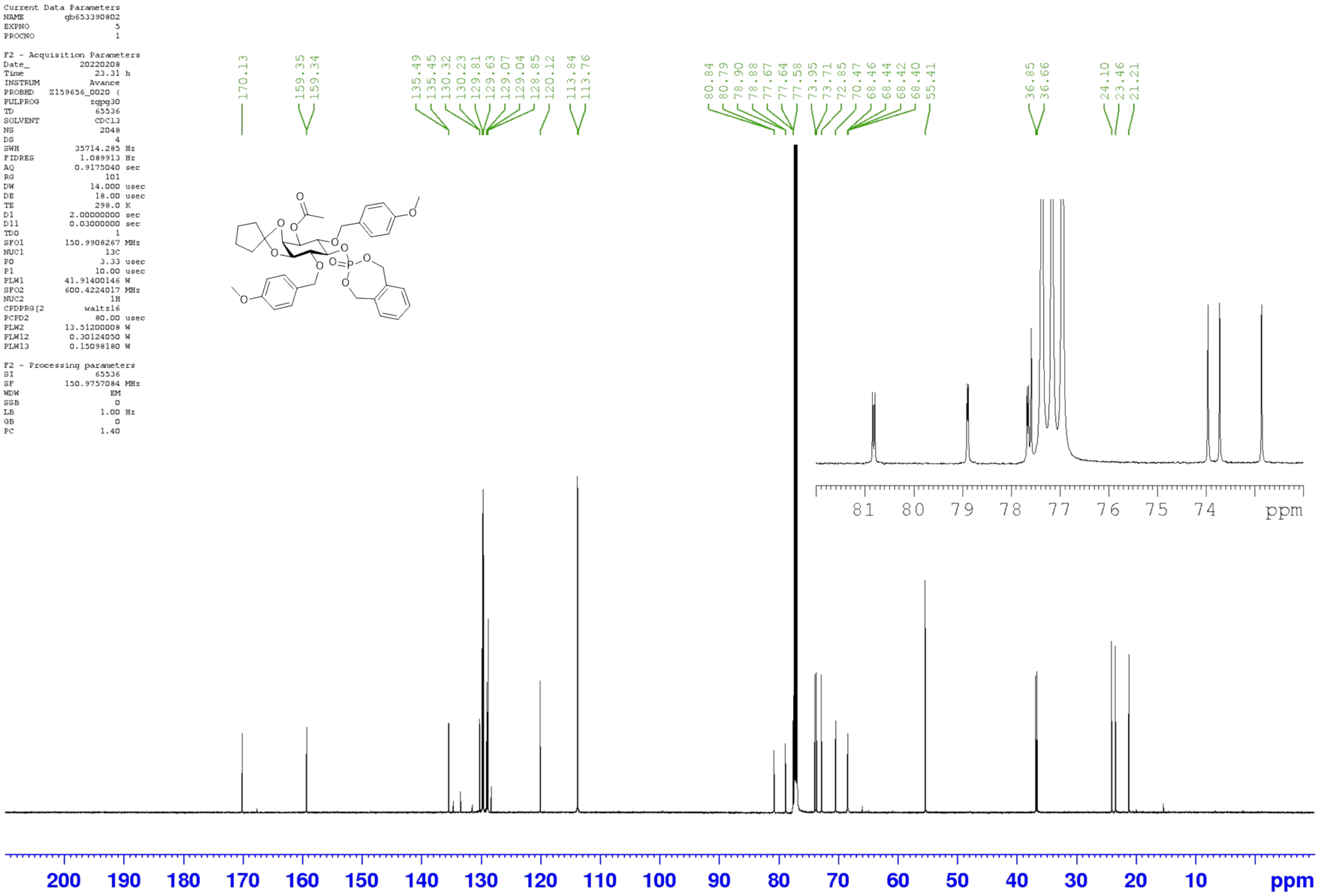

1D-1-*O*-Acetate-2,3-*O*-cyclopentylidene-4,6-di-*O*-(4-methoxybenzyl)-5-*O*-(2-oxo-5.6-benzo-1,3,2-dioxaphosphep-2-yl)-*myo*-inositol (−)-18 HRMS

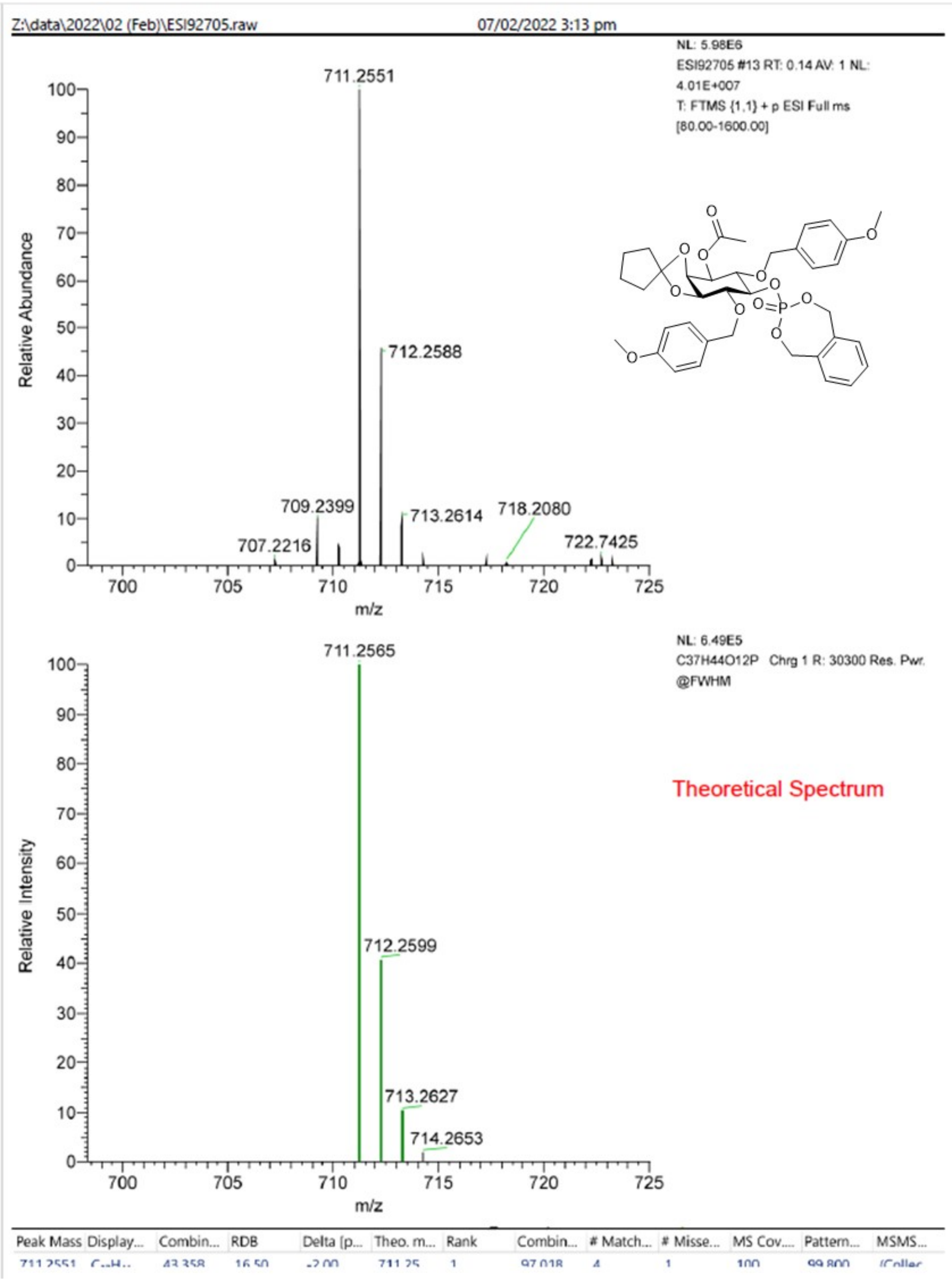

1D-2,3-*O*-Cyclopentylidene-4,6-di-*O*-(4-methoxybenzyl)-5-*O*-(2-oxo-5.6-benzo-1,3,2-dioxaphosphep-2-yl)-*myo*-inositol (+)-19 ^{1}H NMR

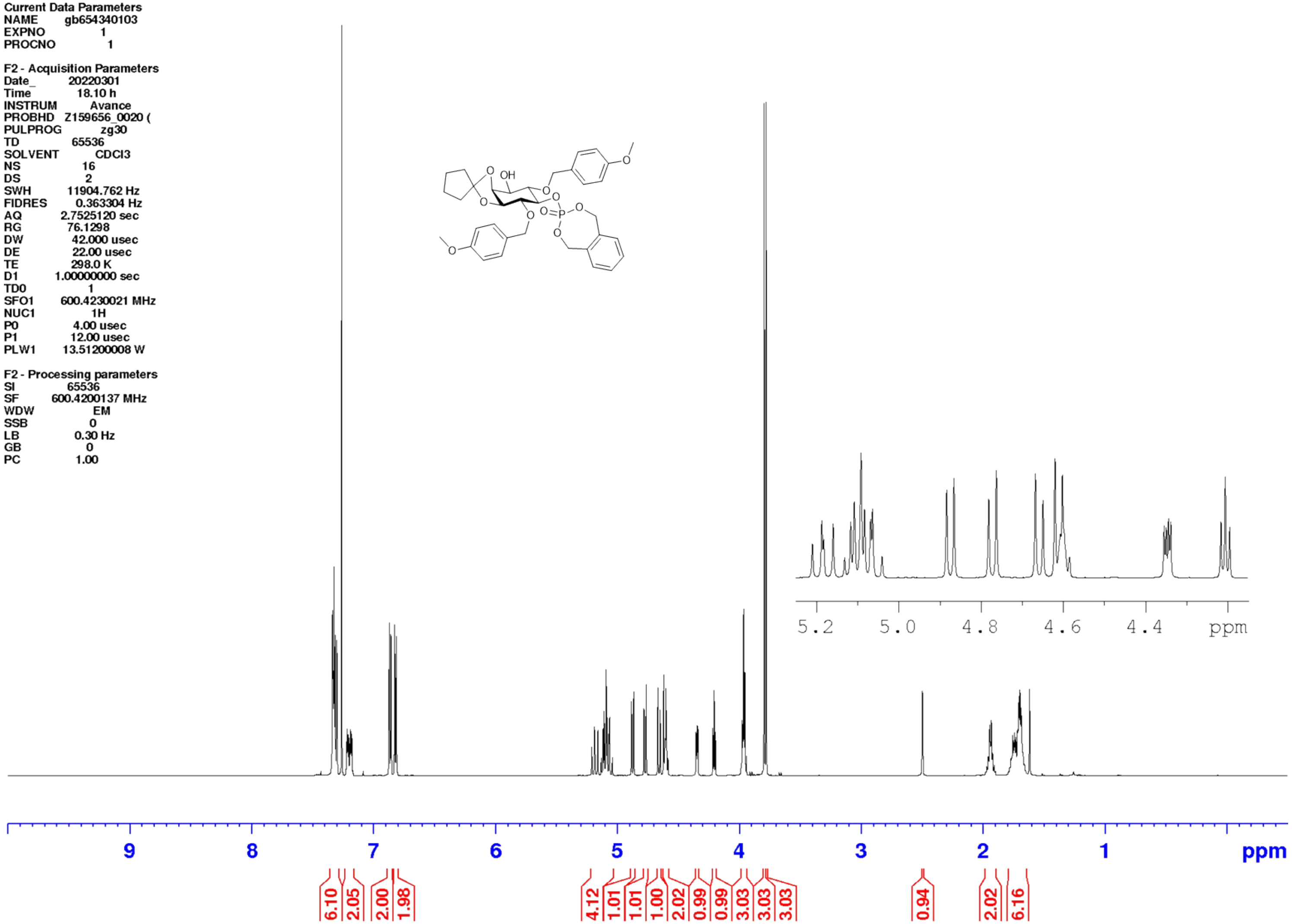

1D-2,3-*O*-Cyclopentylidene-4,6-di-*O*-(4-methoxybenzyl)-5-*O*-(2-oxo-5.6-benzo-1,3,2-dioxaphosphep-2-yl)-*myo*-inositol (+)-19 ^{31}P NMR

```
Current Data Parameters
NAME          gb654340103
EXPNO                   6
PROCNO                  1

F2 - Acquisition Parameters
Date_            20220301
Time                19.46 h
INSTRUM            Avance
PROBHD   Z159656_0020 (
PULPROG            zgpg30
TD                  65536
SOLVENT             CDCl3
NS                     16
DS                      4
SWH             48543.688 Hz
FIDRES           1.481436 Hz
AQ              0.6750208 sec
RG                    101
DW                 10.300 usec
DE                  18.00 usec
TE                  298.0 K
D1             2.00000000 sec
D11            0.03000000 sec
TD0                     1
SFO1          243.0423184 MHz
NUC1                  31P
P0                   4.00 usec
P1                  12.00 usec
PLW1          39.40800095 W
SFO2          600.4224017 MHz
NUC2                   1H
CPDPRG[2          waltz16
PCPD2               80.00 usec
PLW2          13.51200008 W
PLW12          0.30124050 W
PLW13          0.15098180 W

F2 - Processing parameters
SI                  32768
SF            243.0544711 MHz
WDW                    EM
SSB                     0
LB                   1.00 Hz
GB                      0
PC                   1.40
```

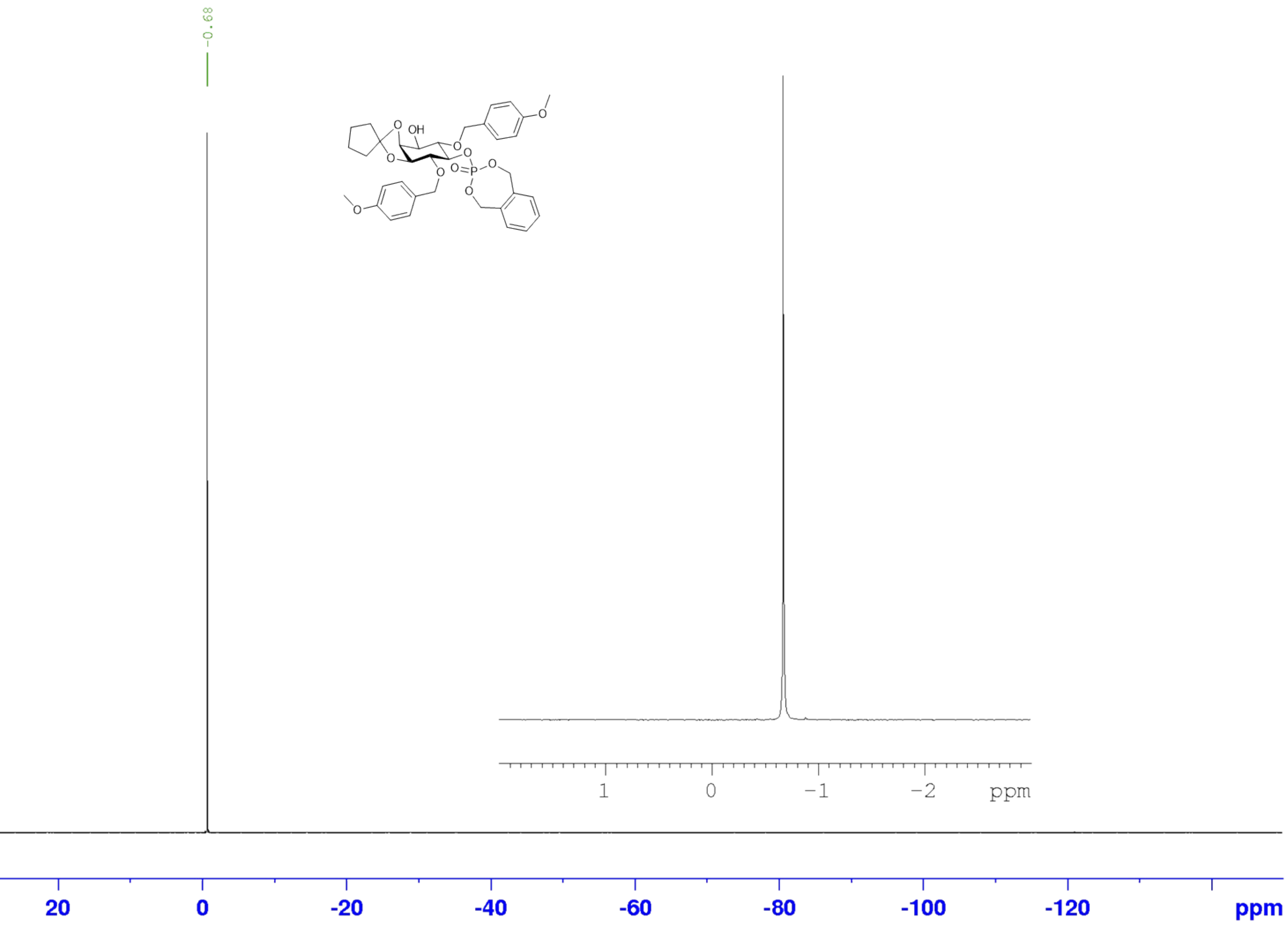

1D-2,3-*O*-Cyclopentylidene-4,6-di-*O*-(4-methoxybenzyl)-5-*O*-(2-oxo-5.6-benzo-1,3,2-dioxaphosphep-2-yl)-*myo*-inositol (+)-19 ^{13}C NMR

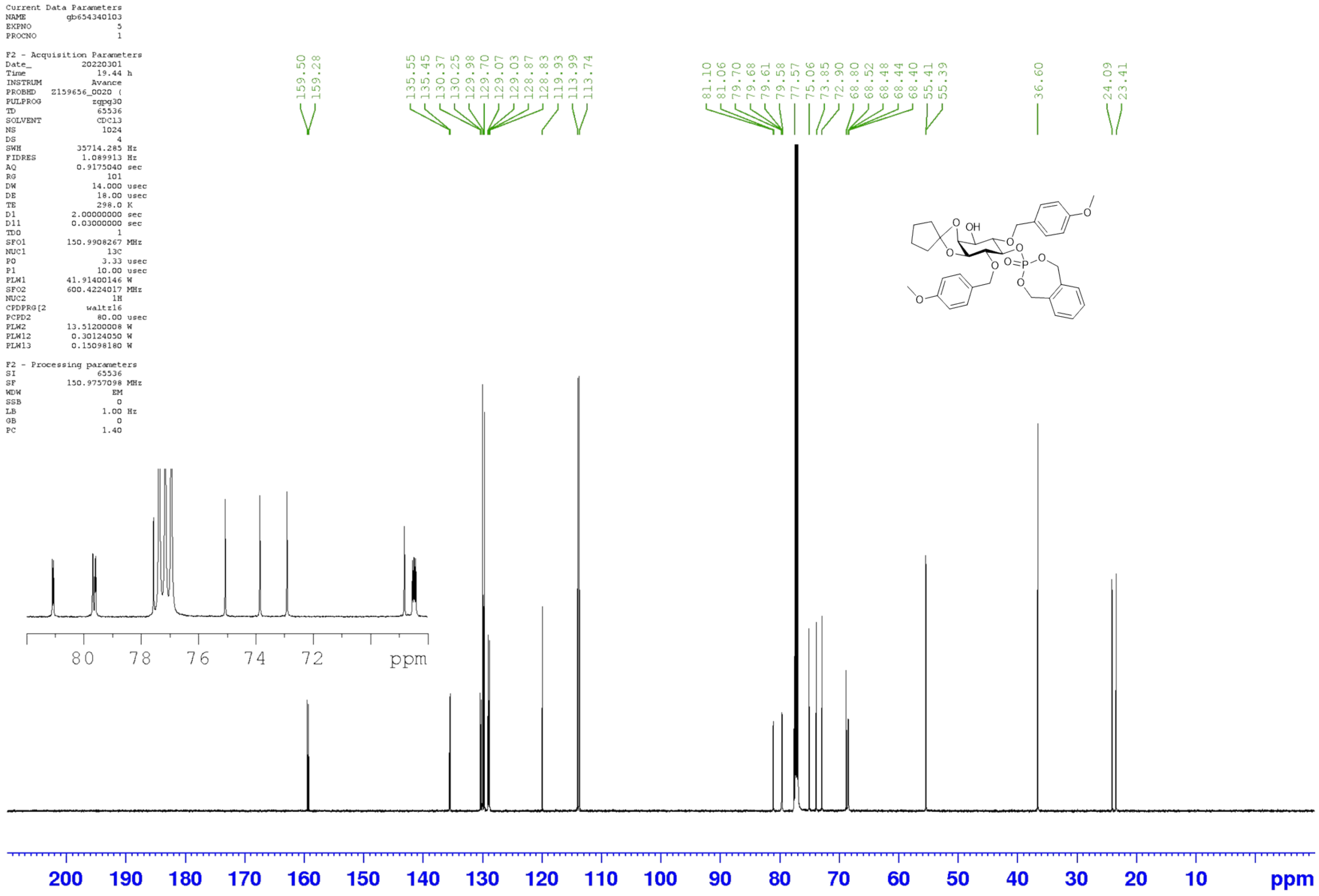

1D-2,3-*O*-Cyclopentylidene-4,6-di-*O*-(4-methoxybenzyl)-5-*O*-(2-oxo-5.6-benzo-1,3,2-dioxaphosphep-2-yl)-*myo*-inositol (+)-19 HRMS

Expanded Spectrum RT 0.10, NL 2842247, Peak [1], Target Mass 669.2459

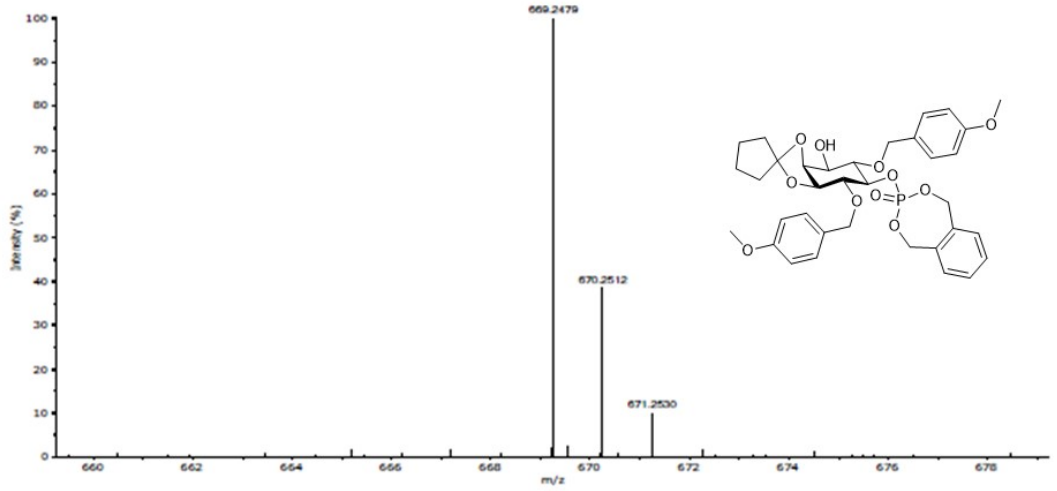

Theoretical Spectrum for C35H42O11P, Minimum Abundance 0.01%

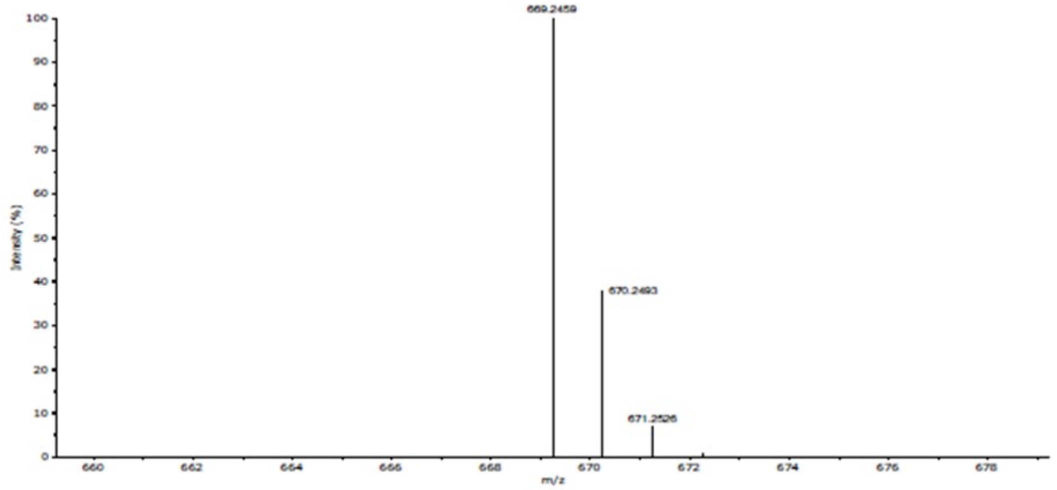

Measured Mass	Calculated Mass	Error (mDa)	Error (ppm)	Formula $[M+H]^+$	Response
669.2479	669.2459	1.97	2.94	$C_{35}H_{42}O_{11}P$	2534295

13-{(tetrahydropyran-2'-yl)oxy}tridecan-1-yne 22 ^{1}H NMR

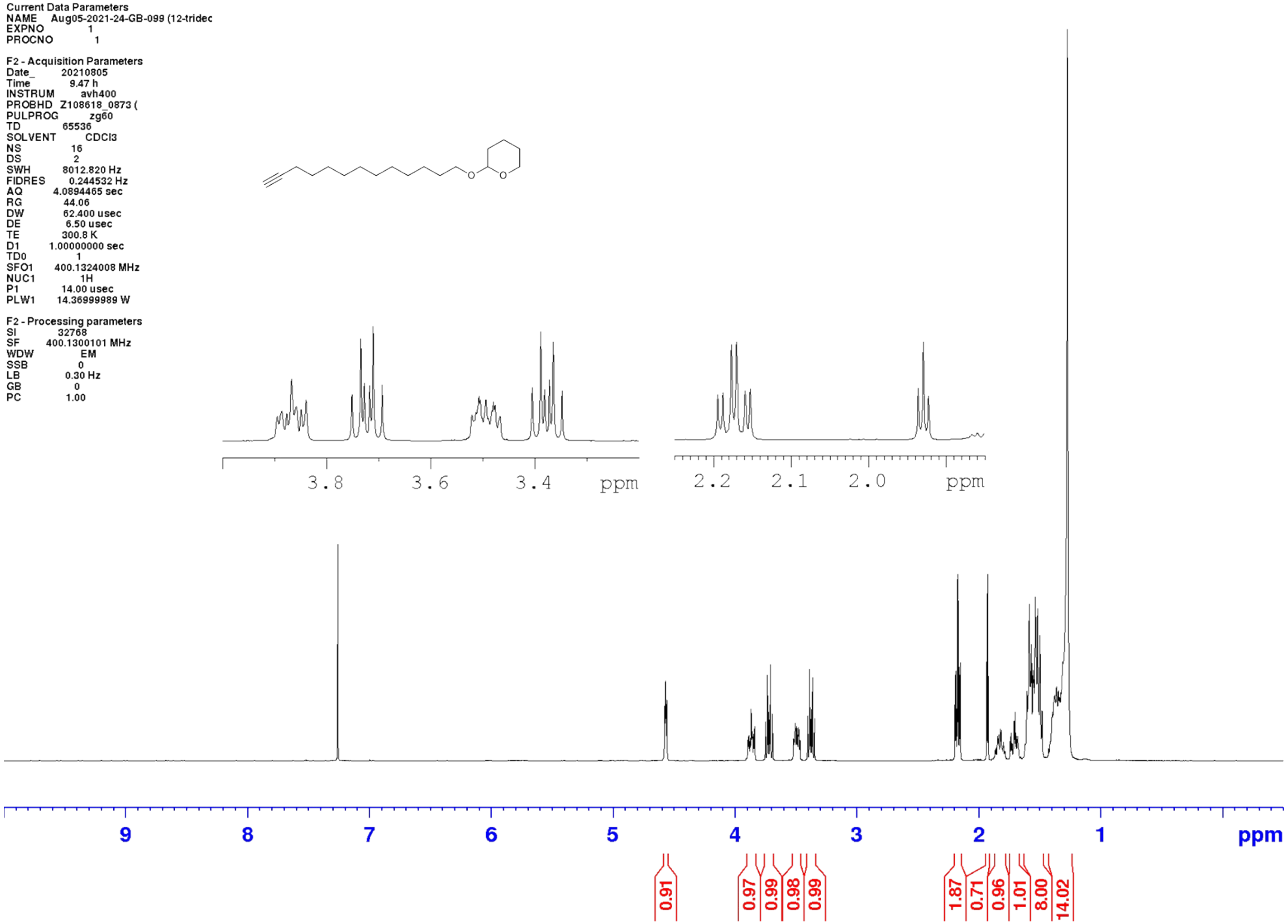

13-{(tetrahydropyran-2'-yl)oxy}tridecan-1-yne 22 ^{13}C NMR

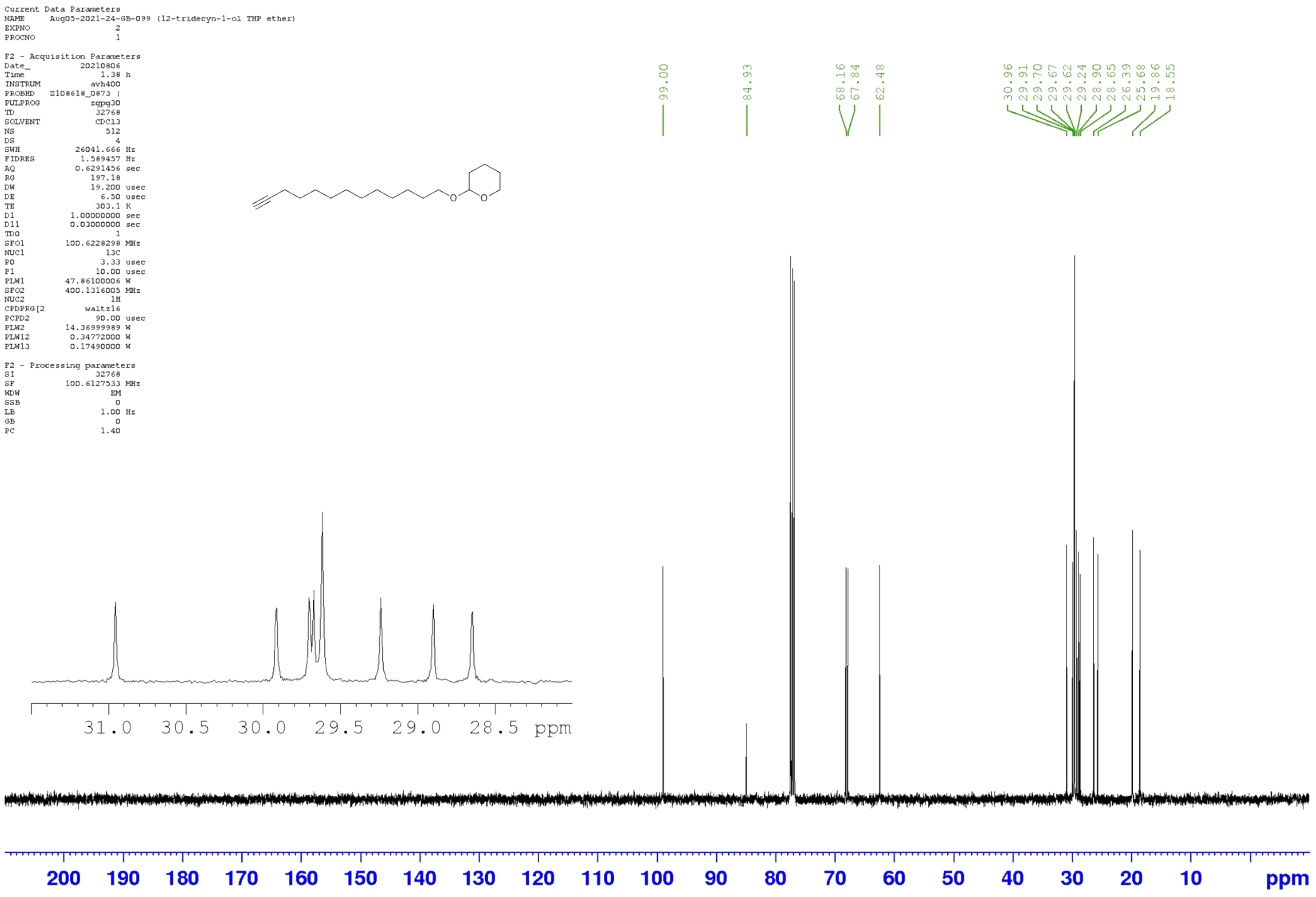

13-{(tetrahydropyran-2'-yl)oxy}tridecan-1-yne 22 HRMS

Expanded Spectrum RT 0.10, NL 760738, Peak [1], Target Mass 303.2295

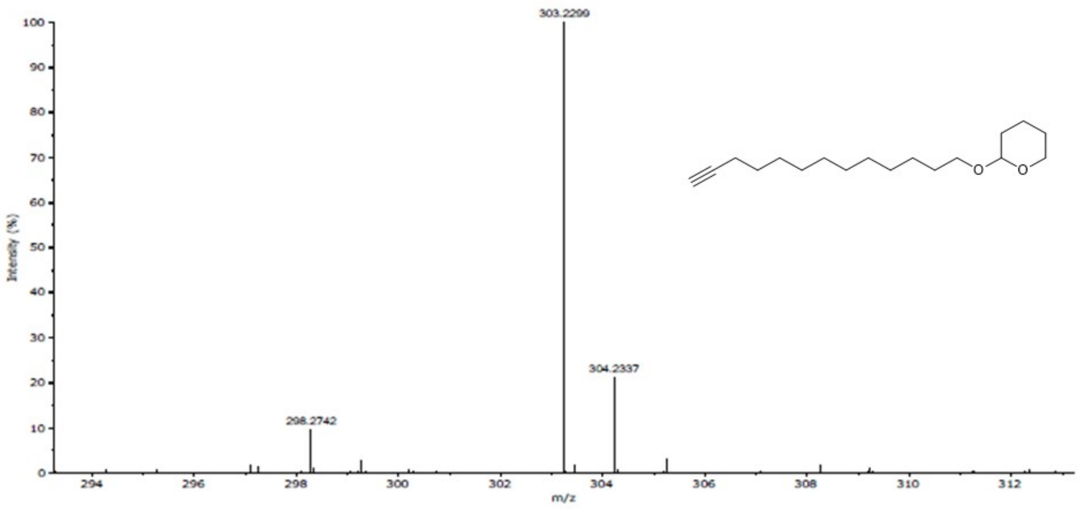

Theoretical Spectrum for C18H32O2Na, Minimum Abundance 0.01%

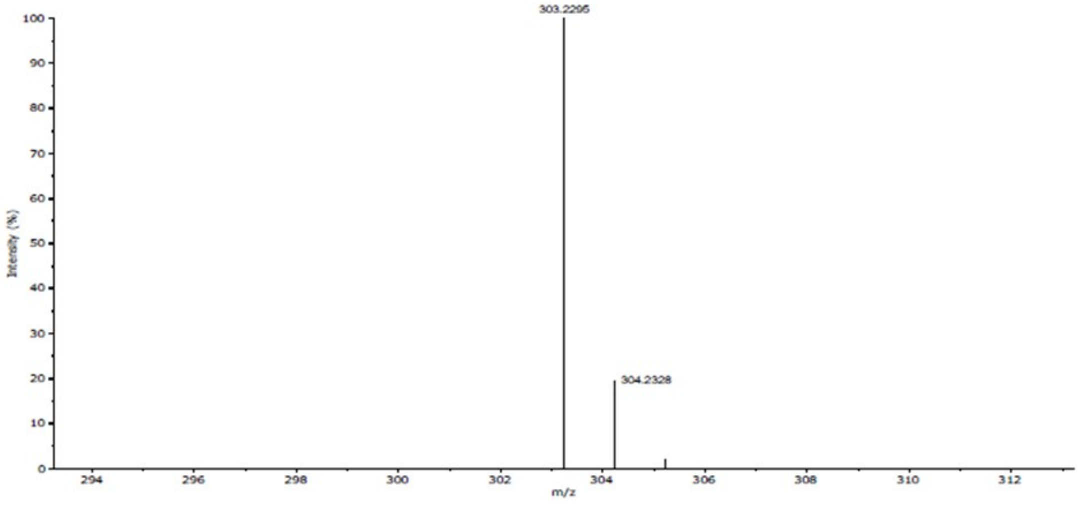

Measured Mass	Calculated Mass	Error (mDa)	Error (ppm)	Formula $[M+Na]^+$	Response
303.2299	303.2295	0.44	1.47	$C_{18}H_{32}O_2Na$	314088

ethyl 4-oxohexadec-15-ynoate 25 ^{1}H NMR

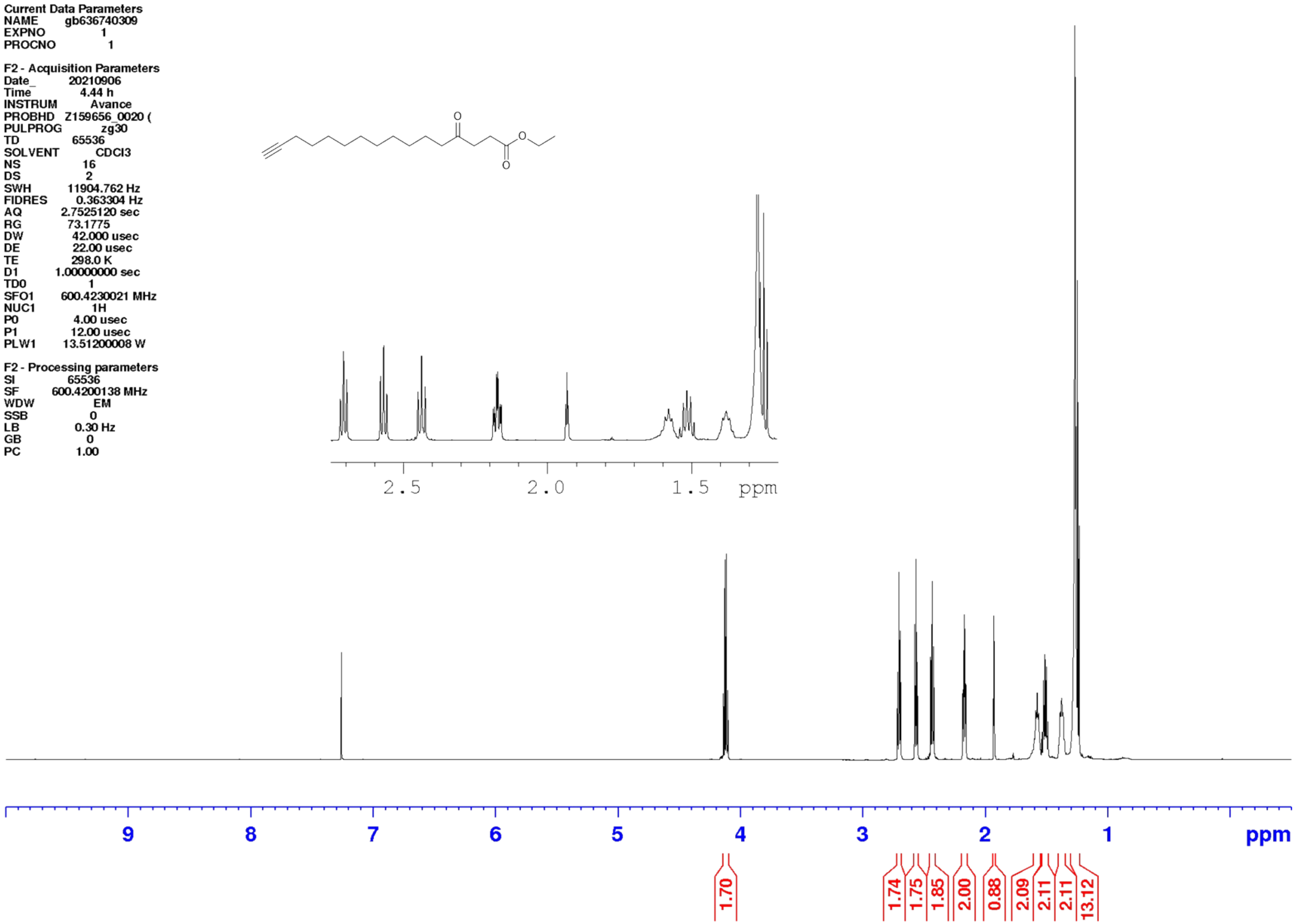

ethyl 4-oxohexadec-15-ynoate 25 ^{13}C NMR

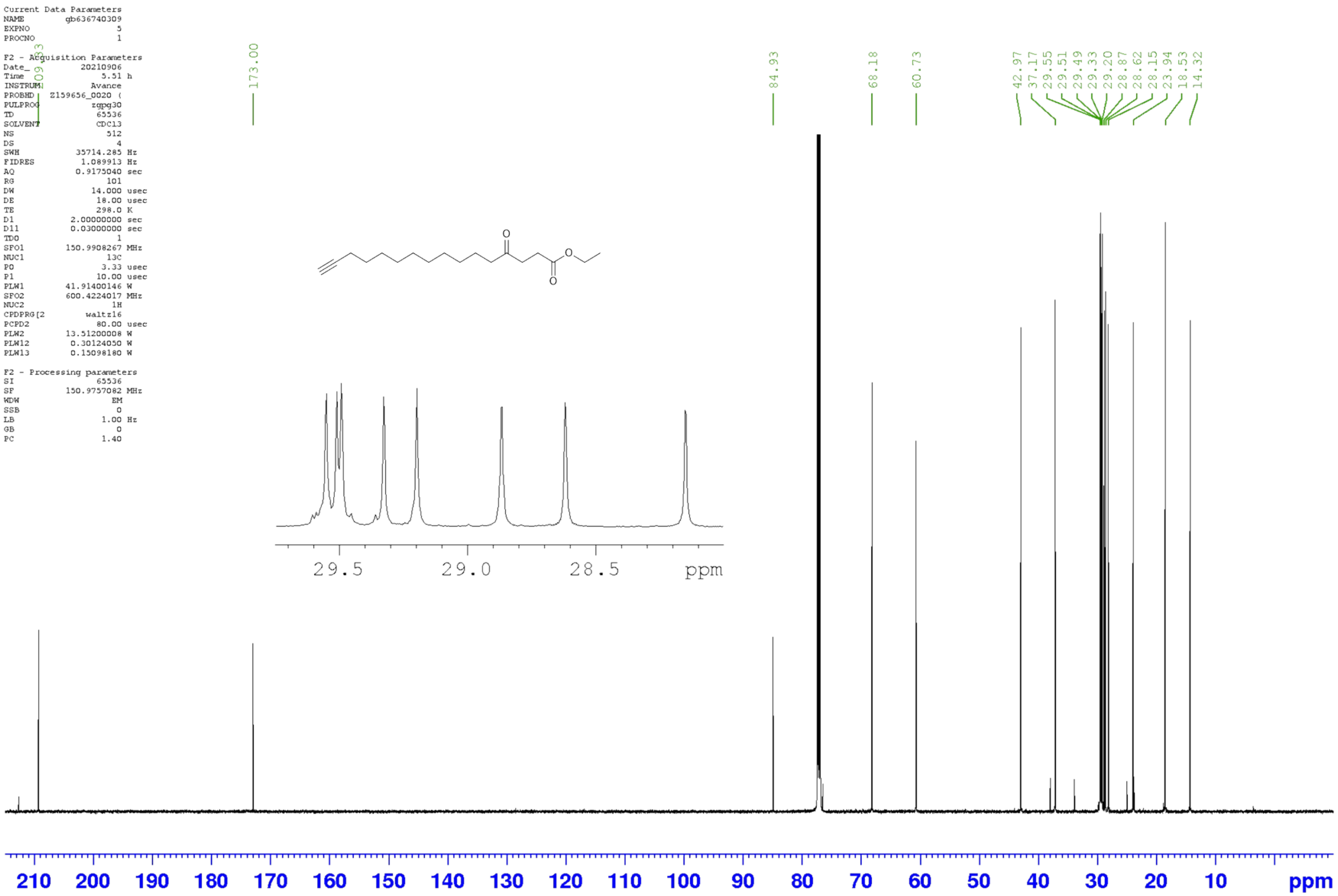

ethyl 4-oxohexadec-15-ynoate 25 HMRS

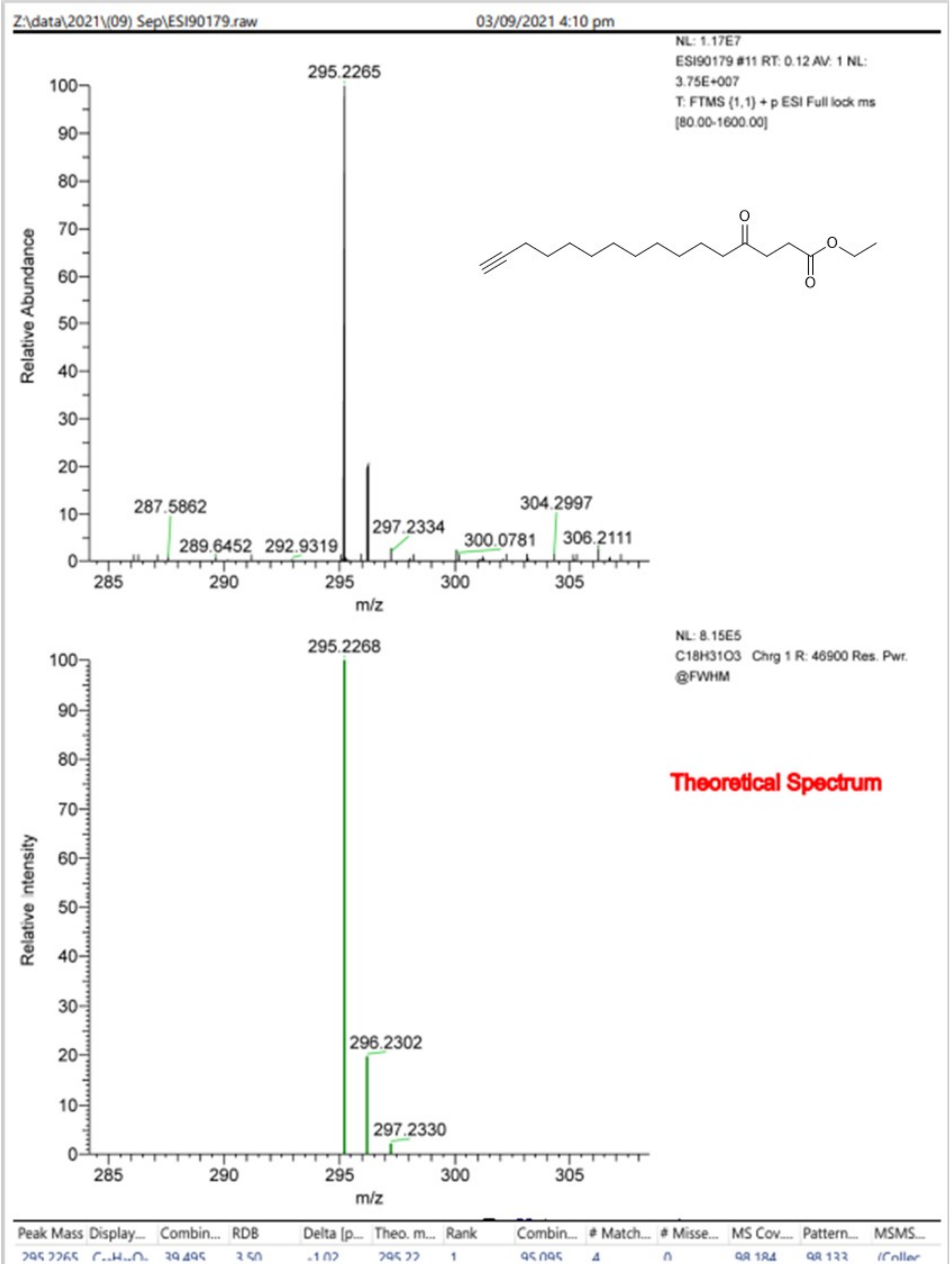

4-oxohexadec-15-ynoic acid 26 ^{1}H NMR

Current Data Parameters
NAME gb636950609
EXPNO 1
PROCNO 1

F2 - Acquisition Parameters
Date_ 20210907
Time 5.20 h
INSTRUM Avance
PROBHD Z159656_0020 (
PULPROG zg30
TD 65536
SOLVENT CDCl3
NS 16
DS 2
SWH 11904.762 Hz
FIDRES 0.363304 Hz
AQ 2.7525120 sec
RG 77.4444
DW 42.000 usec
DE 22.00 usec
TE 298.0 K
D1 1.00000000 sec
TD0 1
SFO1 600.4230021 MHz
NUC1 1H
P0 4.00 usec
P1 12.00 usec
PLW1 13.51200008 W

F2 - Processing parameters
SI 65536
SF 600.4200145 MHz
WDW EM
SSB 0
LB 0.30 Hz
GB 0
PC 1.00

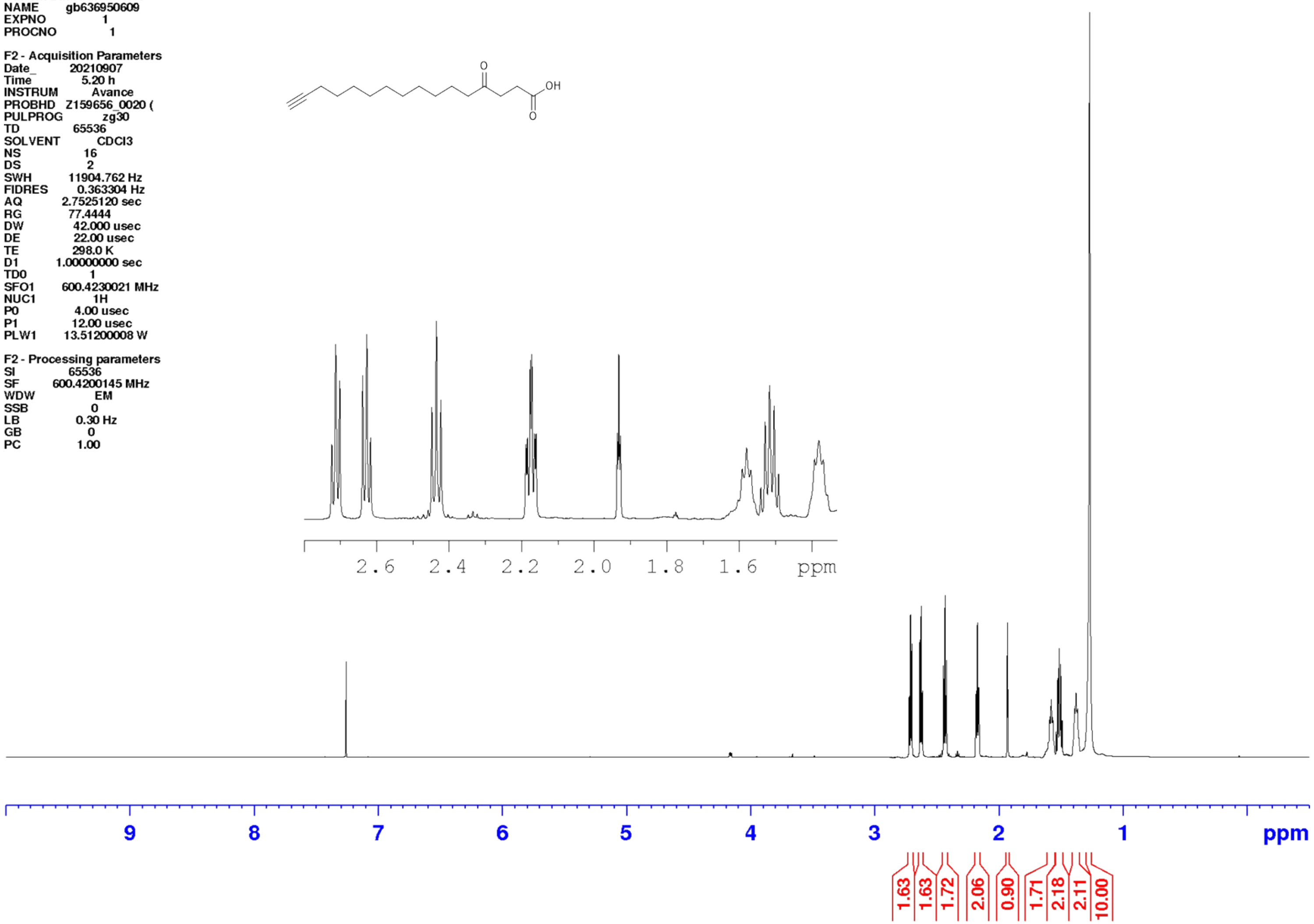

4-oxohexadec-15-ynoic acid 26 ^{13}C NMR

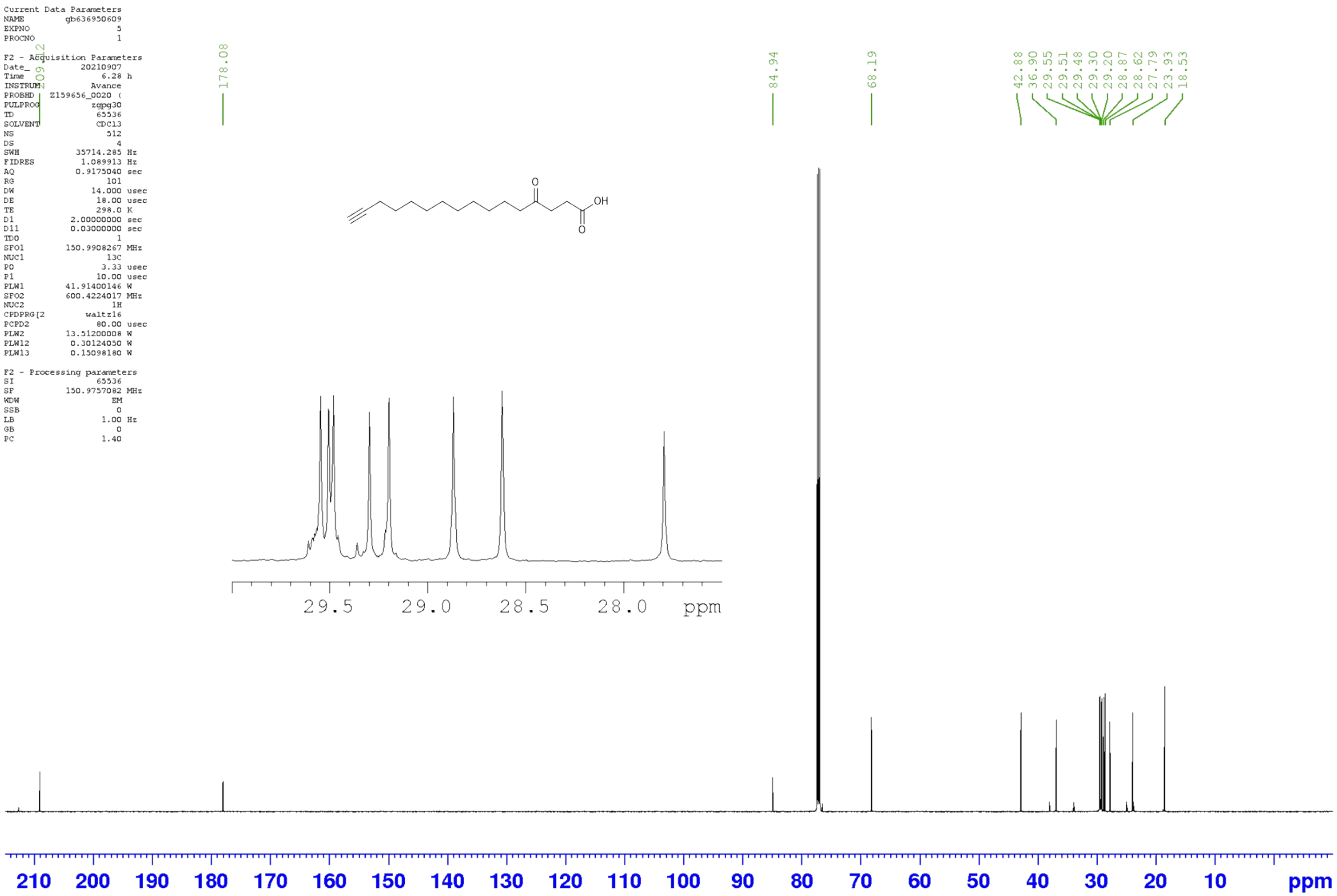

4-oxohexadec-15-ynoic acid 26 HRMS

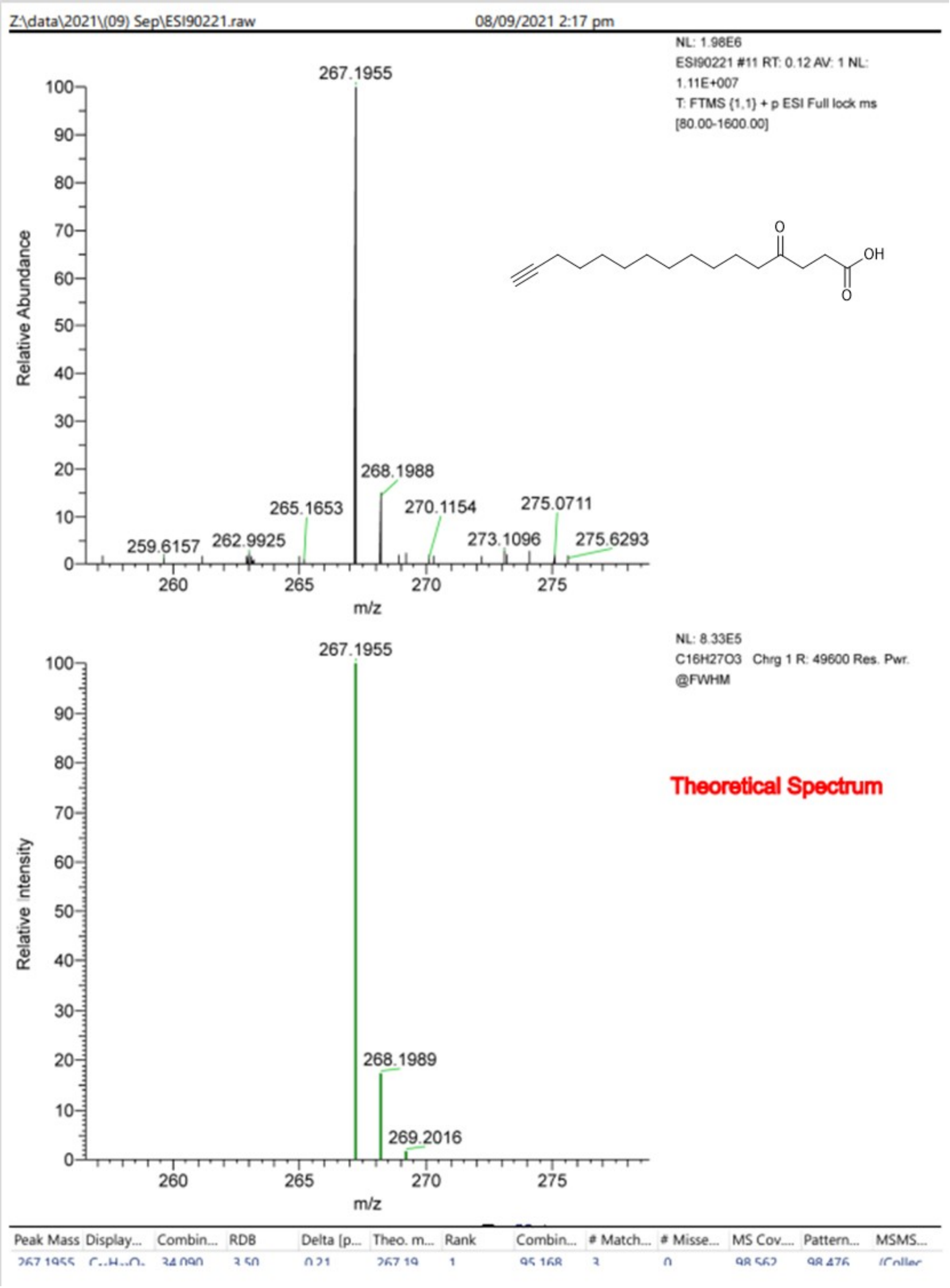

3-[3-(dodec-11-yn-1-yl)diazirin-3-yl]propanoic acid 27 ^{1}H NMR

Current Data Parameters
NAME gb664210305 (C16 Diaz Acid)
EXPNO 1
PROCNO 1

F2 - Acquisition Parameters
Date_ 20220504
Time 1.15 h
INSTRUM Avance
PROBHD Z159656_0020 (
PULPROG zg30
TD 65536
SOLVENT CDCl3
NS 16
DS 2
SWH 11904.762 Hz
FIDRES 0.363304 Hz
AQ 2.7525120 sec
RG 75.0793
DW 42.000 usec
DE 22.00 usec
TE 298.0 K
D1 1.00000000 sec
TD0 1
SFO1 600.4230021 MHz
NUC1 1H
P0 4.00 usec
P1 12.00 usec
PLW1 13.51200008 W

F2 - Processing parameters
SI 65536
SF 600.4200145 MHz
WDW EM
SSB 0
LB 0.30 Hz
GB 0
PC 1.00

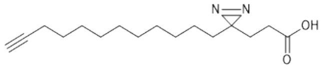

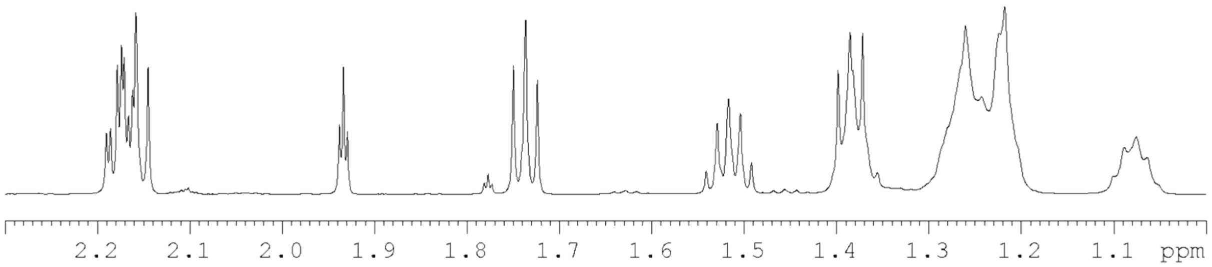

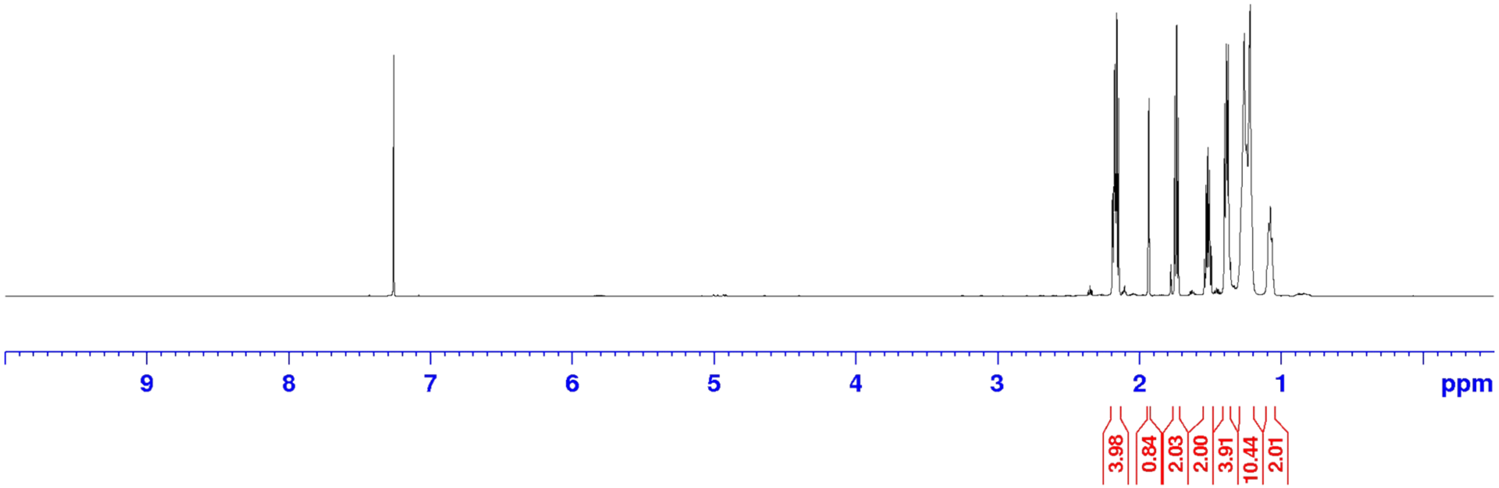

3-[3-(dodec-11-yn-1-yl)diazirin-3-yl]propanoic acid 27 ^{13}C NMR

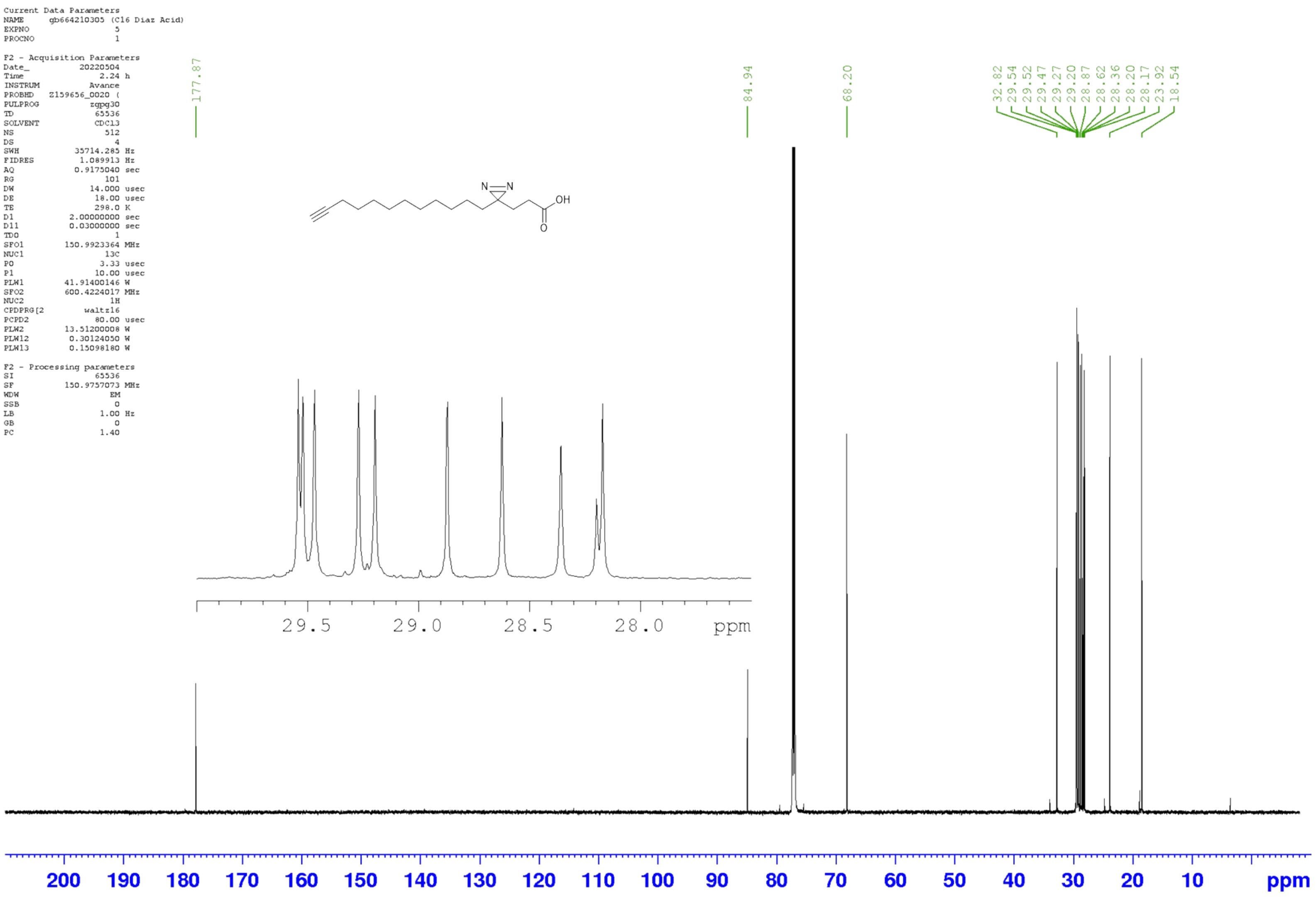

3-[3-(dodec-11-yn-1-yl)diazirin-3-yl]propanoic acid 27 HRMS

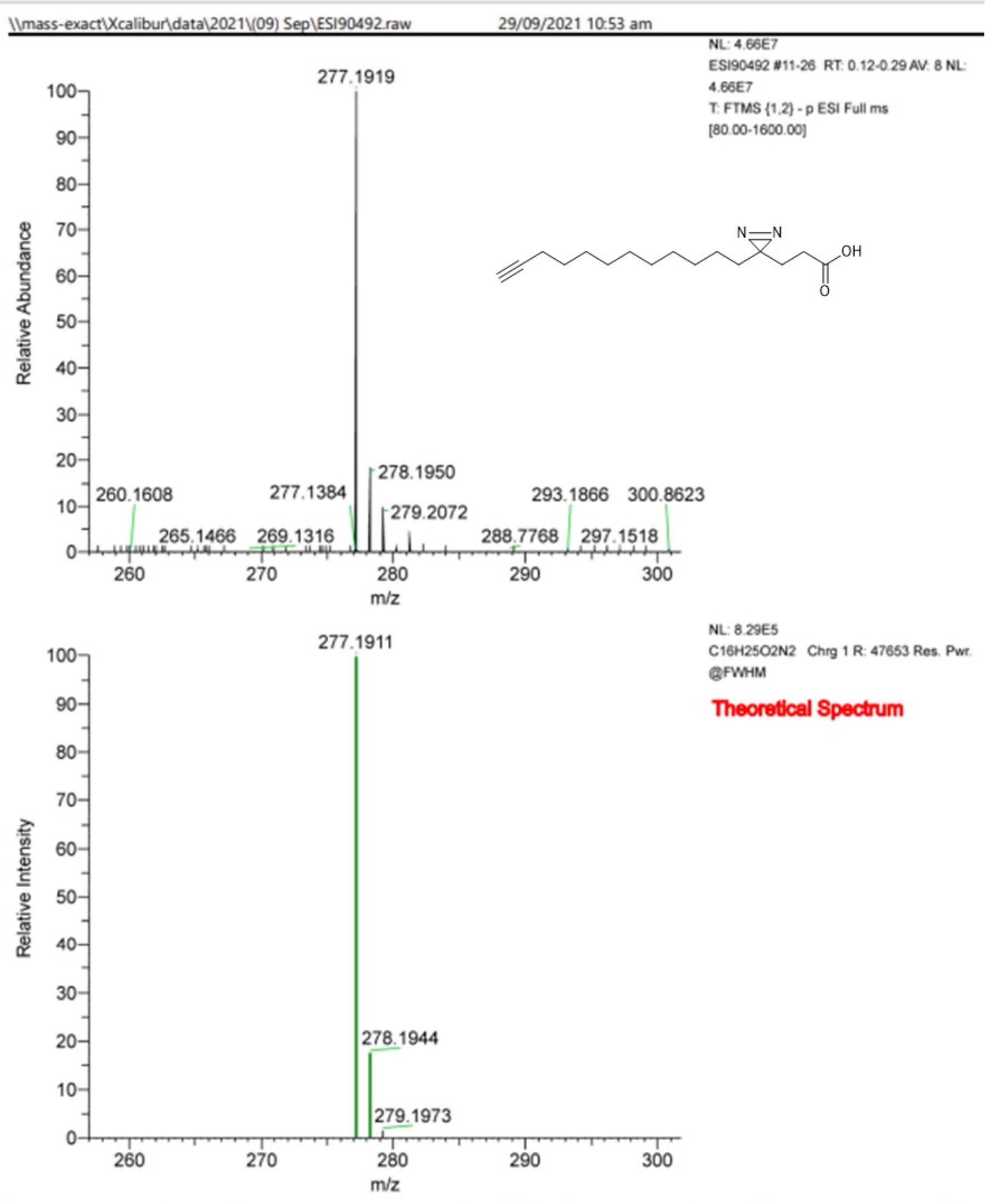

Peak Mass	Display...	Delta [p...	Combin...	RDB	Theo. m...	Rank	Combin...	# Match...	# Misse...	MS Cov....	Pattern...	MSMS...
277.1919	$C_{16}H_{25}$...	2.90	23.059...	5.50	277.19...	1	84.796...	4	0	88.226...	98.455...	(Collec...

(+)-(*R*)-2-((3-(3-(dodec-11-yn-1-yl)-3H-diazirin-3-yl)propanoyl)oxy)-3-((triethylsilyl)oxy)propyl palmitate 31 ^{1}H NMR

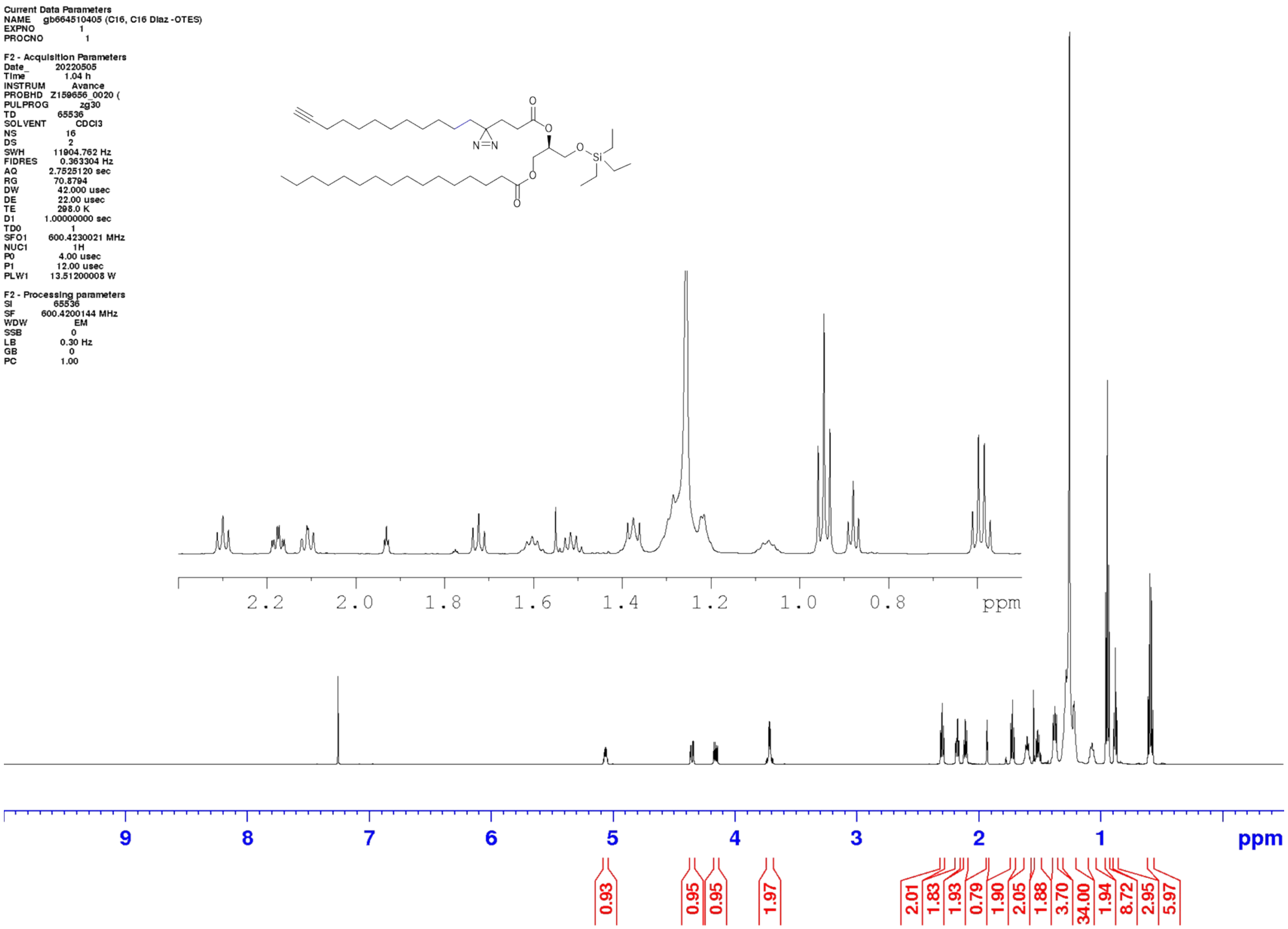

(+)-(*R*)-2-((3-(3-(dodec-11-yn-1-yl)-3H-diazirin-3-yl)propanoyl)oxy)-3-((triethylsilyl)oxy)propyl palmitate 31 ^{13}C NMR

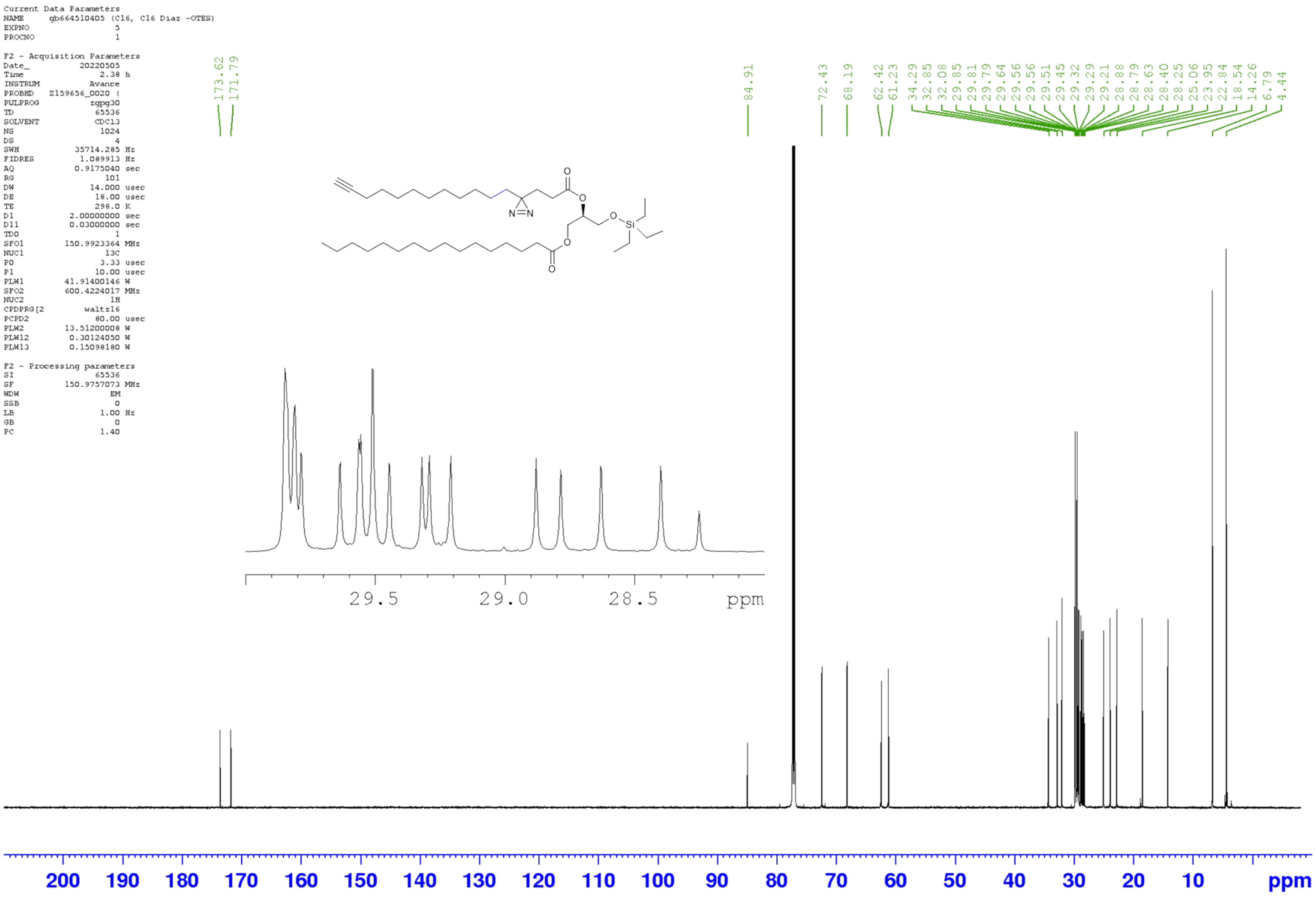

(+)-(*R*)-2-((3-(3-(dodec-11-yn-1-yl)-3H-diazirin-3-yl)propanoyl)oxy)-3-((triethylsilyl)oxy)propyl palmitate 31 HRMS

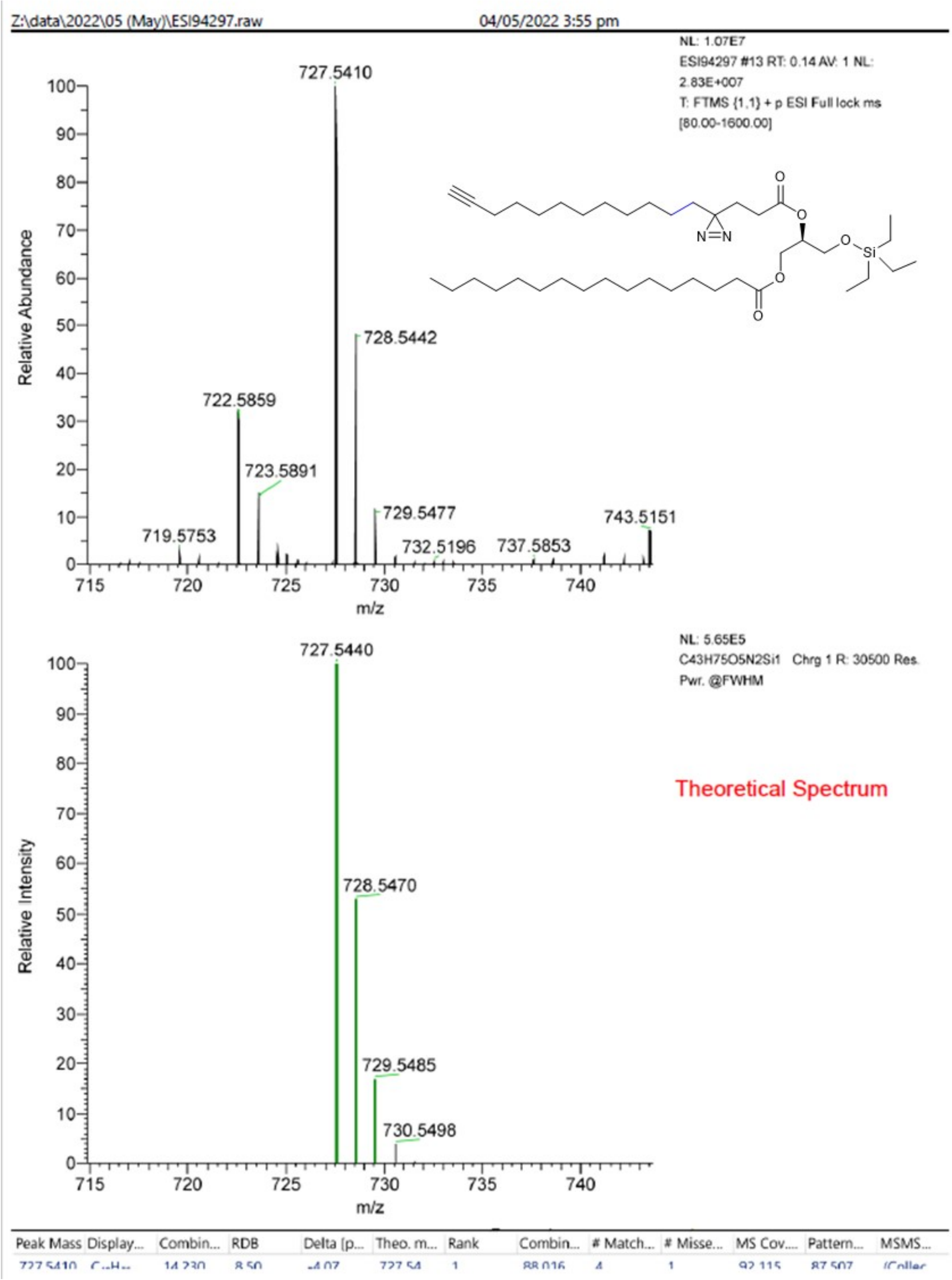

(*S*)-2-((3-(3-(dodec-11-yn-1-yl)-3H-diazirin-3-yl)propanoyl)oxy)-3-hydroxypropyl palmitate 32 ^{1}H NMR

Current Data Parameters
NAME gb665341005 (C16, C16 Diaz -OH)
EXPNO 1
PROCNO 1

F2 - Acquisition Parameters
Date_ 20220511
Time 13.23 h
INSTRUM Avance
PROBHD Z159656_0020 (
PULPROG zg30
TD 65536
SOLVENT CDCl3
NS 16
DS 2
SWH 11904.762 Hz
FIDRES 0.363304 Hz
AQ 2.7525120 sec
RG 74.9728
DW 42.000 usec
DE 22.00 usec
TE 298.0 K
D1 1.00000000 sec
TD0 1
SFO1 600.4230021 MHz
NUC1 1H
P0 4.00 usec
P1 12.00 usec
PLW1 13.51200008 W

F2 - Processing parameters
SI 65536
SF 600.4200139 MHz
WDW EM
SSB 0
LB 0.30 Hz
GB 0
PC 1.00

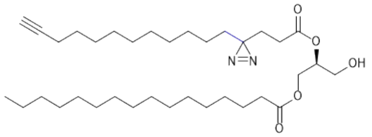

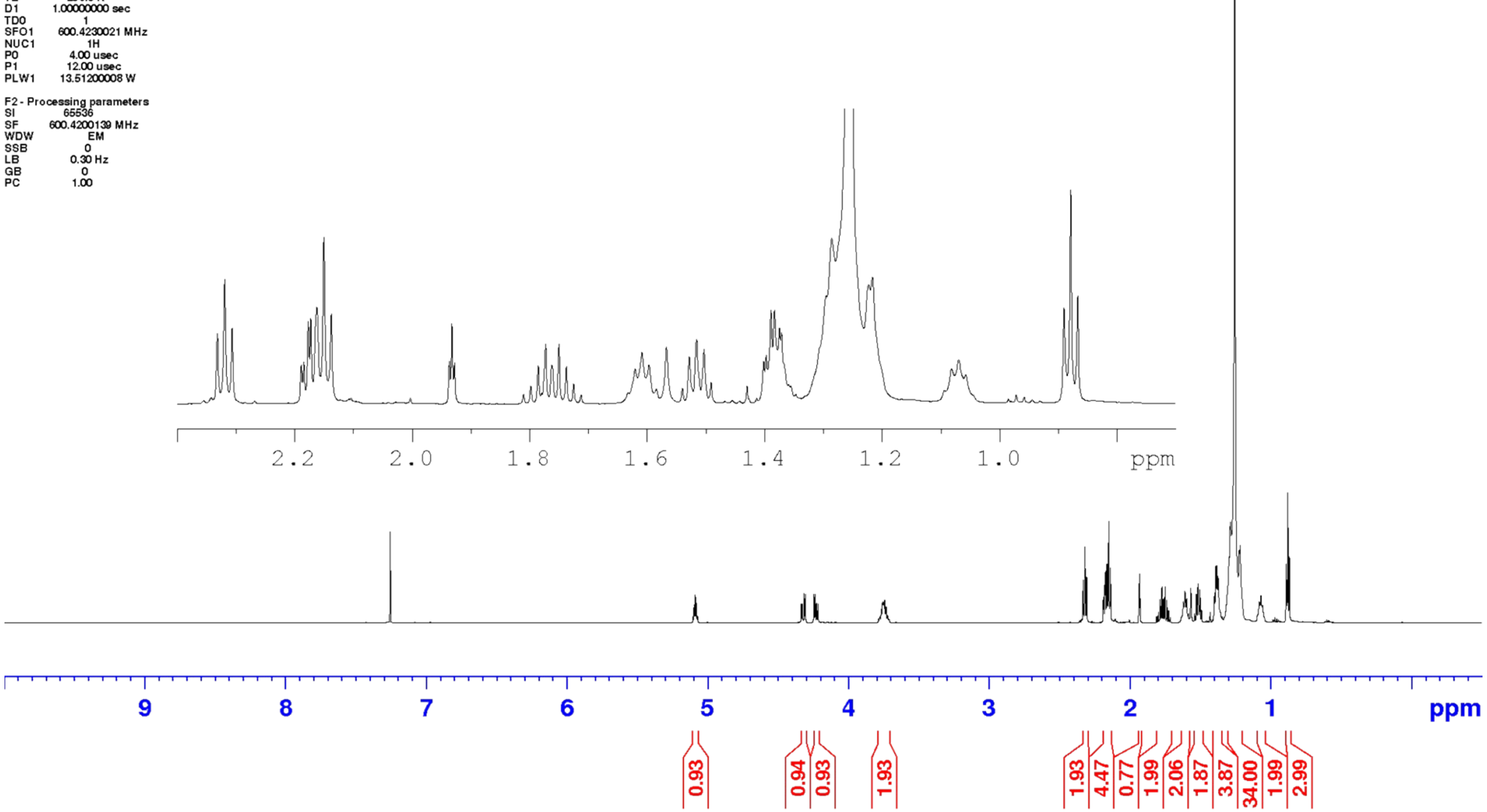

(*S*)-2-((3-(3-(dodec-11-yn-1-yl)-3H-diazirin-3-yl)propanoyl)oxy)-3-hydroxypropyl palmitate 32 ^{13}C NMR

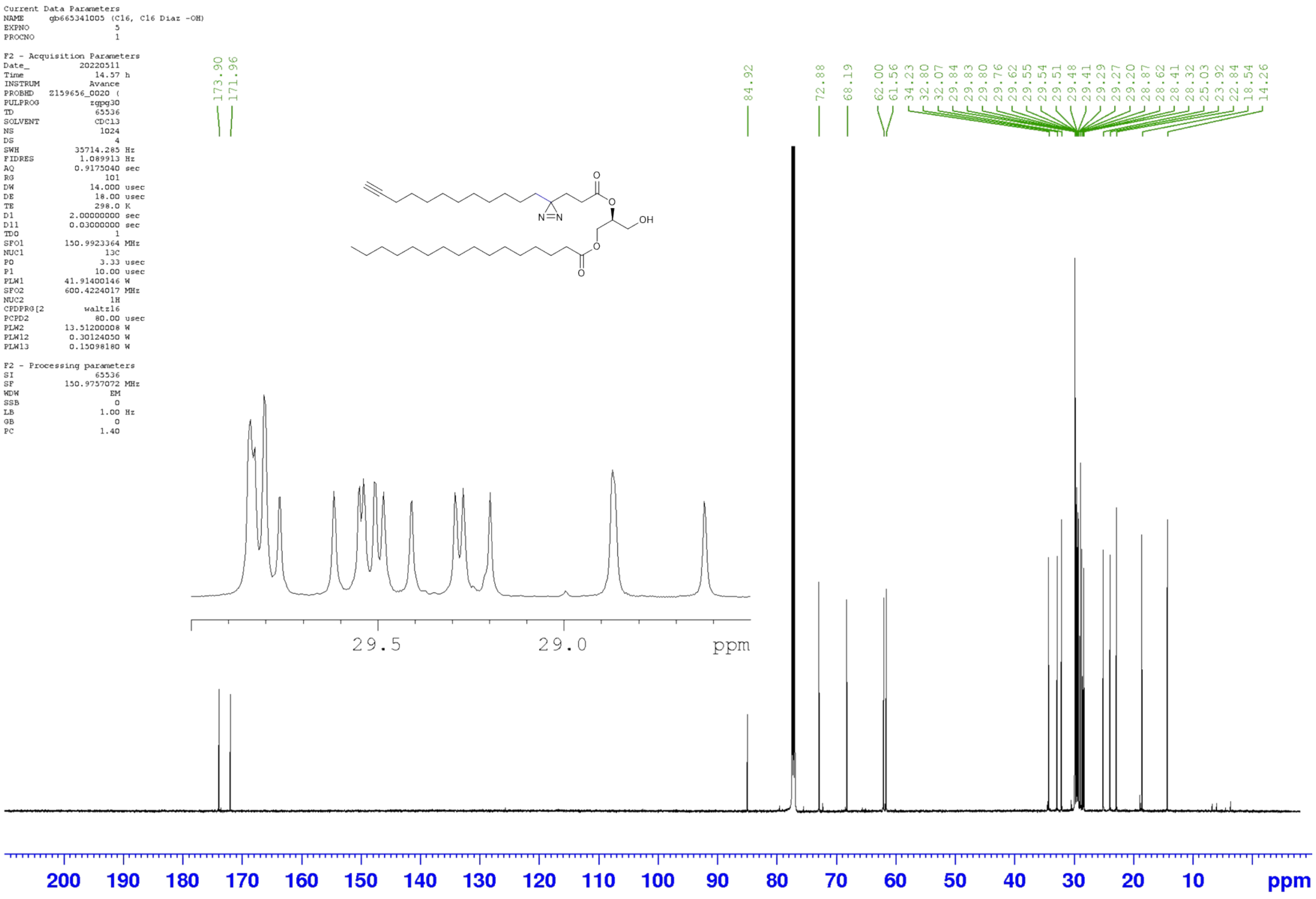

(*S*)-2-((3-(3-(dodec-11-yn-1-yl)-3H-diazirin-3-yl)propanoyl)oxy)-3-hydroxypropyl palmitate 32

HRMS

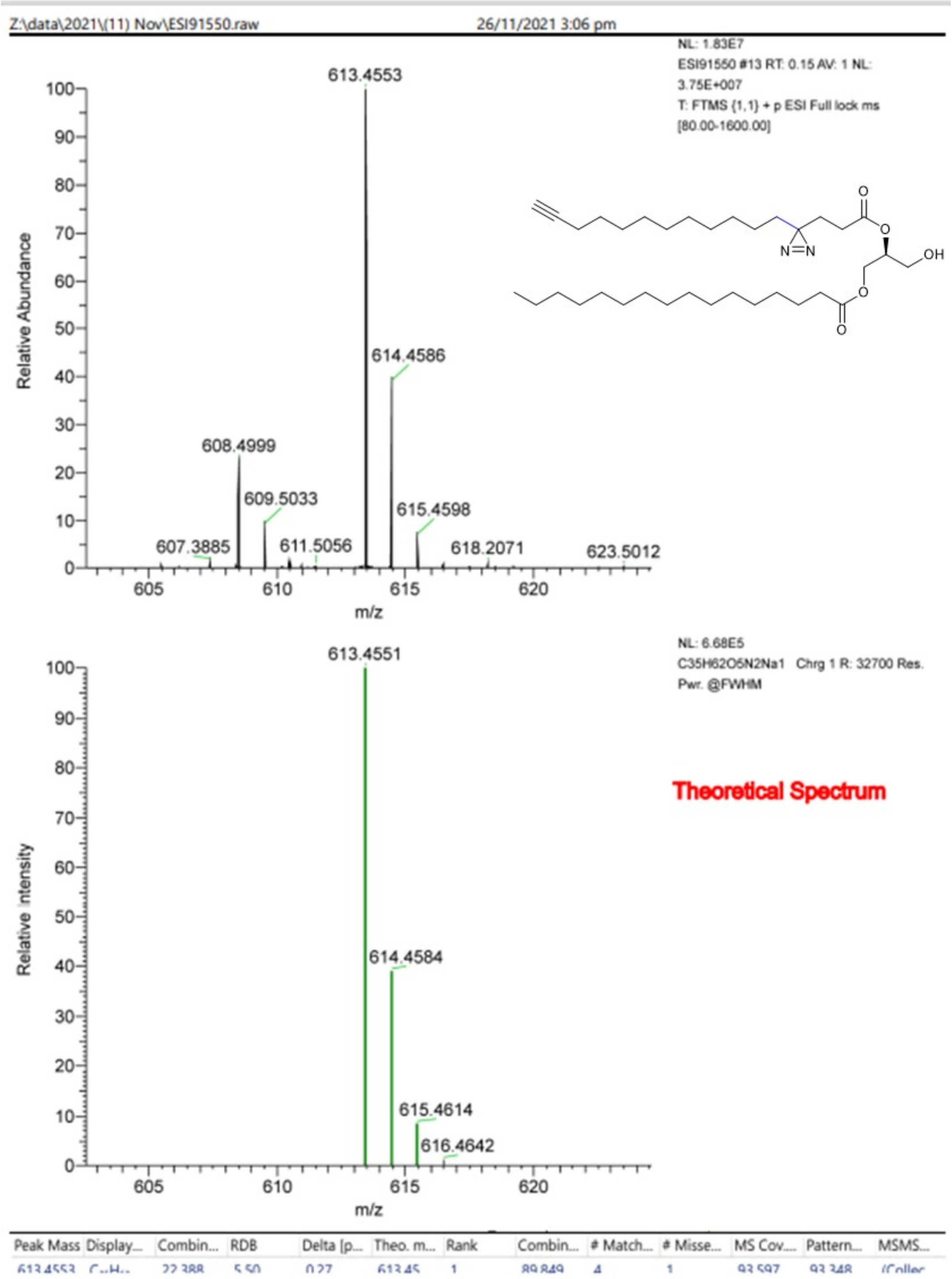

(2*R*)-3-(((benzyloxy)(diisopropylamino)phosphaneyl)oxy)-2-((3-(3-(dodec-11-yn-1-yl)-3H-diazirin-3-yl)propanoyl)oxy)propyl palmitate 34 ^{1}H NMR

Current Data Parameters
NAME Dec08-2021-22-GB-161 C16 Diaz
EXPNO 1
PROCNO 1

F2 - Acquisition Parameters
Date_ 20211208
Time 15.30 h
INSTRUM avh400
PROBHD Z108618_0873 (
PULPROG zg60
TD 65536
SOLVENT CDCl3
NS 16
DS 2
SWH 8012.820 Hz
FIDRES 0.244532 Hz
AQ 4.0894465 sec
RG 88.17
DW 62.400 usec
DE 6.50 usec
TE 298.5 K
D1 1.00000000 sec
TD0 1
SFO1 400.1324008 MHz
NUC1 1H
P1 14.00 usec
PLW1 14.36999989 W

F2 - Processing parameters
SI 32768
SF 400.1300100 MHz
WDW EM
SSB 0
LB 0.30 Hz
GB 0
PC 1.00

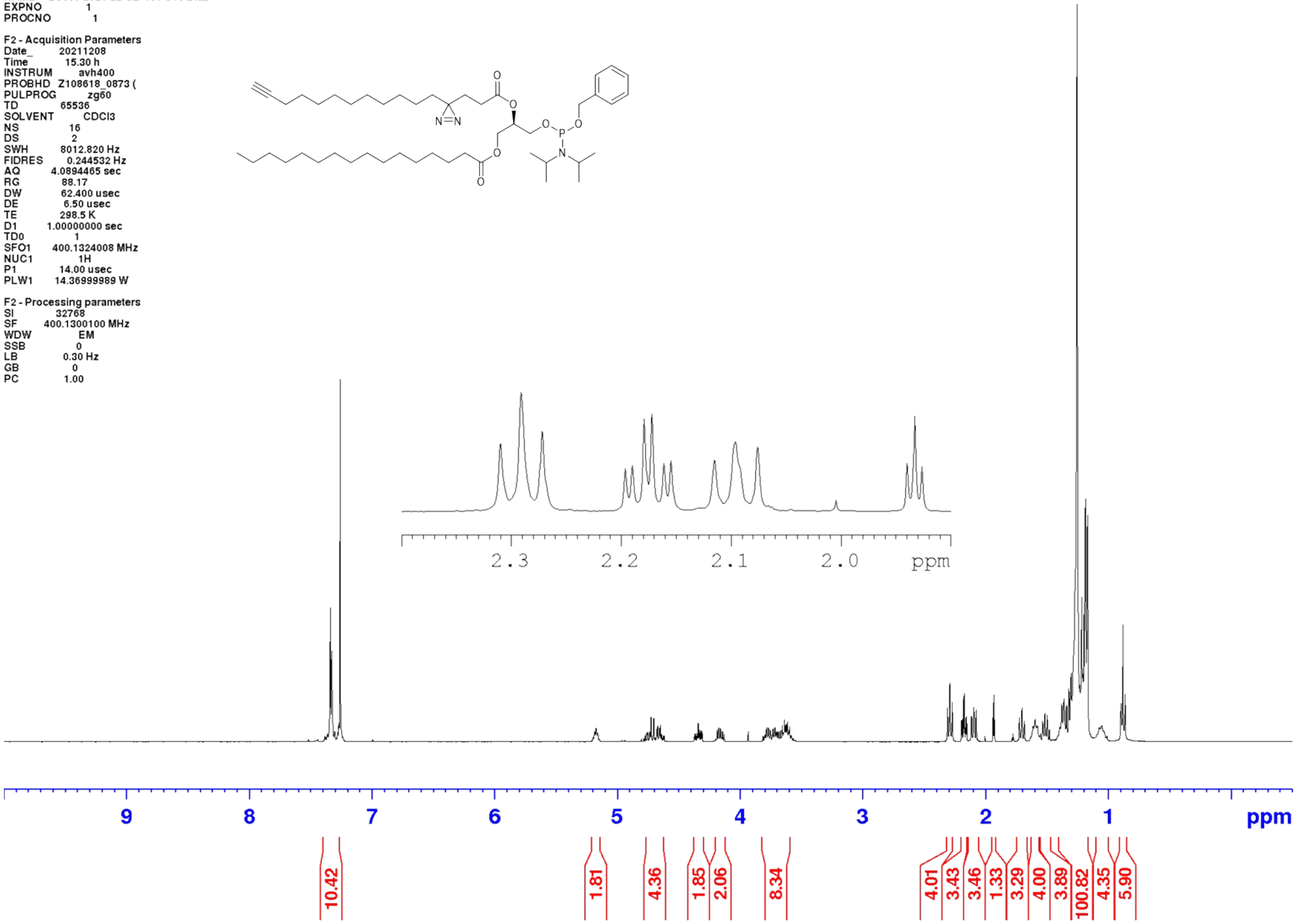

(2*R*)-3-(((benzyloxy)(diisopropylamino)phosphaneyl)oxy)-2-((3-(3-(dodec-11-yn-1-yl)-3H-diazirin-3-yl)propanoyl)oxy)propyl palmitate 34 ^{31}P NMR

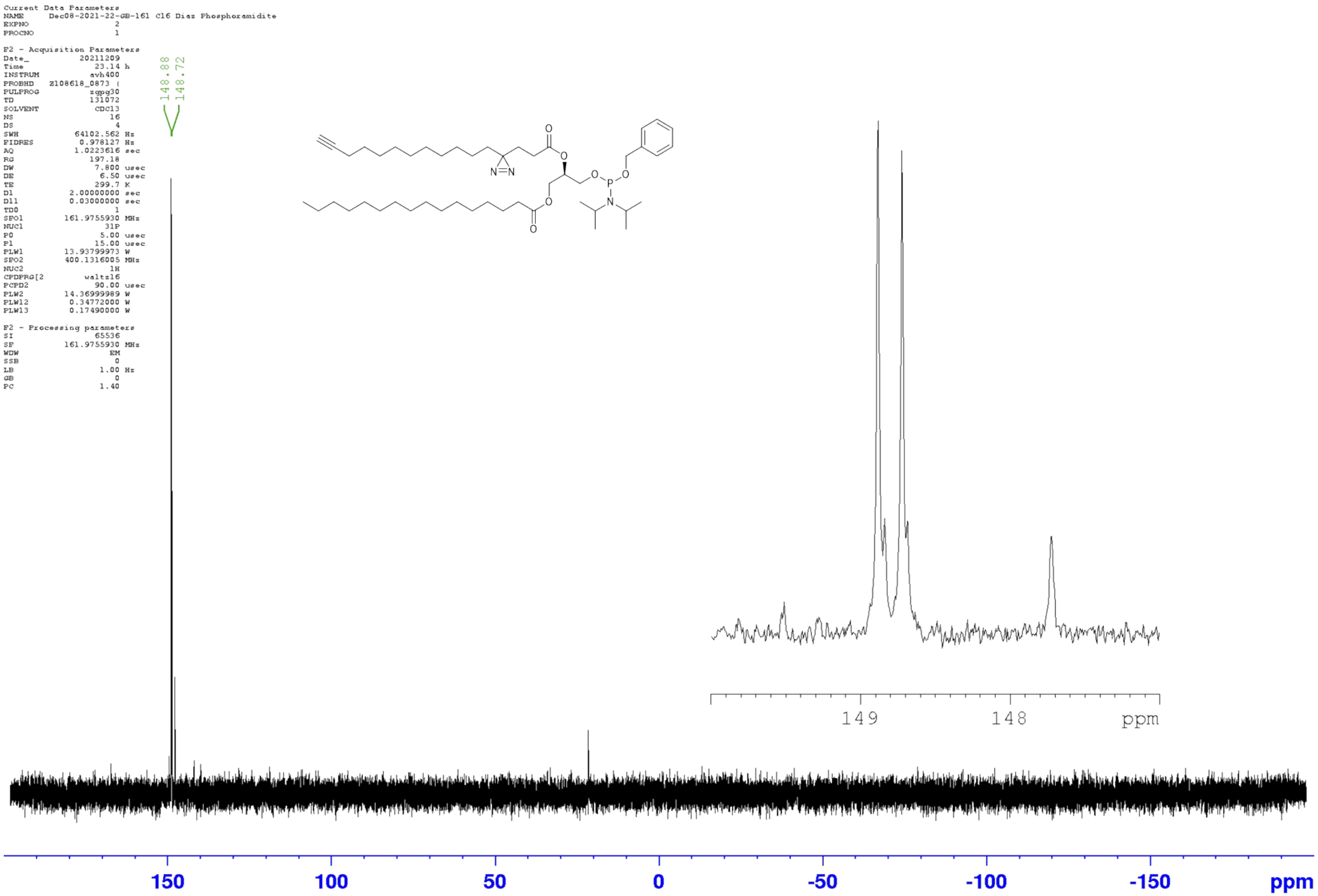

(2*R*)-3-(((benzyloxy)(diisopropylamino)phosphaneyl)oxy)-2-((3-(3-(dodec-11-yn-1-yl)-3H-diazirin-3-yl)propanoyl)oxy)propyl palmitate 34 HRMS

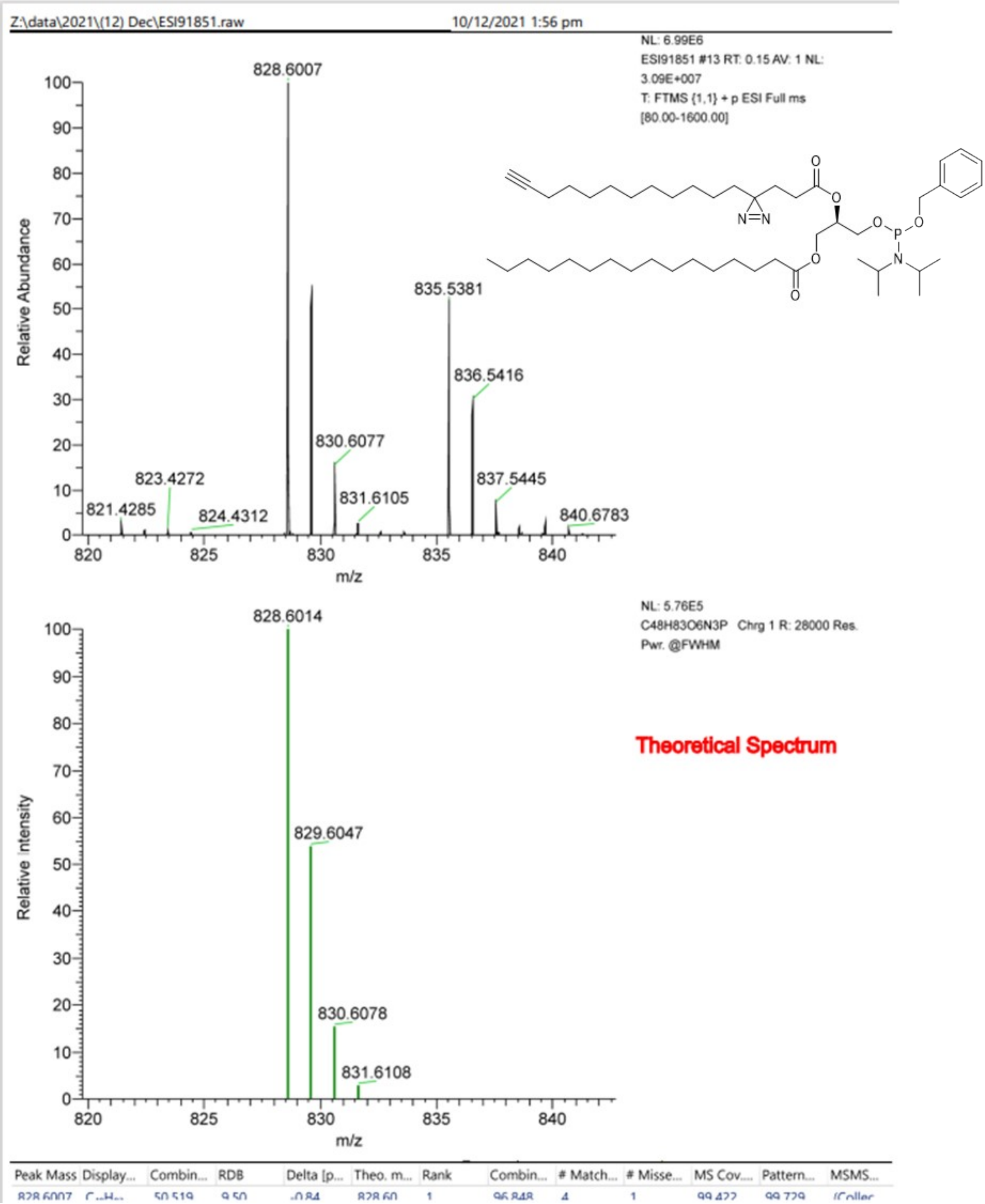

(+)-(2*R*)-3-(((Benzyloxy)(((3a*R*,4*R*,5*S*,6*R*,7*S*,7a*R*)-4,6-bis((4-methoxybenzyl)oxy)-5-((3-oxido-1,5-dihydrobenzo[e][1,3,2]dioxaphosphepin-3-yl)oxy)hexahydrospiro[benzo[d][1,3]dioxole-2,1'-cyclopentan]-7-yl)oxy)phosphoryl)oxy)-2-((3-(3-(dodec-11-yn-1-yl)-3*H*-diazirin-3-yl)propanoyl)oxy)propyl palmitate (+)-35 ^{1}H NMR

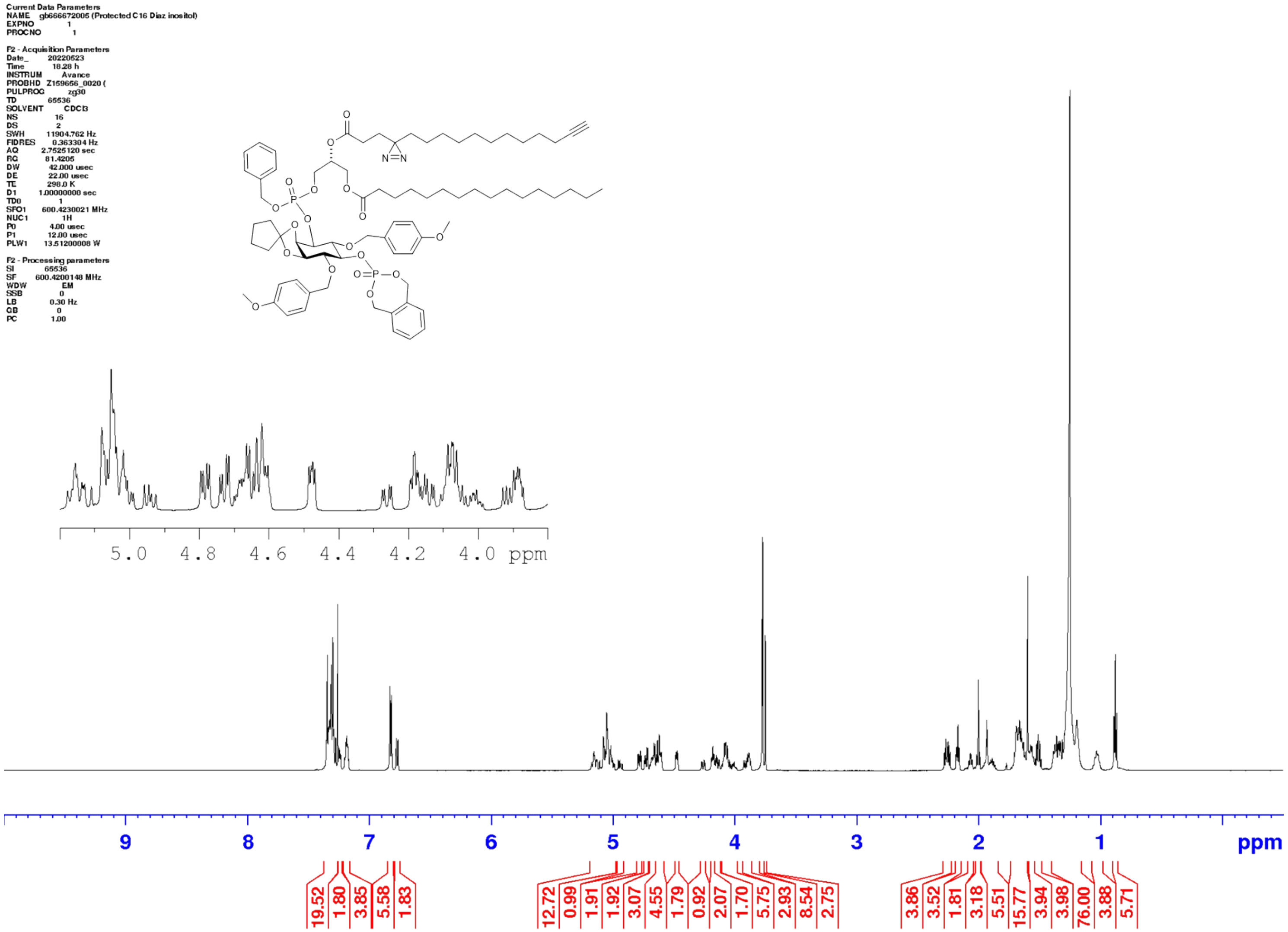

(+)-(2*R*)-3-(((Benzyloxy)(((3a*R*,4*R*,5*S*,6*R*,7*S*,7a*R*)-4,6-bis((4-methoxybenzyl)oxy)-5-((3-oxido-1,5-dihydrobenzo[e][1,3,2]dioxaphosphepin-3-yl)oxy)hexahydrospiro[benzo[d][1,3]dioxole-2,1'-cyclopentan]-7-yl)oxy)phosphoryl)oxy)-2-((3-(3-(dodec-11-yn-1-yl)-3*H*-diazirin-3-yl)propanoyl)oxy)propyl palmitate (+)-35 ^{31}P NMR

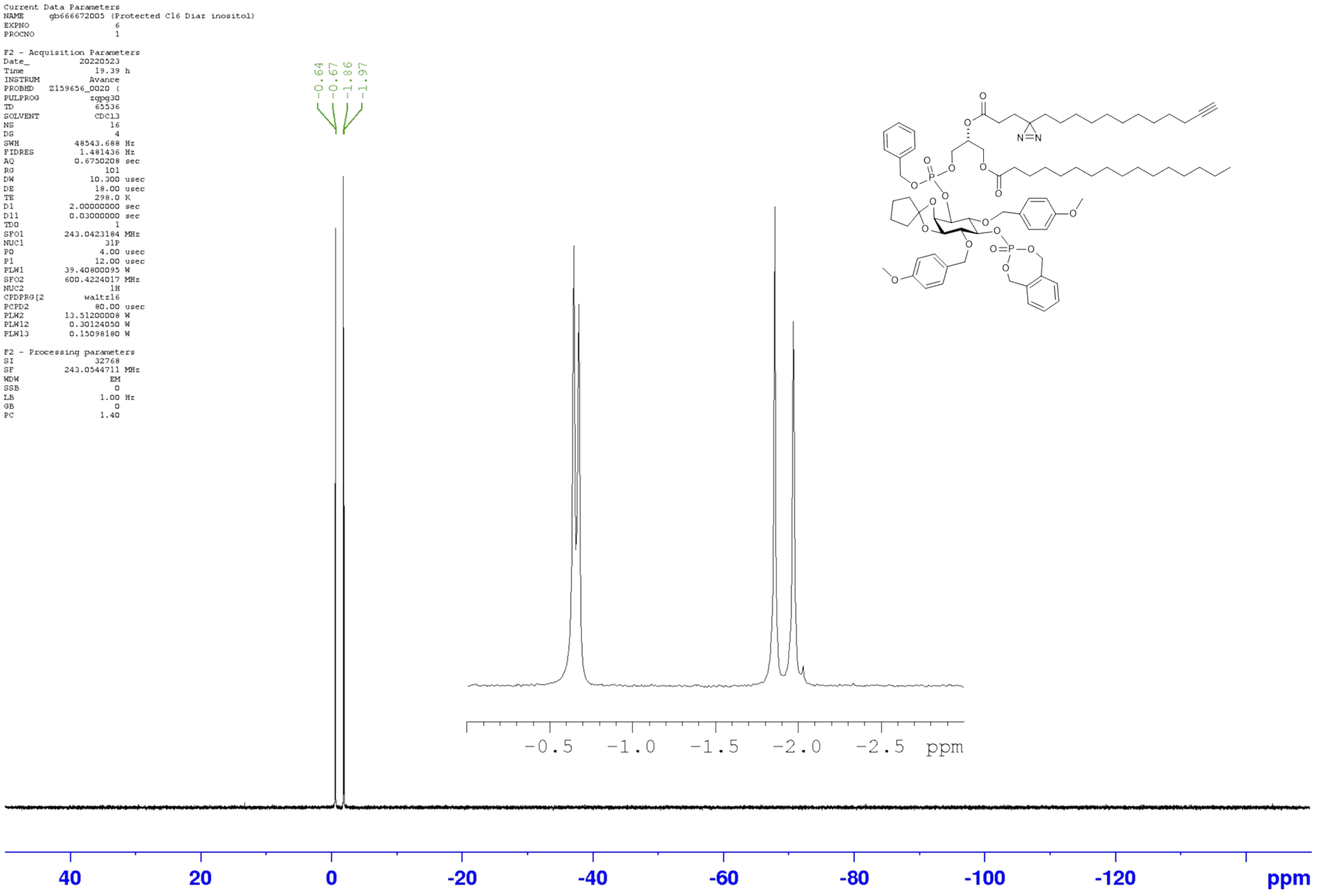

(+)-(2*R*)-3-(((Benzyloxy)(((3a*R*,4*R*,5*S*,6*R*,7*S*,7a*R*)-4,6-bis((4-methoxybenzyl)oxy)-5-((3-oxido-1,5-dihydrobenzo[e][1,3,2]dioxaphosphepin-3-yl)oxy)hexahydrospiro[benzo[d][1,3]dioxole-2,1'-cyclopentan]-7-yl)oxy)phosphoryl)oxy)-2-((3-(3-(dodec-11-yn-1-yl)-3*H*-diazirin-3-yl)propanoyl)oxy)propyl palmitate (+)-35 ^{13}C NMR

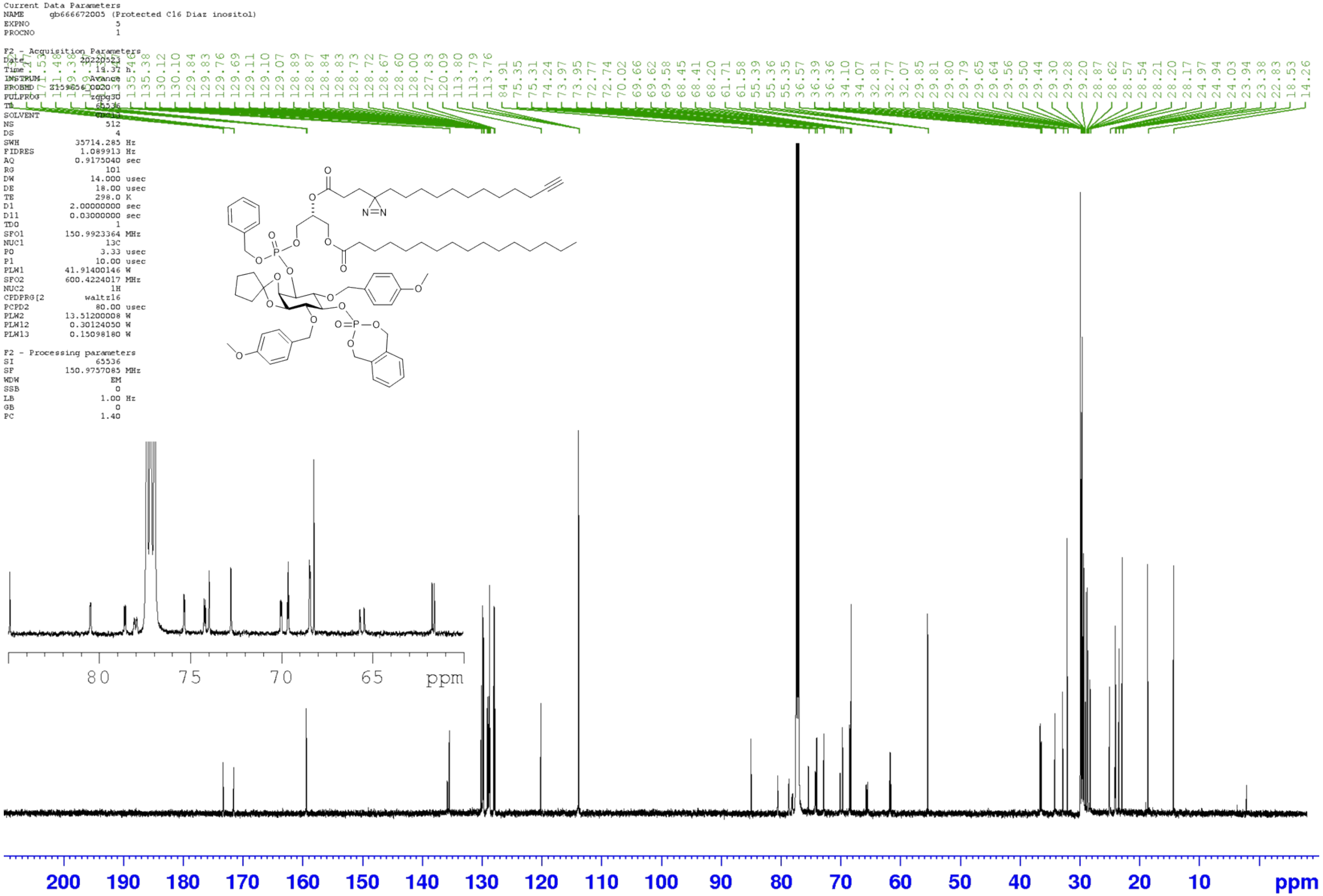

(+)-(2*R*)-3-(((Benzyloxy)(((3a*R*,4*R*,5*S*,6*R*,7*S*,7a*R*)-4,6-bis((4-methoxybenzyl)oxy)-5-((3-oxido-1,5-dihydrobenzo[e][1,3,2]dioxaphosphepin-3-yl)oxy)hexahydrospiro[benzo[d][1,3]dioxole-2,1'-cyclopentan]-7-yl)oxy)phosphoryl)oxy)-2-((3-(3-(dodec-11-yn-1-yl)-3*H*-diazirin-3-yl)propanoyl)oxy)propyl palmitate (+)-35

HPLC 280nm

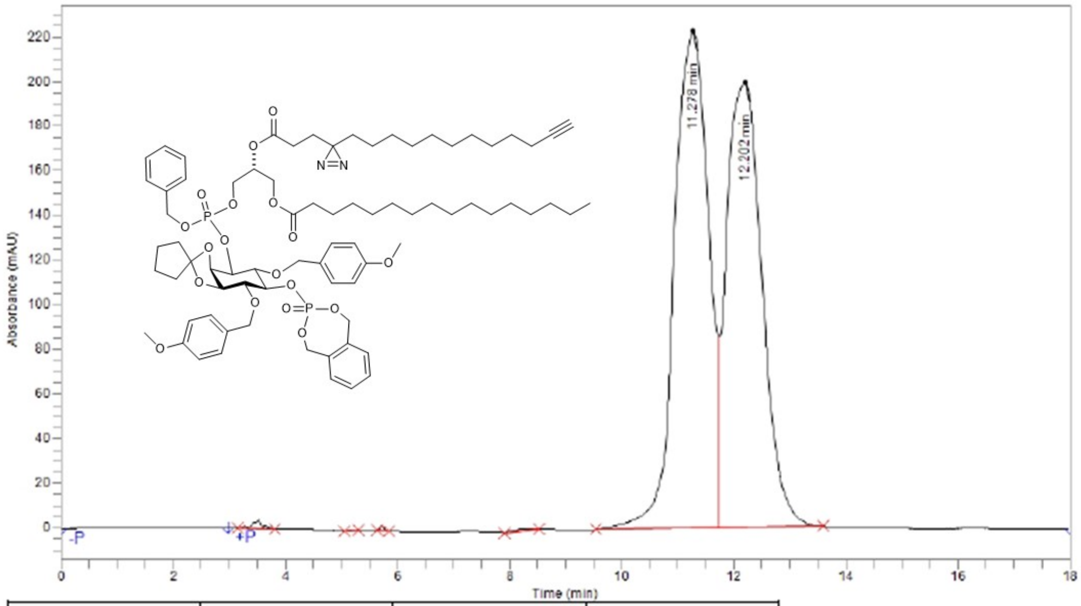

Time	Height	Area	Area %
3.277	875.6	4,641.7	0.03
3.536	3,577.9	31,004.7	0.17
3.634	1,172.9	6,234.2	0.03
5.162	546.7	3,752.2	0.02
5.722	2,163.9	10,848.1	0.06
8.419	419.9	25,820.5	0.14
11.278	222,879.7	9,439,156.3	51.54
12.202	199,256.7	8,792,337.5	48.01
**Total**		18,313,795.1	100.00

(+)-(2*R*)-3-(((Benzyloxy)(((3a*R*,4*R*,5*S*,6*R*,7*S*,7a*R*)-4,6-bis((4-methoxybenzyl)oxy)-5-((3-oxido-1,5-dihydrobenzo[e][1,3,2]dioxaphosphepin-3-yl)oxy)hexahydrospiro[benzo[d][1,3]dioxole-2,1'-cyclopentan]-7-yl)oxy)phosphoryl)oxy)-2-((3-(3-(dodec-11-yn-1-yl)-3*H*-diazirin-3-yl)propanoyl)oxy)propyl palmitate (+)-35

HPLC 254nm

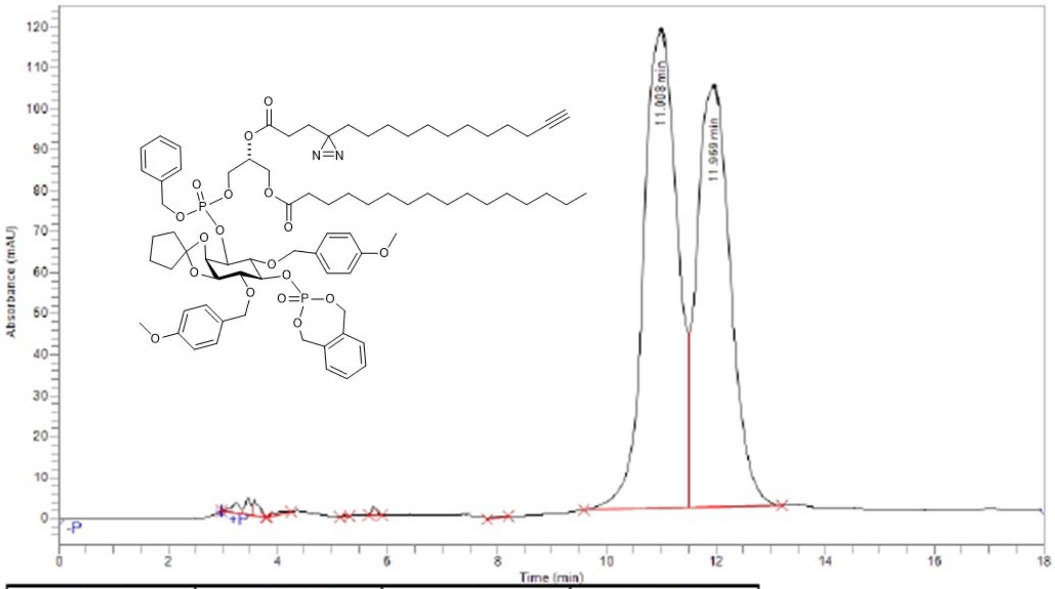

Time	Height	Area	Area %
3.266	2,471.9	29,013.9	0.30
3.473	4,153.9	30,649.7	0.32
3.586	3,855.9	30,122.2	0.32
4.194	245.7	9,503.5	0.10
5.241	236.7	1,224.2	0.01
5.765	2,005.5	11,704.4	0.12
8.112	231.2	7,705.9	0.08
11.008	116,975.3	4,977,390.4	52.24
11.969	102,803.8	4,430,765.5	46.50
Total		9,528,079.6	100.00

(+)-(2*R*)-3-(((Benzyloxy)(((3a*R*,4*R*,5*S*,6*R*,7*S*,7a*R*)-4,6-bis((4-methoxybenzyl)oxy)-5-((3-oxido-1,5-dihydrobenzo[e][1,3,2]dioxaphosphepin-3-yl)oxy)hexahydrospiro[benzo[d][1,3]dioxole-2,1'-cyclopentan]-7-yl)oxy)phosphoryl)oxy)-2-((3-(3-(dodec-11-yn-1-yl)-3*H*-diazirin-3-yl)propanoyl)oxy)propyl palmitate (+)-35 HRMS

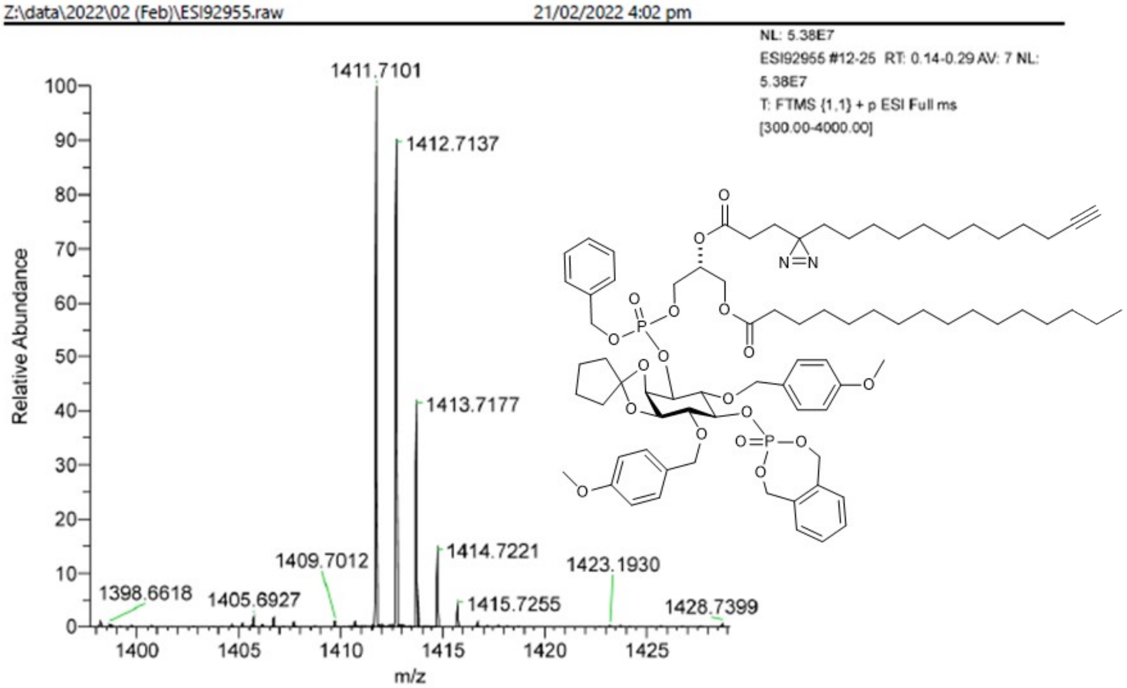

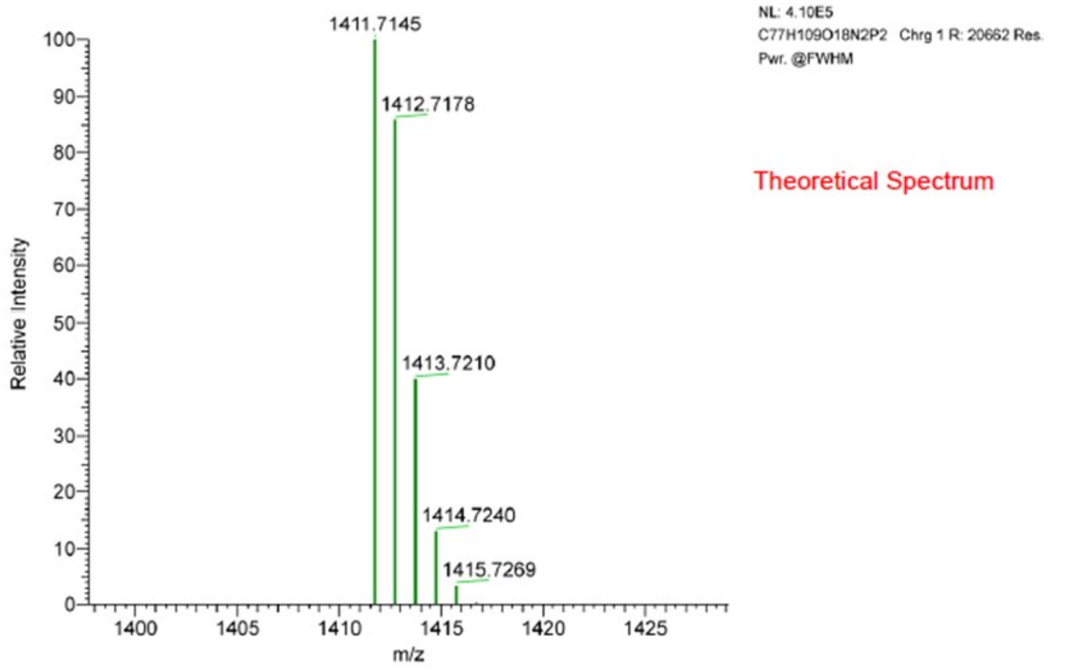

Peak Mass	Display...	Combin...	RDB	Delta [p...	Theo. m...	Rank	Combin...	# Match...	# Misse...	MS Cov....	Pattern...	MSMS...
1411.7	[illegible]	50.588	25.50	-3.12	1411.7	1	94.616	7	0	97.062	99.947	(Collec

Ammonium (1S,2R,3R,4R,5R,6R)-3-((((R)-2-((3-(3-(dodec-11-yn-1-yl)-3H-diazirin-3-yl)propanoyl)oxy)-3-(palmitoyloxy)propoxy)oxidophosphoryl)oxy)-2,4,5,6-tetrahydroxycyclohexyl phosphate 9 ^{1}H NMR

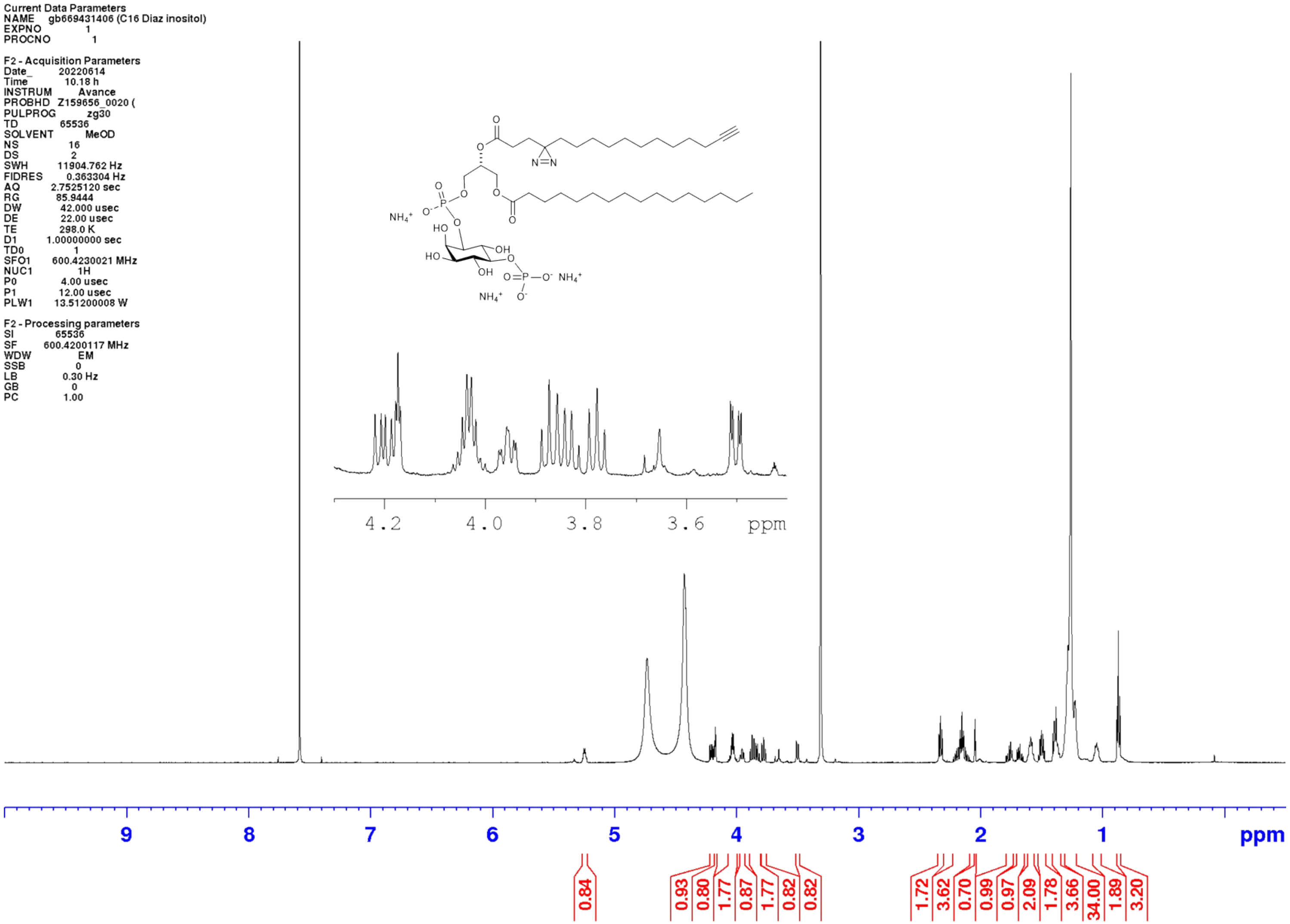

Ammonium (1S,2R,3R,4R,5R,6R)-3-((((R)-2-((3-(3-(dodec-11-yn-1-yl)-3H-diazirin-3-yl)propanoyl)oxy)-3-(palmitoyloxy)propoxy)oxidophosphoryl)oxy)-2,4,5,6-tetrahydroxycyclohexyl phosphate 9 ^{31}P NMR

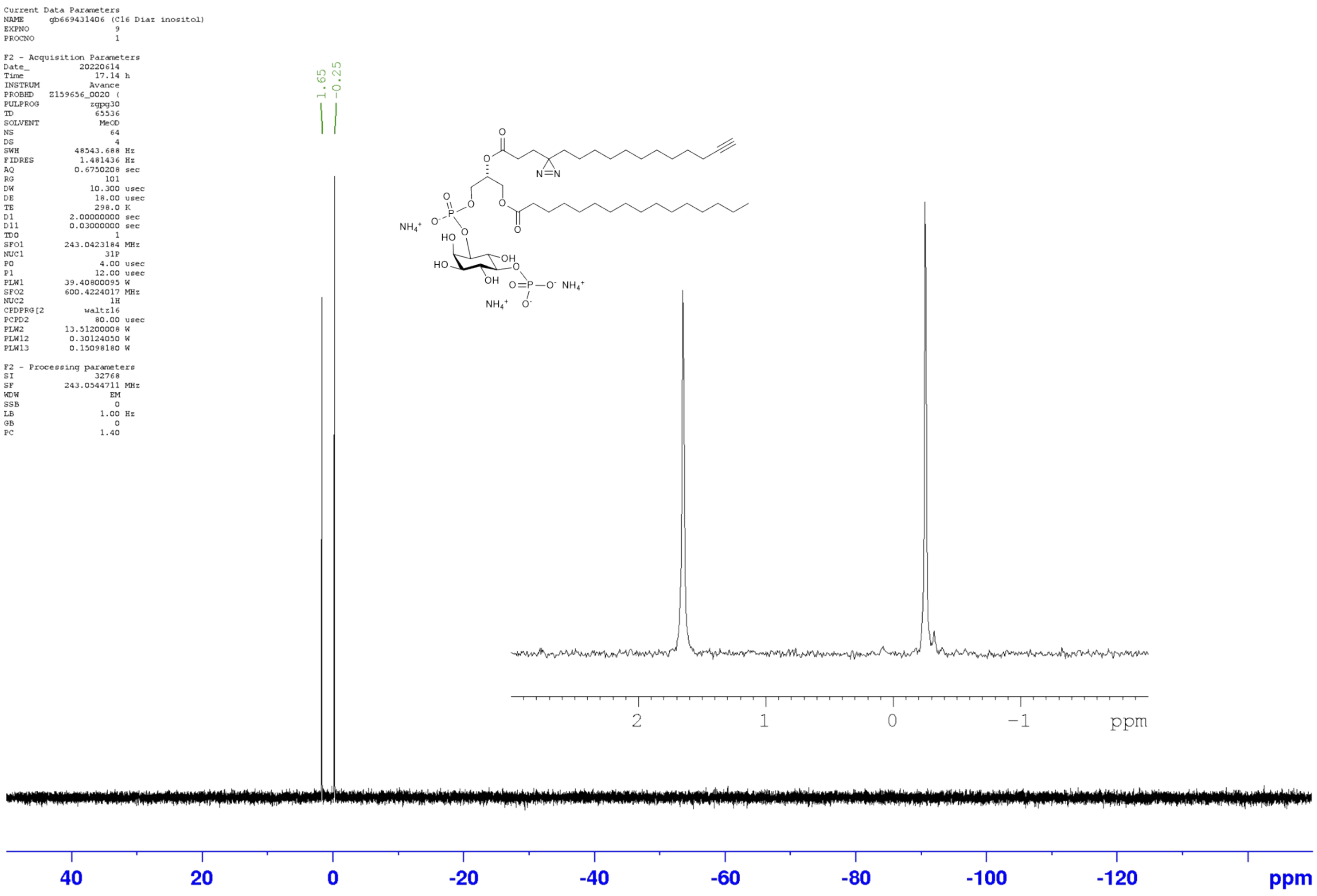

Ammonium (1S,2R,3R,4R,5R,6R)-3-((((R)-2-((3-(3-(dodec-11-yn-1-yl)-3H-diazirin-3-yl)propanoyl)oxy)-3-(palmitoyloxy)propoxy)oxidophosphoryl)oxy)-2,4,5,6-tetrahydroxycyclohexyl phosphate 9 ^{13}C NMR

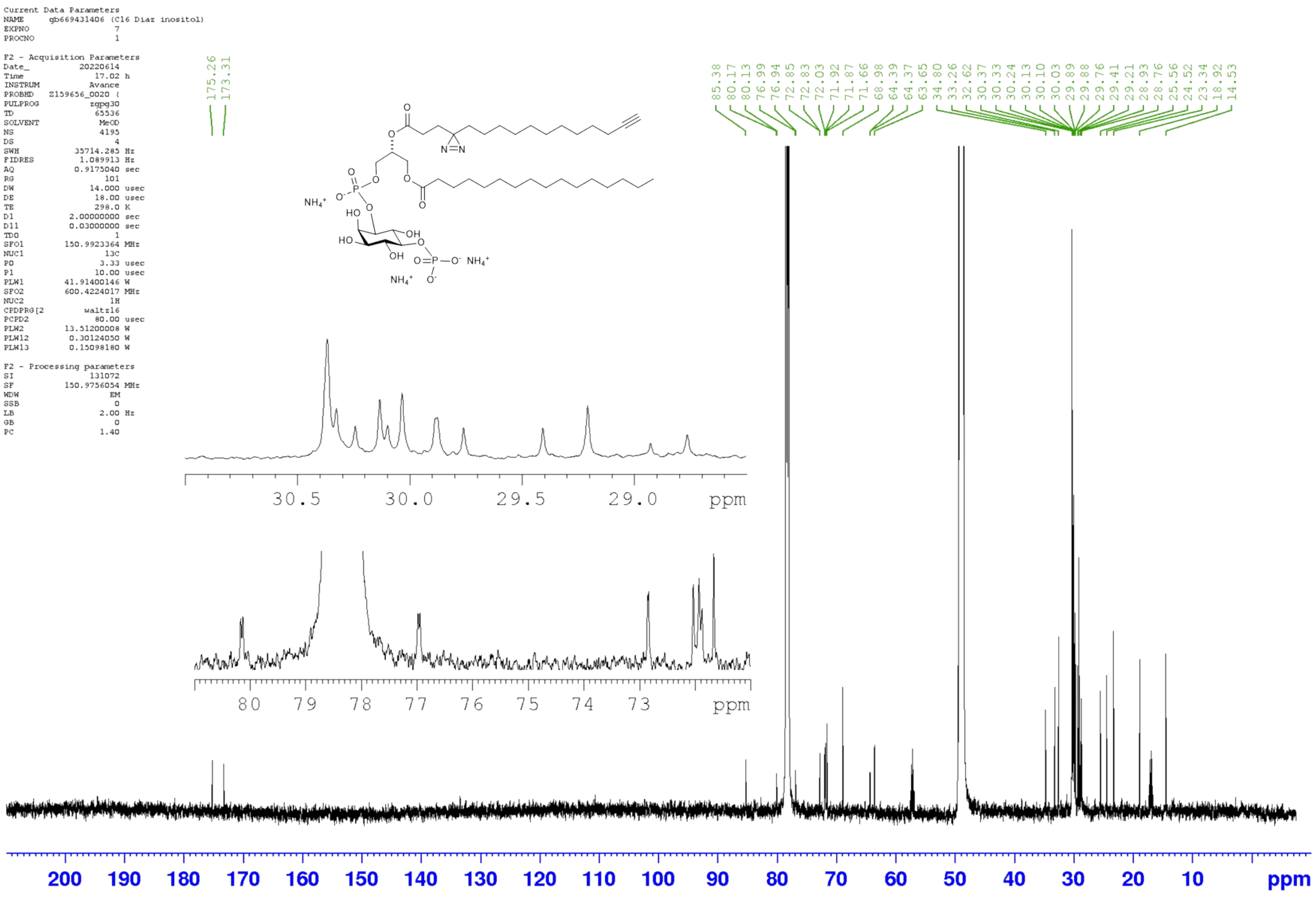

Ammonium (1S,2R,3R,4R,5R,6R)-3-((((R)-2-((3-(3-(dodec-11-yn-1-yl)-3H-diazirin-3-yl)propanoyl)oxy)-3-(palmitoyloxy)propoxy)oxidophosphoryl)oxy)-2,4,5,6-tetrahydroxycyclohexyl phosphate 9 ^{1}H to ^{13}C HSQC NMR

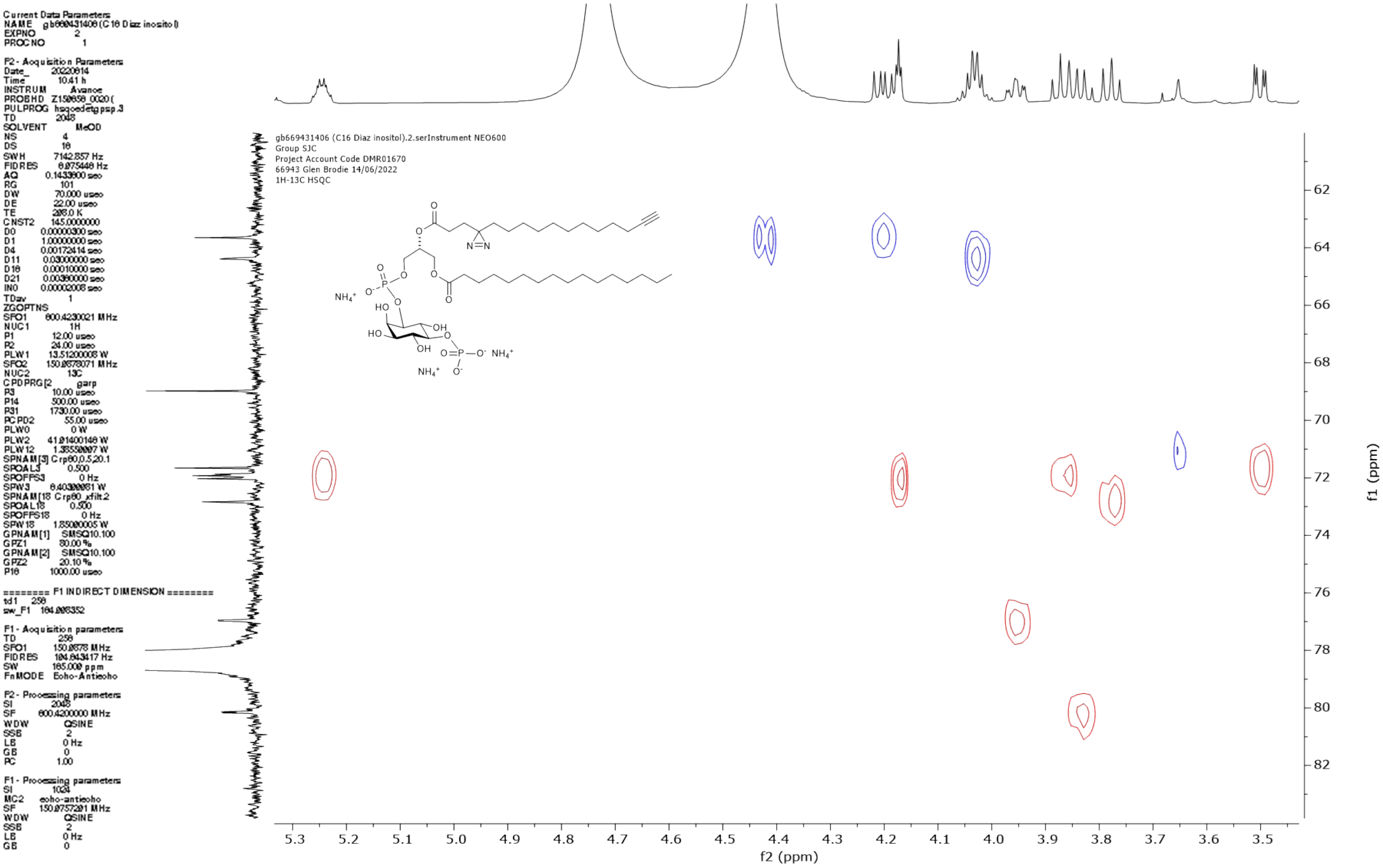

Ammonium (1S,2R,3R,4R,5R,6R)-3-((((R)-2-((3-(3-(dodec-11-yn-1-yl)-3H-diazirin-3-yl)propanoyl)oxy)-3-(palmitoyloxy)propoxy)oxidophosphoryl)oxy)-2,4,5,6-tetrahydroxycyclohexyl phosphate 9 HRMS

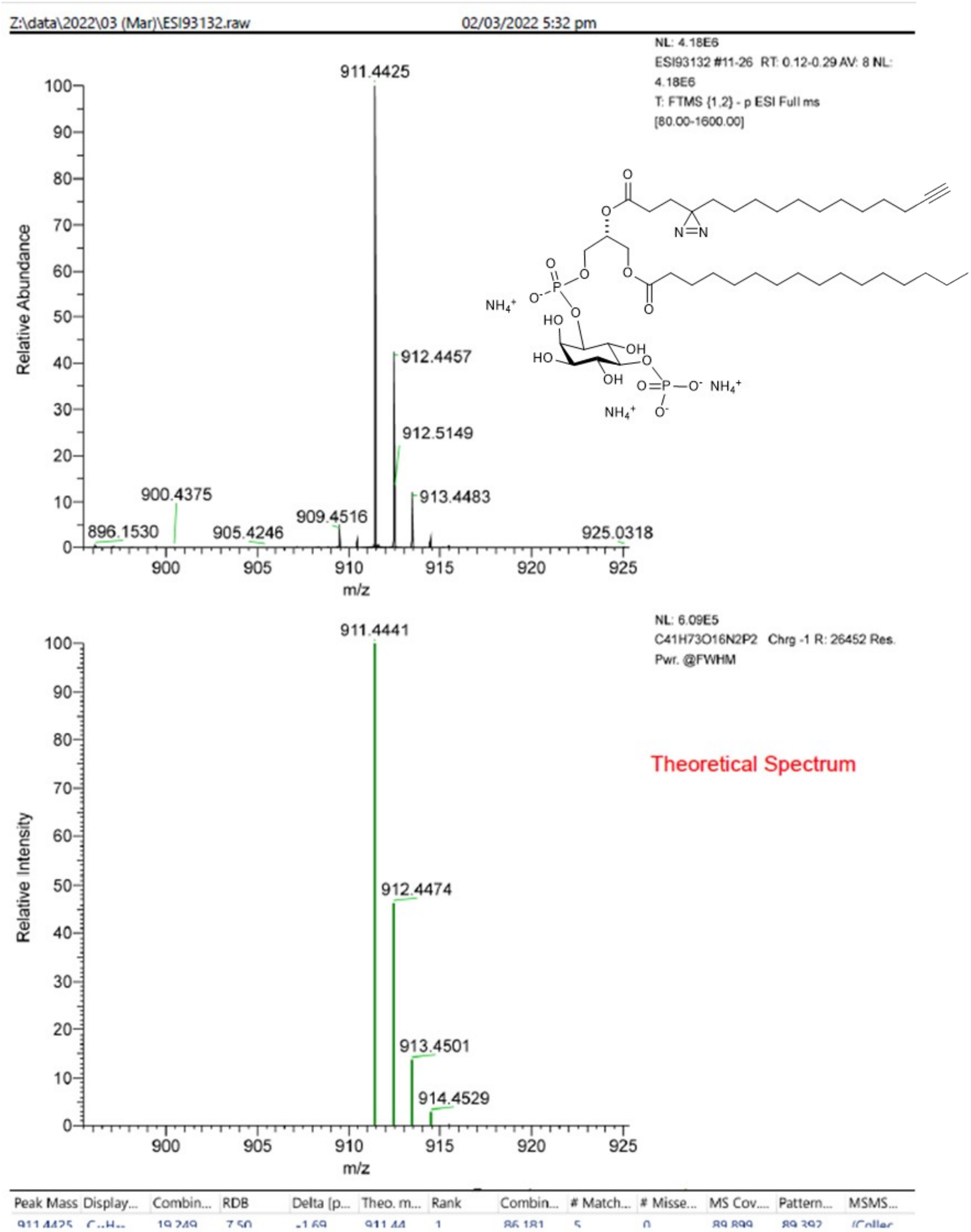

(+)-(2*S*)-3-[bis(4-methoxyphenyl)(phenyl)methoxy]-2-hydroxypropyl hexadecanoate (+)-S10 ^{1}H NMR

Current Data Parameters
NAME Apr01-2021-42-GB-043
EXPNO 1
PROCNO 1

F2 - Acquisition Parameters
Date_ 20210401
Time 8.45 h
INSTRUM avg400
PROBHD Z108618_0816 (
PULPROG zg60
TD 65536
SOLVENT CDCl3
NS 16
DS 2
SWH 8012.820 Hz
FIDRES 0.244532 Hz
AQ 4.0894465 sec
RG 35.2
DW 62.400 usec
DE 6.50 usec
TE 298.1 K
D1 1.00000000 sec
TD0 1
SFO1 400.2024012 MHz
NUC1 1H
P1 14.00 usec
PLW1 14.00000000 W

F2 - Processing parameters
SI 32768
SF 400.2000094 MHz
WDW EM
SSB 0
LB 0.30 Hz
GB 0
PC 1.00

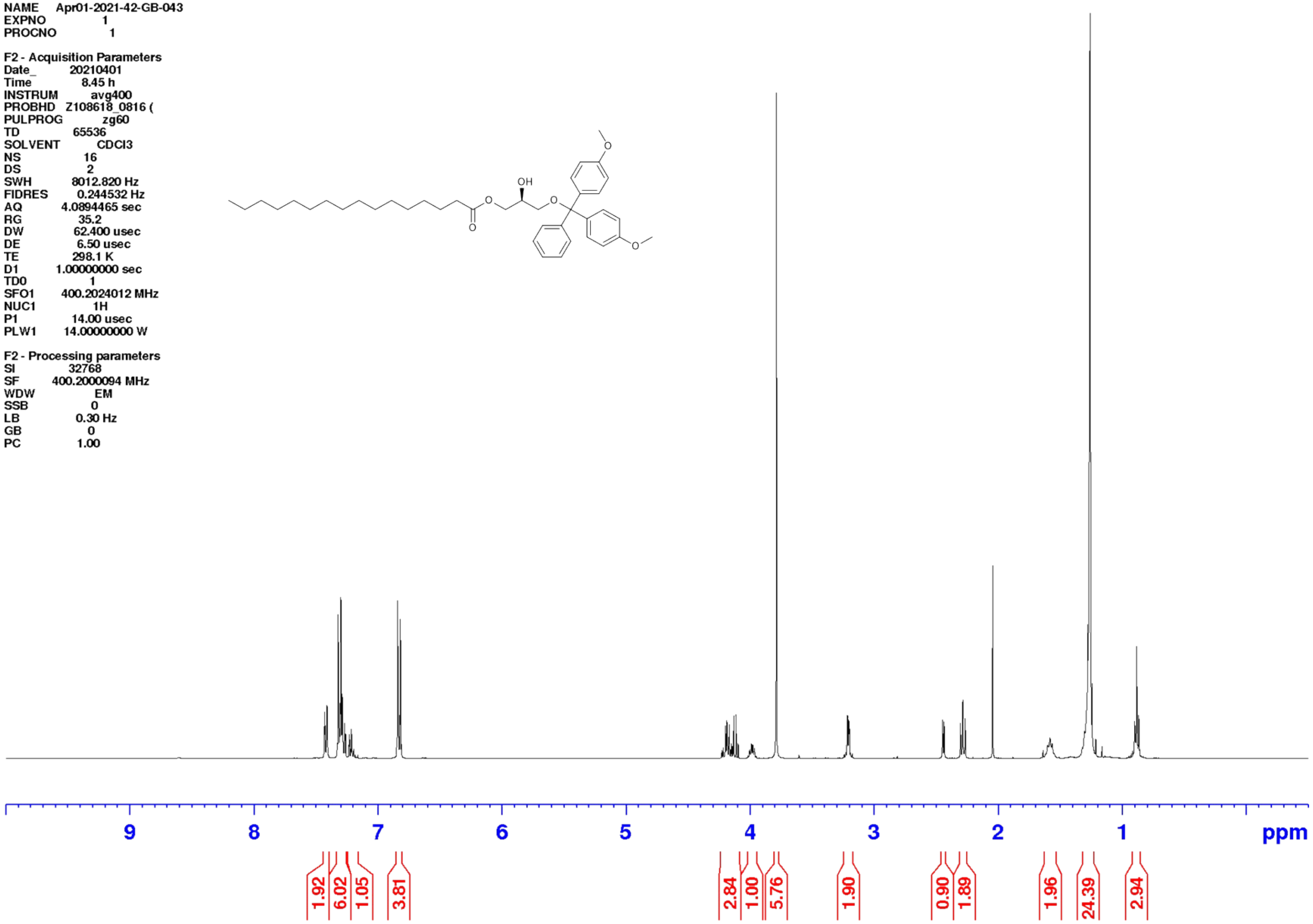

(+)-(2*S*)-3-[bis(4-methoxyphenyl)(phenyl)methoxy]-2-hydroxypropyl hexadecanoate (+)-S10 ^{13}C NMR

```
Current Data Parameters
NAME      Apr01-2021-42-GB-043
EXPNO                    2
PROCNO                   1

F2 - Acquisition Parameters
Date_              20210401
Time                  10.06 h
INSTRUM              avg400
PROBHD     Z108618_0816 (
PULPROG             zgpg30
TD                   32768
SOLVENT              CDCl3
NS                     512
DS                       4
SWH              26041.666 Hz
FIDRES            1.589457 Hz
AQ               0.6291456 sec
RG                  206.87
DW                  19.200 usec
DE                    6.50 usec
TE                   299.0 K
D1              1.00000000 sec
D11             0.03000000 sec
TD0                      1
SFO1           100.6404331 MHz
NUC1                   13C
P0                    3.33 usec
P1                   10.00 usec
PLW1           56.00000000 W
SFO2           400.2016008 MHz
NUC2                    1H
CPDPRG[2           waltz16
PCPD2                90.00 usec
PLW2           14.00000000 W
PLW12           0.33877000 W
PLW13           0.17039999 W

F2 - Processing parameters
SI                   32768
SF             100.6303590 MHz
WDW                     EM
SSB                      0
LB                    1.00 Hz
GB                       0
PC                    1.40
```

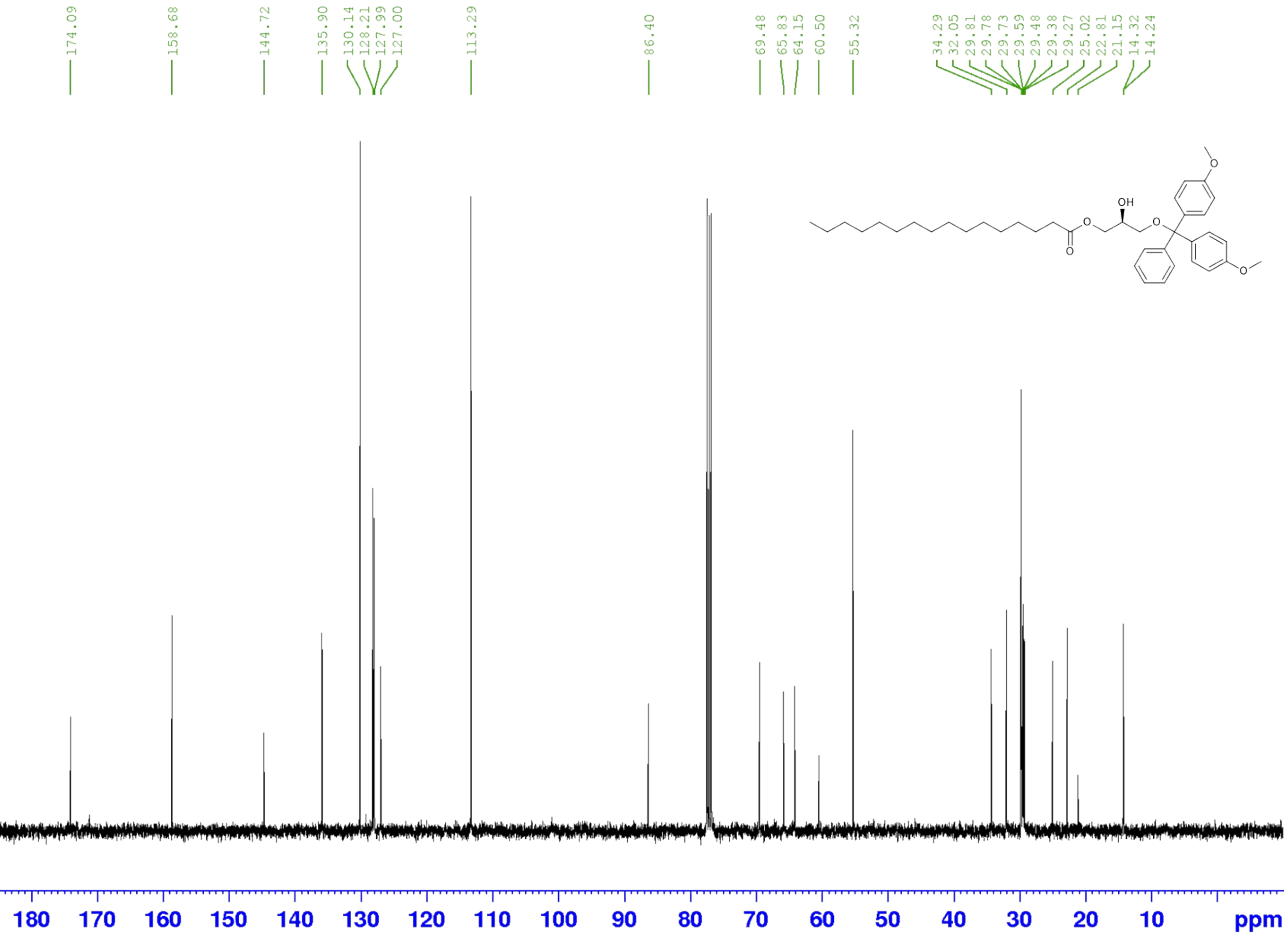

(+)-(2*S*)-3-[bis(4-methoxyphenyl)(phenyl)methoxy]-2-hydroxypropyl hexadecanoate (+)-S10
HRMS

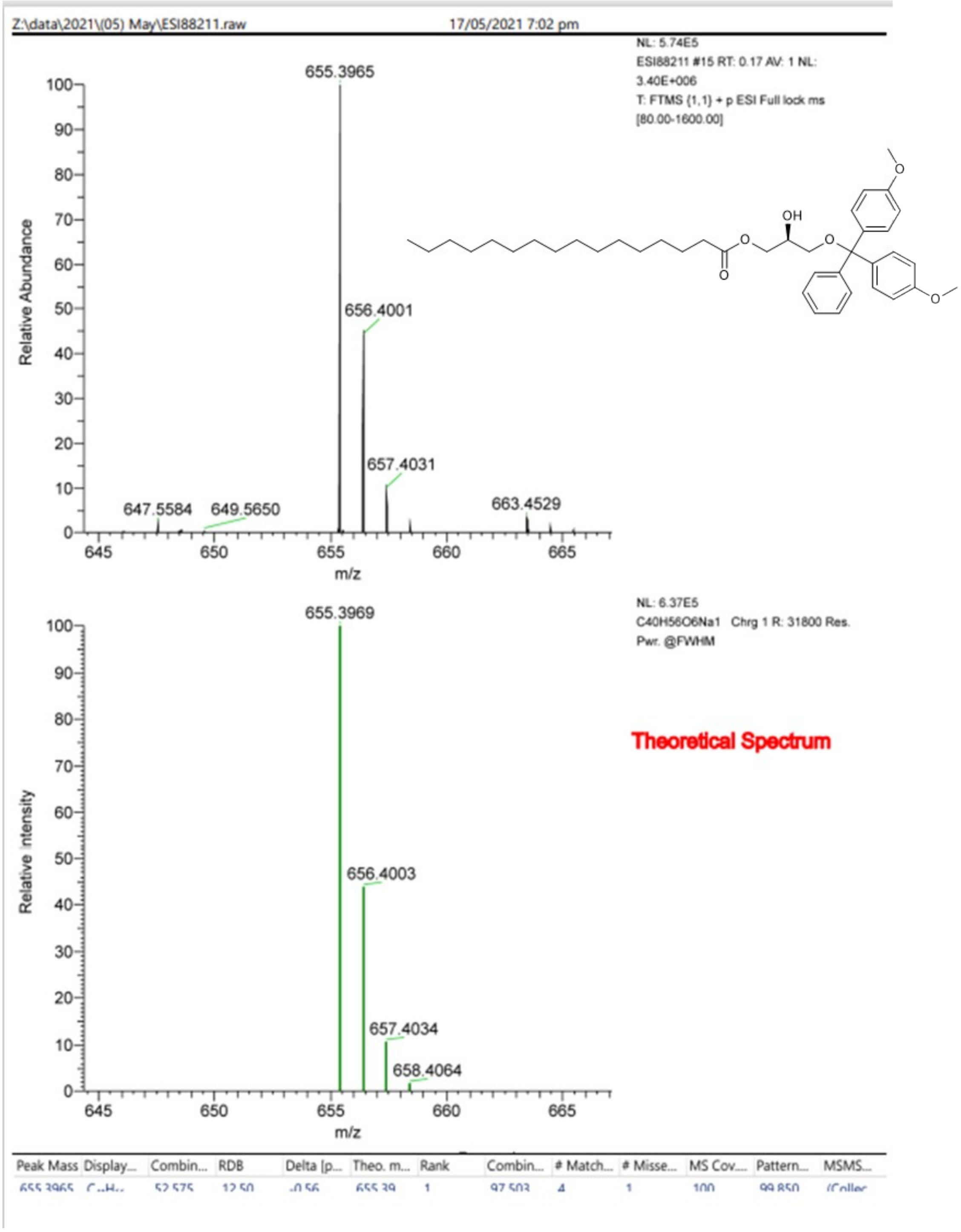

(+)-(2*S*)-3-[bis(4-methoxyphenyl)(phenyl)methoxy]-2-hydroxypropyl octanoate (+)-S11 ^{1}H NMR

Current Data Parameters
NAME Apr05-2021-2-GB-044
EXPNO 1
PROCNO 1

F2 - Acquisition Parameters
Date_ 20210405
Time 9.29 h
INSTRUM avg400
PROBHD Z108618_0816 (
PULPROG zg60
TD 65536
SOLVENT CDCl3
NS 16
DS 2
SWH 8012.820 Hz
FIDRES 0.244532 Hz
AQ 4.0894465 sec
RG 91.39
DW 62.400 usec
DE 6.50 usec
TE 297.7 K
D1 1.00000000 sec
TD0 1
SFO1 400.2024012 MHz
NUC1 1H
P1 14.00 usec
PLW1 14.00000000 W

F2 - Processing parameters
SI 32768
SF 400.2000094 MHz
WDW EM
SSB 0
LB 0.30 Hz
GB 0
PC 1.00

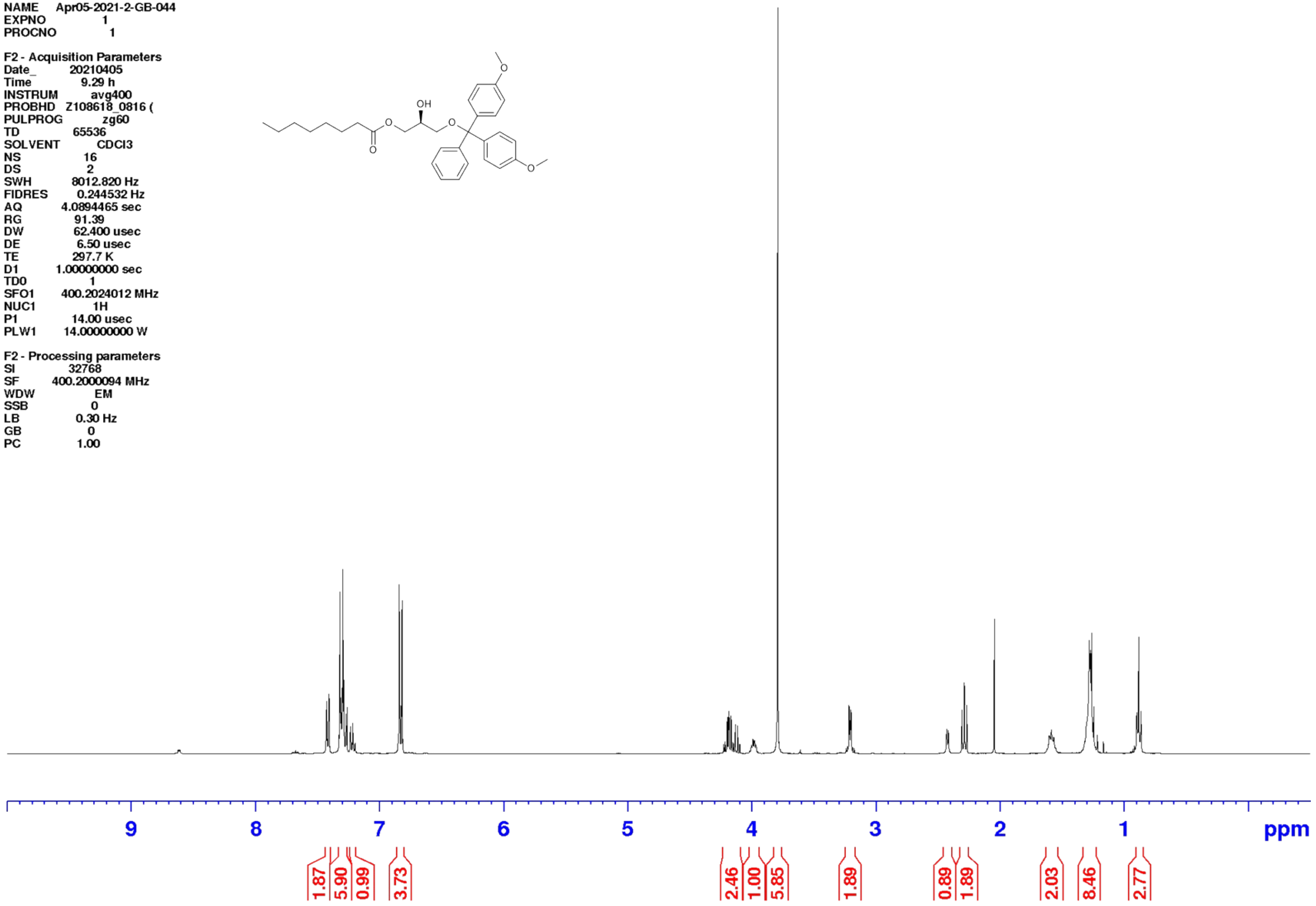

(+)-(2*S*)-3-[bis(4-methoxyphenyl)(phenyl)methoxy]-2-hydroxypropyl octanoate (+)-S11 ^{13}C NMR

```
Current Data Parameters
NAME     Apr05-2021-2-GB-044
EXPNO                      2
PROCNO                     1

F2 - Acquisition Parameters
Date_              20210405
Time                  10.11 h
INSTRUM              avg400
PROBHD   Z108618_0816 (
PULPROG             zgpg30
TD                   32768
SOLVENT              CDCl3
NS                     512
DS                       4
SWH              26041.666 Hz
FIDRES            1.589457 Hz
AQ               0.6291456 sec
RG                  206.87
DW                  19.200 usec
DE                    6.50 usec
TE                   298.8 K
D1              1.00000000 sec
D11             0.03000000 sec
TD0                      1
SFO1           100.6404331 MHz
NUC1                   13C
P0                    3.33 usec
P1                   10.00 usec
PLW1           56.00000000 W
SFO2           400.2016008 MHz
NUC2                    1H
CPDPRG[2           waltz16
PCPD2                90.00 usec
PLW2           14.00000000 W
PLW12           0.33877000 W
PLW13           0.17039999 W

F2 - Processing parameters
SI                   32768
SF             100.6303577 MHz
WDW                     EM
SSB                      0
LB                    1.00 Hz
GB                       0
PC                    1.40
```

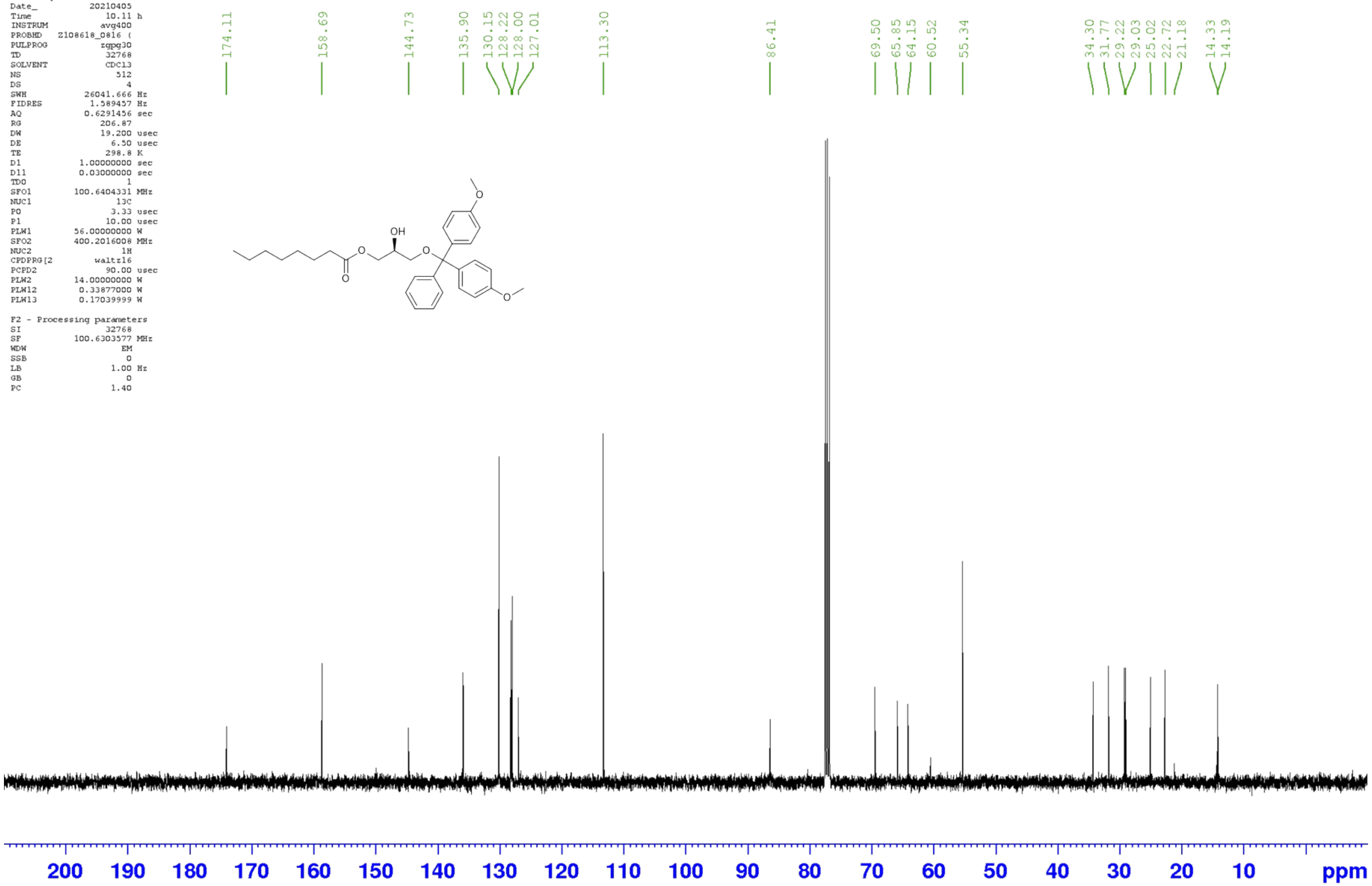

(+)-(2*S*)-3-[bis(4-methoxyphenyl)(phenyl)methoxy]-2-hydroxypropyl octanoate (+)-S11 HRMS

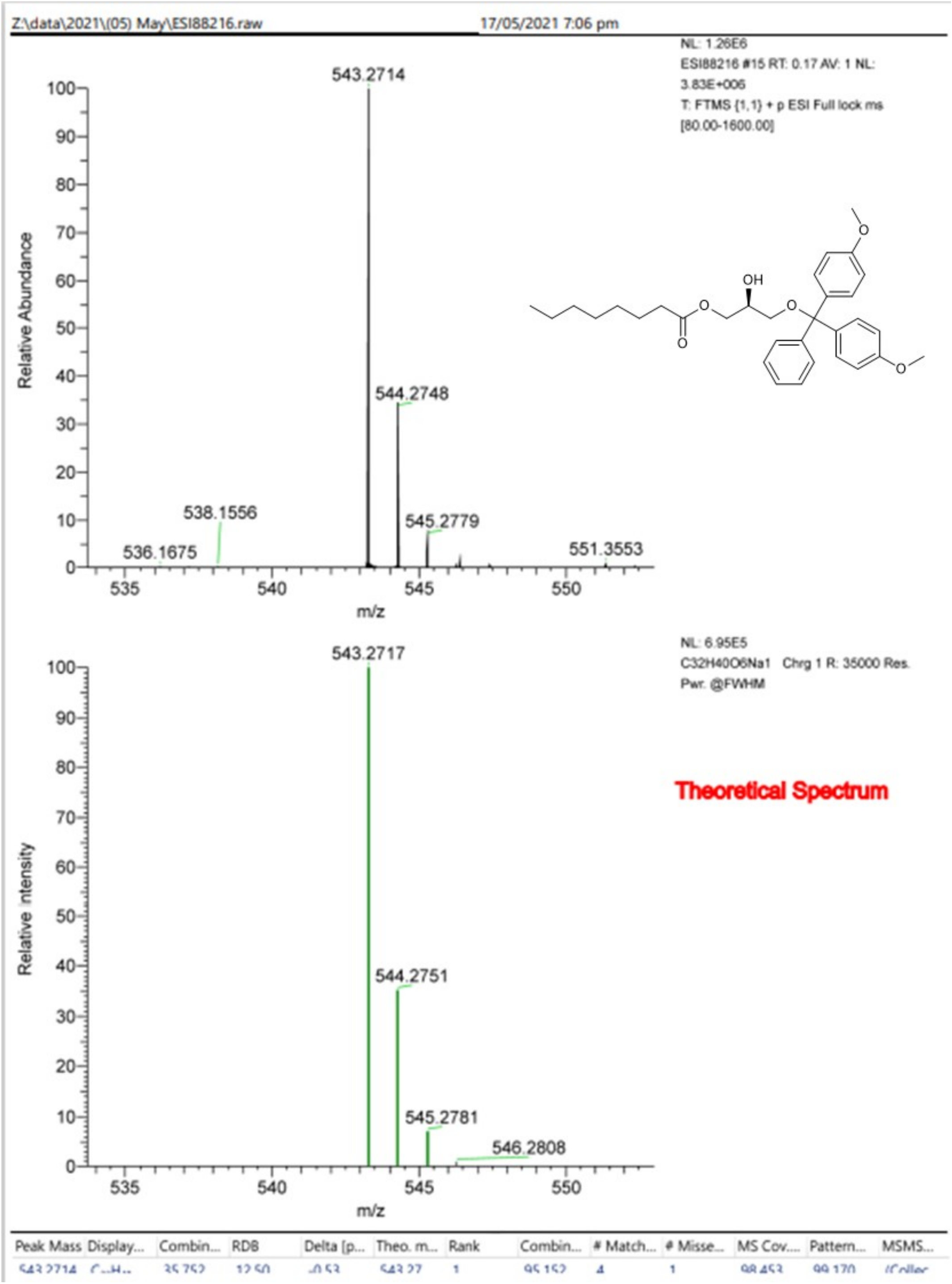

(+)-(2*S*)-3-[bis(4-methoxyphenyl)(phenyl)methoxy]-2-(octanoyloxy)propyl hexadecanoate (+)-S12 ^{1}H NMR

Current Data Parameters
NAME Apr05-2021-3-GB-045
EXPNO 1
PROCNO 1

F2 - Acquisition Parameters
Date_ 20210405
Time 9.34 h
INSTRUM avg400
PROBHD Z108618_0816 (
PULPROG zg60
TD 65536
SOLVENT CDCl3
NS 16
DS 2
SWH 8012.820 Hz
FIDRES 0.244532 Hz
AQ 4.0894465 sec
RG 39.24
DW 62.400 usec
DE 6.50 usec
TE 297.7 K
D1 1.00000000 sec
TD0 1
SFO1 400.2024012 MHz
NUC1 1H
P1 14.00 usec
PLW1 14.00000000 W

F2 - Processing parameters
SI 32768
SF 400.2000099 MHz
WDW EM
SSB 0
LB 0.30 Hz
GB 0
PC 1.00

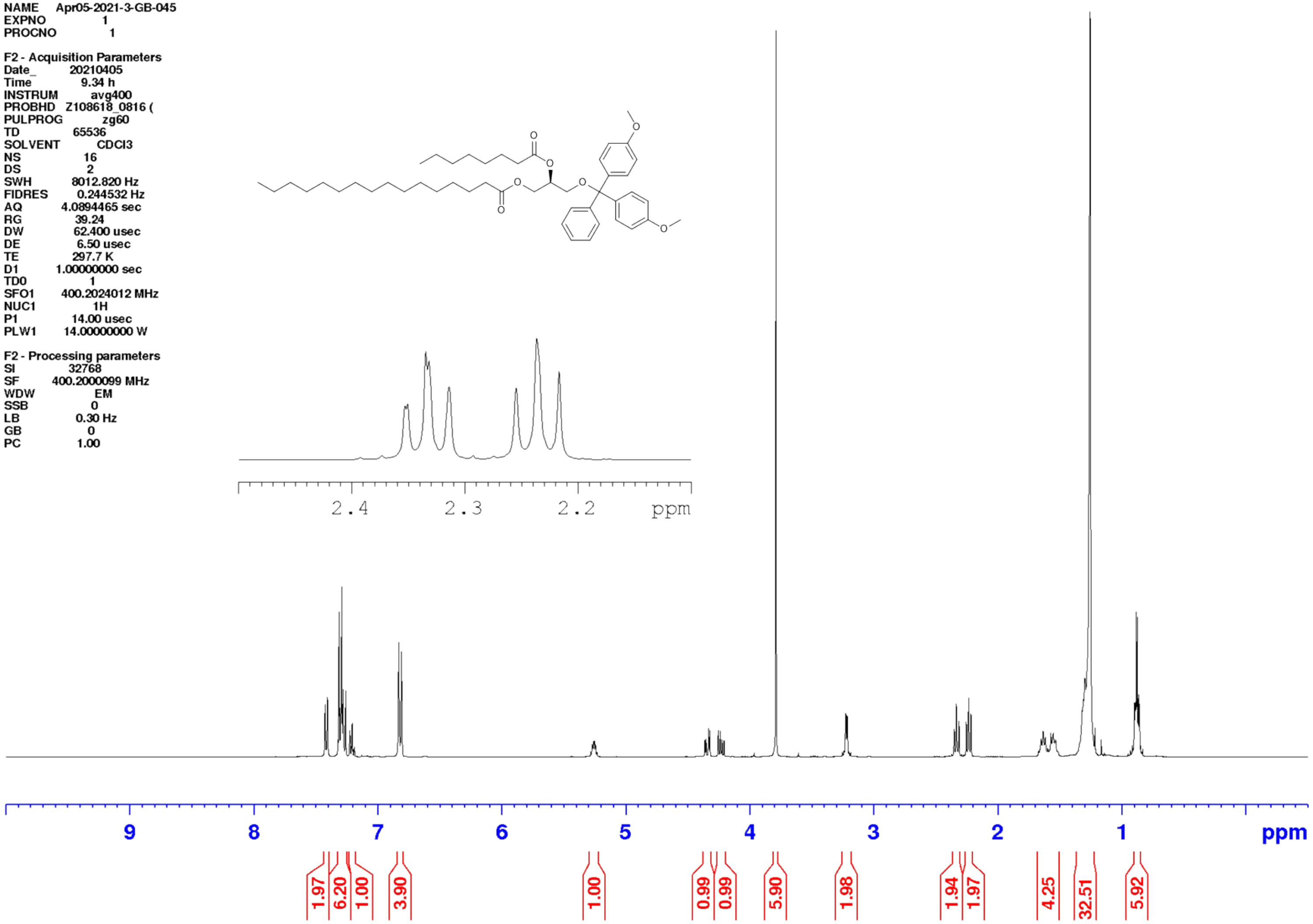

(+)-(2*S*)-3-[bis(4-methoxyphenyl)(phenyl)methoxy]-2-(octanoyloxy)propyl hexadecanoate (+)-S12 ^{13}C NMR

```
Current Data Parameters
NAME       Apr05-2021-3-GB-045
EXPNO                        2
PROCNO                       1

F2 - Acquisition Parameters
Date_               20210405
Time                   10.29 h
INSTRUM               avg400
PROBHD     Z108618_0816 (
PULPROG              zgpg30
TD                     32768
SOLVENT                CDCl3
NS                       512
DS                         4
SWH                26041.666 Hz
FIDRES              1.589457 Hz
AQ                 0.6291456 sec
RG                    206.87
DW                    19.200 usec
DE                      6.50 usec
TE                     298.8 K
D1                1.00000000 sec
D11               0.03000000 sec
TD0                        1
SFO1             100.6404331 MHz
NUC1                     13C
P0                      3.33 usec
P1                     10.00 usec
PLW1             56.00000000 W
SFO2             400.2016008 MHz
NUC2                      1H
CPDPRG[2             waltz16
PCPD2                  90.00 usec
PLW2             14.00000000 W
PLW12             0.33877000 W
PLW13             0.17039999 W

F2 - Processing parameters
SI                     32768
SF               100.6303589 MHz
WDW                       EM
SSB                        0
LB                      1.00 Hz
GB                         0
PC                      1.40
```

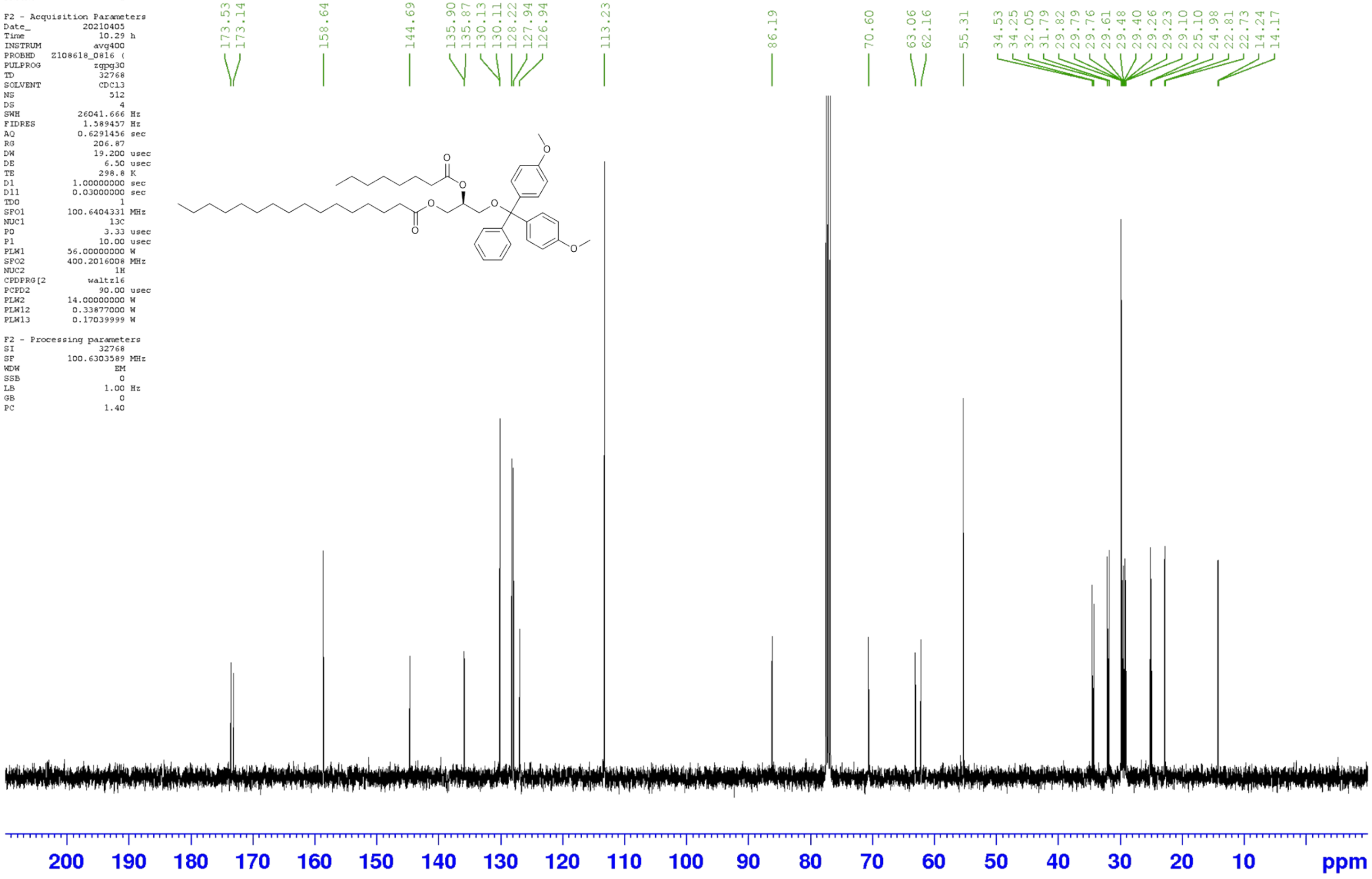

(+)-(2*S*)-3-[bis(4-methoxyphenyl)(phenyl)methoxy]-2-(octanoyloxy)propyl hexadecanoate (+)-S12 HRMS

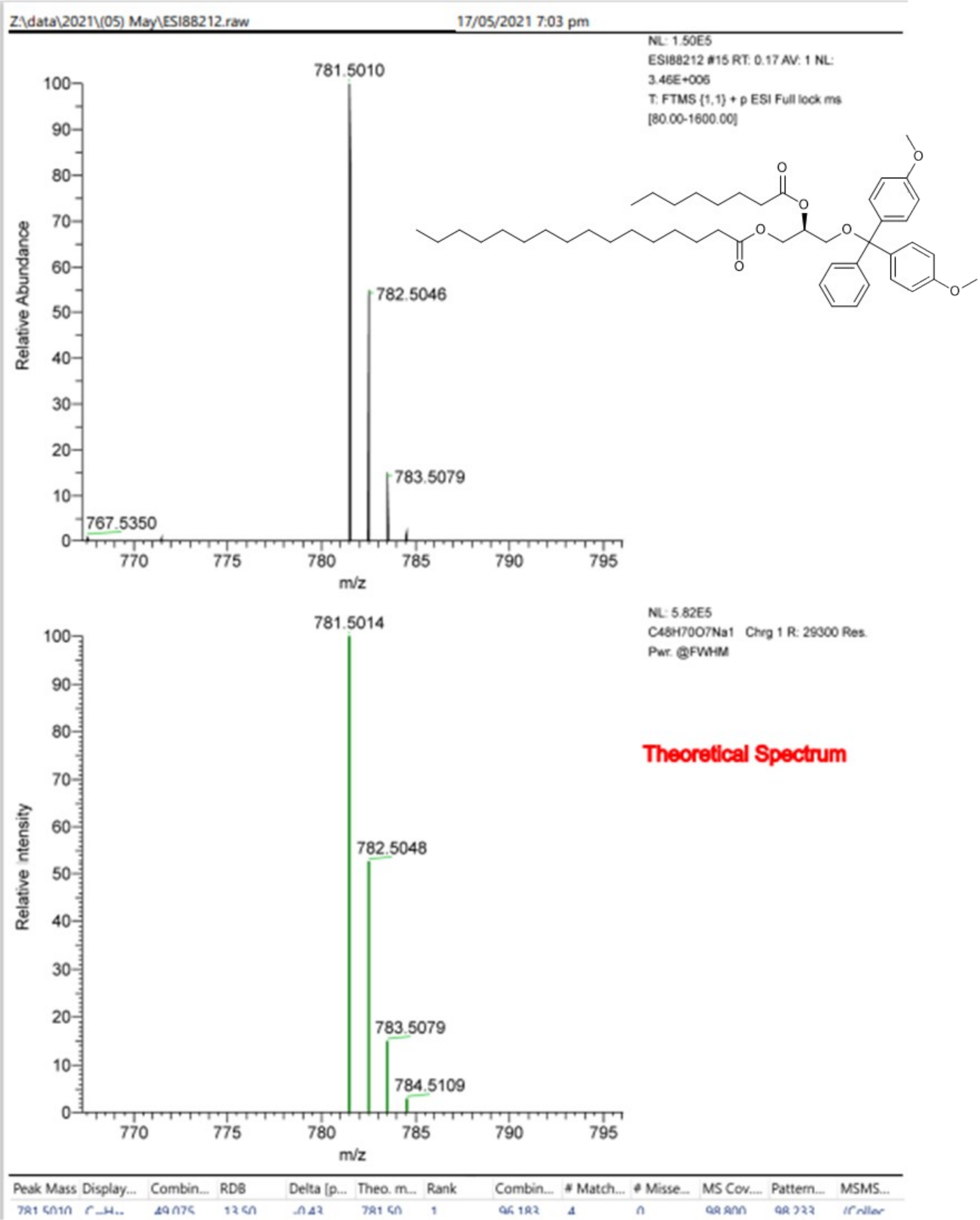

(+)-(2*S*)-1-[bis(4-methoxyphenyl)(phenyl)methoxy]-3-(octanoyloxy)propan-2-yl hexadecanoate (+)-S13 ^{1}H NMR

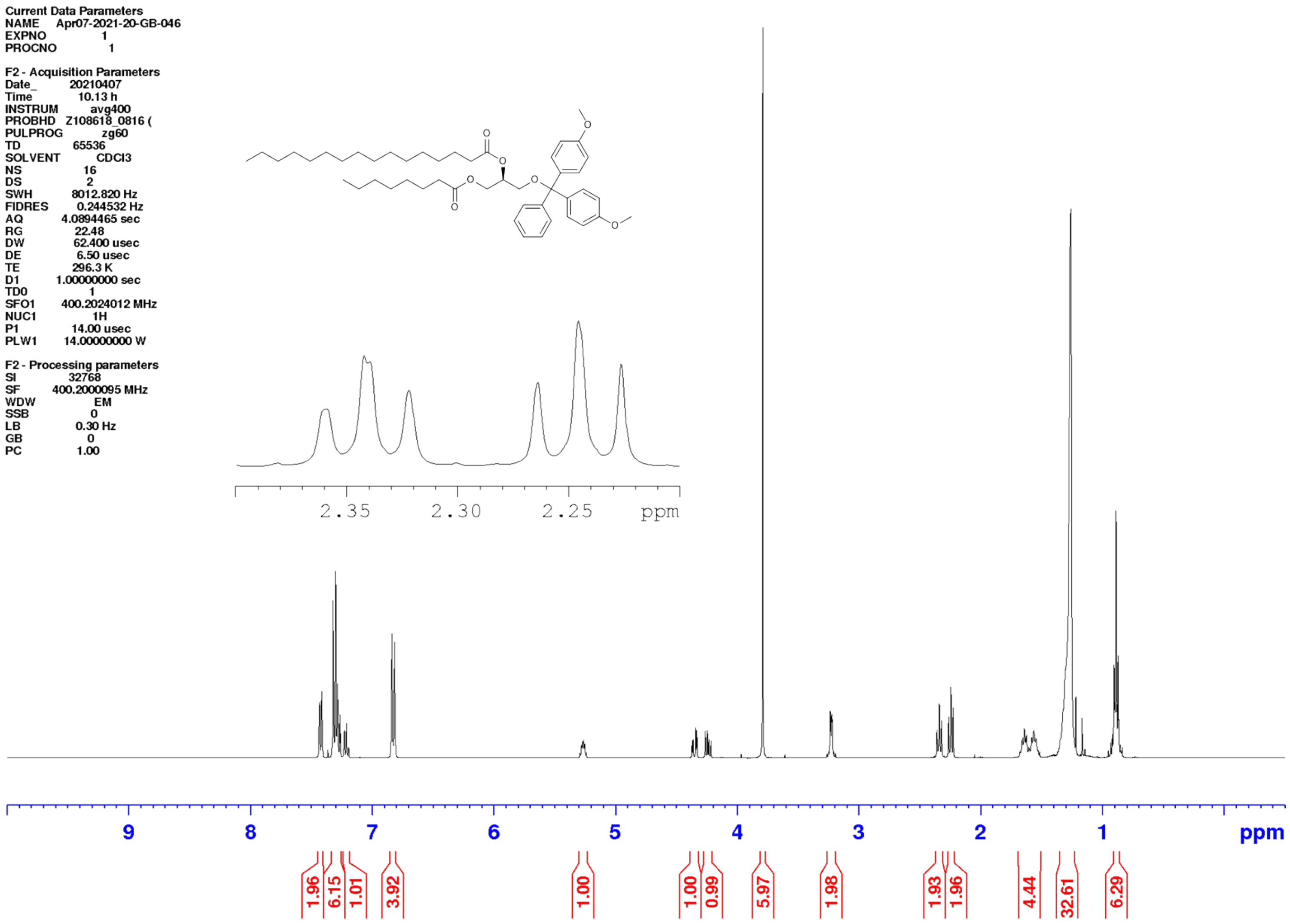

(+)-(2*S*)-1-[bis(4-methoxyphenyl)(phenyl)methoxy]-3-(octanoyloxy)propan-2-yl hexadecanoate (+)-S13 ^{13}C NMR

```
Current Data Parameters
NAME     Apr07-2021-20-GB-046
EXPNO                       2
PROCNO                      1

F2 - Acquisition Parameters
Date_                20210407
Time                  11.05 h
INSTRUM                avg400
PROBHD     Z108618_0816 (
PULPROG               zgpg30
TD                     32768
SOLVENT                CDCl3
NS                       512
DS                         4
SWH               26041.666 Hz
FIDRES             1.589457 Hz
AQ                0.6291456 sec
RG                    206.87
DW                    19.200 usec
DE                      6.50 usec
TE                     297.4 K
D1                1.00000000 sec
D11               0.03000000 sec
TD0                        1
SFO1             100.6404331 MHz
NUC1                     13C
P0                      3.33 usec
P1                     10.00 usec
PLW1             56.00000000 W
SFO2             400.2016008 MHz
NUC2                      1H
CPDPRG[2             waltz16
PCPD2                  90.00 usec
PLW2             14.00000000 W
PLW12             0.33877000 W
PLW13             0.17039999 W

F2 - Processing parameters
SI                     32768
SF               100.6303598 MHz
WDW                       EM
SSB                        0
LB                      1.00 Hz
GB                         0
PC                      1.40
```

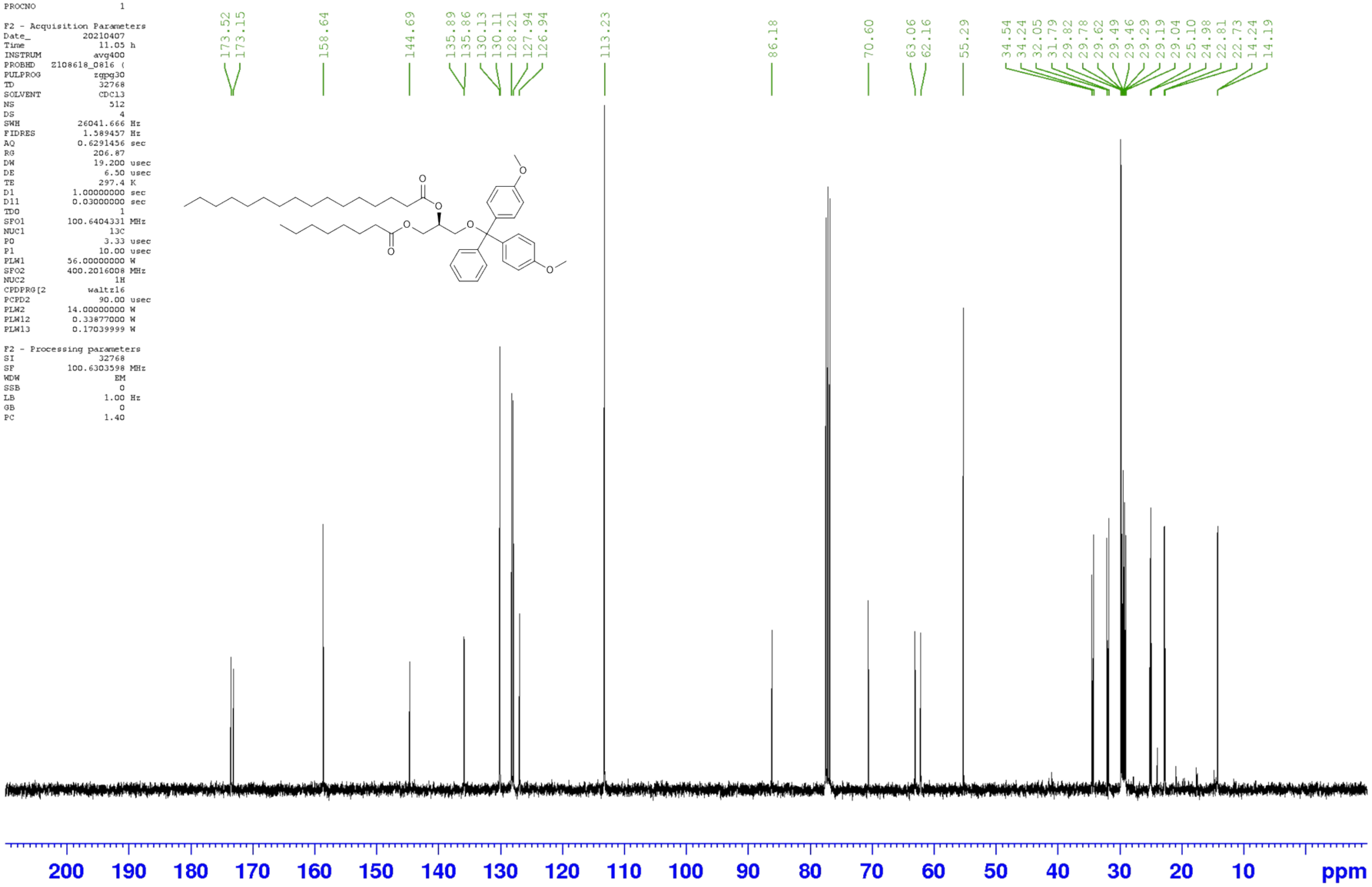

(+)-(2*S*)-1-[bis(4-methoxyphenyl)(phenyl)methoxy]-3-(octanoyloxy)propan-2-yl hexadecanoate (+)-S13 HMRS

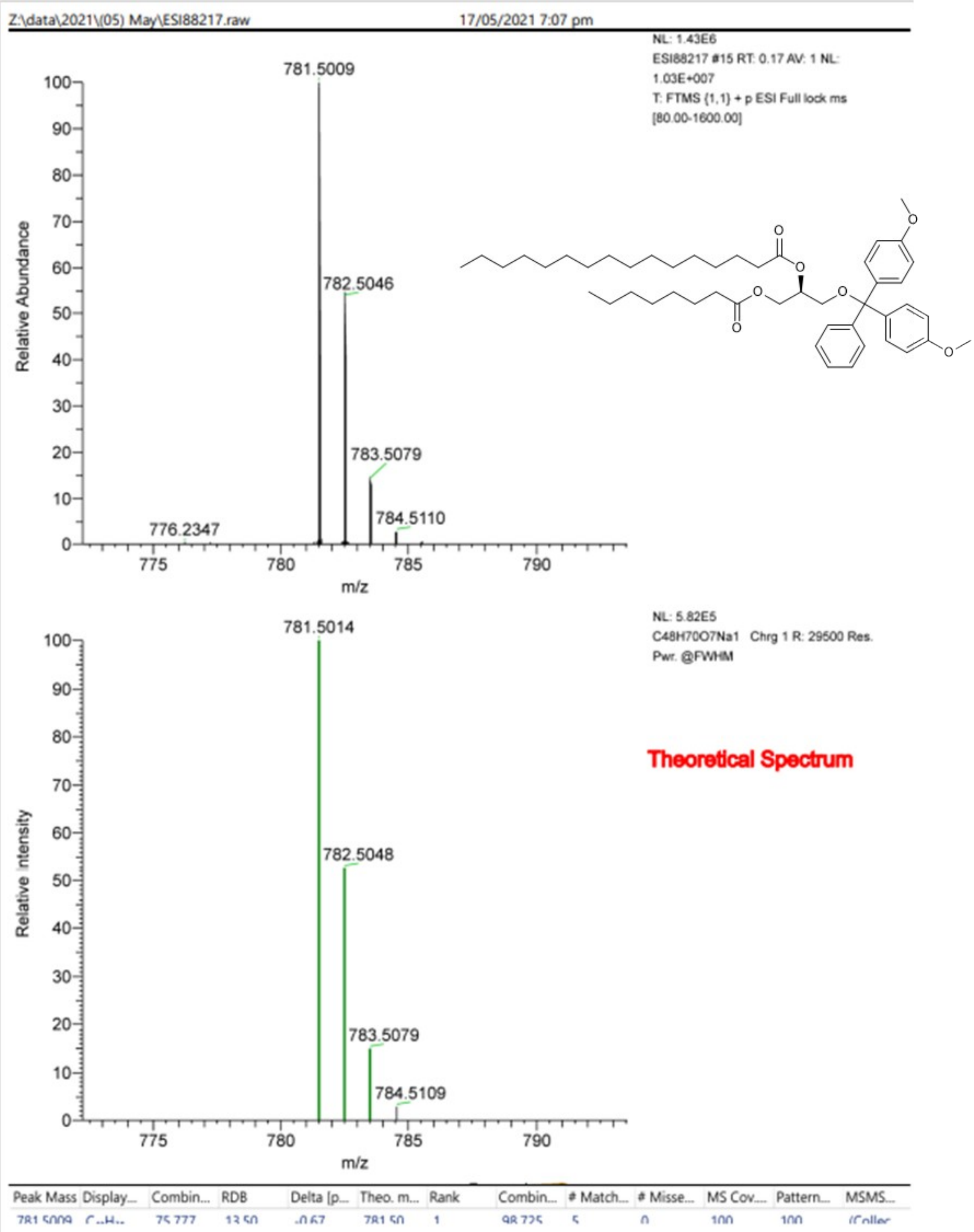

(–)-(2*S*)-3-hydroxy-2-(octanoyloxy)propyl hexadecanoate (–)-S14 ^{1}H NMR

Current Data Parameters
NAME gb710731807 (C16, C8 -OH)
EXPNO 1
PROCNO 1

F2 - Acquisition Parameters
Date_ 20230718
Time 20.43 h
INSTRUM Avance
PROBHD Z159656_0020 (
PULPROG zg30
TD 65536
SOLVENT CDCl3
NS 16
DS 2
SWH 11904.762 Hz
FIDRES 0.363304 Hz
AQ 2.7525120 sec
RG 64
DW 42.000 usec
DE 22.00 usec
TE 298.0 K
D1 1.00000000 sec
TD0 1
SFO1 600.4230021 MHz
NUC1 1H
P0 4.00 usec
P1 12.00 usec
PLW1 13.51200008 W

F2 - Processing parameters
SI 65536
SF 600.4200137 MHz
WDW EM
SSB 0
LB 0.30 Hz
GB 0
PC 1.00

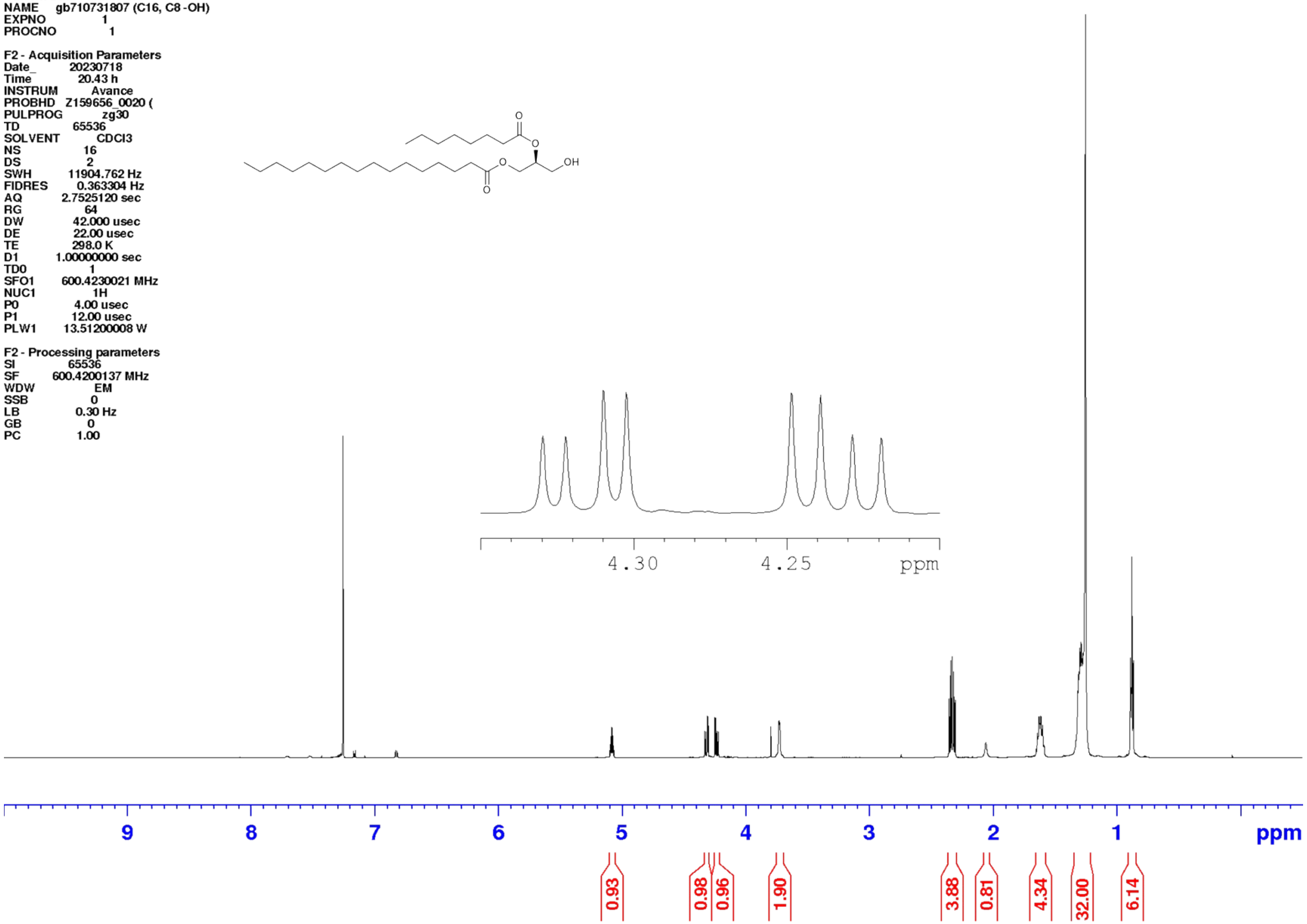

(–)-(2*S*)-3-hydroxy-2-(octanoyloxy)propyl hexadecanoate (–)-S14 ^{13}C NMR

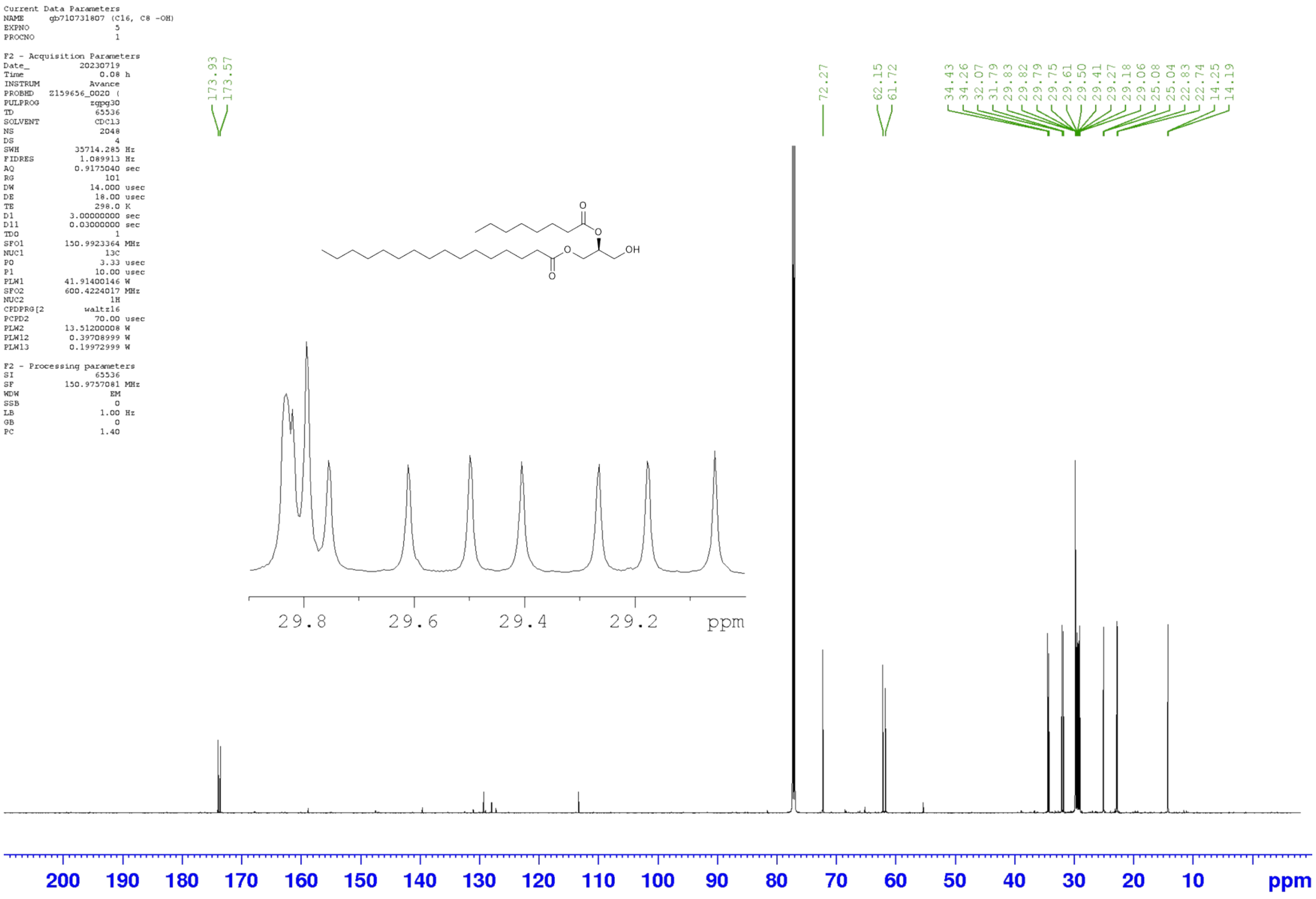

(–)-(2*S*)-3-hydroxy-2-(octanoyloxy)propyl hexadecanoate (–)-S14 HRMS

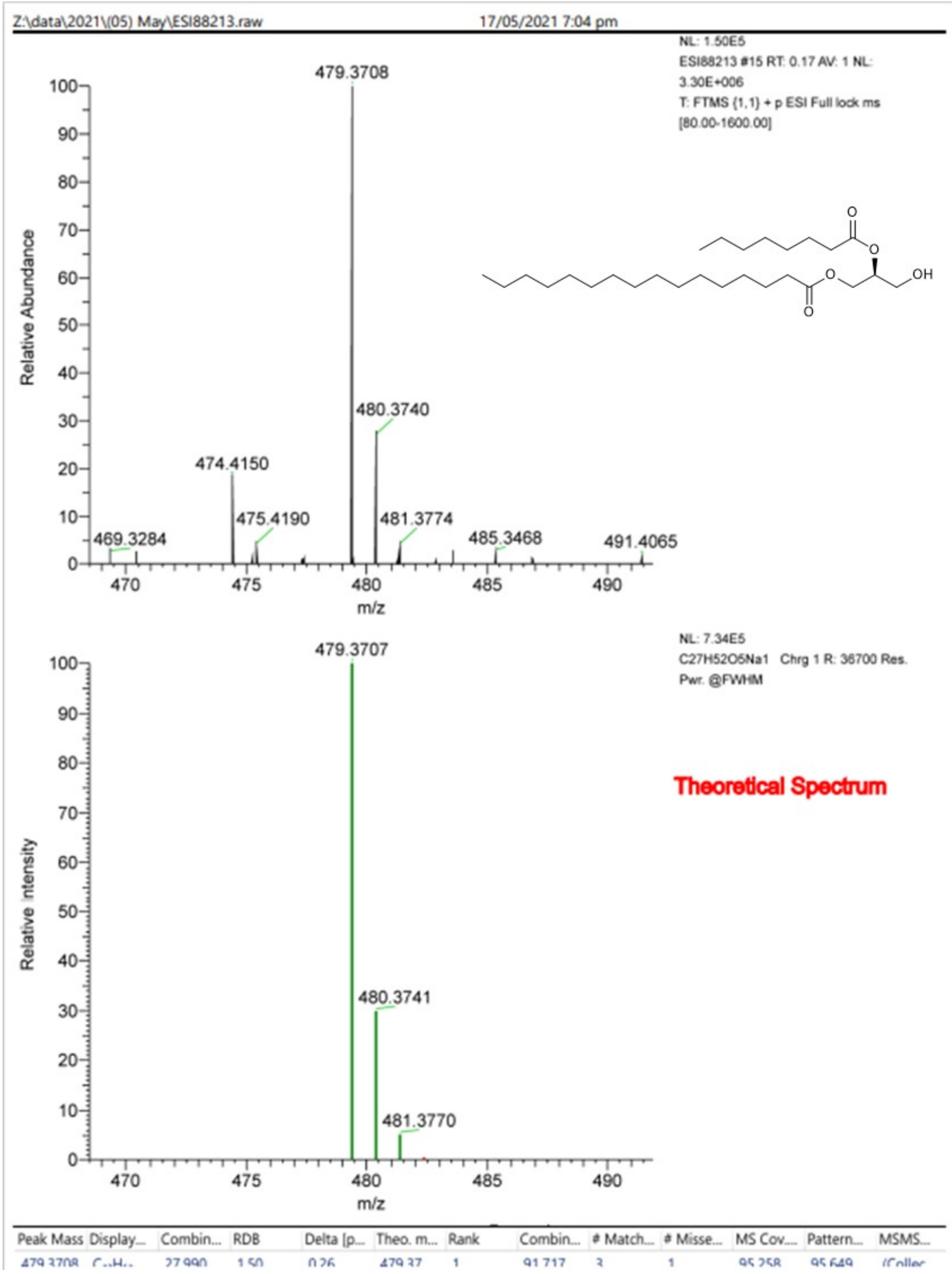

(2*S*)-1-hydroxy-3-(octanoyloxy)propan-2-yl hexadecanoate (–)-S15 ^{1}H NMR

Current Data Parameters
NAME Apr08-2021-6-GB-050
EXPNO 1
PROCNO 1

F2 - Acquisition Parameters
Date_ 20210408
Time 9.49 h
INSTRUM avg400
PROBHD Z108618_0816 (
PULPROG zg60
TD 65536
SOLVENT CDCl3
NS 16
DS 2
SWH 8012.820 Hz
FIDRES 0.244532 Hz
AQ 4.0894465 sec
RG 91.39
DW 62.400 usec
DE 6.50 usec
TE 297.3 K
D1 1.00000000 sec
TD0 1
SFO1 400.2024012 MHz
NUC1 1H
P1 14.00 usec
PLW1 14.00000000 W

F2 - Processing parameters
SI 32768
SF 400.2000098 MHz
WDW EM
SSB 0
LB 0.30 Hz
GB 0
PC 1.00

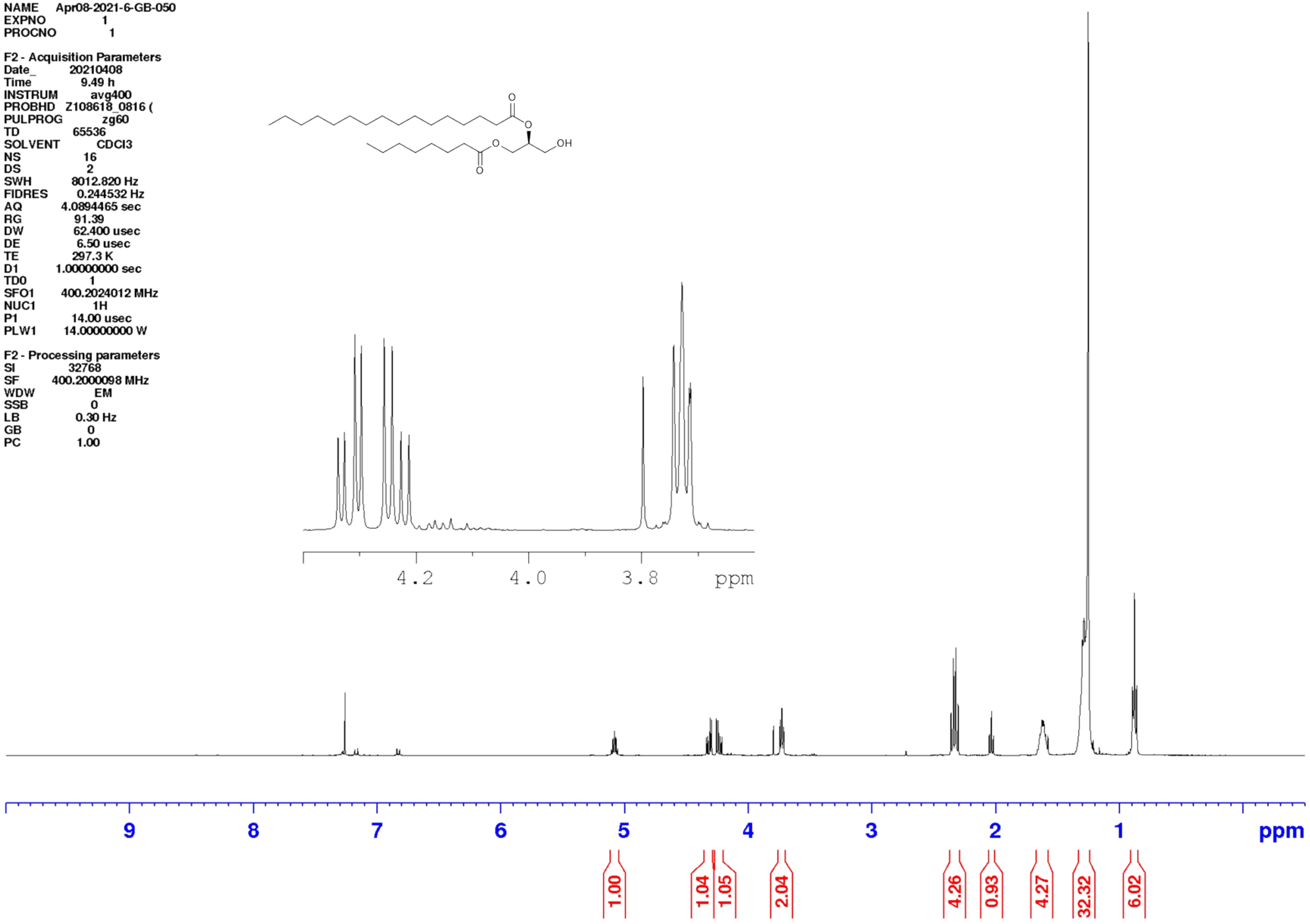

(2*S*)-1-hydroxy-3-(octanoyloxy)propan-2-yl hexadecanoate (–)-S15 ^{13}C NMR

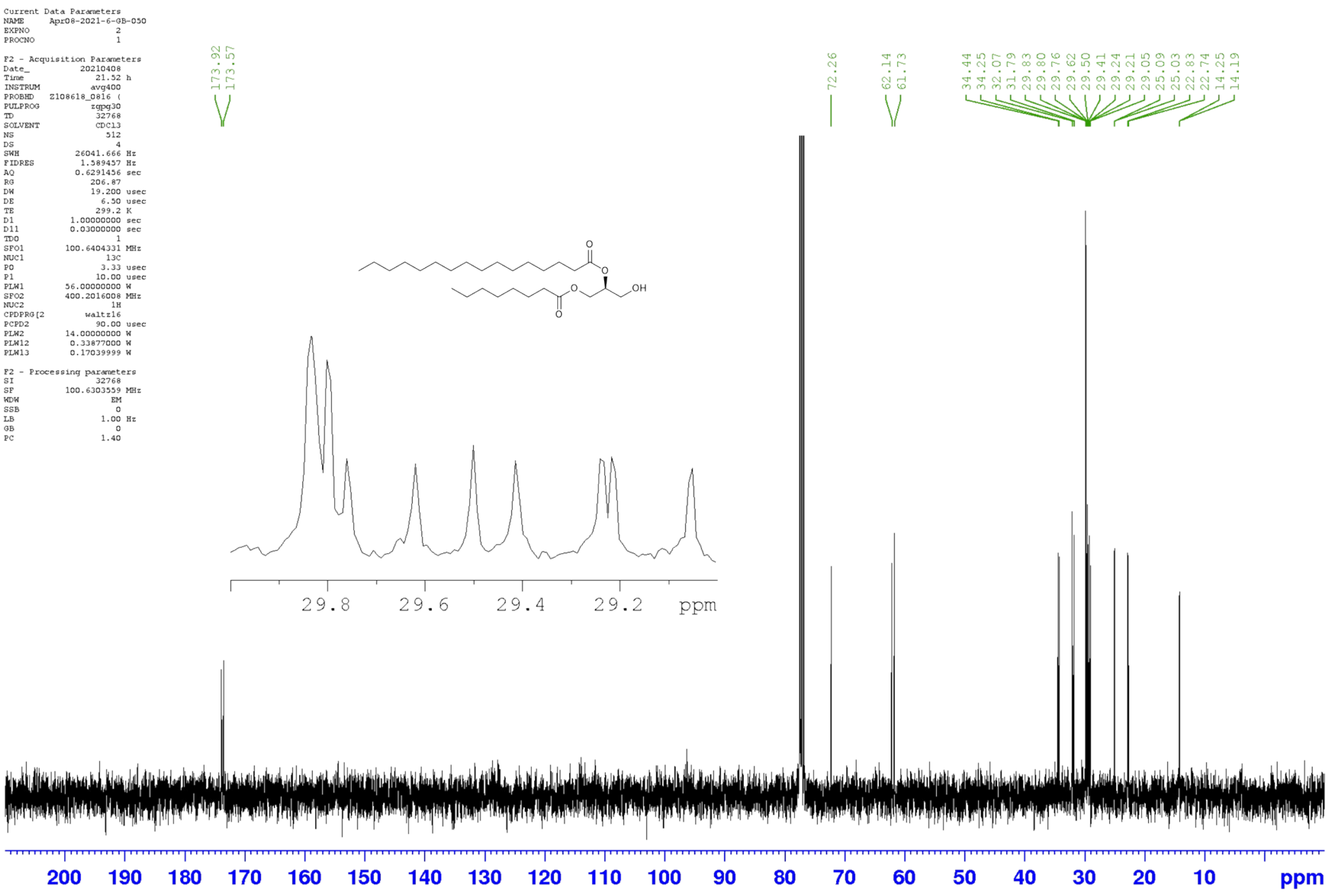

(2*S*)-1-hydroxy-3-(octanoyloxy)propan-2-yl hexadecanoate (–)-S15 HRMS

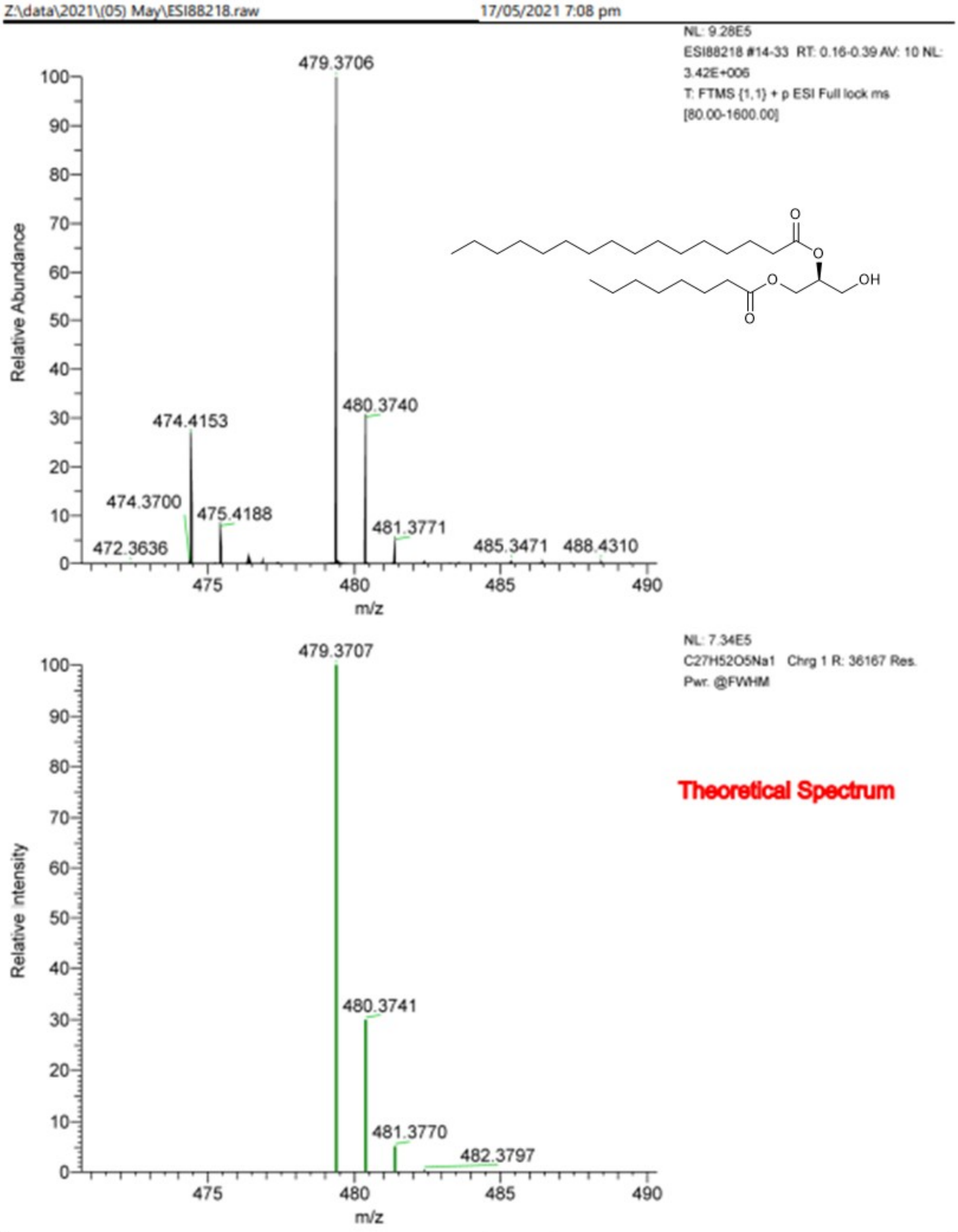

Peak Mass	Display...	Combin...	RDB	Delta [p...	Theo. m...	Rank	Combin...	# Match...	# Misse...	MS Cov....	Pattern...	MSMS...
479.3706	[illegible]	44.273	1.50	-0.20	479.37	1	94.252	4	0	97.028	99.504	[illegible]

(+)-(2*R*)-3-({(benzyloxy)[di(propan-2-yl)amino]phosphanyl}oxy)-2-(octanoyloxy)propyl hexadecanoate (+)-S16 ^{1}H NMR

Current Data Parameters
NAME Apr09-2021-28-GB-048t1-3 (C16-C8 phosphoramidite)
EXPNO 1
PROCNO 1

F2 - Acquisition Parameters
Date_ 20210409
Time 10.46 h
INSTRUM avh400
PROBHD Z108618_0873 (
PULPROG zg30
TD 65536
SOLVENT CDCl3
NS 16
DS 2
SWH 8012.820 Hz
FIDRES 0.244532 Hz
AQ 4.0894465 sec
RG 50.46
DW 62.400 usec
DE 6.50 usec
TE 298.5 K
D1 1.00000000 sec
TD0 1
SFO1 400.1324008 MHz
NUC1 1H
P1 14.00 usec
PLW1 14.36999989 W

F2 - Processing parameters
SI 32768
SF 400.1300099 MHz
WDW EM
SSB 0
LB 0.30 Hz
GB 0
PC 1.00

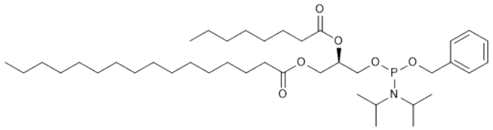

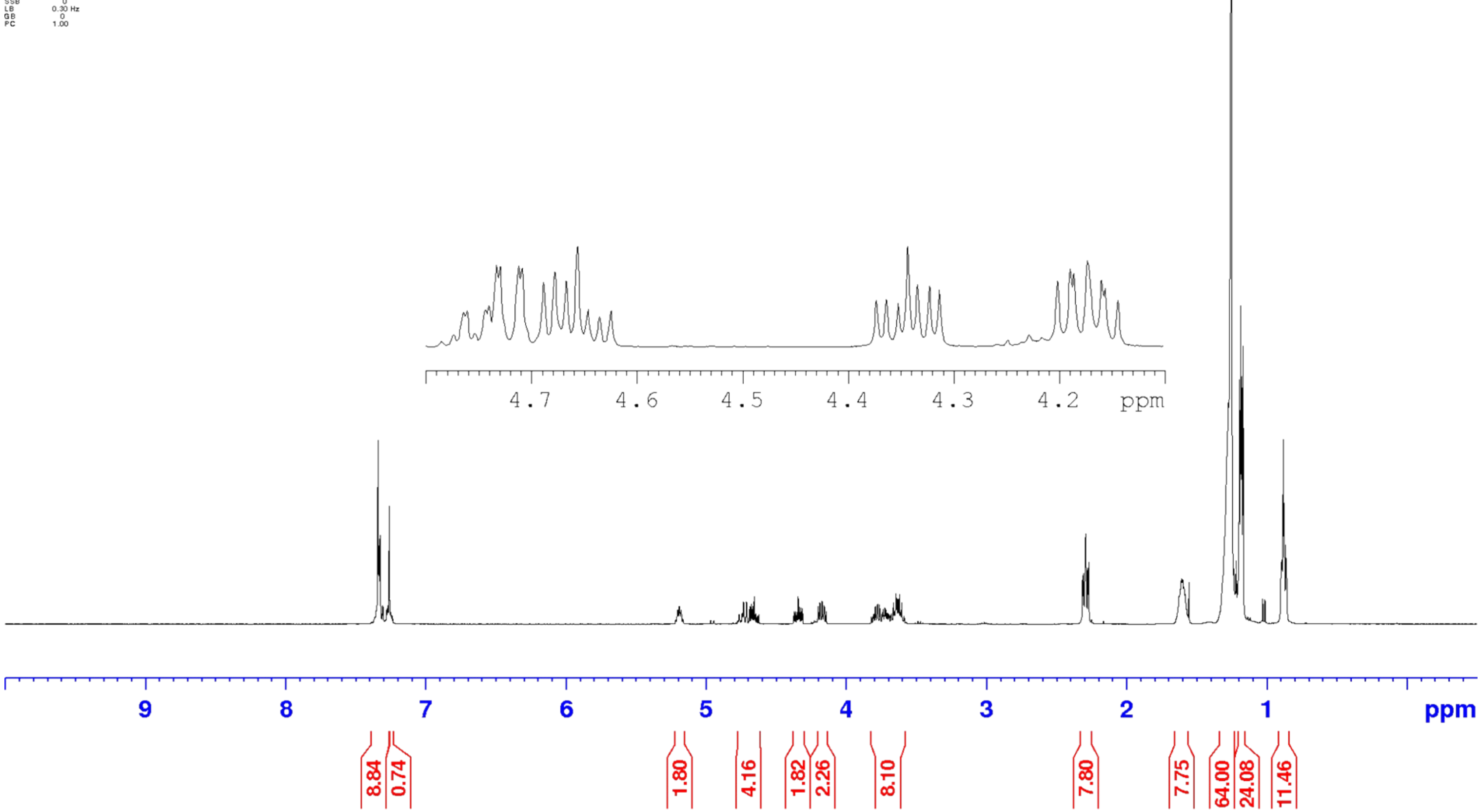

(+)-(2*R*)-3-({(benzyloxy)[di(propan-2-yl)amino]phosphanyl}oxy)-2-(octanoyloxy)propyl hexadecanoate (+)-S16 ^{31}P NMR

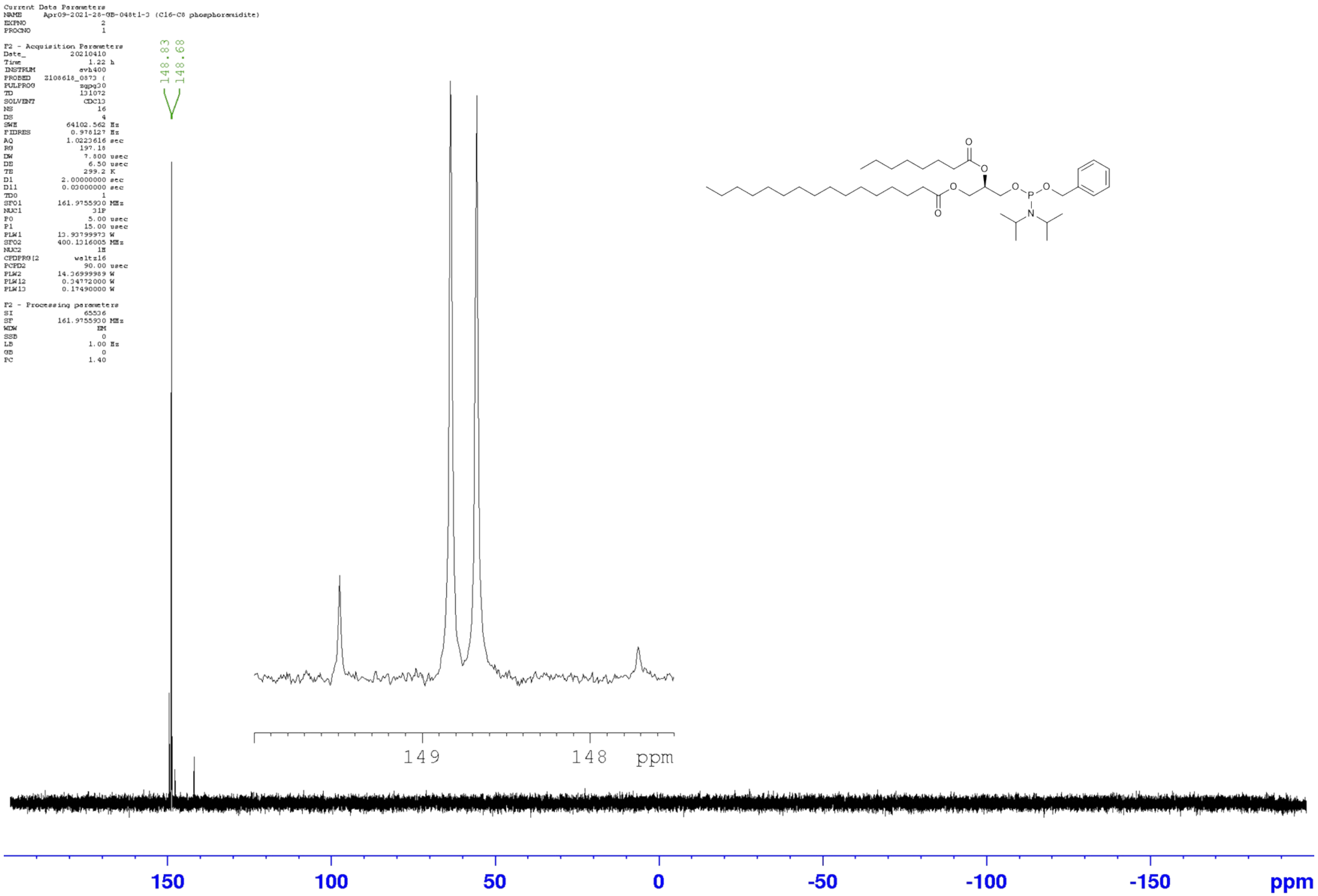

(+)-(2*R*)-3-({(benzyloxy)[di(propan-2-yl)amino]phosphanyl}oxy)-2-(octanoyloxy)propyl hexadecanoate (+)-S16 HRMS

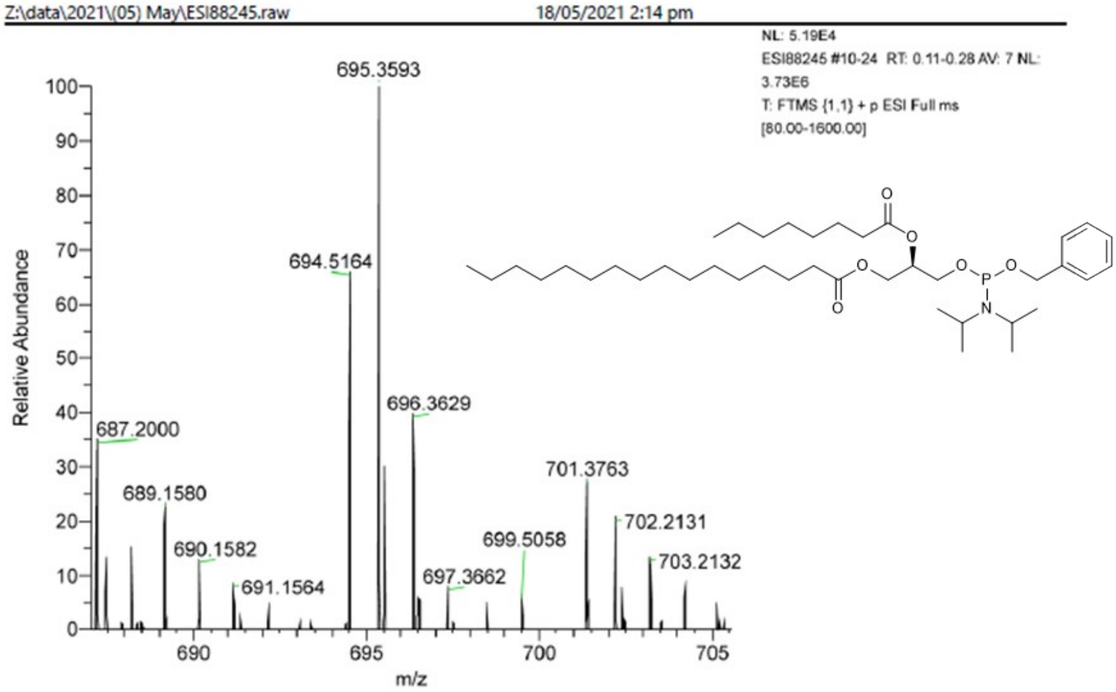

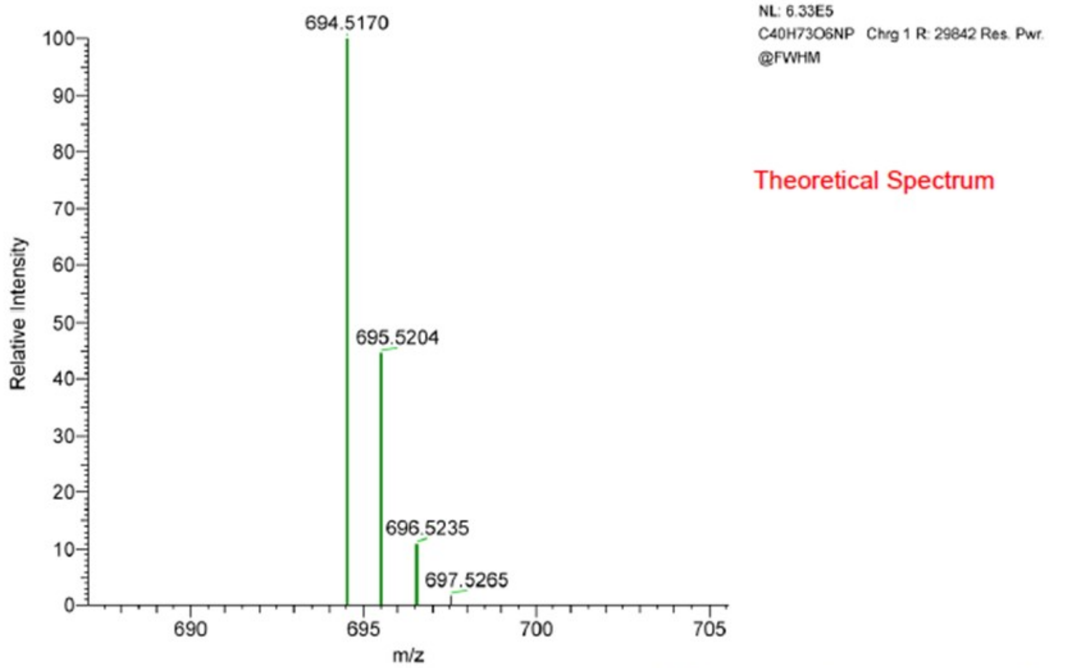

Peak Mass	Display...	Combin...	RDB	Delta [p...	Theo. m...	Rank	Combin...	# Match...	# Misse...	MS Cov....	Pattern...	MSMS...
694.5164	[illegible]	60.573	5.50	-0.82	694.51	1	42.316	3	0	41.302	100	(Collec

(+)-(7*R*)-4-(benzyloxy)-2-methyl-10-oxo-3-(propan-2-yl)-5,9-dioxa-3-aza-4-phosphaheptadecan-7-yl hexadecanoate (+)-S17 ^{1}H NMR

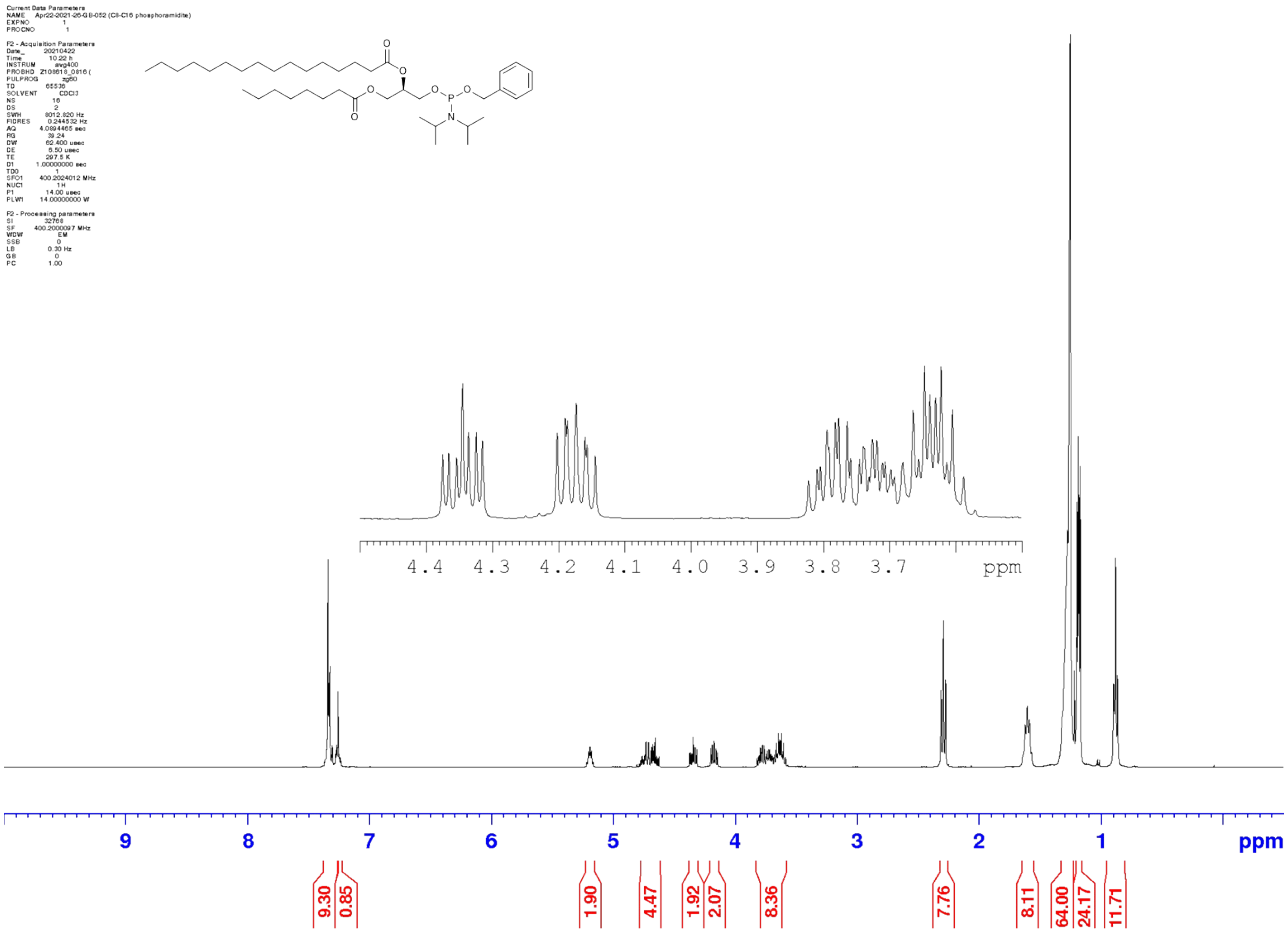

(+)-(7*R*)-4-(benzyloxy)-2-methyl-10-oxo-3-(propan-2-yl)-5,9-dioxa-3-aza-4-phosphaheptadecan-7-yl hexadecanoate (+)-S17 ^{31}P NMR

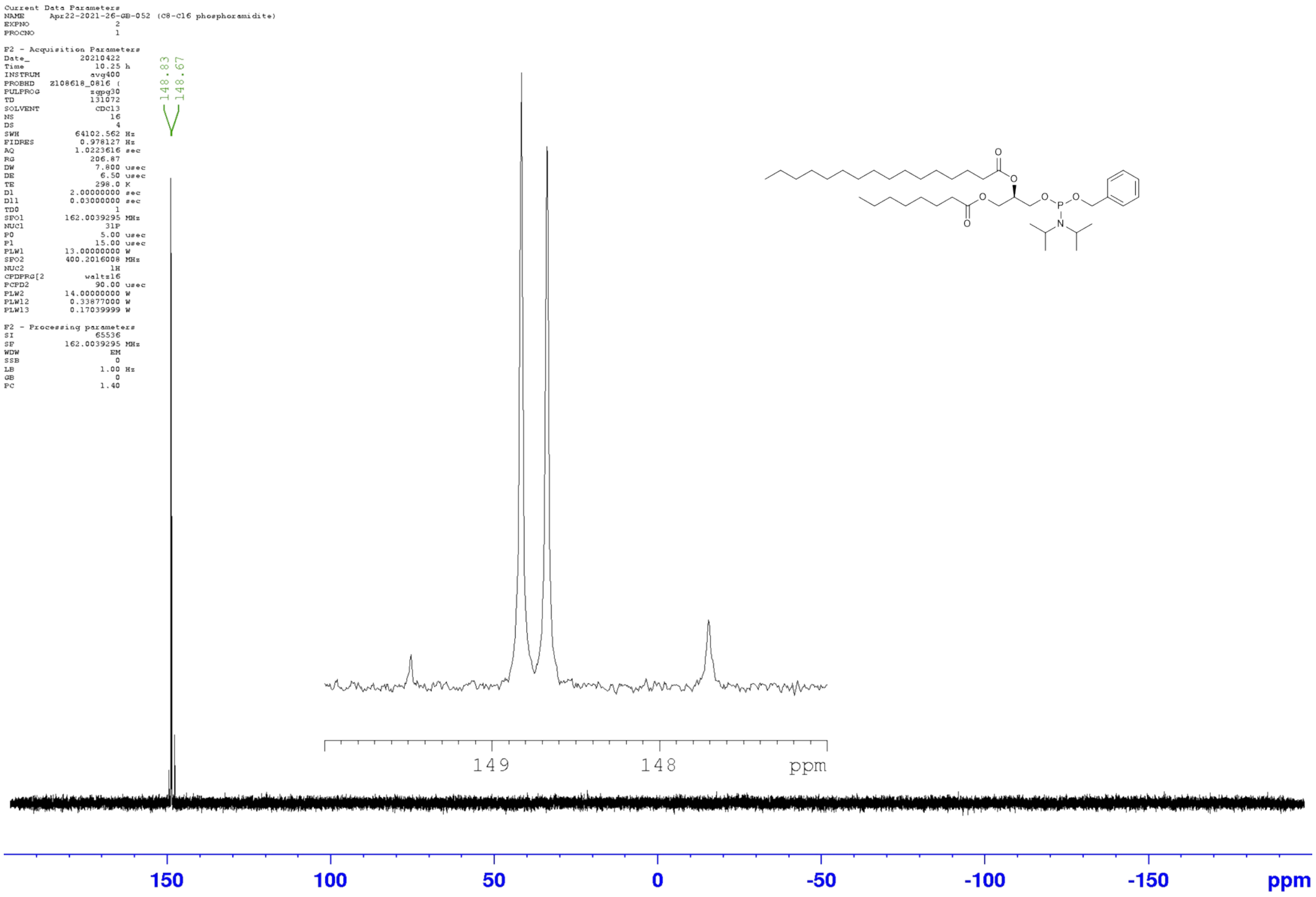

(+)-(2*R*)-3-{[(benzyloxy)({(3a*R*,4*S*,5*R*,6*S*,7*R*,7a*R*)-5,7-bis(benzyloxy)-6-[(3-oxo-1,5-dihydro-3*H*-2,4,3λ5-benzodioxaphosphepin-3-yl)oxy]hexahydrospiro[1,3-benzodioxole-2,1'-cyclopentan]-4-yl}oxy)phosphoryl]oxy}-2-(octanoyloxy)propyl hexadecanoate (+)-S18 ^{1}H NMR

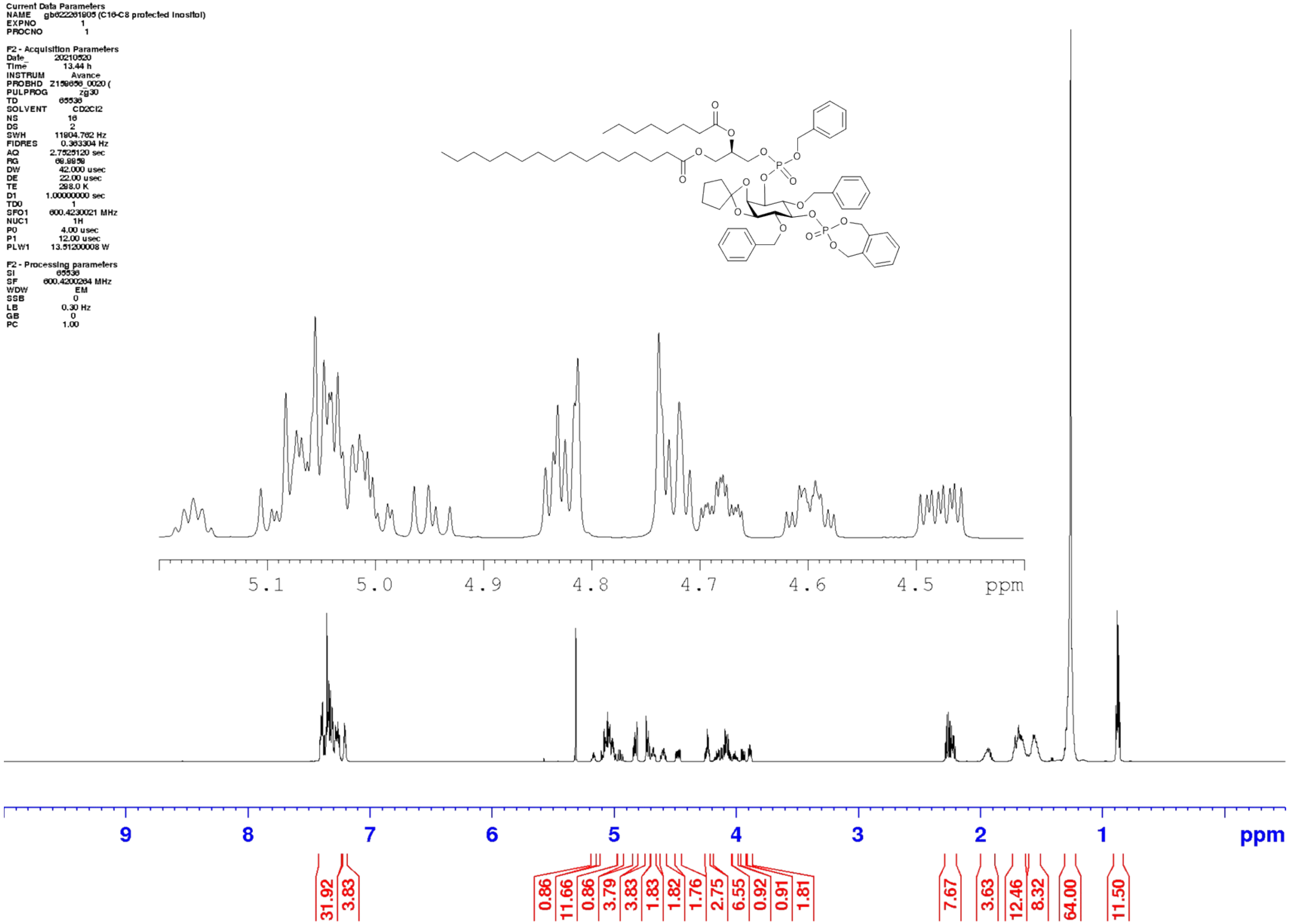

(+)-(2*R*)-3-{[(benzyloxy)({(3a*R*,4*S*,5*R*,6*S*,7*R*,7a*R*)-5,7-bis(benzyloxy)-6-[(3-oxo-1,5-dihydro-3*H*-2,4,3λ5-benzodioxaphosphepin-3-yl)oxy]hexahydrospiro[1,3-benzodioxole-2,1'-cyclopentan]-4-yl}oxy)phosphoryl]oxy}-2-(octanoyloxy)propyl hexadecanoate (+)-S18 ^{31}P NMR

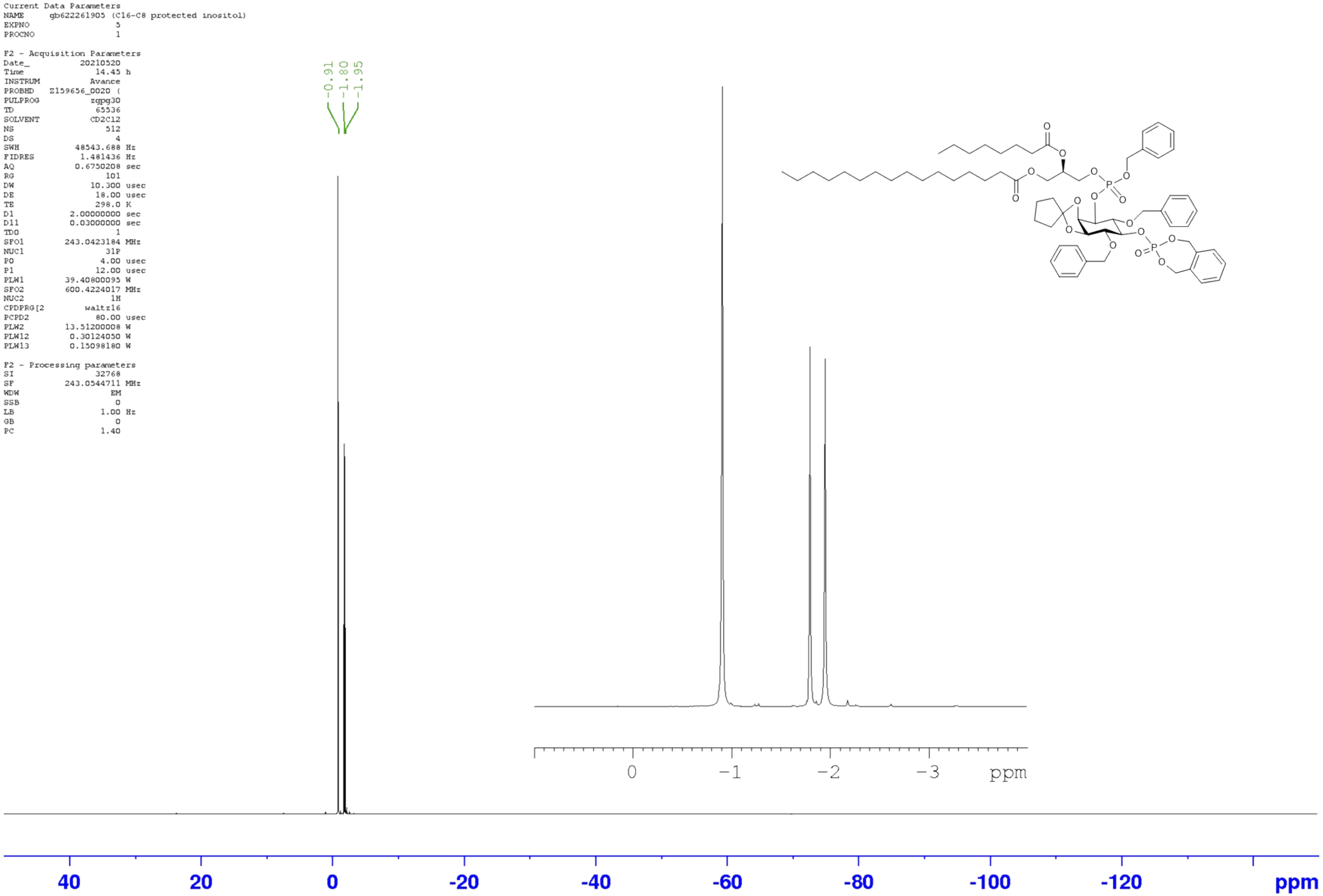

(+)-(2*R*)-3-{[(benzyloxy)({(3a*R*,4*S*,5*R*,6*S*,7*R*,7a*R*)-5,7-bis(benzyloxy)-6-[(3-oxo-1,5-dihydro-3*H*-2,4,3λ5-benzodioxaphosphepin-3-yl)oxy]hexahydrospiro[1,3-benzodioxole-2,1'-cyclopentan]-4-yl}oxy)phosphoryl]oxy}-2-(octanoyloxy)propyl hexadecanoate (+)-S18 ^{13}C NMR

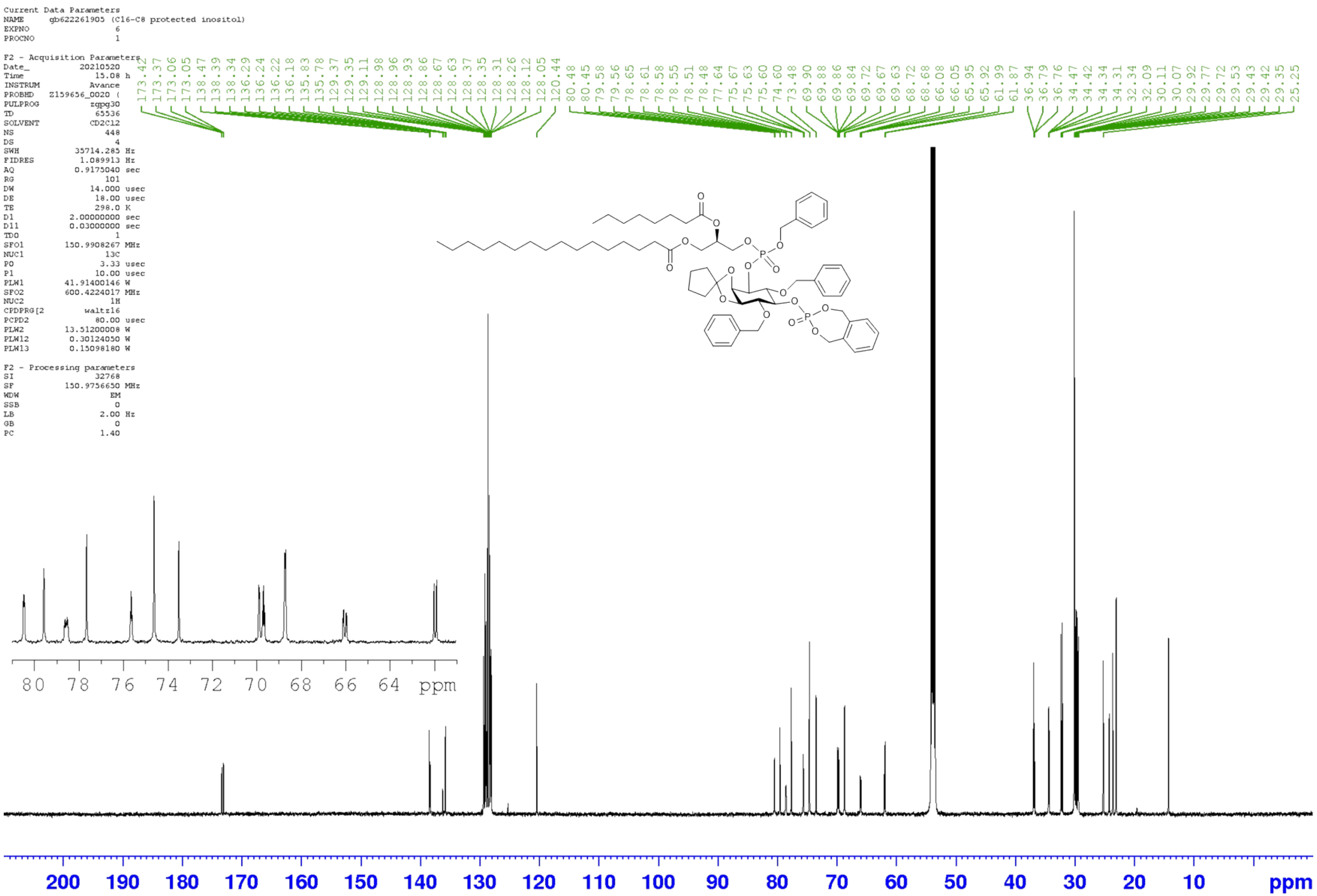

(+)-(2*R*)-3-{[(benzyloxy)({(3a*R*,4*S*,5*R*,6*S*,7*R*,7a*R*)-5,7-bis(benzyloxy)-6-[(3-oxo-1,5-dihydro-3*H*-2,4,3λ5-benzodioxaphosphepin-3-yl)oxy]hexahydrospiro[1,3-benzodioxole-2,1'-cyclopentan]-4-yl}oxy)phosphoryl]oxy}-2-(octanoyloxy)propyl hexadecanoate (+)-S18 HPLC 254nm

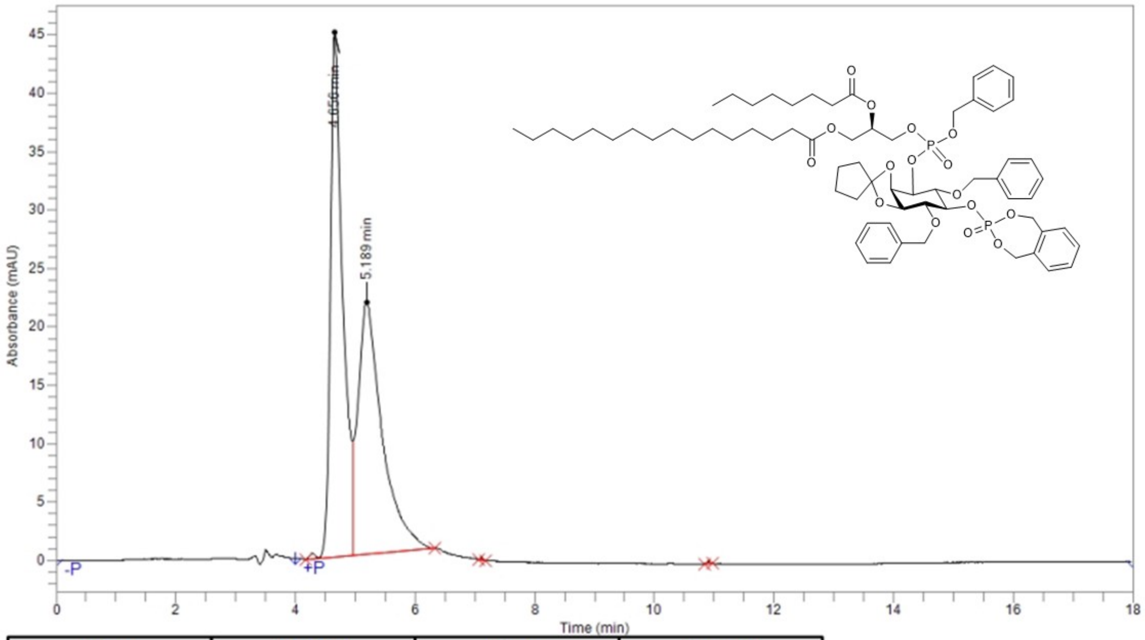

Time	Height	Area	Area %
4.278	477.3	2,996.1	0.24
4.656	44,957.3	649,405.9	51.36
5.189	21,603.0	610,963.4	48.32
7.115	164.2	432.9	0.03
10.910	231.5	683.8	0.05
**Total**		1,264,482.1	100.00

(+)-(2*R*)-3-{[(benzyloxy)({(3a*R*,4*S*,5*R*,6*S*,7*R*,7a*R*)-5,7-bis(benzyloxy)-6-[(3-oxo-1,5-dihydro-3*H*-2,4,3λ5-benzodioxaphosphepin-3-yl)oxy]hexahydrospiro[1,3-benzodioxole-2,1'-cyclopentan]-4-yl}oxy)phosphoryl]oxy}-2-(octanoyloxy)propyl hexadecanoate (+)-S18 HPLC 220nm

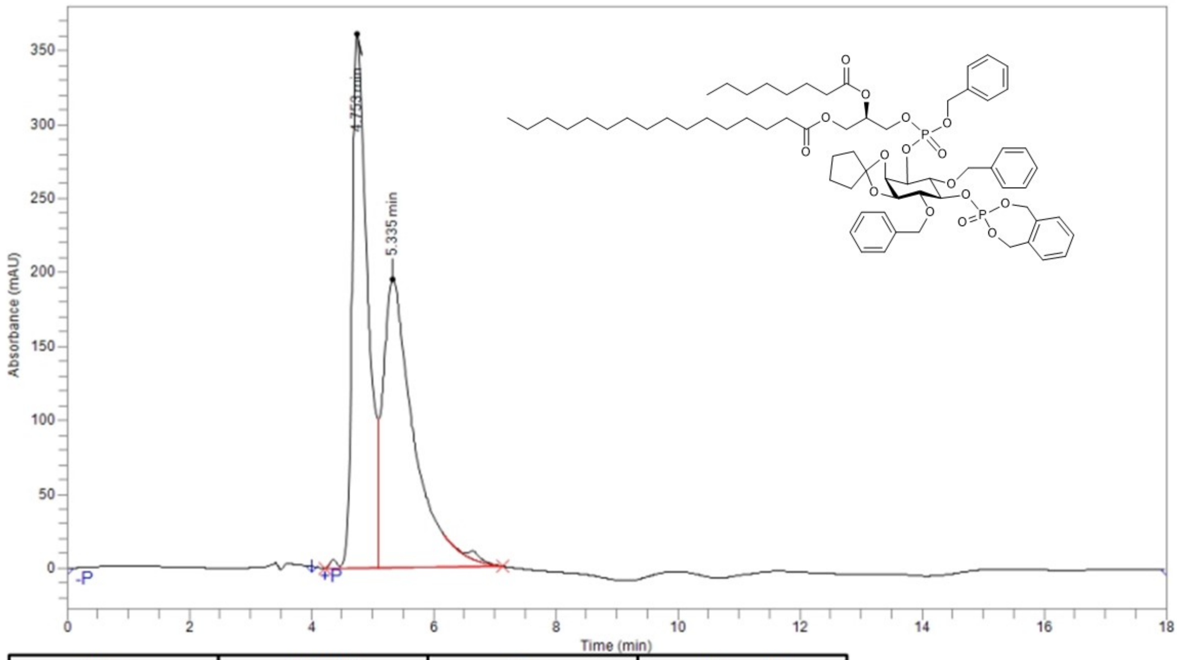

Time	Height	Area	Area %
4.355	6,102.3	45,838.7	0.34
4.753	361,524.5	6,326,487.7	47.36
5.335	194,836.1	6,893,991.7	51.61
6.636	5,553.0	91,593.1	0.69
**Total**		13,357,911.2	100.00

(+)-(2*R*)-3-{[(benzyloxy)({(3a*R*,4*S*,5*R*,6*S*,7*R*,7a*R*)-5,7-bis(benzyloxy)-6-[(3-oxo-1,5-dihydro-3*H*-2,4,3λ5-benzodioxaphosphepin-3-yl)oxy]hexahydrospiro[1,3-benzodioxole-2,1'-cyclopentan]-4-yl}oxy)phosphoryl]oxy}-2-(octanoyloxy)propyl hexadecanoate (+)-S18 HRMS

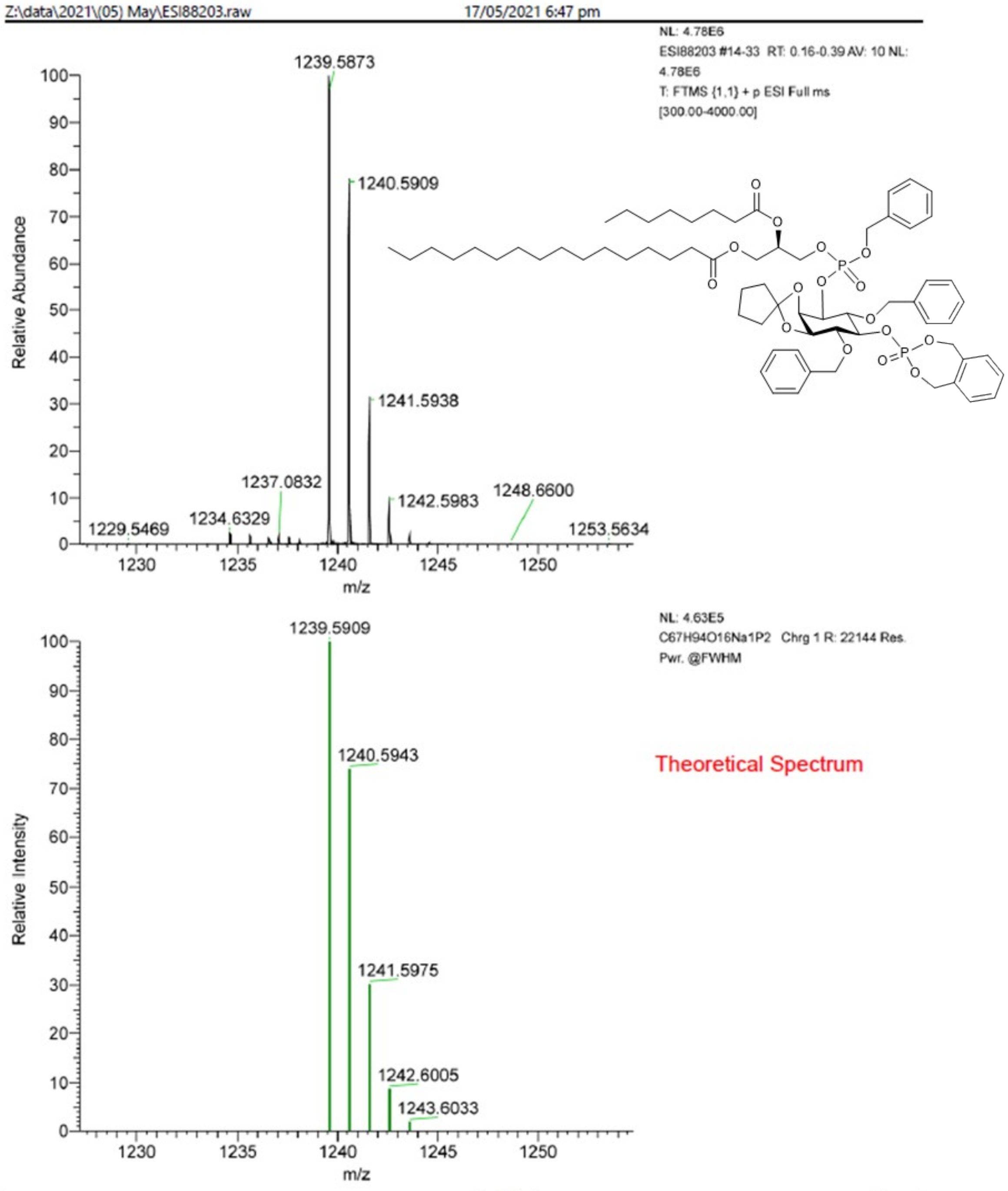

Peak Mass	Display...	Combin...	RDB	Delta (p...	Theo. m...	Rank	Combin...	# Match...	# Misse...	MS Cov....	Pattern...	MSMS...
1239.5	[illegible]	58.787	21.50	-2.94	1239.5	1	94.329	6	0	96.304	100	(Coller

(+)-(6*R*)-3-({(3a*R*,4*S*,5*R*,6*S*,7*R*,7a*R*)-5,7-bis(benzyloxy)-6-[(3-oxo-1,5-dihydro-3*H*-2,4,3λ5-benzodioxaphosphepin-3-yl)oxy]hexahydrospiro[1,3-benzodioxole-2,1'-cyclopentan]-4-yl}oxy)-3,9-dioxo-1-phenyl-2,4,8-trioxa-3λ5-phosphahexadecan-6-yl hexadecanoate (+)-S19 ¹H NMR

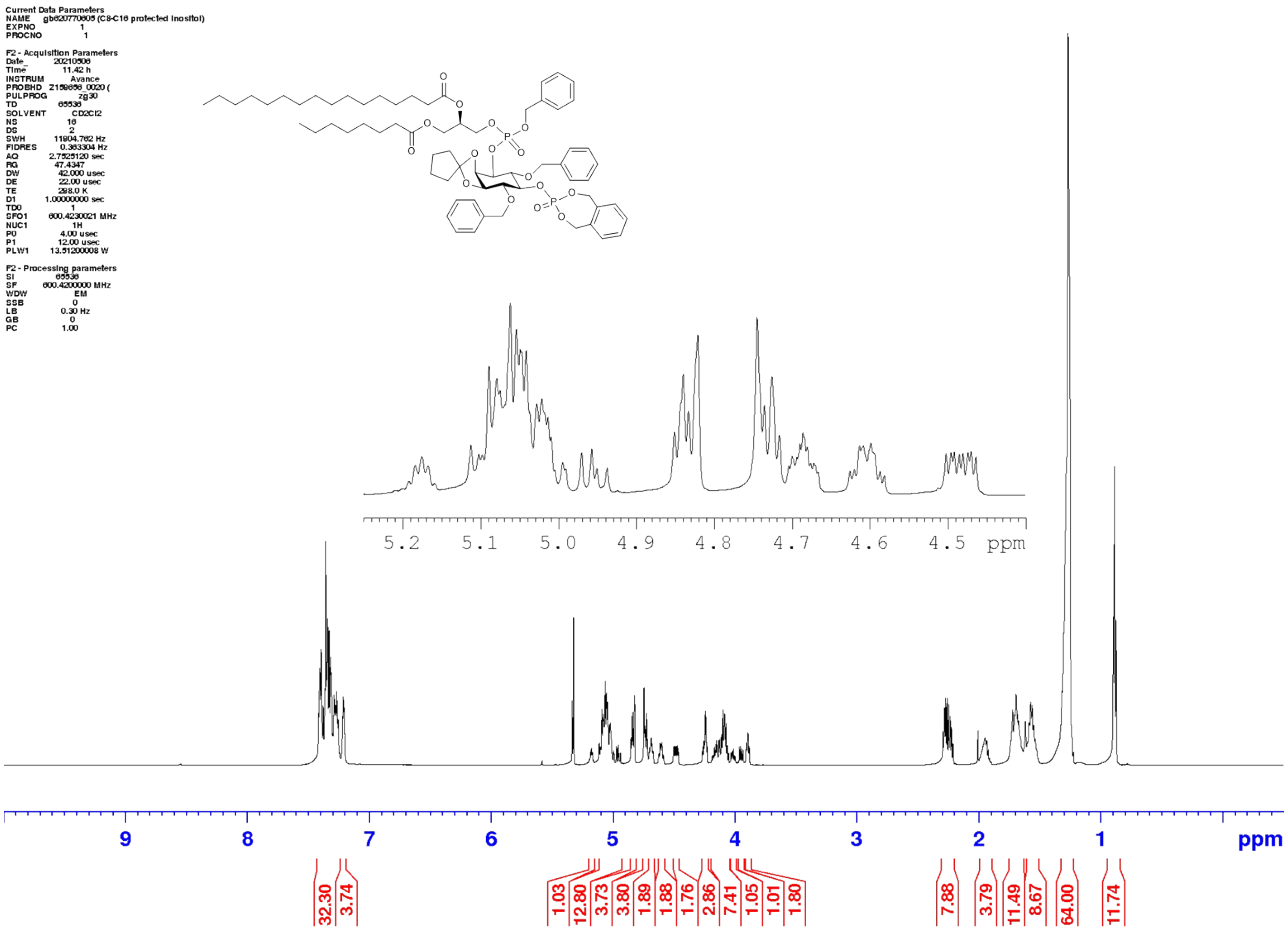

(+)-(6*R*)-3-({(3a*R*,4*S*,5*R*,6*S*,7*R*,7a*R*)-5,7-bis(benzyloxy)-6-[(3-oxo-1,5-dihydro-3*H*-2,4,3λ5-benzodioxaphosphepin-3-yl)oxy]hexahydrospiro[1,3-benzodioxole-2,1'-cyclopentan]-4-yl}oxy)-3,9-dioxo-1-phenyl-2,4,8-trioxa-3λ5-phosphahexadecan-6-yl hexadecanoate (+)-S19 ^{31}P NMR

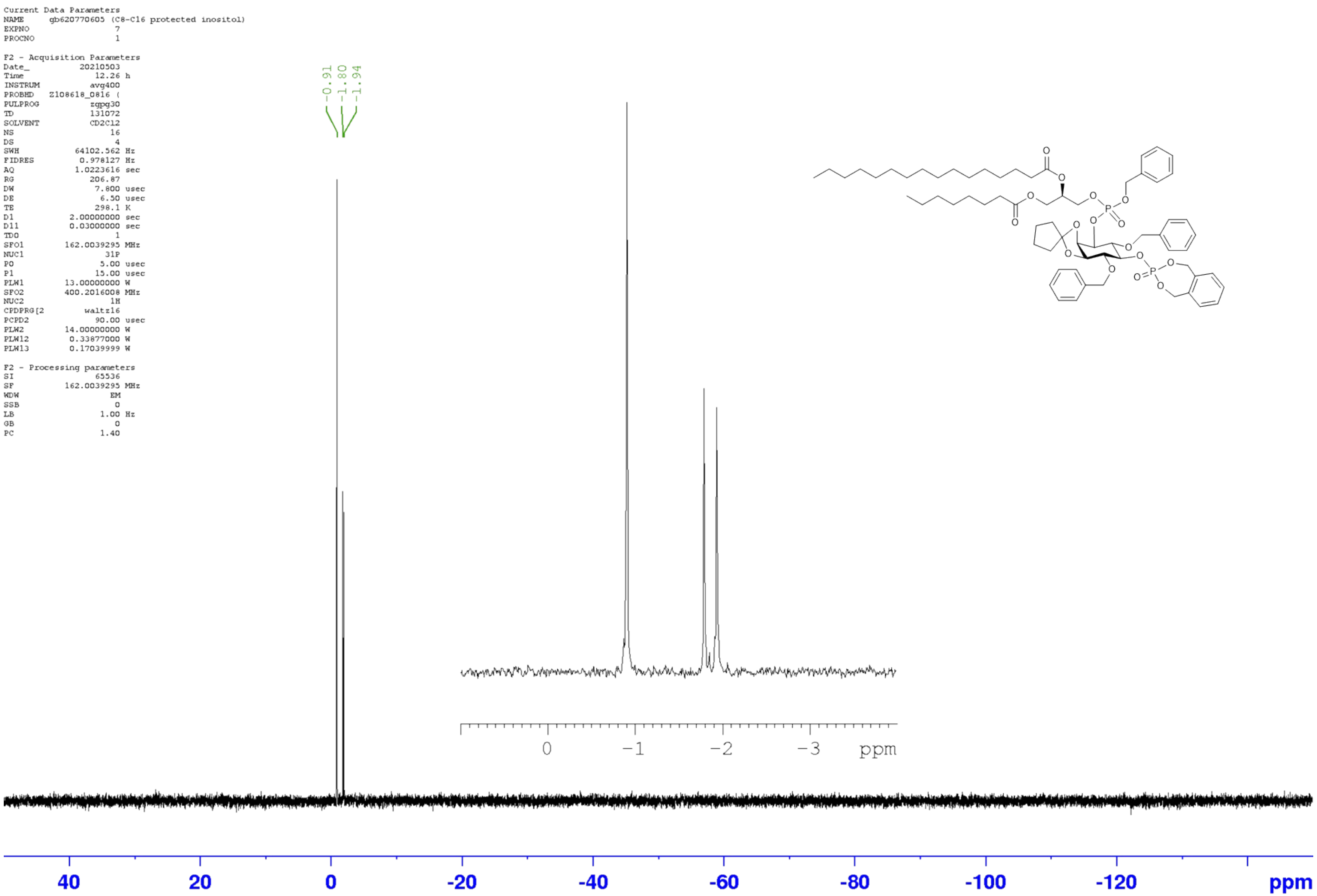

(+)-(6*R*)-3-({(3a*R*,4*S*,5*R*,6*S*,7*R*,7a*R*)-5,7-bis(benzyloxy)-6-[(3-oxo-1,5-dihydro-3*H*-2,4,3λ5-benzodioxaphosphepin-3-yl)oxy]hexahydrospiro[1,3-benzodioxole-2,1'-cyclopentan]-4-yl}oxy)-3,9-dioxo-1-phenyl-2,4,8-trioxa-3λ5-phosphahexadecan-6-yl hexadecanoate (+)-S19 ^{13}C NMR

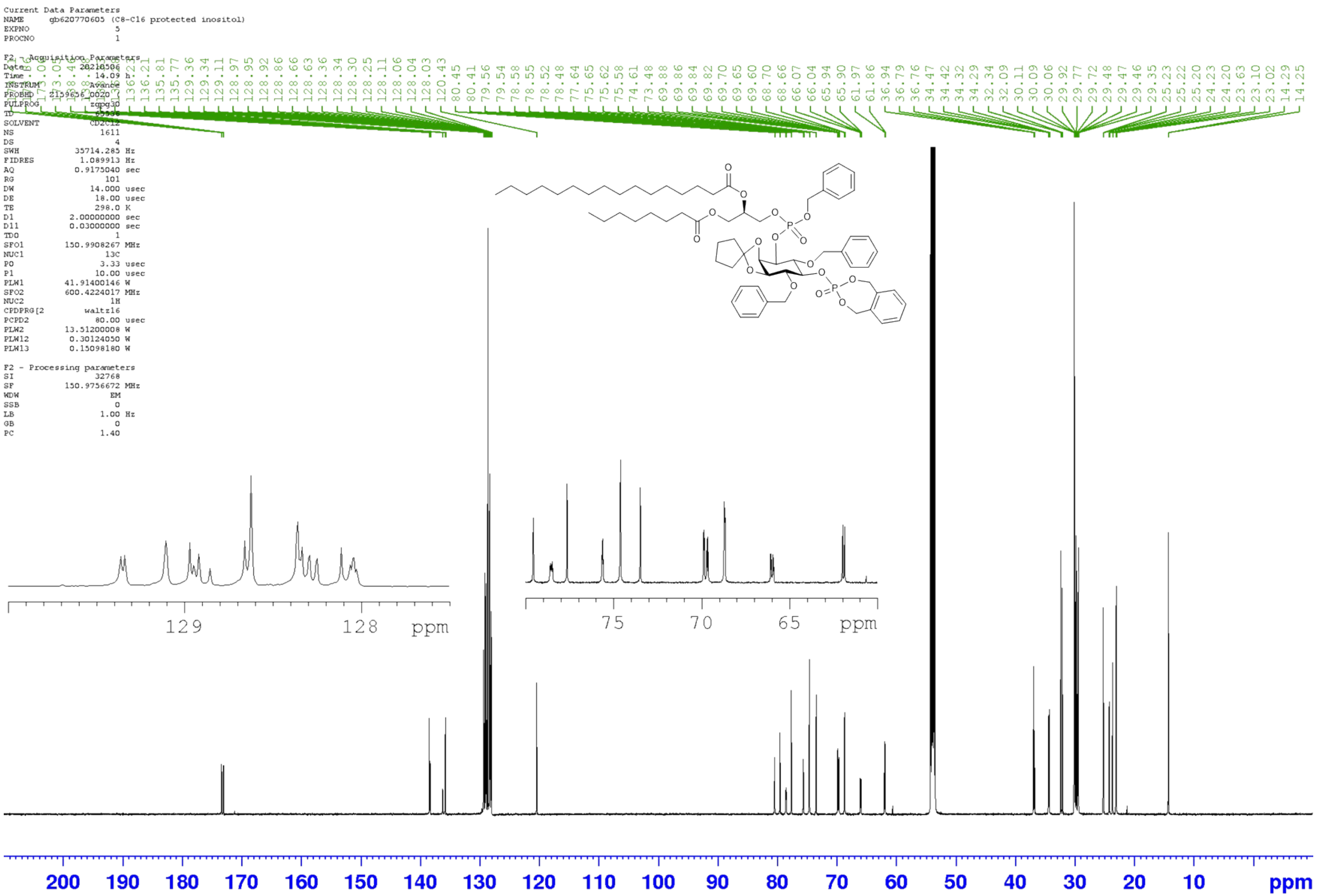

(+)-(6*R*)-3-({(3a*R*,4*S*,5*R*,6*S*,7*R*,7a*R*)-5,7-bis(benzyloxy)-6-[(3-oxo-1,5-dihydro-3*H*-2,4,3λ5-benzodioxaphosphepin-3-yl)oxy]hexahydrospiro[1,3-benzodioxole-2,1'-cyclopentan]-4-yl}oxy)-3,9-dioxo-1-phenyl-2,4,8-trioxa-3λ5-phosphahexadecan-6-yl hexadecanoate (+)-S19 HPLC 254 nm

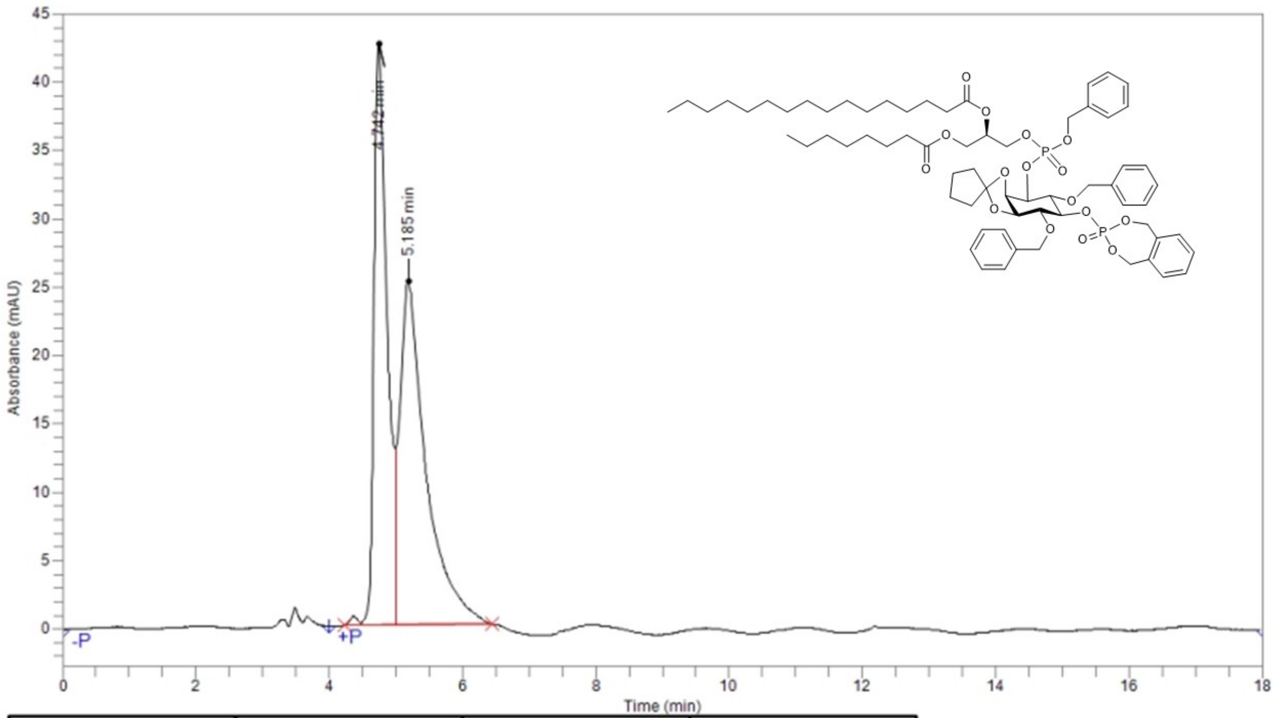

Time	Height	Area	Area %
4.362	691.6	5,588.2	0.44
4.742	42,578.5	568,610.9	44.87
5.185	25,163.5	693,050.8	54.69
**Total**		1,267,249.9	100.00

(+)-(6*R*)-3-({(3a*R*,4*S*,5*R*,6*S*,7*R*,7a*R*)-5,7-bis(benzyloxy)-6-[(3-oxo-1,5-dihydro-3*H*-2,4,3λ5-benzodioxaphosphepin-3-yl)oxy]hexahydrospiro[1,3-benzodioxole-2,1'-cyclopentan]-4-yl}oxy)-3,9-dioxo-1-phenyl-2,4,8-trioxa-3λ5-phosphahexadecan-6-yl hexadecanoate (+)-S19 HPLC 220 nm

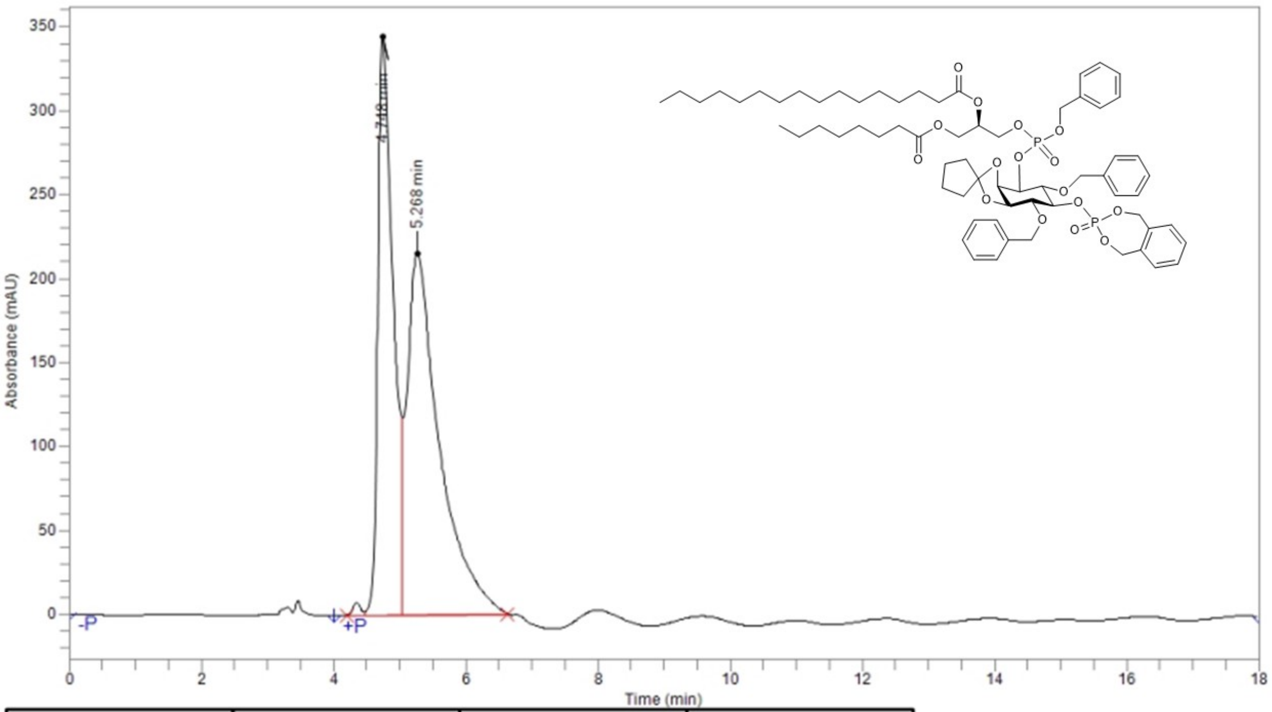

Time	Height	Area	Area %
4.347	7,433.2	57,182.2	0.44
4.748	344,882.0	5,605,897.8	43.22
5.268	215,370.0	7,308,067.4	56.34
**Total**		12,971,147.4	100.00

(+)-(6*R*)-3-({(3a*R*,4*S*,5*R*,6*S*,7*R*,7a*R*)-5,7-bis(benzyloxy)-6-[(3-oxo-1,5-dihydro-3*H*-2,4,3λ5-benzodioxaphosphepin-3-yl)oxy]hexahydrospiro[1,3-benzodioxole-2,1'-cyclopentan]-4-yl}oxy)-3,9-dioxo-1-phenyl-2,4,8-trioxa-3λ5-phosphahexadecan-6-yl hexadecanoate (+)-S19 HRMS

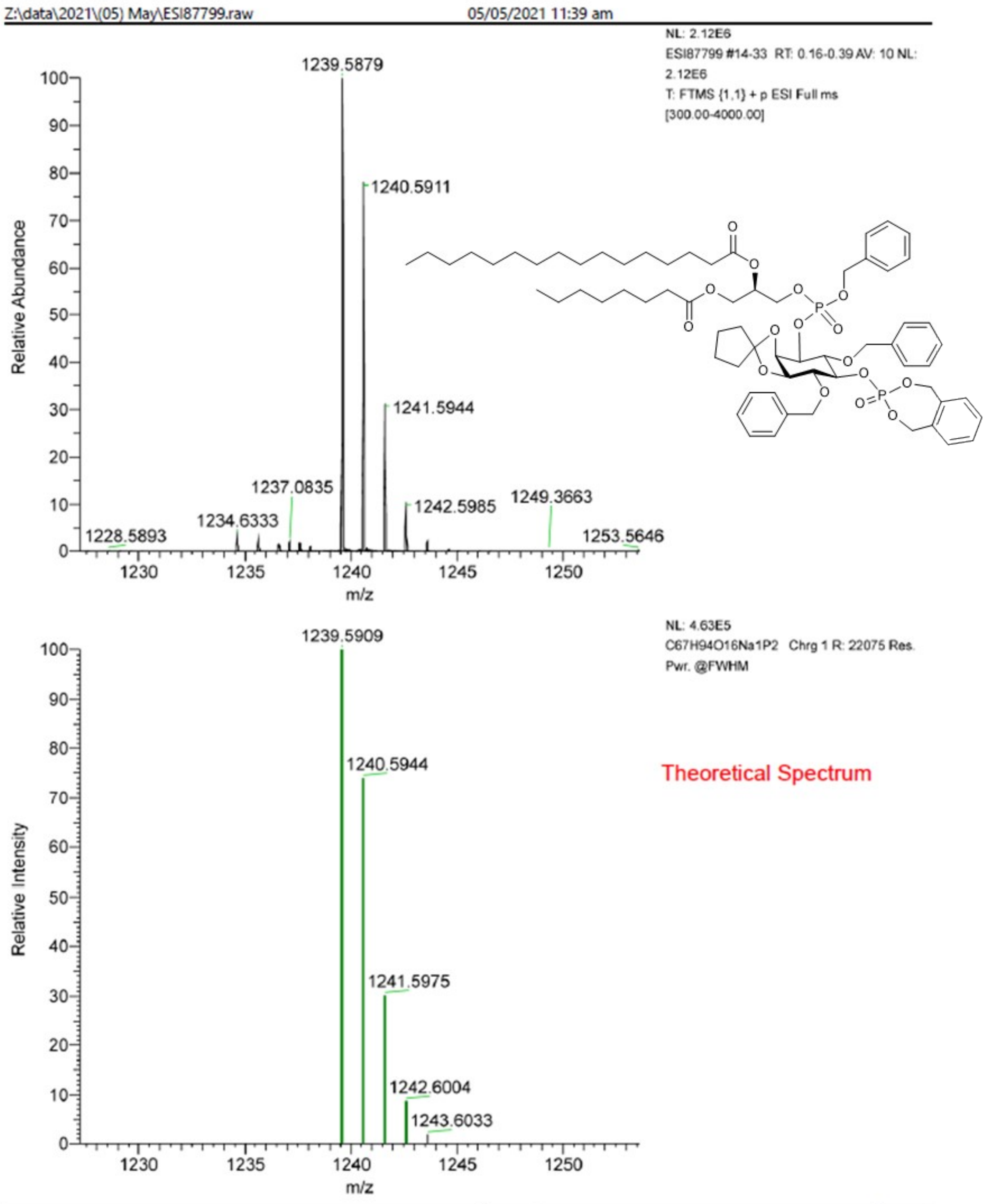

Peak Mass	Display...	Combin...	RDB	Delta (p...	Theo. m...	Rank	Combin...	# Match...	# Misse...	MS Cov....	Pattern...	MSMS...
1239.5	[illegible]	67.931	21.50	-2.46	1239.5	1	94.892	6	0	96.390	100	(Collec

(+)-(2*R*)-3-[(hydroxy{[(1R,2R,3R,4R,5S,6R)-2,3,4,6-tetrahydroxy-5-(phosphonooxy)cyclohexyl]oxy}phosphoryl)oxy]-2-(octanoyloxy)propyl hexadecanoate (+)-7 ^{1}H NMR

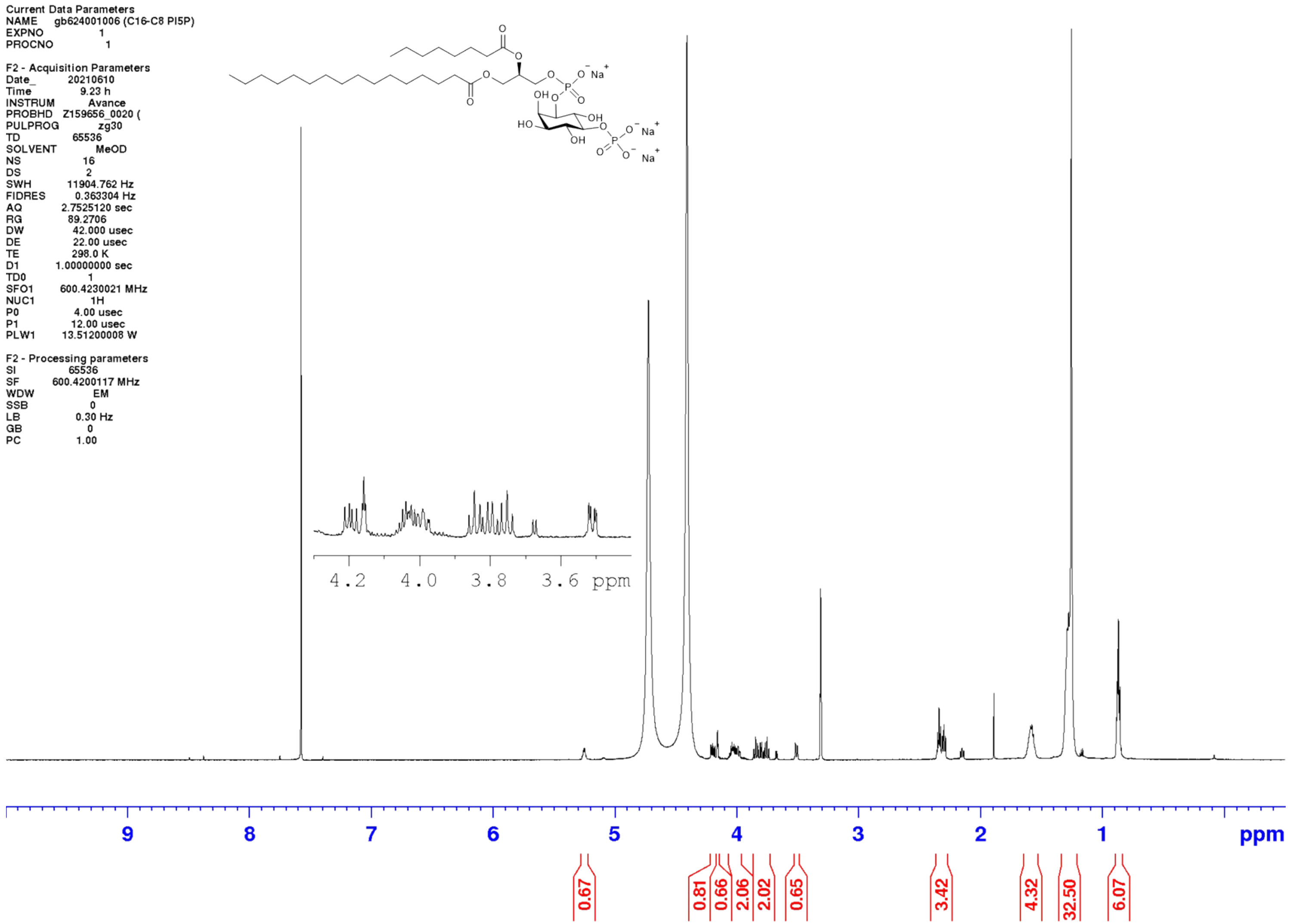

(+)-(2*R*)-3-[(hydroxy{[(1R,2R,3R,4R,5S,6R)-2,3,4,6-tetrahydroxy-5-(phosphonooxy)cyclohexyl]oxy}phosphoryl)oxy]-2-(octanoyloxy)propyl hexadecanoate (+)-7 ^{31}P NMR

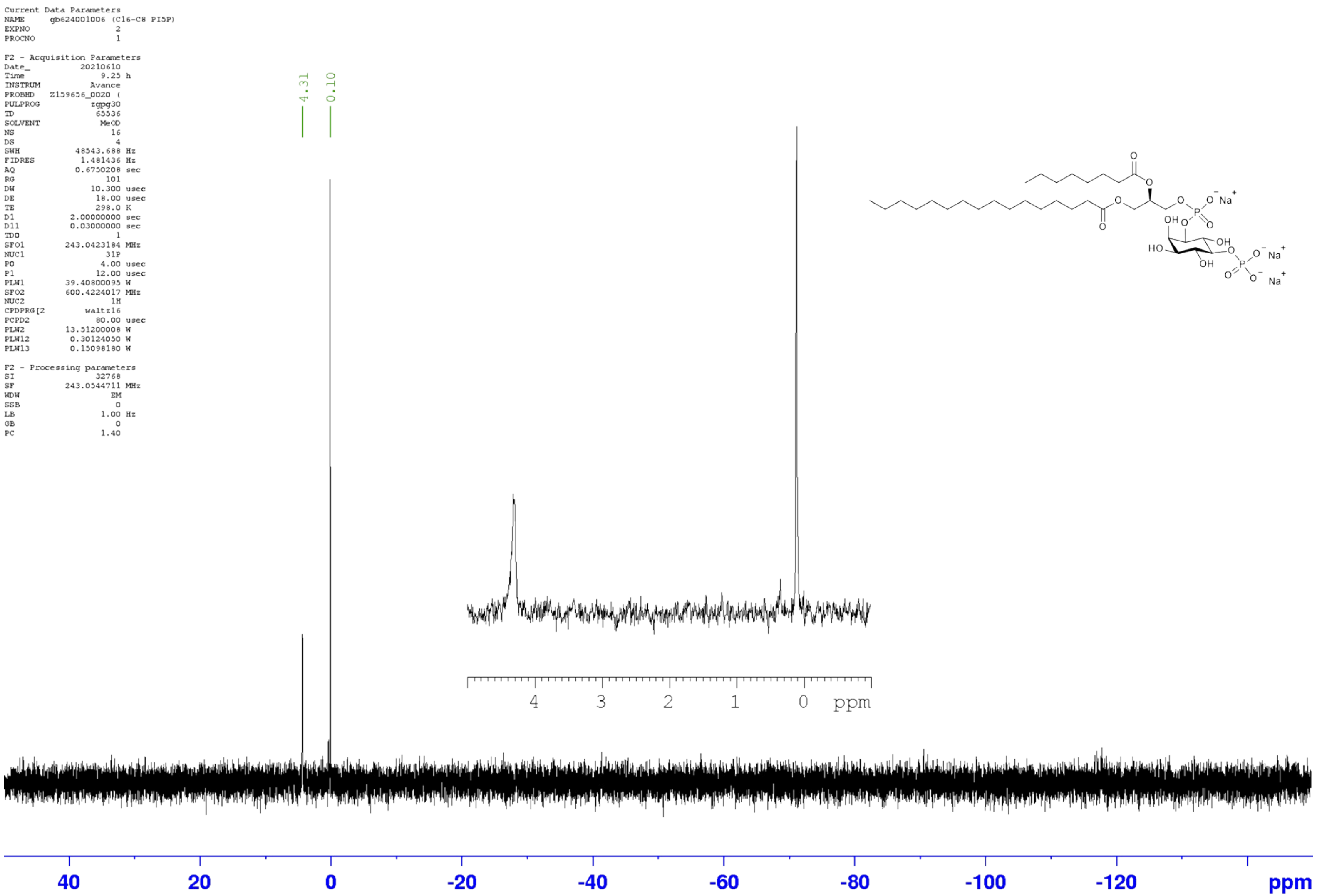

(+)-(2*R*)-3-[(hydroxy{[(1R,2R,3R,4R,5S,6R)-2,3,4,6-tetrahydroxy-5-(phosphonooxy)cyclohexyl]oxy}phosphoryl)oxy]-2-(octanoyloxy)propyl hexadecanoate (+)-7 ^{13}C NMR

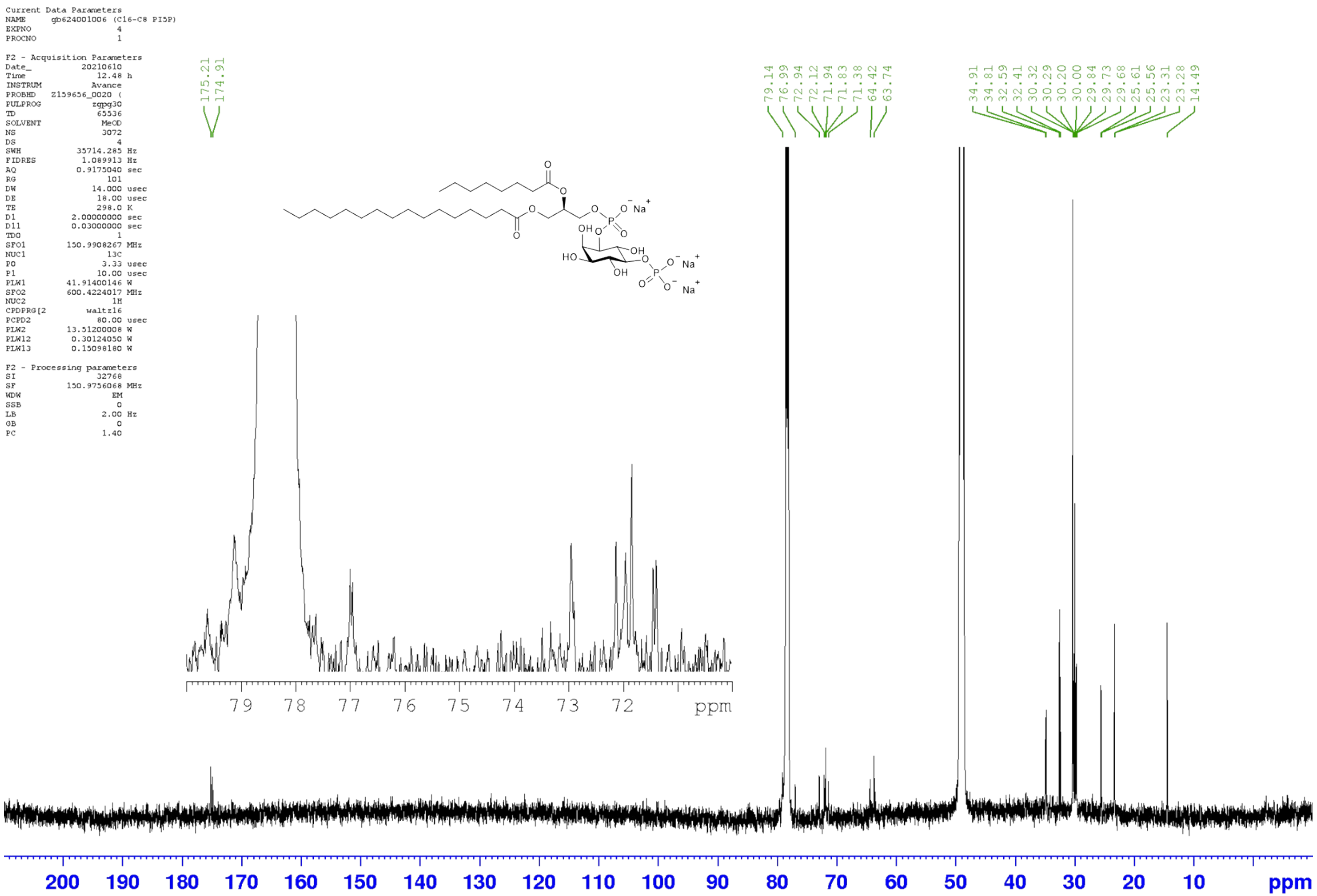

(+)-(2*R*)-3-[(hydroxy{[(1R,2R,3R,4R,5S,6R)-2,3,4,6-tetrahydroxy-5-(phosphonooxy)cyclohexyl]oxy}phosphoryl)oxy]-2-(octanoyloxy)propyl hexadecanoate (+)-7 ^{1}H to ^{13}C HSQC NMR

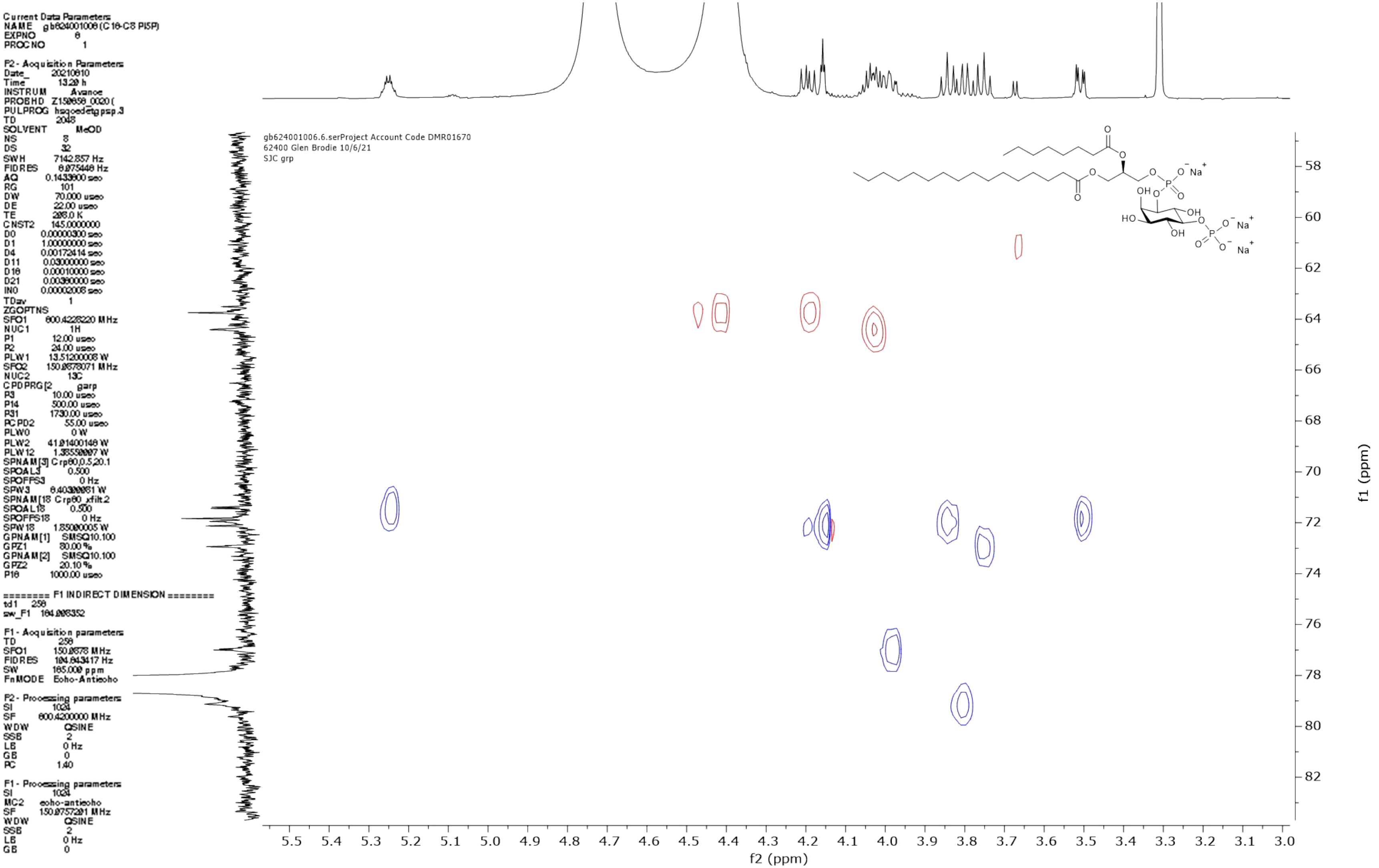

(+)-(2*R*)-3-[(hydroxy{[(1R,2R,3R,4R,5S,6R)-2,3,4,6-tetrahydroxy-5-(phosphonooxy)cyclohexyl]oxy}phosphoryl)oxy]-2-(octanoyloxy)propyl hexadecanoate (+)-7
HRMS

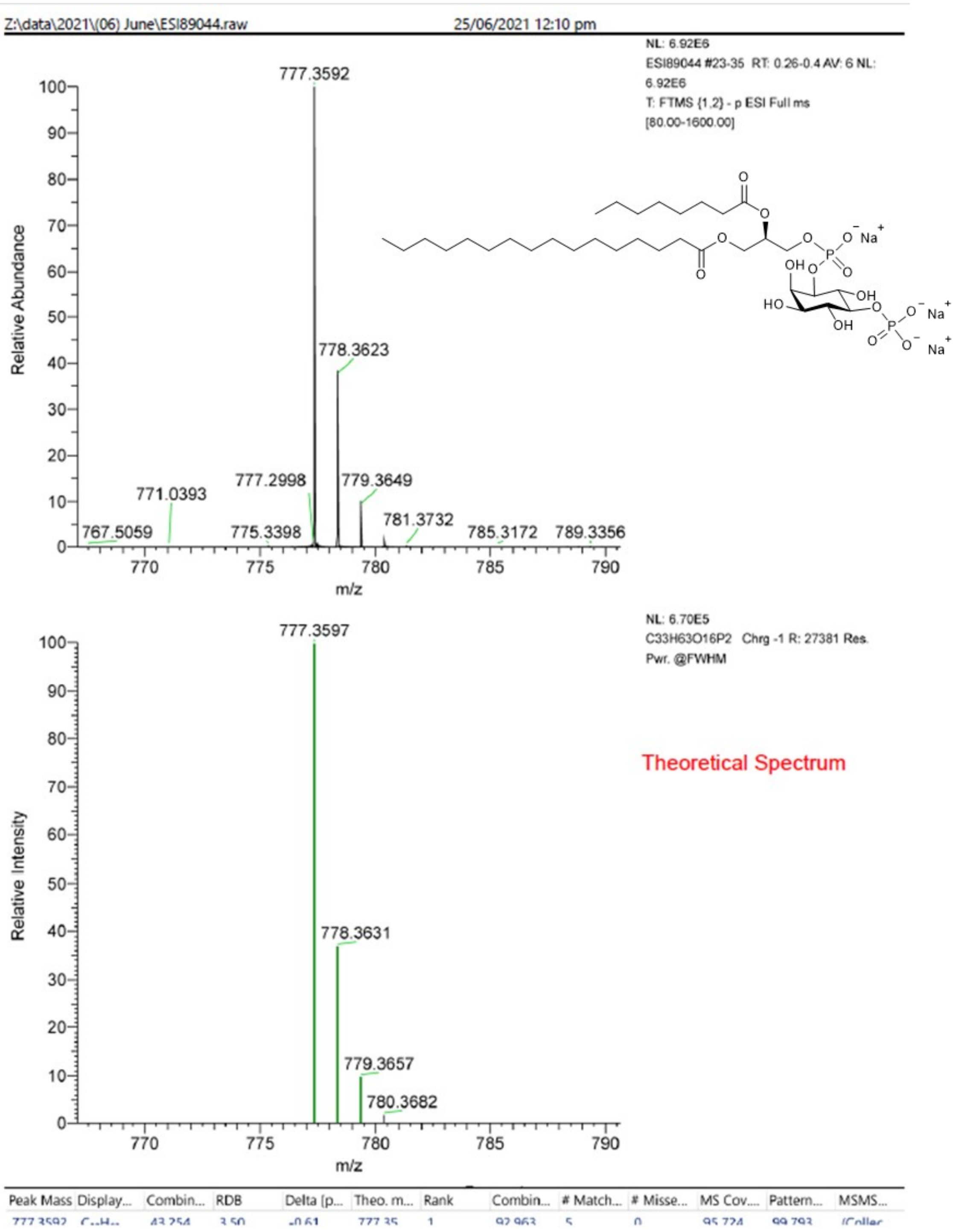

(2*R*)-1-[(hydroxy{[(1*R*,2*R*,3*R*,4*R*,5*S*,6*R*)-2,3,4,6-tetrahydroxy-5-(phosphonooxy)cyclohexyl]oxy}phosphoryl)oxy]-3-(octanoyloxy)propan-2-yl hexadecanoate (+)-8 ^{1}H NMR

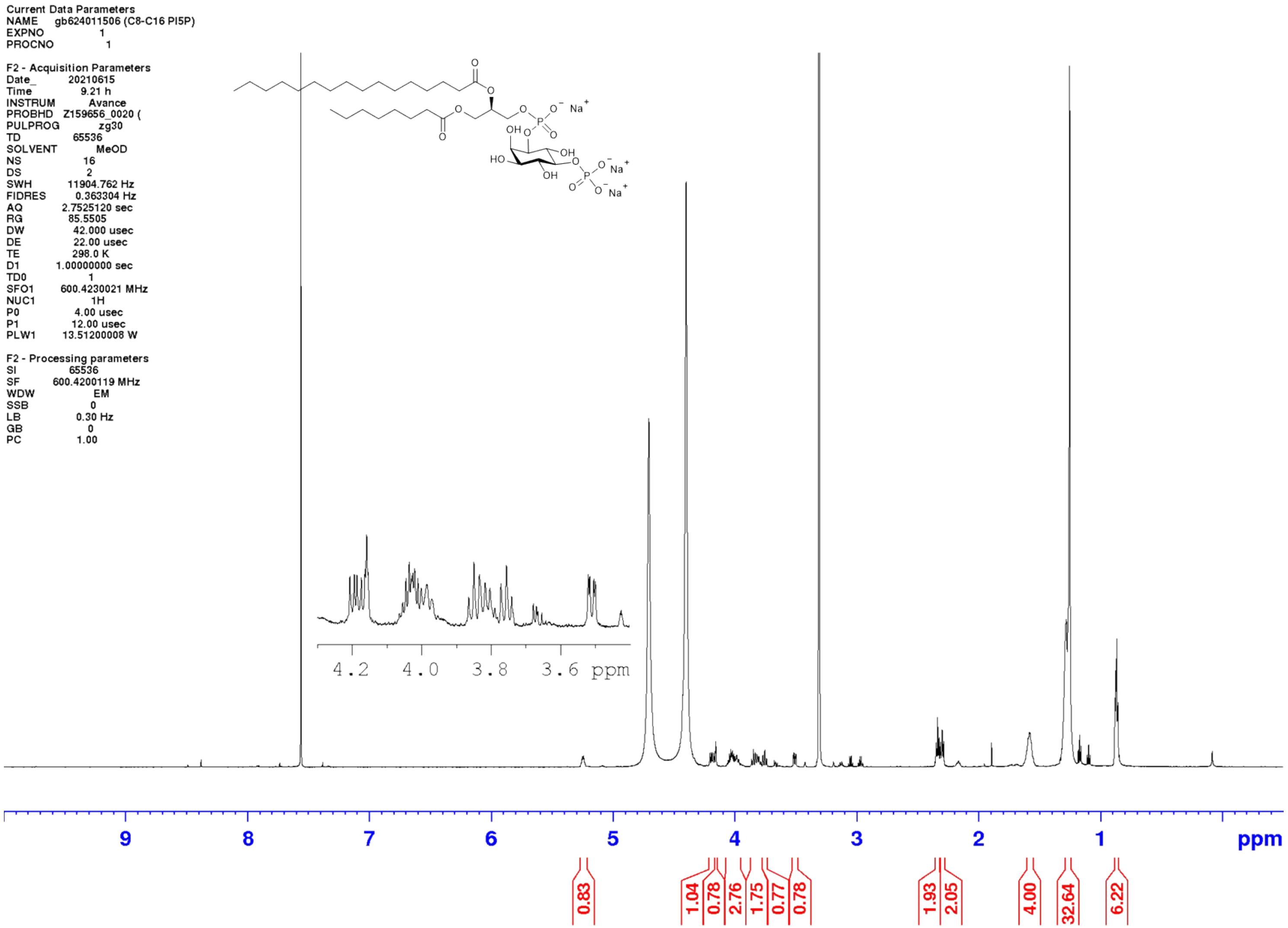

(2*R*)-1-[(hydroxy{[(1*R*,2*R*,3*R*,4*R*,5*S*,6*R*)-2,3,4,6-tetrahydroxy-5-(phosphonooxy)cyclohexyl]oxy}phosphoryl)oxy]-3-(octanoyloxy)propan-2-yl hexadecanoate (+)-8 ^{31}P NMR

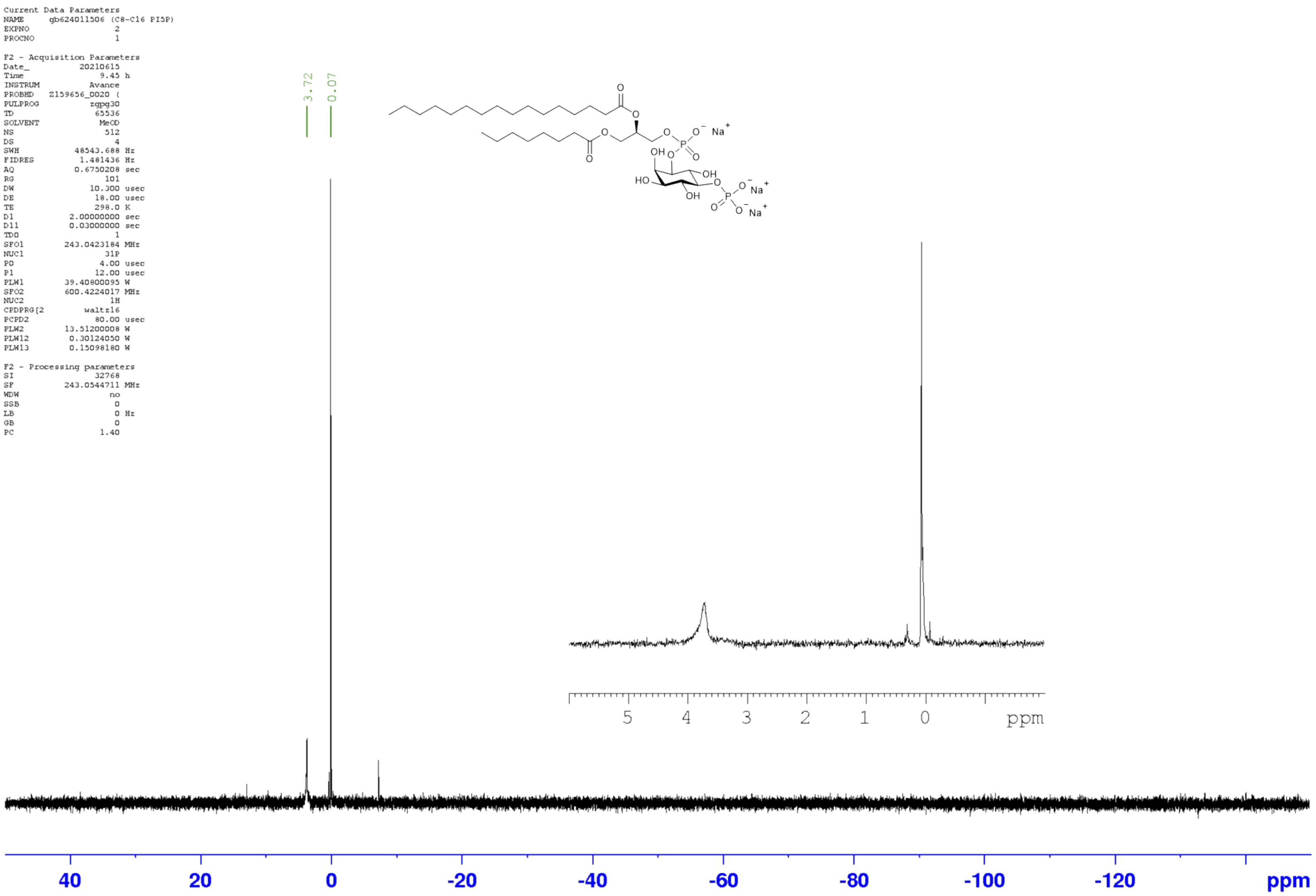

(2*R*)-1-[(hydroxy{[(1*R*,2*R*,3*R*,4*R*,5*S*,6*R*)-2,3,4,6-tetrahydroxy-5-(phosphonooxy)cyclohexyl]oxy}phosphoryl)oxy]-3-(octanoyloxy)propan-2-yl hexadecanoate (+)-8 ^{13}C NMR

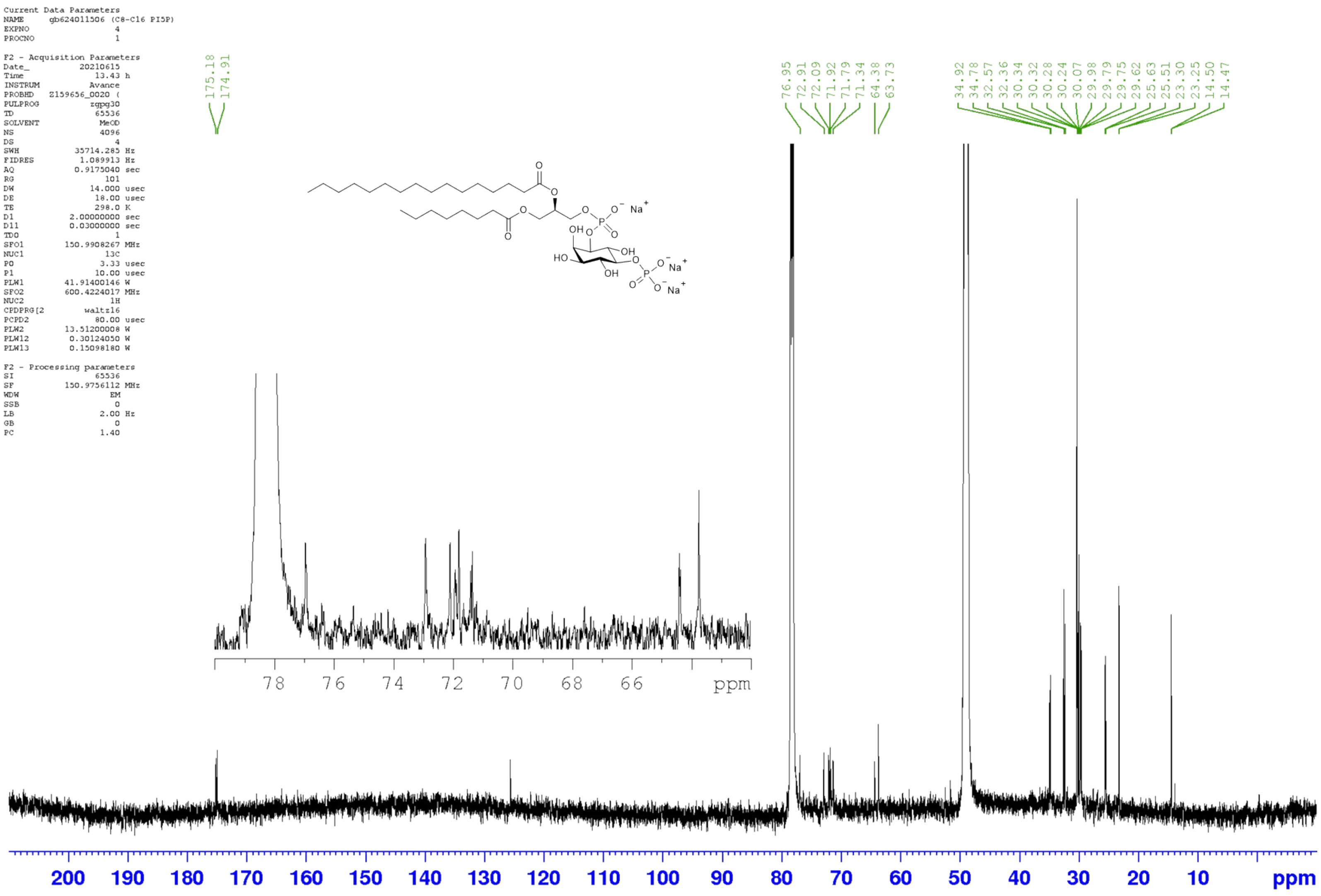

(2*R*)-1-[(hydroxy{[(1*R*,2*R*,3*R*,4*R*,5*S*,6*R*)-2,3,4,6-tetrahydroxy-5-(phosphonooxy)cyclohexyl]oxy}phosphoryl)oxy]-3-(octanoyloxy)propan-2-yl hexadecanoate (+)-8 ^{1}H to ^{13}C HSQC NMR

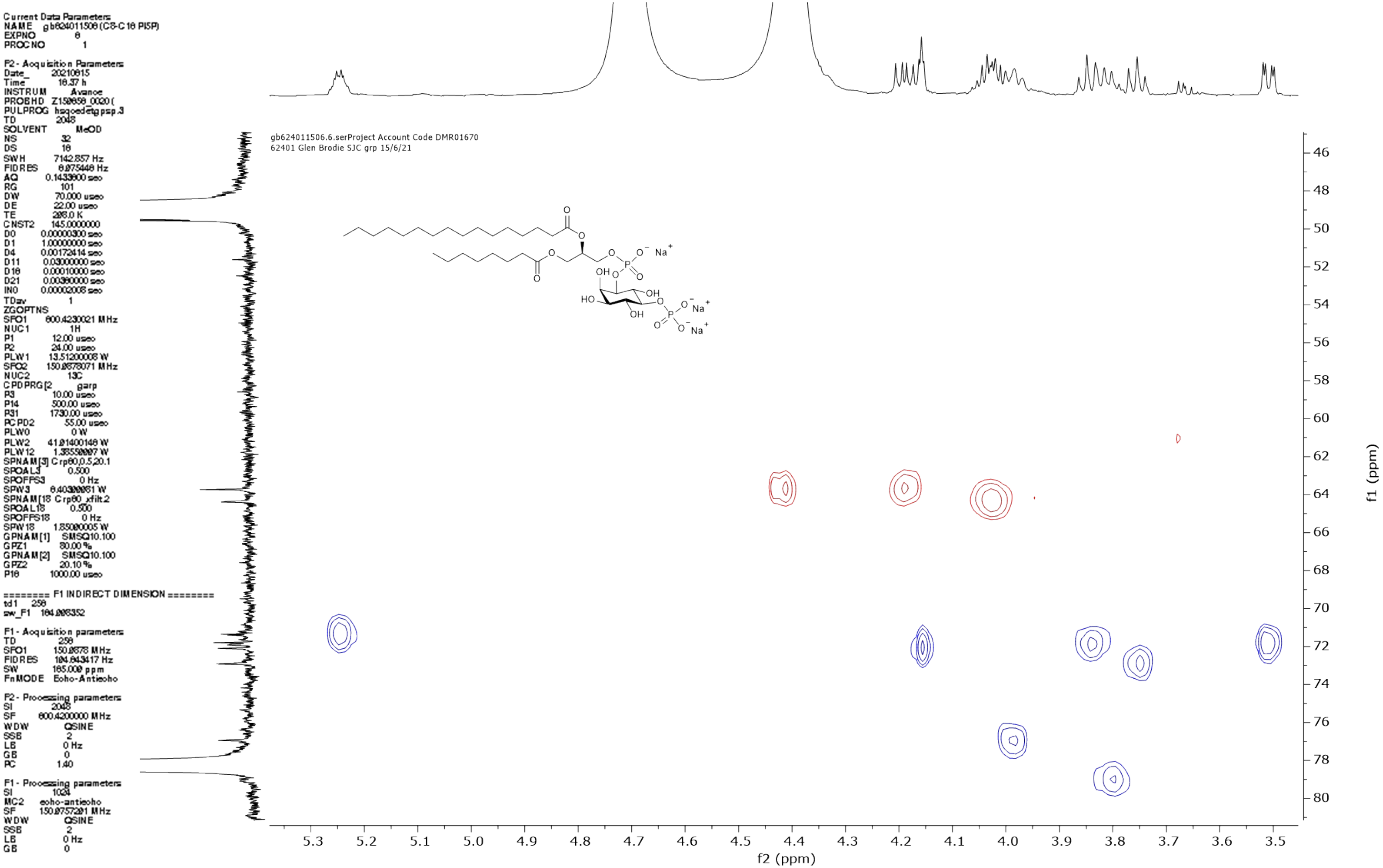

(2*R*)-1-[(hydroxy{[(1*R*,2*R*,3*R*,4*R*,5*S*,6*R*)-2,3,4,6-tetrahydroxy-5-(phosphonooxy)cyclohexyl]oxy}phosphoryl)oxy]-3-(octanoyloxy)propan-2-yl hexadecanoate (+)-8 HRMS

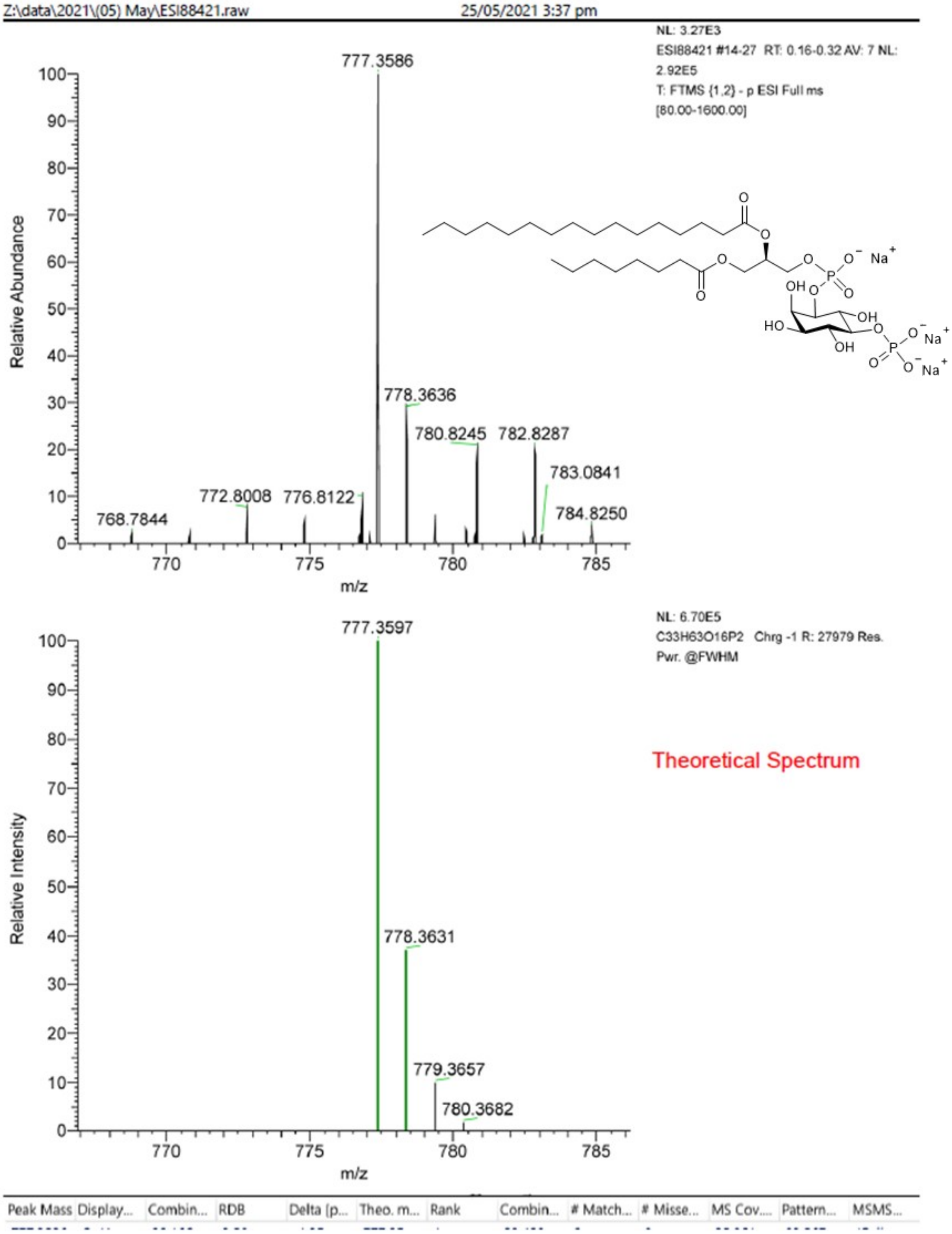

(+)-(2*R*)-3-{[(2*S*)-2-methoxy-2-phenylacetyl]oxy}-2-(octanoyloxy)propyl hexadecanoate (+)-S20 ^{1}H NMR

Current Data Parameters
NAME gb634271208 (C16, C-8 S MPA)
EXPNO 1
PROCNO 1

F2 - Acquisition Parameters
Date_ 20210813
Time 16.56 h
INSTRUM Avance
PROBHD Z159656_0020 (
PULPROG zg30
TD 65536
SOLVENT CDCl3
NS 16
DS 2
SWH 11904.762 Hz
FIDRES 0.363304 Hz
AQ 2.7525120 sec
RG 52.7314
DW 42.000 usec
DE 22.00 usec
TE 298.0 K
D1 1.00000000 sec
TD0 1
SFO1 600.4230021 MHz
NUC1 1H
P0 4.00 usec
P1 12.00 usec
PLW1 13.51200008 W

F2 - Processing parameters
SI 65536
SF 600.4200136 MHz
WDW EM
SSB 0
LB 0.30 Hz
GB 0
PC 1.00

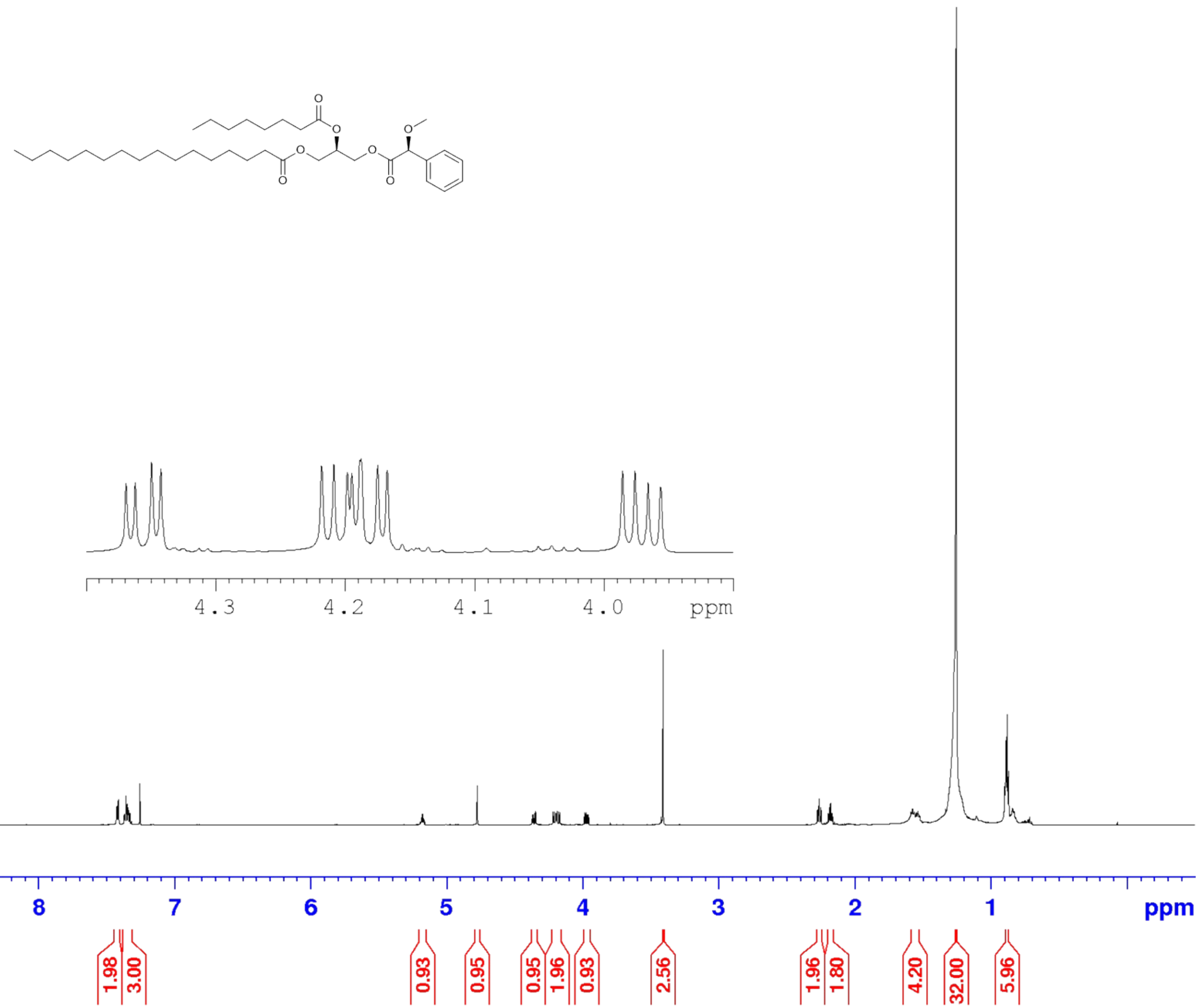

(+)-(2*R*)-3-{[(2*S*)-2-methoxy-2-phenylacetyl]oxy}-2-(octanoyloxy)propyl hexadecanoate (+)-S20 ^{13}C NMR

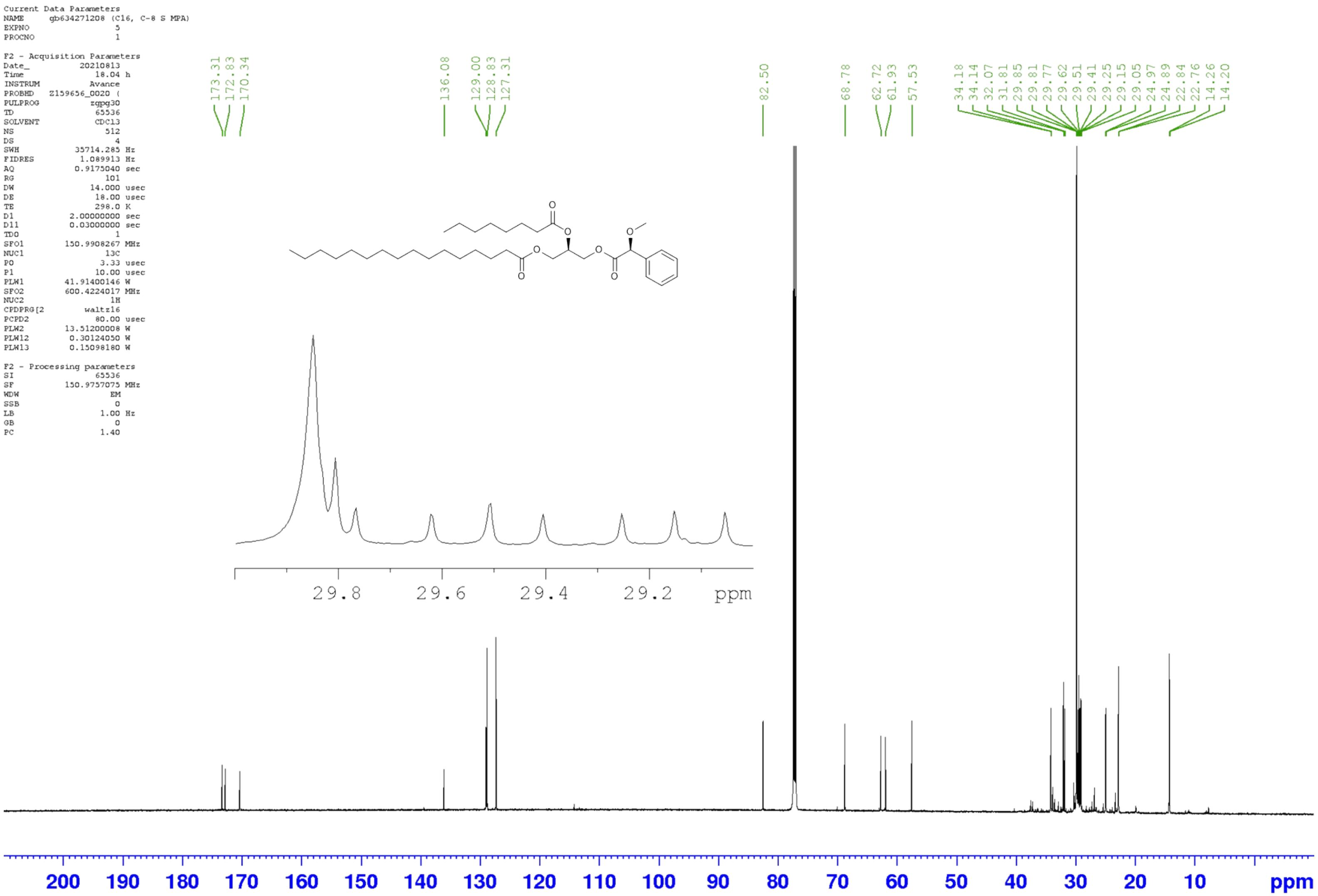

(+)-(2*R*)-3-{[(2*S*)-2-methoxy-2-phenylacetyl]oxy}-2-(octanoyloxy)propyl hexadecanoate (+)-S20
HRMS

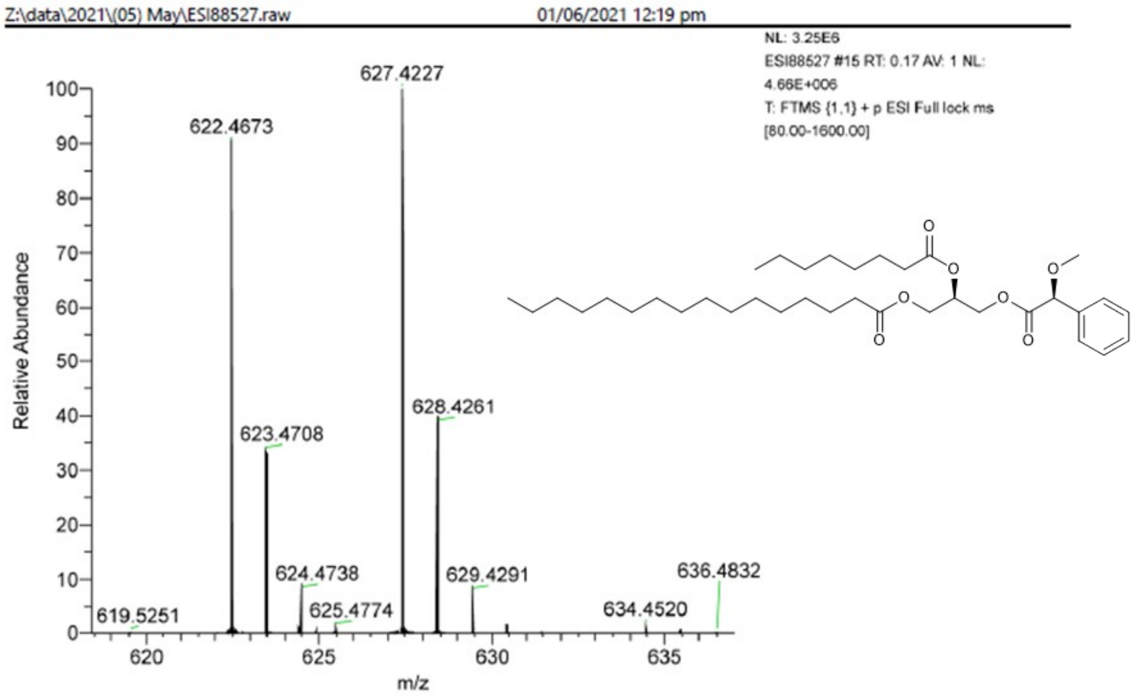

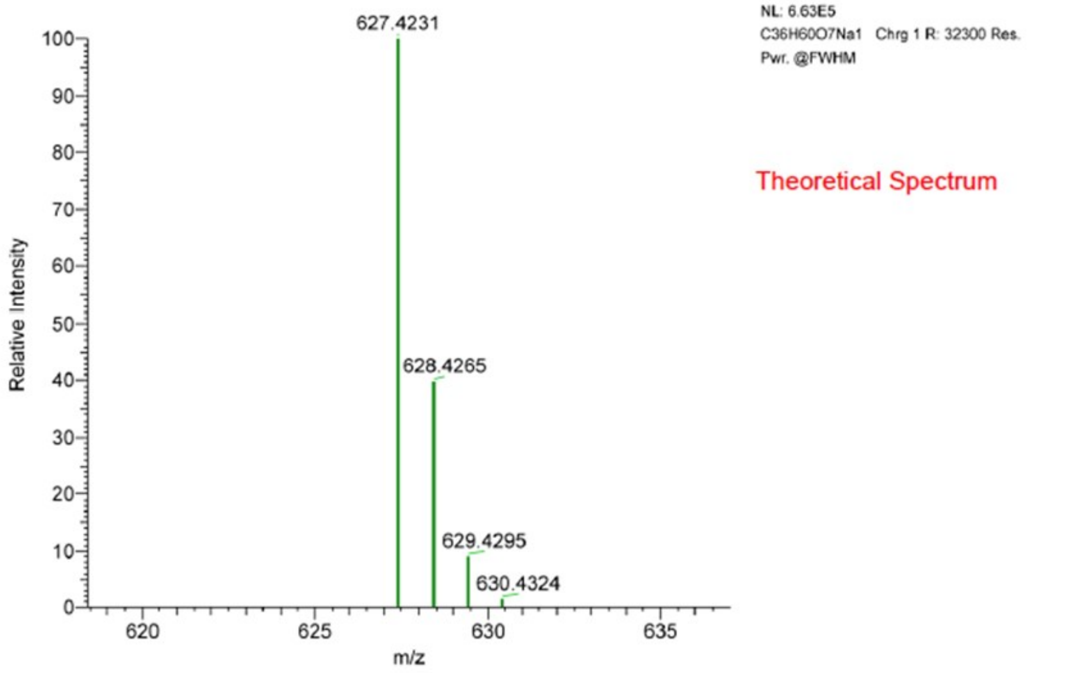

Peak Mass	Display...	Combin...	RDB	Delta [p...	Theo. m...	Rank	Combin...	# Match...	# Misse...	MS Cov....	Pattern...	MSMS...
627.4227	[illegible]	73.449	6.50	-0.63	627.42	1	98.551	5	0	99.945	100	(Collec

(–)-(2*R*)-3-{[(2*R*)-2-methoxy-2-phenylacetyl]oxy}-2-(octanoyloxy)propyl hexadecanoate (–)-S21 ^{1}H NMR

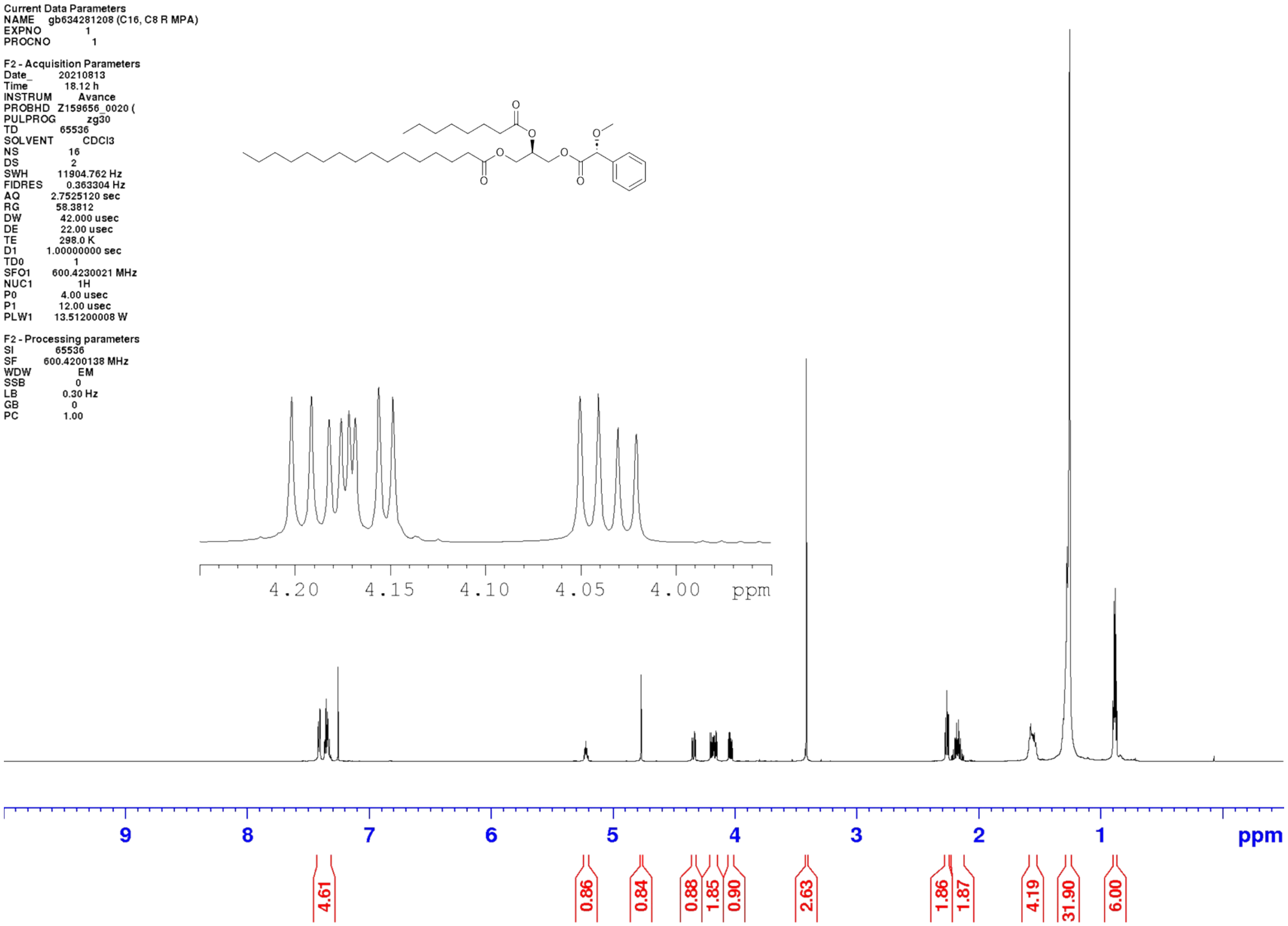

(–)-(2*R*)-3-{[(2*R*)-2-methoxy-2-phenylacetyl]oxy}-2-(octanoyloxy)propyl hexadecanoate (–)-S21 ^{13}C NMR

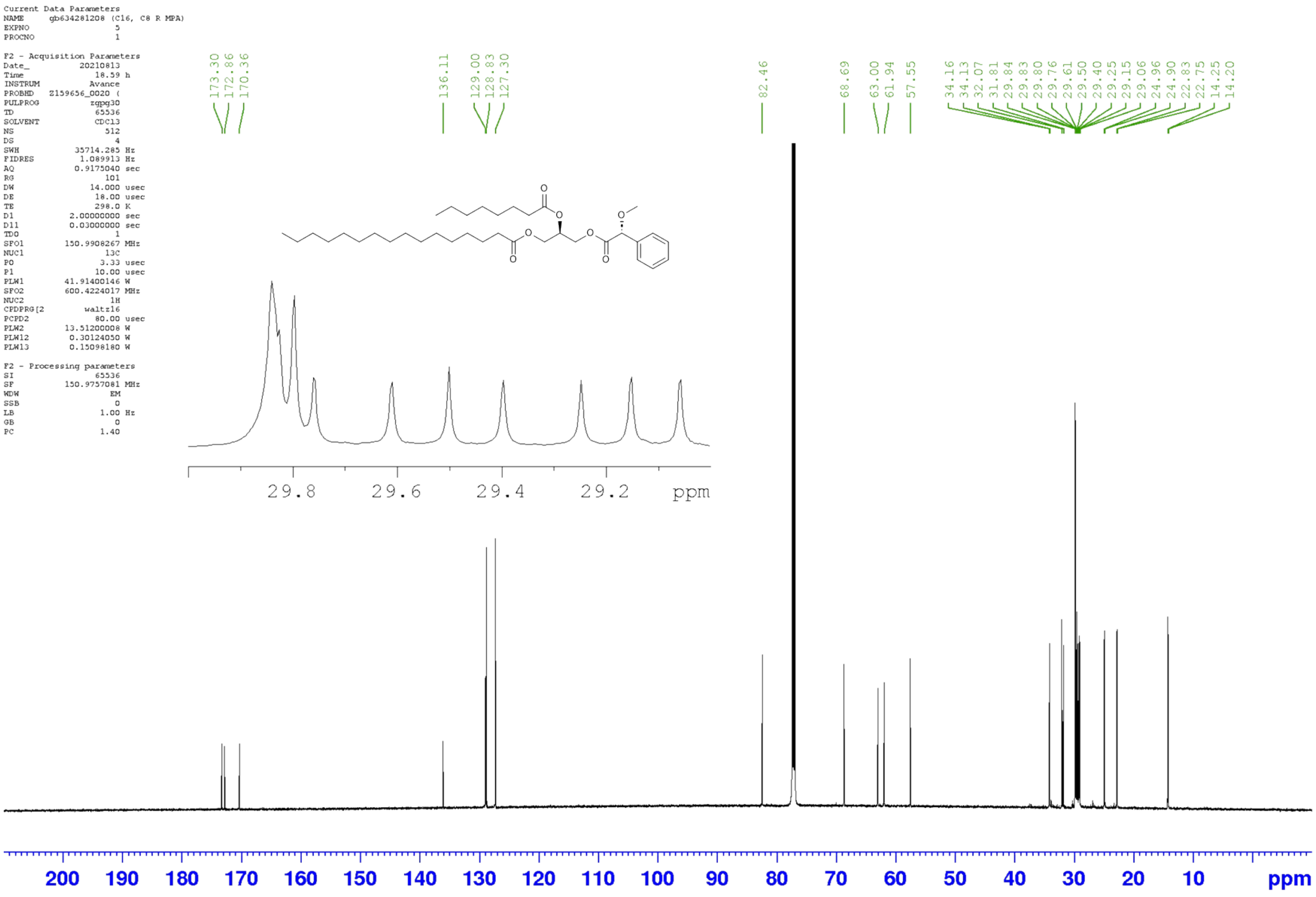

(–)-(2*R*)-3-{[(2*R*)-2-methoxy-2-phenylacetyl]oxy}-2-(octanoyloxy)propyl hexadecanoate (–)-S21
HRMS

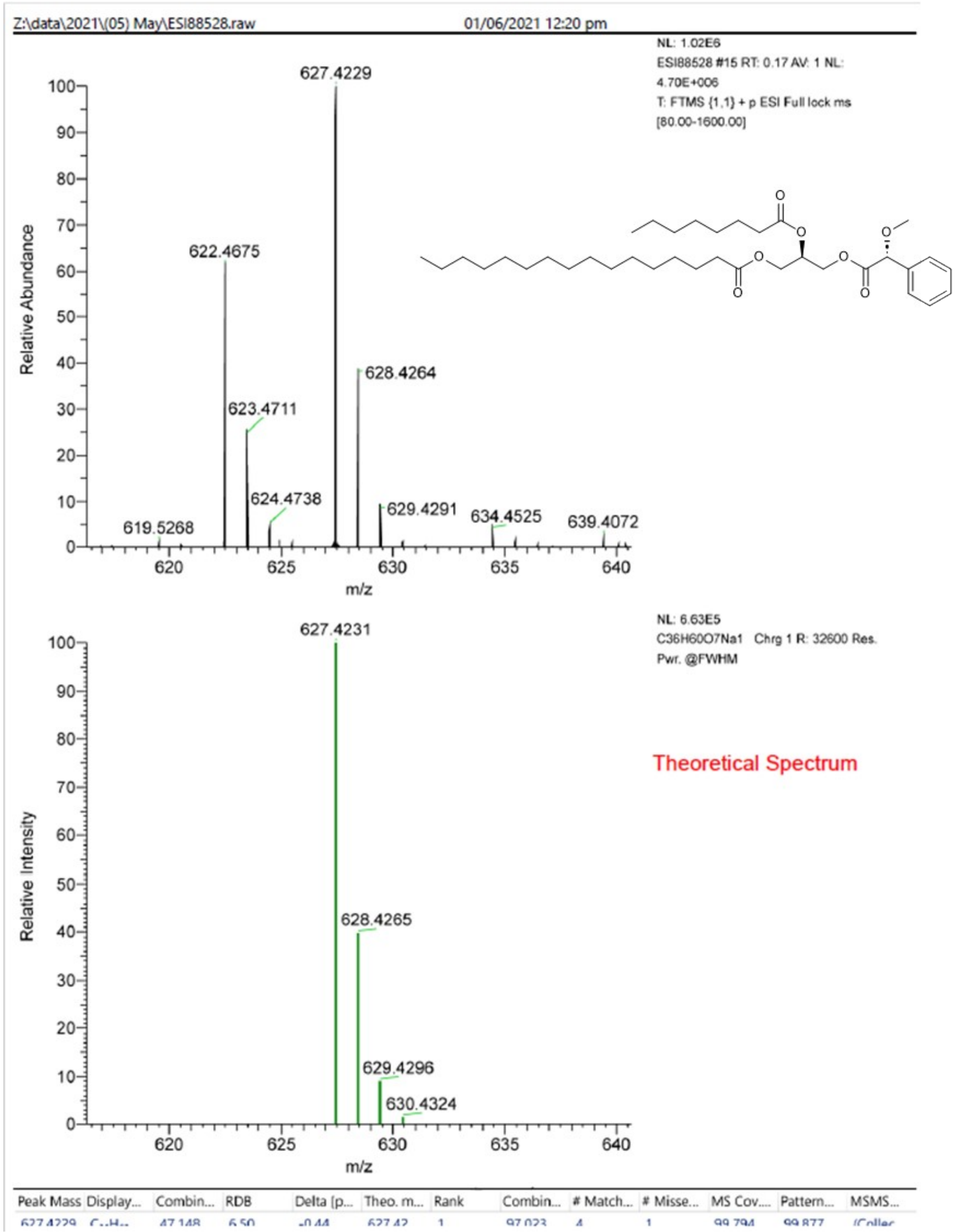

(+)-(2*R*)-1-{[(2*S*)-2-methoxy-2-phenylacetyl]oxy}-3-(octanoyloxy)propan-2-yl hexadecanoate (+)-S22 ^{1}H NMR

Current Data Parameters
NAME gb634291208 (C8, C16 S MPA)
EXPNO 1
PROCNO 1

F2 - Acquisition Parameters
Date_ 20210813
Time 19.07 h
INSTRUM Avance
PROBHD Z159656_0020 (
PULPROG zg30
TD 65536
SOLVENT CDCl3
NS 16
DS 2
SWH 11904.762 Hz
FIDRES 0.363304 Hz
AQ 2.7525120 sec
RG 74.5949
DW 42.000 usec
DE 22.00 usec
TE 298.0 K
D1 1.00000000 sec
TD0 1
SFO1 600.4230021 MHz
NUC1 1H
P0 4.00 usec
P1 12.00 usec
PLW1 13.51200008 W

F2 - Processing parameters
SI 65536
SF 600.4200145 MHz
WDW EM
SSB 0
LB 0.30 Hz
GB 0
PC 1.00

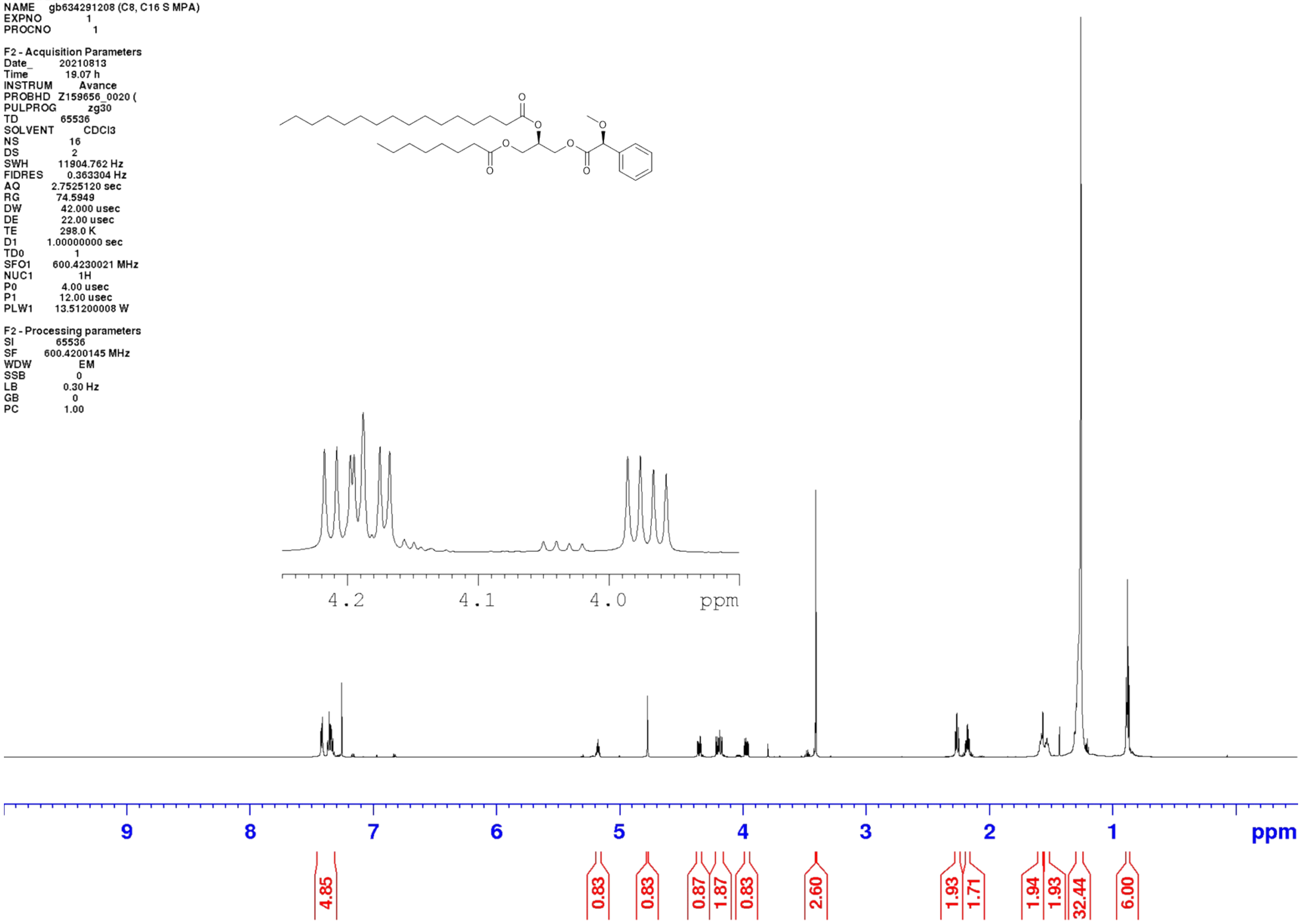

(+)-(2*R*)-1-{[(2*S*)-2-methoxy-2-phenylacetyl]oxy}-3-(octanoyloxy)propan-2-yl hexadecanoate (+)-S22 ^{13}C NMR

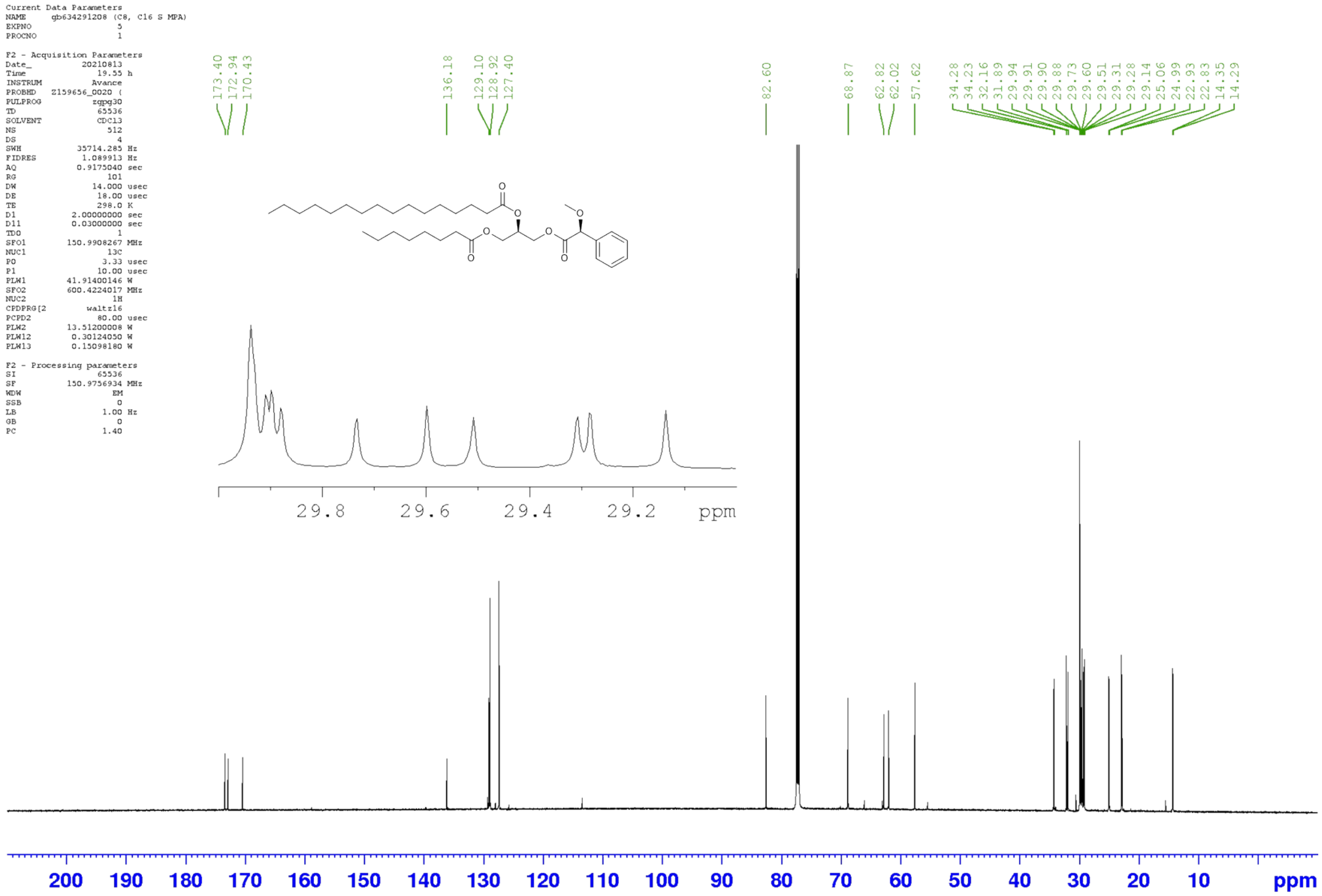

(+)-(2*R*)-1-{[(2*S*)-2-methoxy-2-phenylacetyl]oxy}-3-(octanoyloxy)propan-2-yl hexadecanoate (+)-S22 HRMS

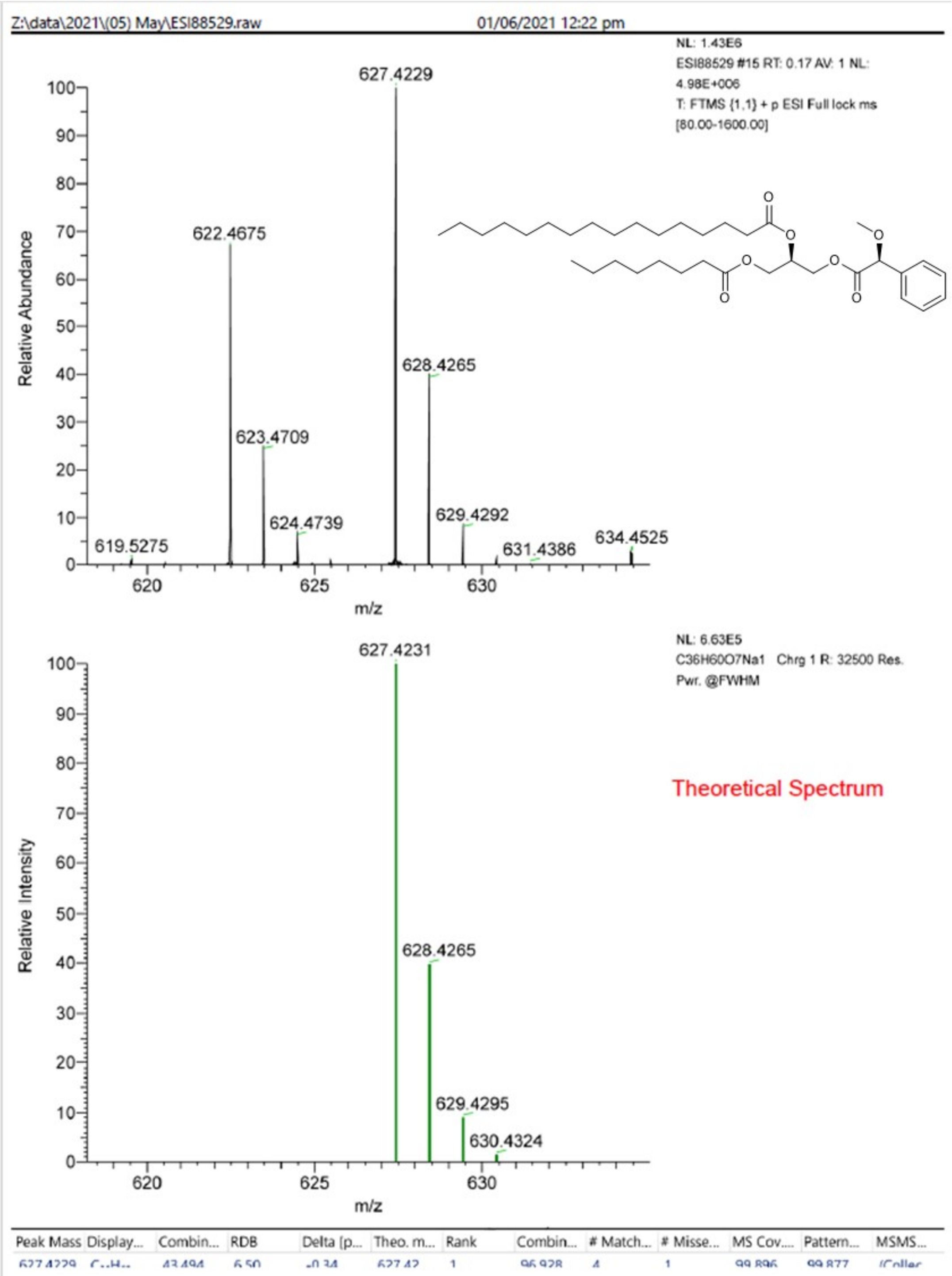

(–)-(2*R*)-1-{[(2*R*)-2-methoxy-2-phenylacetyl]oxy}-3-(octanoyloxy)propan-2-yl hexadecanoate (–)-S23 ¹H NMR

Current Data Parameters
NAME gb634301208 (C8, C16 R MPA)
EXPNO 6
PROCNO 1

F2 - Acquisition Parameters
Date_ 20210817
Time 13.10 h
INSTRUM Avance
PROBHD Z159656_0020 (
PULPROG zg30
TD 65536
SOLVENT CDCl3
NS 16
DS 2
SWH 11904.762 Hz
FIDRES 0.363304 Hz
AQ 2.7525120 sec
RG 77.9052
DW 42.000 usec
DE 22.00 usec
TE 298.0 K
D1 1.00000000 sec
TD0 1
SFO1 600.4230021 MHz
NUC1 1H
P0 4.00 usec
P1 12.00 usec
PLW1 13.51200008 W

F2 - Processing parameters
SI 65536
SF 600.4200145 MHz
WDW EM
SSB 0
LB 0.30 Hz
GB 0
PC 1.00

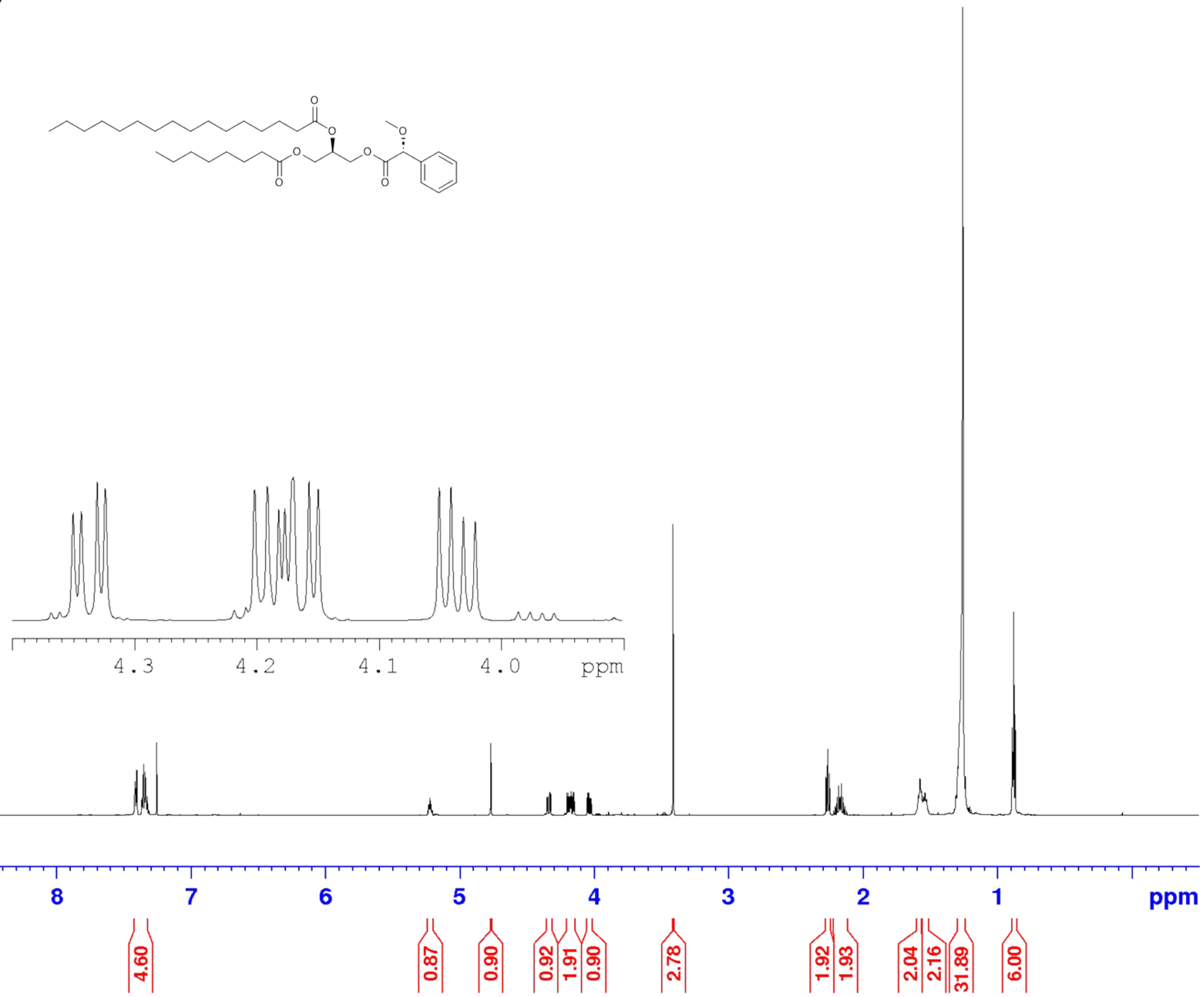

(–)-(2*R*)-1-{[(2*R*)-2-methoxy-2-phenylacetyl]oxy}-3-(octanoyloxy)propan-2-yl hexadecanoate (–)-S23 ^{13}C NMR

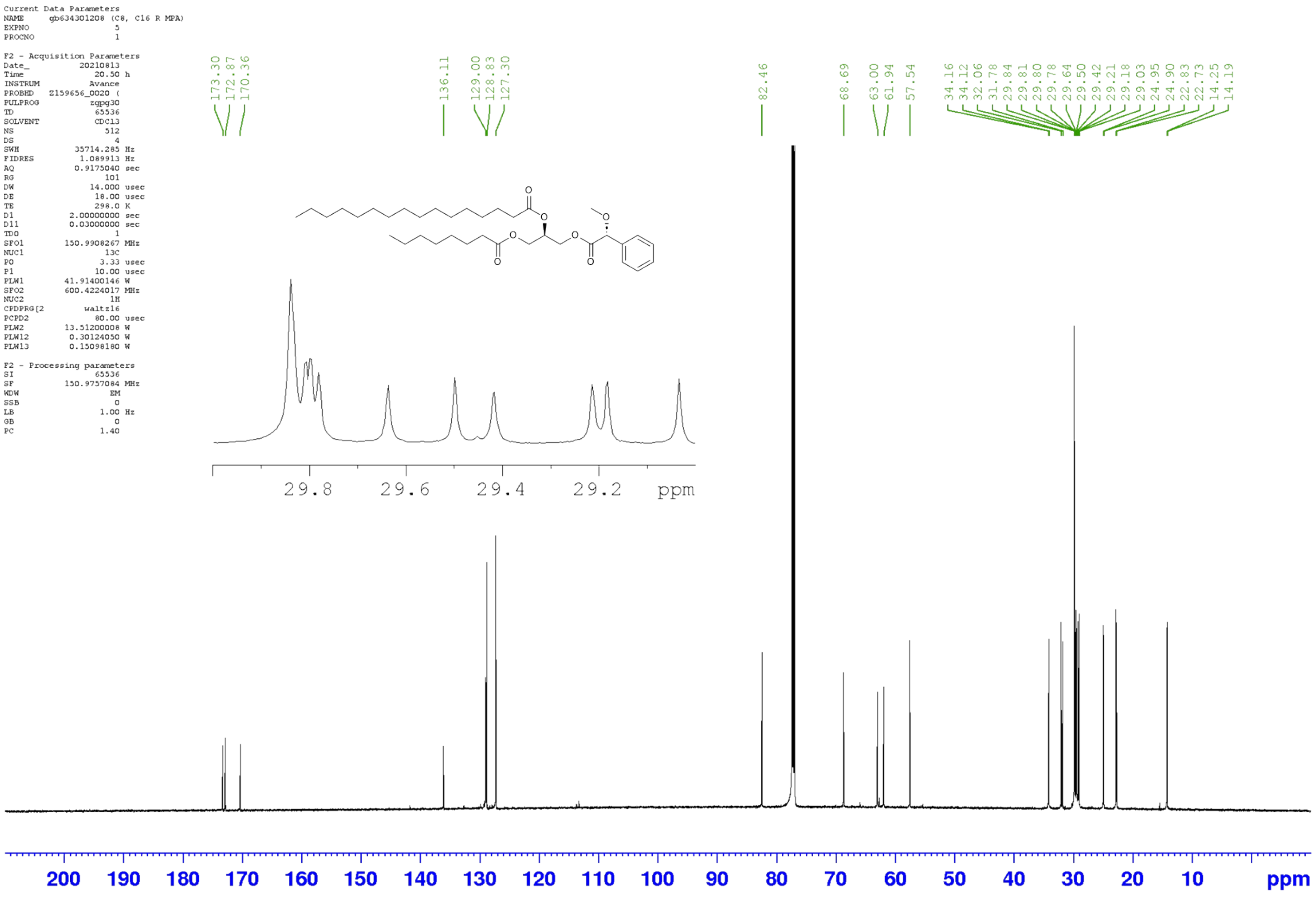

(–)-(2*R*)-1-{[(2*R*)-2-methoxy-2-phenylacetyl]oxy}-3-(octanoyloxy)propan-2-yl hexadecanoate (–)-S23 HRMS

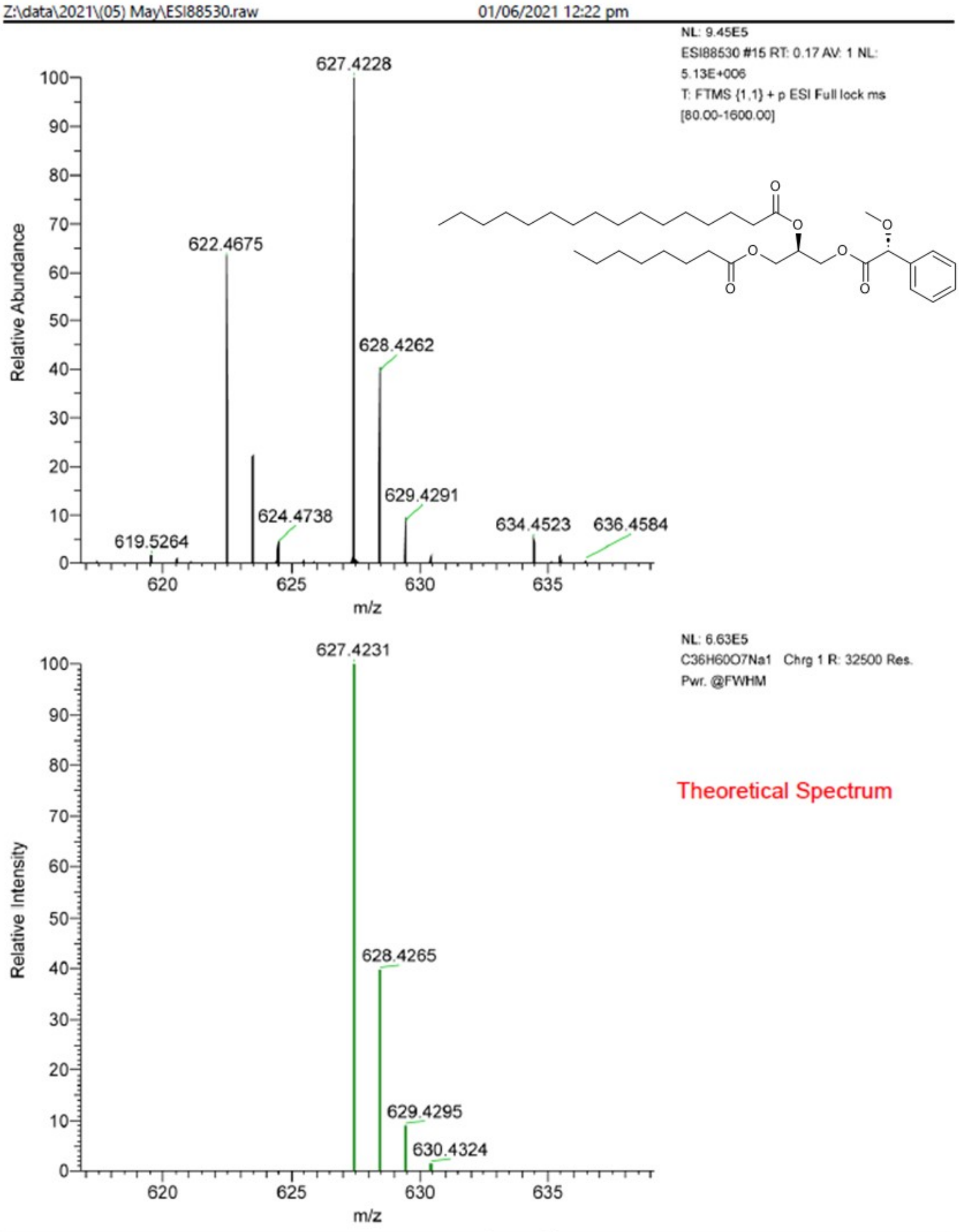

Peak Mass	Display...	Combin...	RDB	Delta (p...	Theo. m...	Rank	Combin...	# Match...	# Misse...	MS Cov....	Pattern...	MSMS...
627.4228	[illegible]	43.899	6.50	-0.53	627.42	1	97.047	4	1	100	99.877	[illegible]

(+)-(*S*)-3-(bis(4-methoxyphenyl)(phenyl)methoxy)-2-((3-(3-(dodec-11-yn-1-yl)-3*H*-diazirin-3-yl)propanoyl)oxy)propyl palmitate S24 ^{1}H NMR

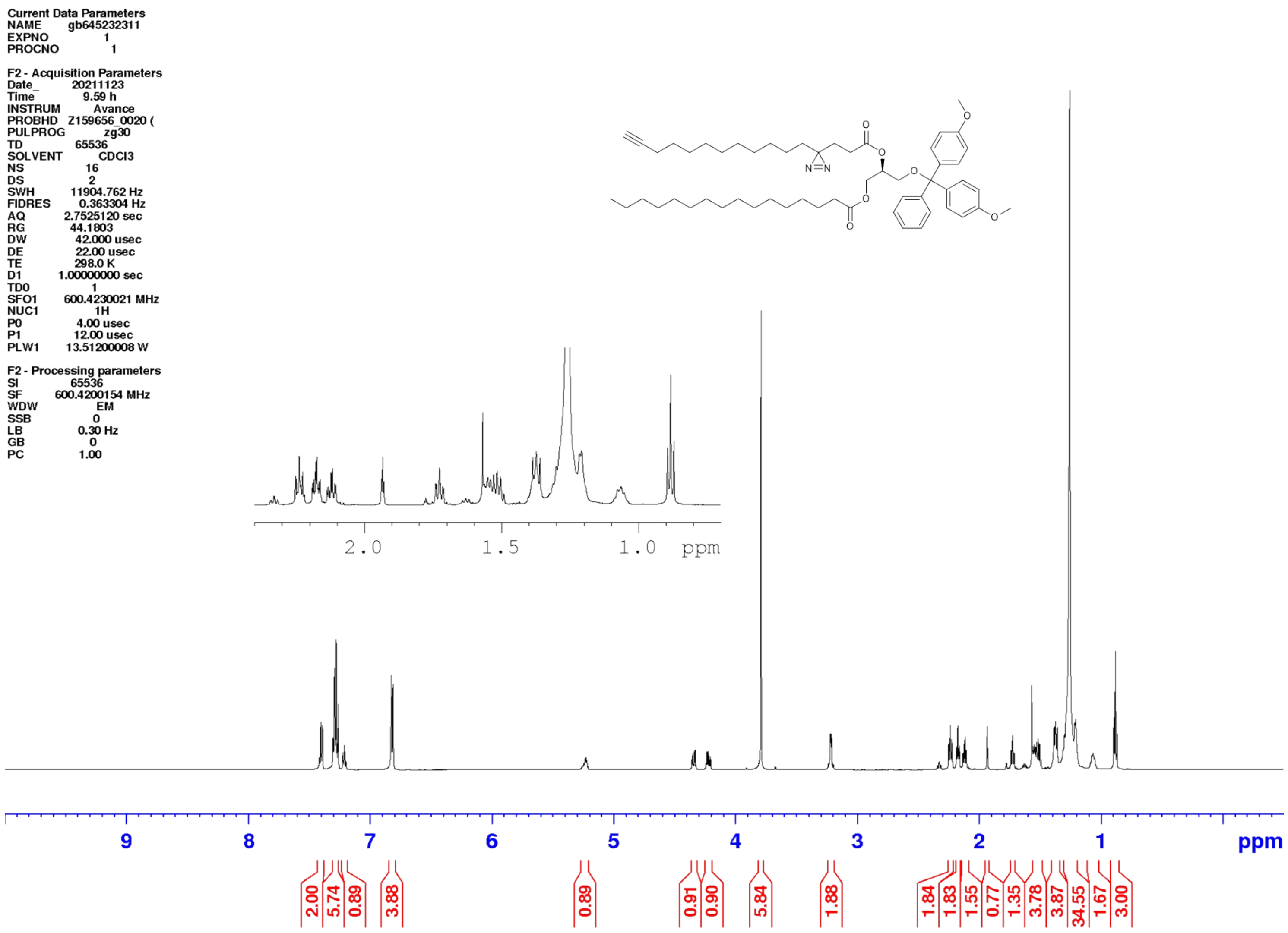

(+)-(*S*)-3-(bis(4-methoxyphenyl)(phenyl)methoxy)-2-((3-(3-(dodec-11-yn-1-yl)-3*H*-diazirin-3-yl)propanoyl)oxy)propyl palmitate S24 ^{13}C NMR

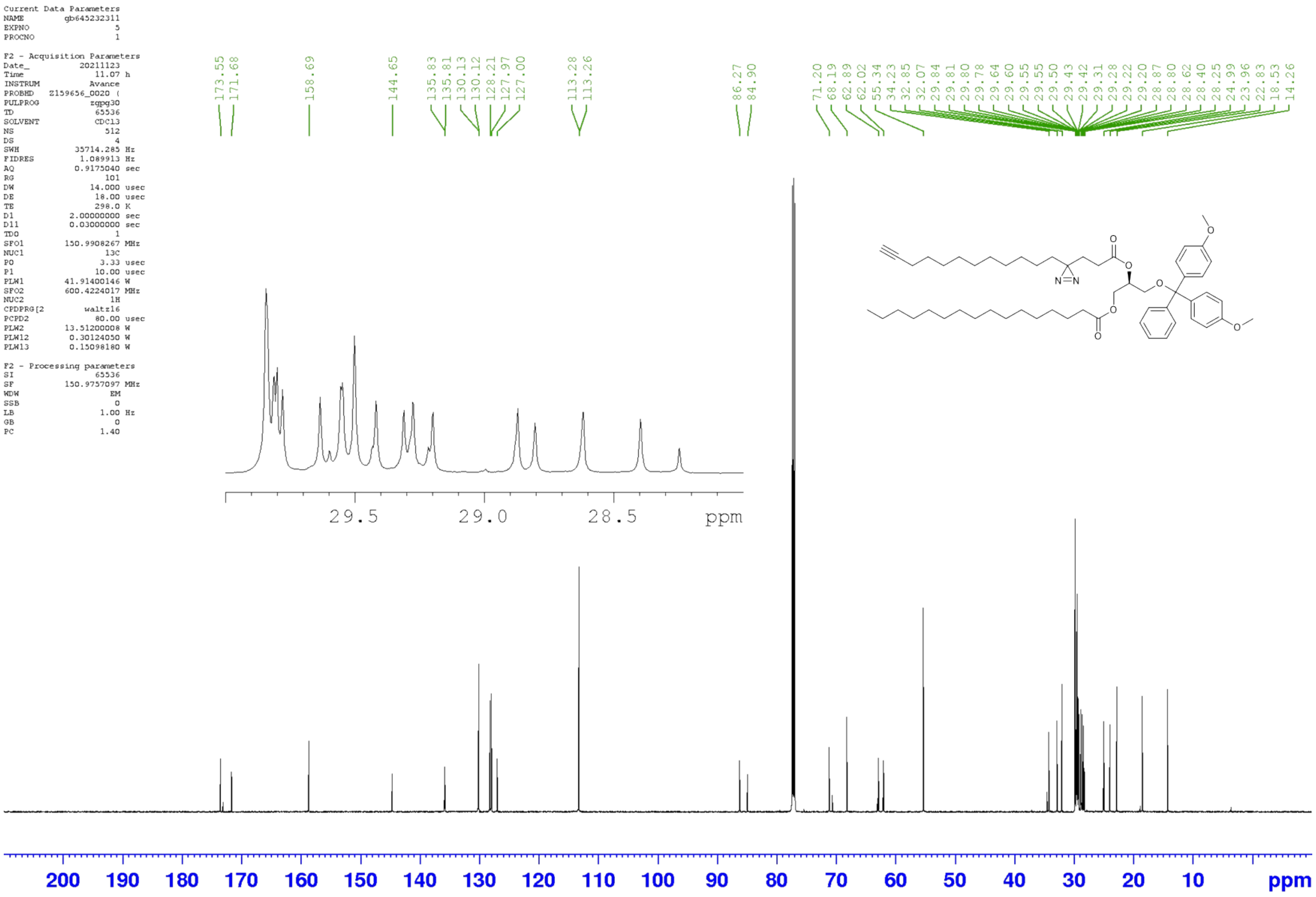

(+)-(*S*)-3-(bis(4-methoxyphenyl)(phenyl)methoxy)-2-((3-(3-(dodec-11-yn-1-yl)-3*H*-diazirin-3-yl)propanoyl)oxy)propyl palmitate S24 HRMS

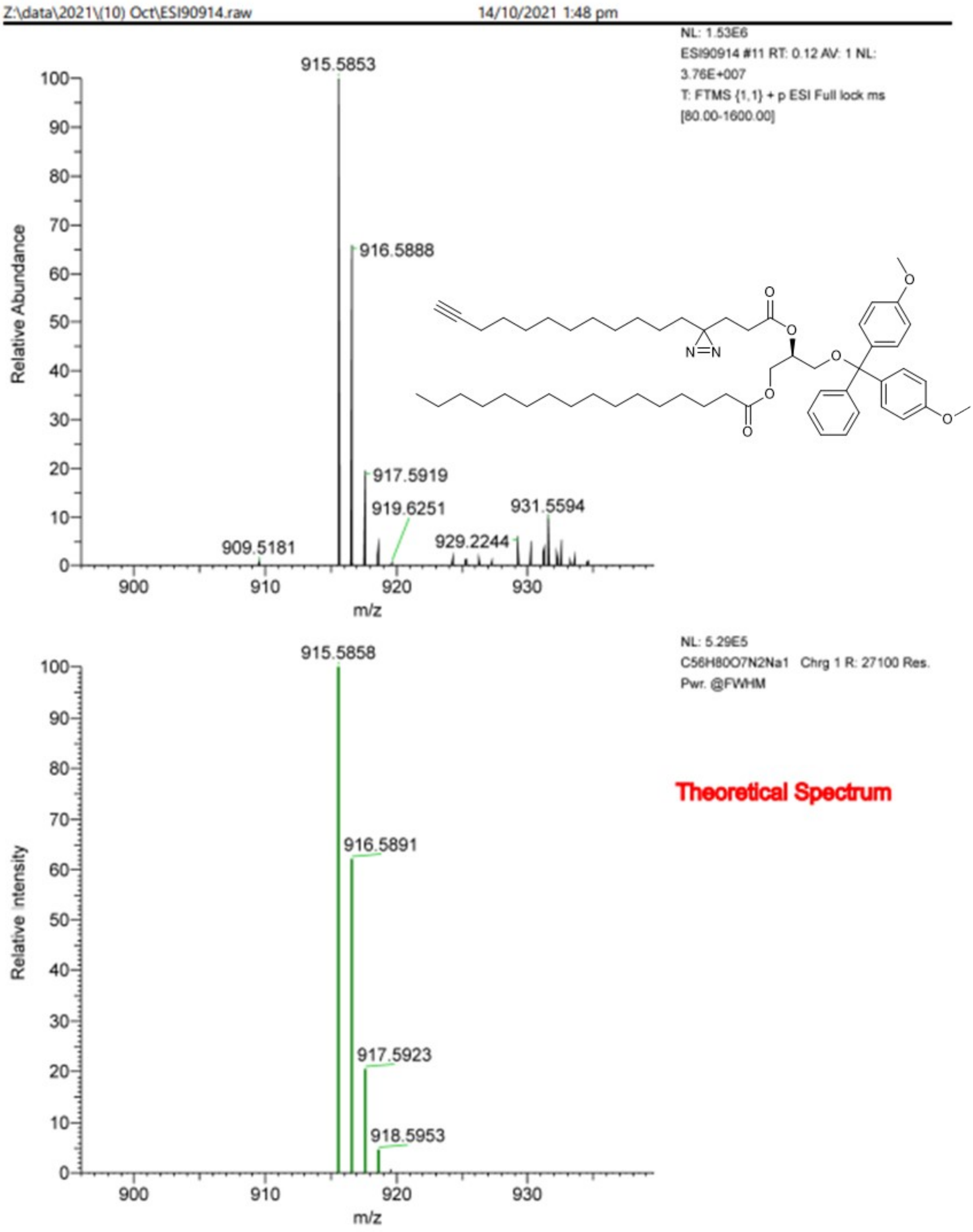

Peak Mass	Display...	Combin...	RDB	Delta (p...	Theo. m...	Rank	Combin...	# Match...	# Misse...	MS Cov...	Pattern...	MSMS...
915.5853	[illegible]	52.696	17.50	-0.49	915.58	1	97.510	4	1	100	99.580	(Collec

(*R*)-2-((3-(3-(dodec-11-yn-1-yl)-3H-diazirin-3-yl)propanoyl)oxy)-3-((*S*)-2-methoxy-2-phenylacetoxy)propyl palmitate (+)-S25 ^{1}H NMR

Current Data Parameters
NAME gb716170509 (R, R)
EXPNO 1
PROCNO 1

F2 - Acquisition Parameters
Date_ 20230905
Time 5.43 h
INSTRUM av600
PROBHD Z130037_0008 (
PULPROG zg30
TD 65536
SOLVENT CDCl3
NS 16
DS 2
SWH 12019.230 Hz
FIDRES 0.366798 Hz
AQ 2.7262976 sec
RG 60.94
DW 41.600 usec
DE 10.00 usec
TE 298.0 K
D1 1.00000000 sec
TD0 1
SFO1 600.1830009 MHz
NUC1 1H
P0 4.00 usec
P1 12.00 usec
PLW1 26.54599953 W

F2 - Processing parameters
SI 65536
SF 600.1800146 MHz
WDW EM
SSB 0
LB 0.30 Hz
GB 0
PC 1.00

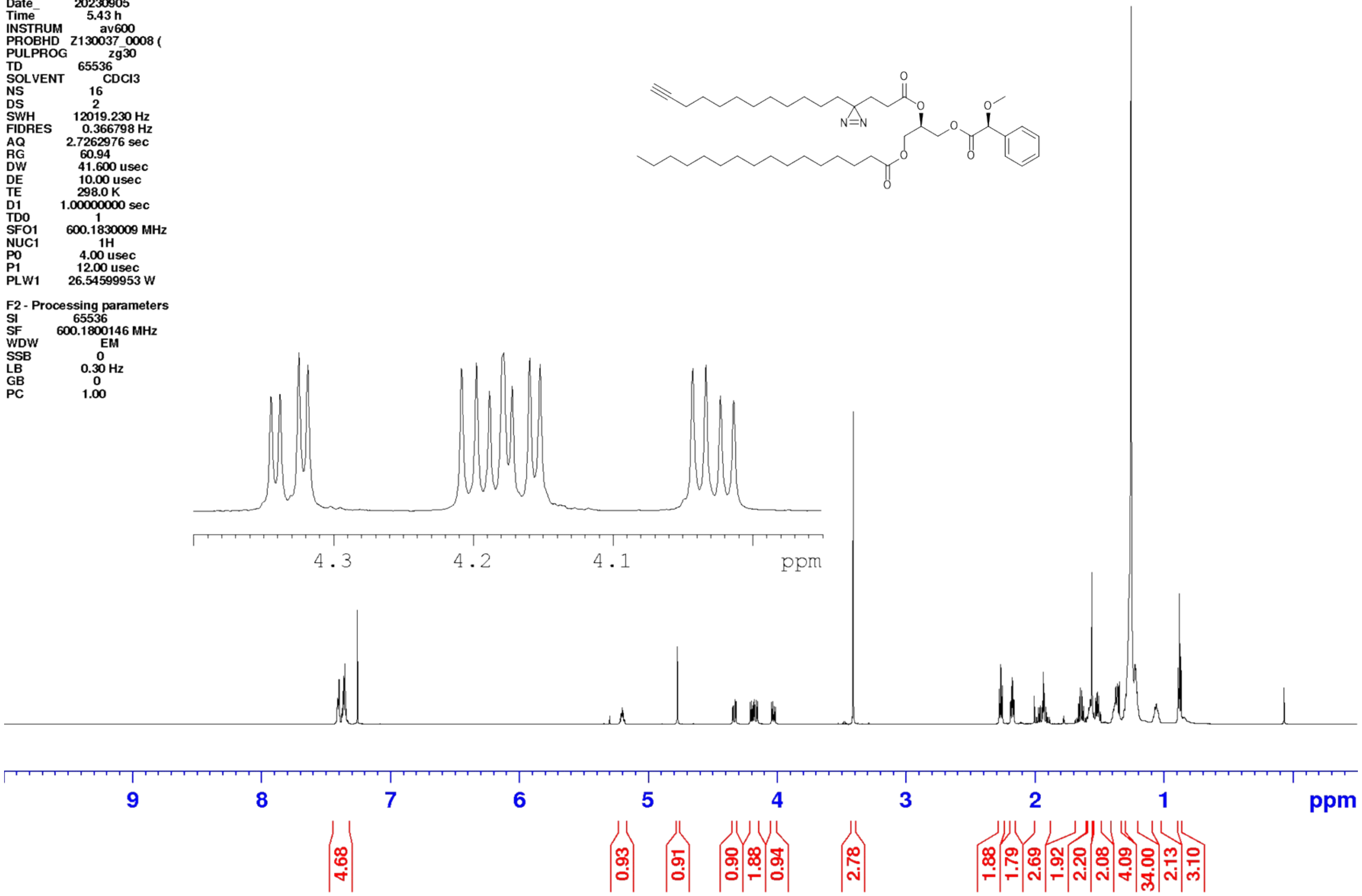

(*R*)-2-((3-(3-(dodec-11-yn-1-yl)-3H-diazirin-3-yl)propanoyl)oxy)-3-((*S*)-2-methoxy-2-phenylacetoxy)propyl palmitate (+)-S25 ^{13}C NMR

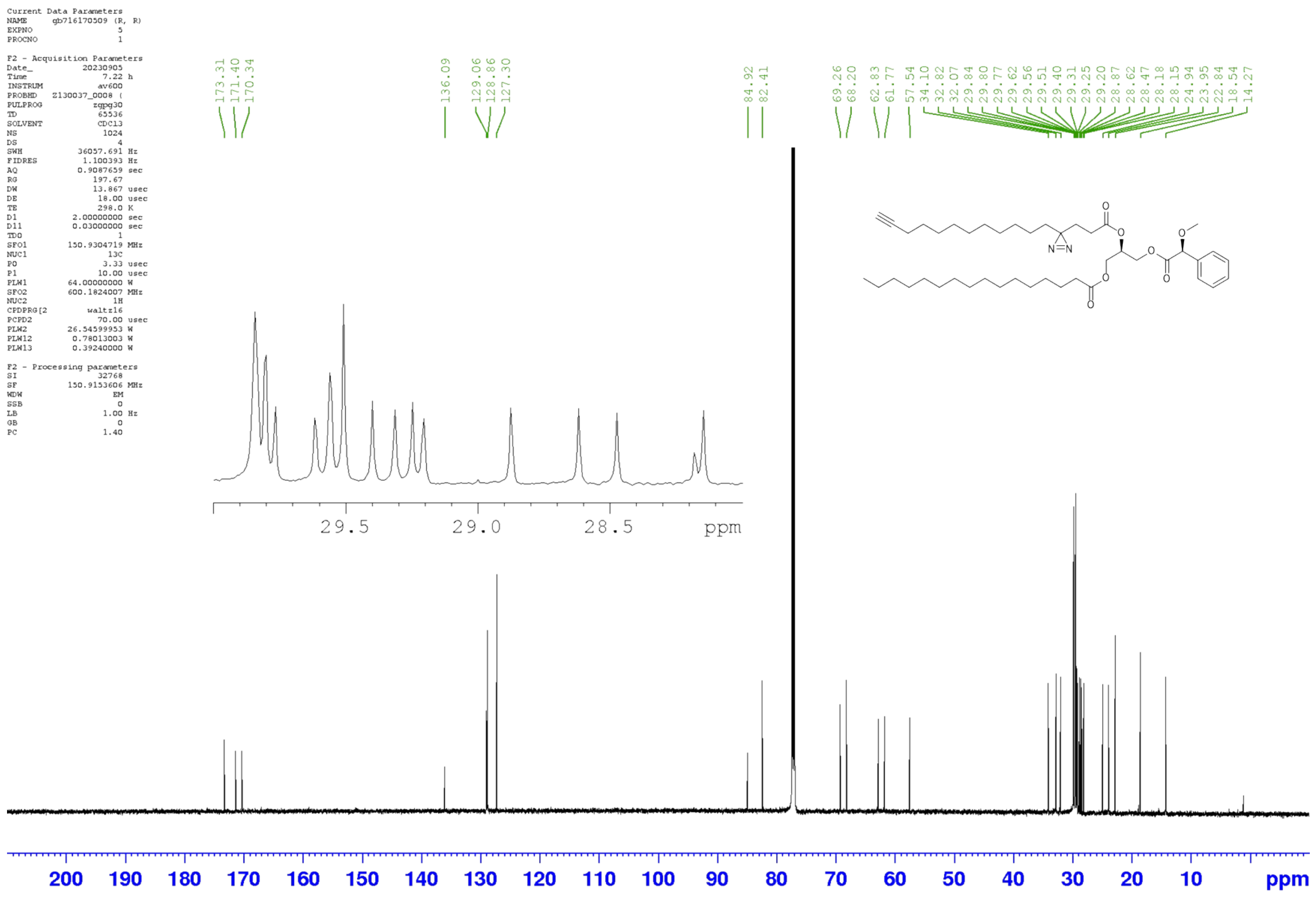

(*R*)-2-((3-(3-(dodec-11-yn-1-yl)-3H-diazirin-3-yl)propanoyl)oxy)-3-((*S*)-2-methoxy-2-phenylacetoxy)propyl palmitate (+)-S25 HRMS

Expanded Spectrum RT 0.09, NL 1248267, Peak [1], Target Mass 761.5075

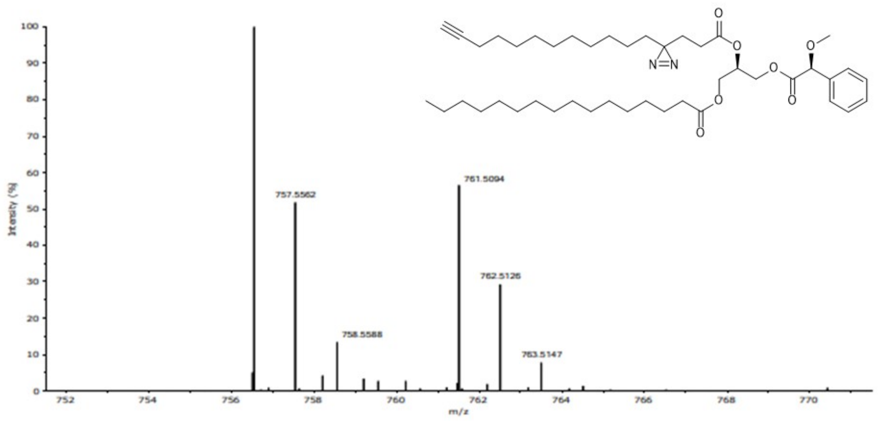

Theoretical Spectrum for C44H70N2O7Na, Minimum Abundance 0.01%

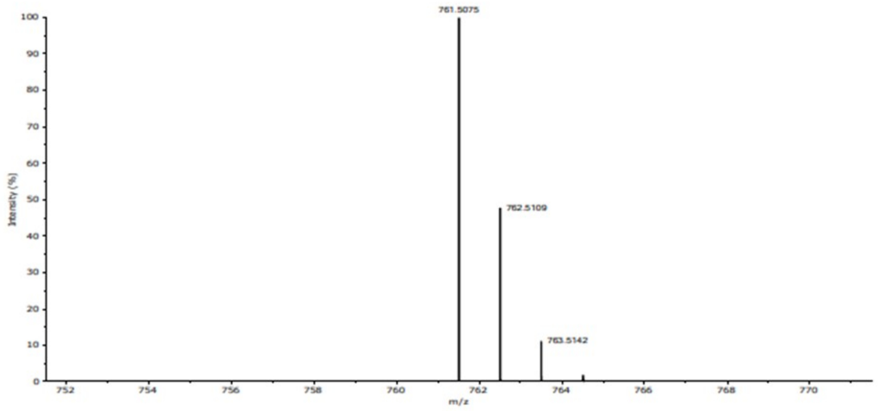

Measured Mass	Calculated Mass	Error (mDa)	Error (ppm)	Formula $[M+Na]^+$	Response
761.5094	761.5075	1.87	2.46	$C_{44}H_{70}N_2O_7Na$	348746

(*R*)-2-((3-(3-(dodec-11-yn-1-yl)-3H-diazirin-3-yl)propanoyl)oxy)-3-((*R*)-2-methoxy-2-phenylacetoxy)propyl palmitate (–)-S26 ^{1}H NMR

Current Data Parameters
NAME gb716180509 (R, S)
EXPNO 1
PROCNO 1

F2 - Acquisition Parameters
Date_ 20230905
Time 7.29 h
INSTRUM av600
PROBHD Z130037_0008 (
PULPROG zg30
TD 65536
SOLVENT CDCl3
NS 16
DS 2
SWH 12019.230 Hz
FIDRES 0.366798 Hz
AQ 2.7262976 sec
RG 60.94
DW 41.600 usec
DE 10.00 usec
TE 298.0 K
D1 1.00000000 sec
TD0 1
SFO1 600.1830009 MHz
NUC1 1H
P0 4.00 usec
P1 12.00 usec
PLW1 26.54599953 W

F2 - Processing parameters
SI 65536
SF 600.1800147 MHz
WDW EM
SSB 0
LB 0.30 Hz
GB 0
PC 1.00

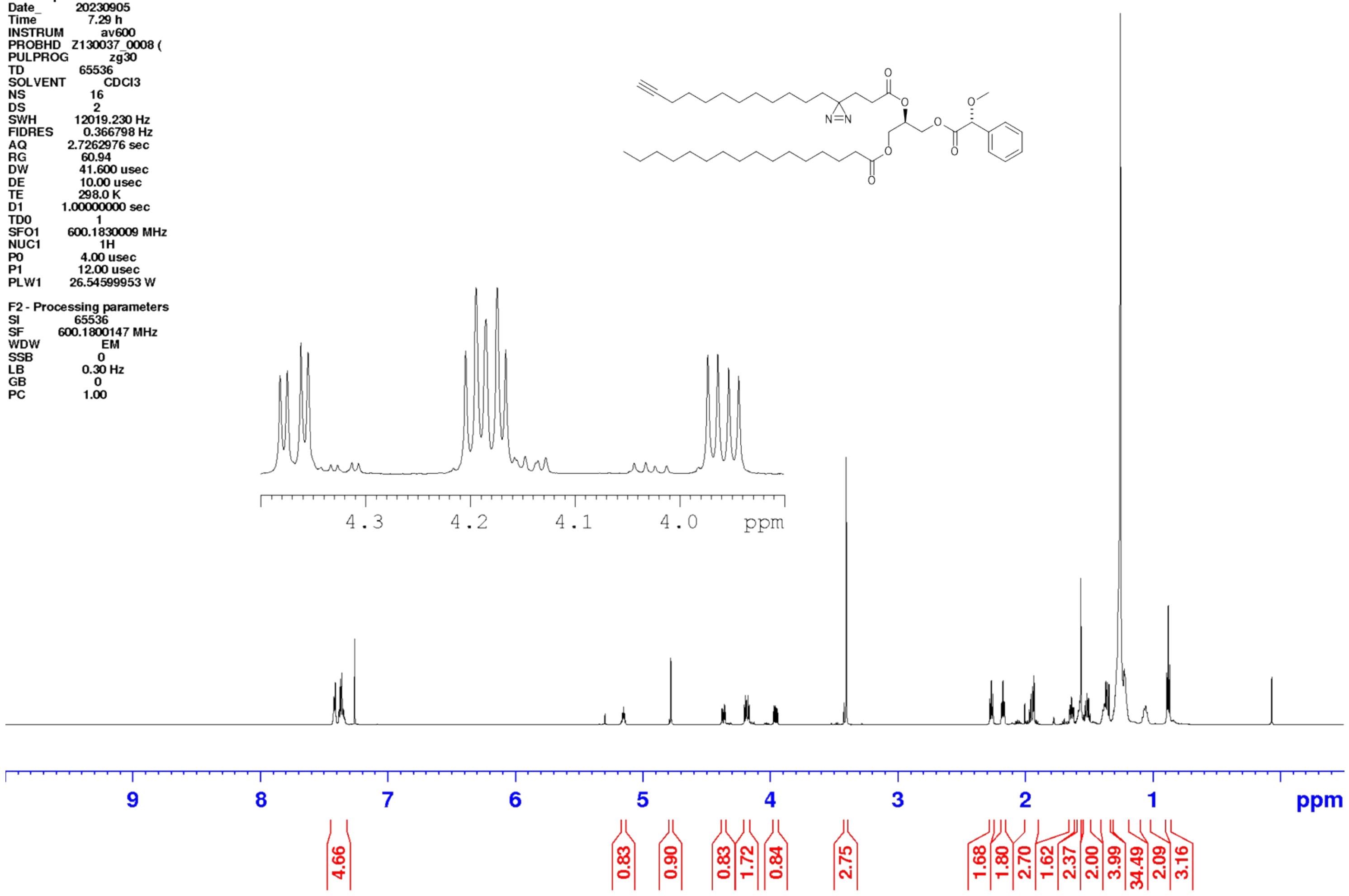

(*R*)-2-((3-(3-(dodec-11-yn-1-yl)-3H-diazirin-3-yl)propanoyl)oxy)-3-((*R*)-2-methoxy-2-phenylacetoxy)propyl palmitate (–)-S26 ^{13}C NMR

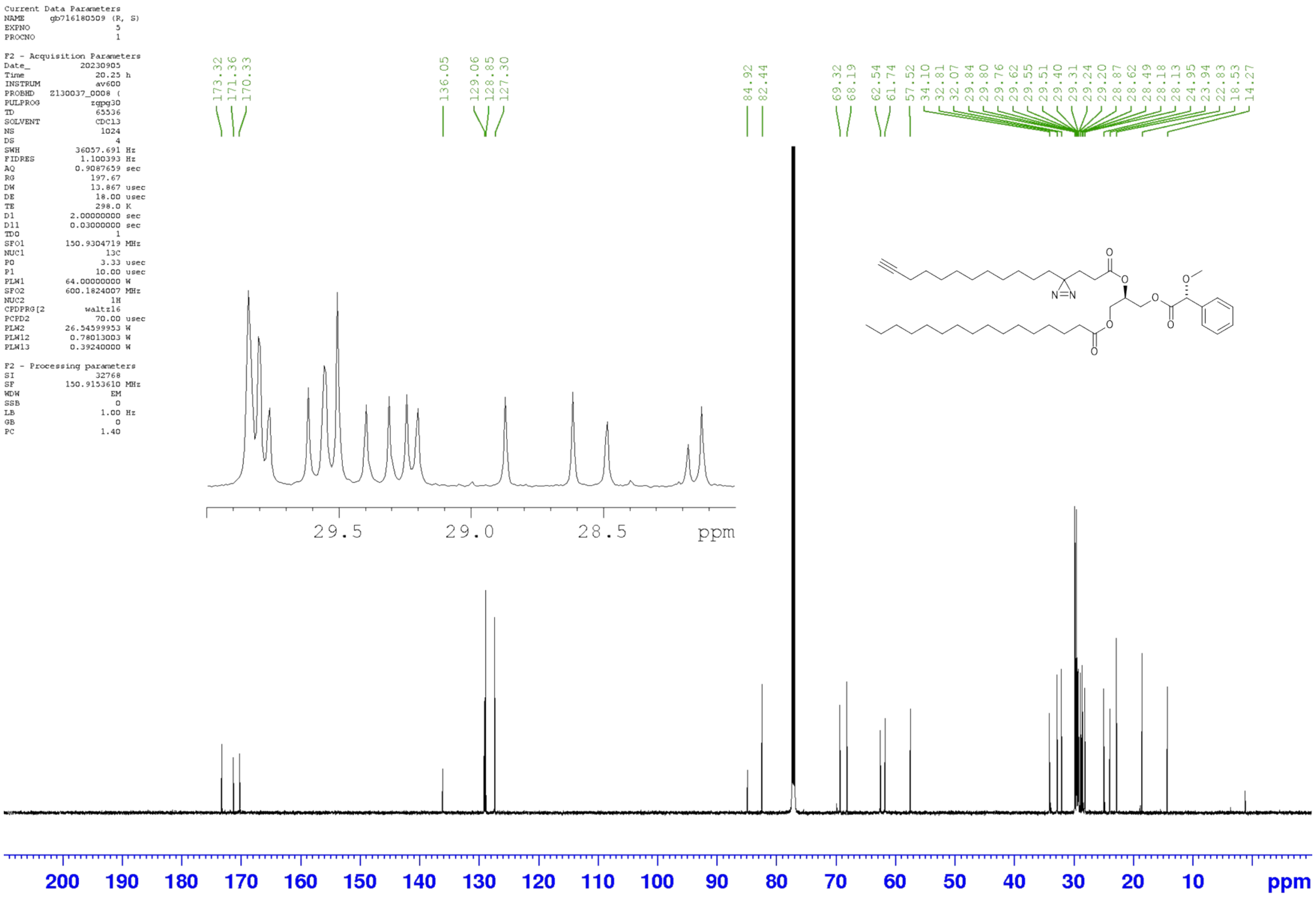

(*R*)-2-((3-(3-(dodec-11-yn-1-yl)-3H-diazirin-3-yl)propanoyl)oxy)-3-((*R*)-2-methoxy-2-phenylacetoxy)propyl palmitate (–)-S26 HRMS

Expanded Spectrum RT 0.09, NL 1347099, Peak [1], Target Mass 761.5075

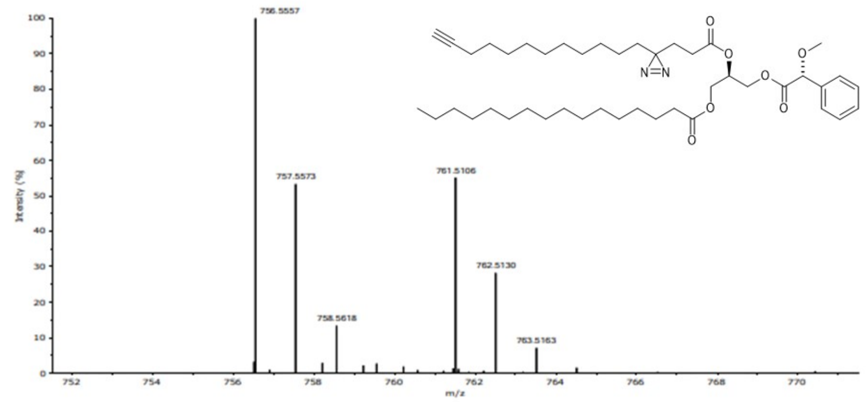

Theoretical Spectrum for C44H70N2O7Na, Minimum Abundance 0.01%

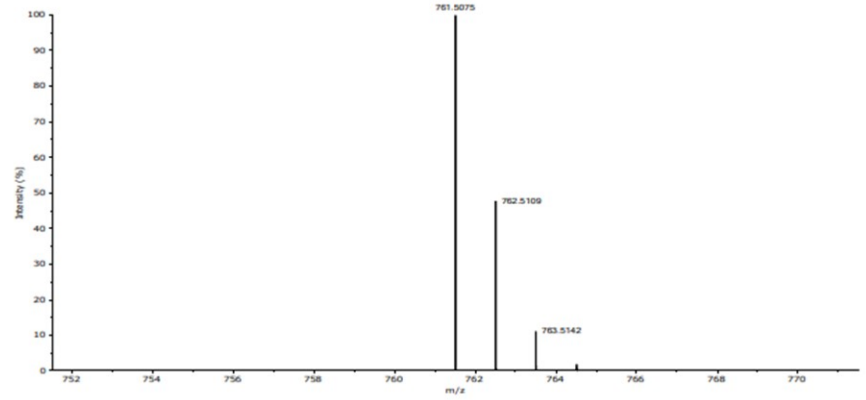

Measured Mass	Calculated Mass	Error (mDa)	Error (ppm)	Formula $[M+Na]^+$	Response
761.5106	761.5075	3.07	4.03	$C_{44}H_{70}N_2O_7Na$	375493

References

(1) Seco, J. M.; Quiñoá, E.; Riguera, R. The Assignment of Absolute Configuration by NMR. *Chem. Rev.* **2004**, *104* (1), 17–118. https://doi.org/10.1021/cr000665j.

(2) Mandal, P.; Eswara, K.; Yerkesh, Z.; Kharchenko, V.; Zandarashvili, L.; Szczepski, K.; Bensaddek, D.; Jaremko, Ł.; Black, B. E.; Fischle, W. Molecular Basis of hUHRF1 Allosteric Activation for Synergistic Histone Modification Binding by PI5P. *Sci. Adv.* **2022**, *8* (34), eabl9461. https://doi.org/10.1126/sciadv.abl9461.

(3) MacKinnon, A. L.; Taunton, J. Target Identification by Diazirine Photo-Cross-Linking and Click Chemistry. *Curr. Protoc. Chem. Biol.* **2009**, *1* (1), 55–73. https://doi.org/10.1002/9780470559277.ch090167.

(4) Fulmer, G. R.; Miller, A. J. M.; Sherden, N. H.; Gottlieb, H. E.; Nudelman, A.; Stoltz, B. M.; Bercaw, J. E.; Goldberg, K. I. NMR Chemical Shifts of Trace Impurities: Common Laboratory Solvents, Organics, and Gases in Deuterated Solvents Relevant to the Organometallic Chemist. *Organometallics* **2010**, *29* (9), 2176–2179. https://doi.org/10.1021/om100106e.

(5) M. Joffrin, A.; M. Saunders, A.; Barneda, D.; Flemington, V.; L. Thompson, A.; J. Sanganee, H.; J. Conway, S. Development of Isotope-Enriched Phosphatidylinositol-4- and 5-Phosphate Cellular Mass Spectrometry Probes. *Chem. Sci.* **2021**. https://doi.org/10.1039/D0SC06219G.

(6) Descôteaux, C.; Leblanc, V.; Bélanger, G.; Parent, S.; Asselin, É.; Bérubé, G. Improved Synthesis of Unique Estradiol-Linked Platinum(II) Complexes Showing Potent Cytocidal Activity and Affinity for the Estrogen Receptor Alpha and Beta. *Steroids* **2008**, *73* (11), 1077–1089. https://doi.org/10.1016/j.steroids.2008.04.009.

(7) Schill, G.; Schweickert, N.; Fritz, H.; Vetter, W. Synthese von [2]-Catenanen aus [2]-Rotaxanen. *Chem. Ber.* **1988**, *121* (5), 961–970. https://doi.org/10.1002/cber.19881210522.

(8) Oppolzer, W.; Radinov, R. N.; El-Sayed, E. Catalytic Asymmetric Synthesis of Macrocyclic (E)-Allylic Alcohols from ω-Alkynals via Intramolecular 1-Alkenylzinc/Aldehyde Additions. *J. Org. Chem.* **2001**, *66* (14), 4766–4770. https://doi.org/10.1021/jo000463n.

(9) Mori, K. Pheromone Synthesis. Part 253: Synthesis of the Racemates and Enantiomers of Triglycerides of Male Drosophila Fruit Flies with Special Emphasis on the Preparation of Enantiomerically Pure 1-Monoglycerides. *Tetrahedron* **2012**, *68* (40), 8441–8449. https://doi.org/10.1016/j.tet.2012.07.086.

(10) Nadler, A.; Reither, G.; Feng, S.; Stein, F.; Reither, S.; Müller, R.; Schultz, C. The Fatty Acid Composition of Diacylglycerols Determines Local Signaling Patterns. *Angew. Chem. Int. Ed.* **2013**, *52* (24), 6330–6334. https://doi.org/10.1002/anie.201301716.

(11) Forget, S. M.; Jee, A.; Smithen, D. A.; Jagdhane, R.; Anjum, S.; Beaton, S. A.; Palmer, D. R. J.; Syvitski, R. T.; Jakeman, D. L. Kinetic Evaluation of Glucose 1-Phosphate Analogues with a Thymidylyltransferase Using a Continuous Coupled Enzyme Assay. *Org. Biomol. Chem.* **2014**, *13* (3), 866–875. https://doi.org/10.1039/C4OB02057J.

(12) Laumen, K.; Ghisalba, O. Preparative-Scale Chemo-Enzymatic Synthesis of Optically Pure d-Myo–Inositol-1-Phosphate. *Biosci. Biotechnol. Biochem.* **1994**, *58* (11), 2046–2049. https://doi.org/10.1271/bbb.58.2046.
